# Supplementary figures and images for: Promoter selectivity of the RhlR quorum-sensing transcription factor receptor in Pseudomonas aeruginosa is coordinated by distinct and overlapping dependencies on C4-homoserine lactone and PqsE
Source: PLoS Genet. 2023 Dec 8;19(12):e1010900. doi: 10.1371/journal.pgen.1010900 (PMC10732425; doi:10.1371/journal.pgen.1010900)

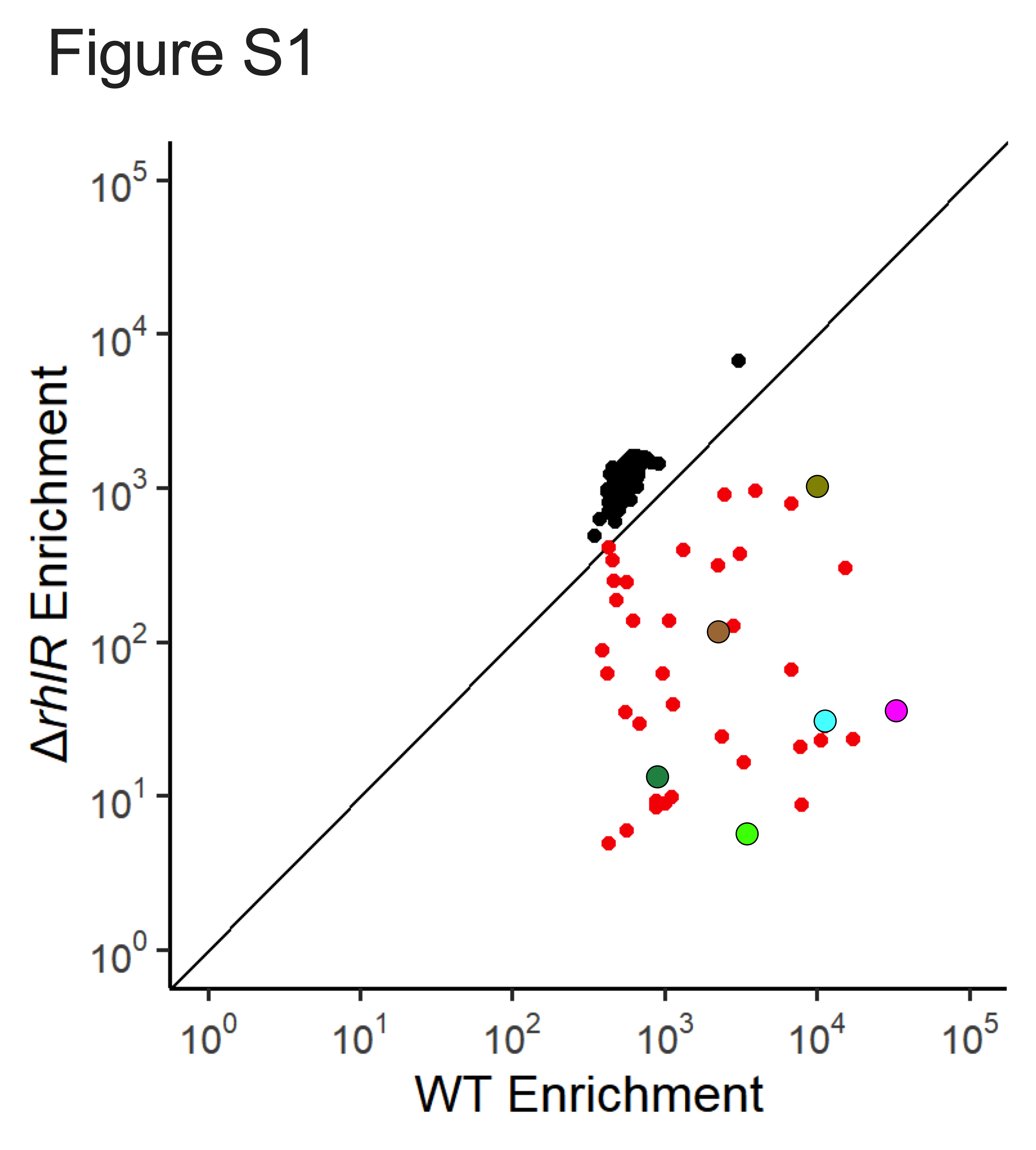

Supplement: S1 Fig — Comparison of ChIP enrichment from the WT PA14 and ΔrhlR strains. The 40 sites bound specifically by RhlR are highlighted in red. All non-specific sites are shown in black. See Table 1 for details on all RhlR specific binding sites. See S1 Table for a list of all RhlR specific and non-specific sites. (TIFF) [file pgen.1010900.s001.tiff]
