# Supplementary material for: Promoter selectivity of the RhlR quorum-sensing transcription factor receptor in Pseudomonas aeruginosa is coordinated by distinct and overlapping dependencies on C4-homoserine lactone and PqsE
Source: PLoS Genet. 2023 Dec 8;19(12):e1010900. doi: 10.1371/journal.pgen.1010900 (PMC10732425; doi:10.1371/journal.pgen.1010900)
Supplement: S1 Table — The average peak values are the average of two biological replicates and two independent ChIP experiments. Peak values were calculated as the sum of all enrichment within 100 bases upstream and downstream of the center of the ChIP peak. (DOCX) [file pgen.1010900.s004.docx]

|  | A | B | C | D | E | F | G | H |
| --- | --- | --- | --- | --- | --- | --- | --- | --- |
| 1 | **Site Location** | **WT** | **Δ*rhlR*** | **Δ*rhlI*** | **Δ*pqsE*** | **Δ*rhlI* Δ*pqsE*** | ***pqsE-* NI** | ***pqsE* (D73A)** |
| 2 | 53066 | 591.4 | 1026.9 | 580.9 | 417.0 | 331.8 | 681.8 | 507.6 |
| 3 | 54148 | 432.0 | 1241.6 | 489.9 | 311.3 | 221.4 | 776.1 | 385.1 |
| 4 | 64211 | 2437.4 | 906.1 | 962.1 | 906.8 | 337.2 | 779.4 | 1647.0 |
| 5 | 139733 | 2364.4 | 24.5 | 2111.9 | 640.1 | 82.2 | 501.9 | 1509.8 |
| 6 | 560072 | 1010.4 | 8.9 | 1461.2 | 596.4 | 238.6 | 597.8 | 686.4 |
| 7 | 711431 | 465.4 | 751.6 | 588.4 | 415.7 | 315.1 | 644.9 | 441.2 |
| 8 | 711675 | 550.1 | 952.4 | 657.0 | 410.0 | 267.6 | 627.3 | 535.0 |
| 9 | 733156 | 600.0 | 1105.2 | 758.7 | 683.3 | 505.2 | 836.8 | 598.4 |
| 10 | 736253 | 598.9 | 838.4 | 660.0 | 615.1 | 424.6 | 726.3 | 638.4 |
| 11 | 736928 | 660.5 | 1160.2 | 873.9 | 724.1 | 517.1 | 920.8 | 671.3 |
| 12 | 737180 | 555.3 | 923.5 | 663.7 | 592.1 | 363.0 | 717.8 | 592.4 |
| 13 | 754615 | 552.9 | 35.1 | 144.2 | 194.2 | 253.8 | 164.9 | 341.4 |
| 14 | 812427 | 3931.3 | 966.2 | 3353.5 | 1641.1 | 263.2 | 1602.2 | 2375.6 |
| 15 | 813529 | 10446.1 | 995.9 | 5496.0 | 1676.3 | 352.0 | 1598.7 | 5131.9 |
| 16 | 895433 | 6809.1 | 800.8 | 794.7 | 1376.8 | 157.7 | 792.7 | 3790.1 |
| 17 | 898184 | 465.6 | 1088.9 | 613.8 | 470.2 | 210.5 | 686.5 | 418.8 |
| 18 | 956560 | 568.5 | 931.5 | 832.7 | 458.8 | 297.1 | 561.5 | 471.9 |
| 19 | 1195684 | 735.5 | 1580.2 | 900.4 | 713.5 | 390.2 | 876.1 | 666.6 |
| 20 | 1195963 | 599.2 | 1305.3 | 862.5 | 698.3 | 399.1 | 995.2 | 575.3 |
| 21 | 1231146 | 630.4 | 1040.8 | 668.5 | 508.8 | 282.5 | 819.9 | 570.8 |
| 22 | 1372370 | 6758.9 | 66.6 | 1152.0 | 1914.7 | 183.9 | 1534.5 | 5258.8 |
| 23 | 1387361 | 878.9 | 9.4 | 296.7 | 605.7 | 42.0 | 404.6 | 796.4 |
| 24 | 1620628 | 1315.4 | 395.0 | 629.5 | 863.9 | 174.4 | 831.3 | 1366.9 |
| 25 | 1621028 | 481.3 | 189.0 | 257.6 | 481.7 | 207.4 | 419.2 | 428.9 |
| 26 | 1635342 | 521.0 | 792.8 | 550.4 | 429.5 | 382.3 | 657.8 | 505.1 |
| 27 | 1648391 | 11271.2 | 28.5 | 2586.2 | 7736.8 | 460.1 | 5875.2 | 7577.1 |
| 28 | 1651804 | 1112.2 | 9.8 | 1021.5 | 1080.2 | 247.9 | 827.1 | 553.6 |
| 29 | 1735823 | 414.7 | 62.4 | 233.3 | 307.4 | 2048.2 | 295.2 | 437.7 |
| 30 | 1762030 | 518.2 | 967.0 | 657.8 | 498.6 | 239.7 | 743.8 | 460.9 |
| 31 | 1765892 | 589.0 | 1008.6 | 564.8 | 487.3 | 390.8 | 742.2 | 587.8 |
| 32 | 1766203 | 526.7 | 971.0 | 522.2 | 421.4 | 240.5 | 744.5 | 452.3 |
| 33 | 1767099 | 510.0 | 913.0 | 624.9 | 431.1 | 338.3 | 658.8 | 520.3 |
| 34 | 1774336 | 32231.7 | 35.0 | 33814.6 | 577.6 | 45.8 | 259.5 | 22562.8 |
| 35 | 1816906 | 15193.0 | 301.7 | 6827.0 | 16297.0 | 3344.4 | 10539.4 | 8477.9 |
| 36 | 1861853 | 517.0 | 994.2 | 662.6 | 522.7 | 310.2 | 763.8 | 467.5 |
| 37 | 1863055 | 461.7 | 860.8 | 562.3 | 388.6 | 208.2 | 677.0 | 430.4 |
| 38 | 1863312 | 690.9 | 1403.3 | 761.5 | 549.9 | 360.8 | 876.9 | 674.6 |
| 39 | 1921598 | 457.3 | 252.1 | 195.7 | 267.8 | 1086.3 | 379.8 | 385.2 |
| 40 | 1924465 | 390.1 | 89.1 | 142.1 | 242.1 | 1145.9 | 269.9 | 331.7 |
| 41 | 1925890 | 527.8 | 1209.1 | 699.9 | 610.6 | 402.1 | 800.7 | 513.3 |
| 42 | 1928093 | 595.5 | 1119.2 | 741.9 | 621.9 | 326.3 | 831.7 | 544.0 |
| 43 | 1928610 | 656.2 | 1466.2 | 862.6 | 672.8 | 372.0 | 942.0 | 666.3 |
| 44 | 1929125 | 486.8 | 1127.1 | 738.4 | 564.4 | 268.1 | 752.7 | 480.4 |
| 45 | 1929320 | 590.3 | 1307.9 | 868.1 | 711.2 | 327.9 | 862.6 | 627.4 |
| 46 | 1929423 | 550.5 | 1285.7 | 838.0 | 743.3 | 295.5 | 957.6 | 560.2 |
| 47 | 1931650 | 648.3 | 1276.1 | 759.1 | 660.1 | 267.7 | 884.6 | 654.3 |
| 48 | 1931828 | 617.5 | 1335.5 | 789.2 | 653.1 | 301.5 | 980.3 | 598.2 |
| 49 | 1931996 | 579.0 | 1157.7 | 767.1 | 667.7 | 239.4 | 919.0 | 531.3 |
| 50 | 1932145 | 519.1 | 1207.3 | 792.9 | 659.3 | 223.6 | 755.9 | 543.3 |
| 51 | 1932413 | 643.3 | 1339.9 | 908.4 | 729.8 | 281.7 | 1026.5 | 626.6 |
| 52 | 1933063 | 501.9 | 1141.3 | 731.7 | 567.3 | 219.3 | 853.5 | 496.4 |
| 53 | 1934437 | 639.6 | 1469.1 | 796.6 | 695.6 | 223.9 | 981.2 | 549.4 |
| 54 | 1934549 | 577.3 | 1433.1 | 717.8 | 678.2 | 199.3 | 981.4 | 569.0 |
| 55 | 1938348 | 666.5 | 1244.9 | 749.0 | 653.2 | 256.2 | 921.3 | 621.8 |
| 56 | 1938815 | 510.9 | 1226.5 | 657.5 | 535.0 | 239.3 | 813.3 | 513.2 |
| 57 | 1939178 | 545.9 | 1050.0 | 654.6 | 629.8 | 317.1 | 833.8 | 512.3 |
| 58 | 2027979 | 581.5 | 1020.4 | 722.0 | 592.9 | 398.2 | 830.4 | 561.1 |

|  | A | B | C | D | E | F | G | H |
| --- | --- | --- | --- | --- | --- | --- | --- | --- |
| 59 | 2028457 | 472.6 | 1284.7 | 717.8 | 592.8 | 253.4 | 832.0 | 500.2 |
| 60 | 2028987 | 463.8 | 987.5 | 803.7 | 535.8 | 217.1 | 714.2 | 438.4 |
| 61 | 2029197 | 578.2 | 1186.1 | 873.7 | 563.8 | 312.2 | 860.7 | 537.6 |
| 62 | 2029507 | 534.7 | 1124.4 | 722.7 | 571.1 | 259.9 | 702.4 | 480.5 |
| 63 | 2029944 | 504.1 | 948.0 | 766.5 | 504.7 | 275.9 | 608.9 | 489.6 |
| 64 | 2030348 | 493.6 | 960.2 | 558.0 | 453.2 | 295.4 | 766.6 | 490.7 |
| 65 | 2030753 | 812.0 | 1477.7 | 819.3 | 520.3 | 374.5 | 1053.9 | 784.0 |
| 66 | 2031255 | 772.7 | 1565.8 | 737.5 | 524.6 | 295.5 | 1107.4 | 723.0 |
| 67 | 2031678 | 535.7 | 1305.9 | 618.6 | 457.1 | 374.2 | 964.1 | 478.1 |
| 68 | 2032697 | 608.6 | 1623.3 | 651.2 | 451.6 | 482.2 | 1345.6 | 551.5 |
| 69 | 2034193 | 541.8 | 1064.3 | 650.6 | 529.4 | 294.1 | 883.0 | 504.3 |
| 70 | 2035624 | 554.3 | 1141.3 | 775.0 | 649.4 | 298.8 | 894.6 | 592.9 |
| 71 | 2035849 | 489.7 | 973.7 | 683.2 | 587.5 | 224.5 | 798.6 | 470.0 |
| 72 | 2036100 | 552.9 | 1087.9 | 619.3 | 519.2 | 183.5 | 688.9 | 507.9 |
| 73 | 2036498 | 515.4 | 1045.3 | 587.0 | 478.0 | 248.7 | 731.6 | 438.9 |
| 74 | 2037512 | 515.9 | 1078.0 | 699.7 | 627.9 | 225.3 | 693.9 | 480.9 |
| 75 | 2039630 | 612.3 | 1048.8 | 677.9 | 556.4 | 262.3 | 824.8 | 550.3 |
| 76 | 2040895 | 517.2 | 1189.2 | 730.0 | 585.3 | 330.4 | 827.3 | 496.6 |
| 77 | 2041043 | 596.3 | 1237.6 | 762.7 | 606.4 | 361.8 | 874.2 | 590.4 |
| 78 | 2422185 | 655.4 | 1030.1 | 812.0 | 544.5 | 349.5 | 829.2 | 601.5 |
| 79 | 2444398 | 885.1 | 8.4 | 166.8 | 321.1 | 27.9 | 170.7 | 736.4 |
| 80 | 2477339 | 526.0 | 983.5 | 634.5 | 493.6 | 243.2 | 703.7 | 526.3 |
| 81 | 2477468 | 510.3 | 890.2 | 601.2 | 447.9 | 376.6 | 585.4 | 533.6 |
| 82 | 2478717 | 494.3 | 727.6 | 611.8 | 478.8 | 265.3 | 611.9 | 481.8 |
| 83 | 2478901 | 547.5 | 947.3 | 808.1 | 684.8 | 334.3 | 868.2 | 544.1 |
| 84 | 2568048 | 7797.7 | 20.9 | 1602.7 | 1780.0 | 283.8 | 1939.8 | 7130.4 |
| 85 | 2647472 | 2294.2 | 118.2 | 1094.3 | 3484.9 | 761.6 | 2963.5 | 1546.8 |
| 86 | 2677542 | 17187.9 | 23.2 | 9811.6 | 90.7 | 136.7 | 53.7 | 11243.3 |
| 87 | 2721761 | 431.0 | 410.1 | 315.4 | 278.4 | 168.4 | 382.9 | 420.0 |
| 88 | 2859603 | 492.7 | 721.2 | 462.9 | 365.8 | 263.7 | 481.8 | 433.7 |
| 89 | 2866123 | 623.9 | 1320.1 | 791.0 | 597.3 | 221.5 | 837.6 | 635.8 |
| 90 | 2867864 | 592.6 | 1287.8 | 529.8 | 454.3 | 178.9 | 788.5 | 539.2 |
| 91 | 2870549 | 587.3 | 1546.6 | 704.2 | 616.3 | 363.9 | 935.2 | 577.3 |
| 92 | 2870709 | 601.0 | 1377.3 | 696.8 | 585.0 | 303.8 | 980.6 | 586.9 |
| 93 | 2870815 | 603.2 | 1120.5 | 642.0 | 528.4 | 293.8 | 811.4 | 513.0 |
| 94 | 2910738 | 347.0 | 487.4 | 210.6 | 248.9 | 387.6 | 308.4 | 427.6 |
| 95 | 3174873 | 533.9 | 1024.4 | 616.2 | 444.4 | 286.6 | 705.2 | 510.8 |
| 96 | 3175027 | 602.4 | 1068.5 | 585.8 | 443.2 | 216.5 | 776.6 | 567.7 |
| 97 | 3181581 | 538.8 | 1246.5 | 806.2 | 670.6 | 324.4 | 993.3 | 564.8 |
| 98 | 3182110 | 497.4 | 941.3 | 642.8 | 480.5 | 301.3 | 725.2 | 493.6 |
| 99 | 3183400 | 576.6 | 1201.2 | 590.0 | 570.8 | 251.6 | 907.1 | 563.0 |
| 100 | 3183679 | 621.3 | 1317.2 | 768.4 | 635.1 | 344.1 | 1025.3 | 669.8 |
| 101 | 3183968 | 544.1 | 1154.4 | 827.1 | 653.8 | 252.0 | 890.3 | 525.4 |
| 102 | 3184251 | 513.4 | 998.2 | 670.4 | 537.1 | 248.2 | 845.0 | 479.8 |
| 103 | 3188223 | 556.8 | 243.6 | 258.0 | 339.1 | 969.3 | 259.0 | 338.6 |
| 104 | 3236436 | 3335.0 | 5.4 | 4885.0 | 342.1 | 111.0 | 274.7 | 2999.7 |
| 105 | 3364761 | 423.7 | 4.9 | 37.0 | 112.0 | 18.8 | 94.0 | 343.8 |
| 106 | 3405263 | 478.9 | 973.4 | 584.7 | 494.4 | 217.8 | 672.4 | 468.5 |
| 107 | 3405951 | 597.1 | 1047.7 | 719.8 | 495.3 | 337.0 | 835.6 | 540.4 |
| 108 | 3407018 | 492.6 | 871.5 | 669.0 | 527.5 | 262.0 | 772.4 | 482.6 |
| 109 | 3512549 | 509.6 | 1282.9 | 644.9 | 510.6 | 223.0 | 1051.2 | 447.9 |
| 110 | 3515027 | 451.6 | 964.7 | 581.2 | 543.1 | 230.4 | 629.1 | 495.3 |
| 111 | 3561157 | 453.4 | 340.6 | 336.5 | 420.0 | 174.7 | 388.6 | 392.9 |
| 112 | 3561969 | 2238.4 | 314.8 | 348.0 | 1218.8 | 106.0 | 1093.1 | 1287.0 |
| 113 | 3600666 | 555.5 | 6.0 | 206.6 | 277.4 | 35.8 | 205.1 | 517.6 |
| 114 | 3831541 | 3119.5 | 373.5 | 629.0 | 1767.9 | 169.5 | 1056.1 | 1815.4 |
| 115 | 4133306 | 470.6 | 1013.1 | 639.9 | 523.3 | 214.9 | 689.6 | 424.9 |
| 116 | 4134459 | 452.7 | 880.3 | 706.6 | 521.6 | 275.4 | 681.2 | 459.7 |

|  | A | B | C | D | E | F | G | H |
| --- | --- | --- | --- | --- | --- | --- | --- | --- |
| 117 | 4134700 | 509.7 | 1241.0 | 808.8 | 708.4 | 348.4 | 870.5 | 537.9 |
| 118 | 4285523 | 1059.8 | 136.6 | 272.9 | 405.7 | 99.7 | 359.3 | 826.5 |
| 119 | 4313855 | 2800.5 | 127.7 | 2645.5 | 2597.9 | 556.1 | 2295.9 | 1970.4 |
| 120 | 4314560 | 620.4 | 137.3 | 651.9 | 974.9 | 377.1 | 885.9 | 416.6 |
| 121 | 4354082 | 528.0 | 1369.7 | 703.9 | 482.6 | 224.5 | 1015.7 | 483.8 |
| 122 | 4354199 | 498.2 | 1302.6 | 634.3 | 450.2 | 237.1 | 898.9 | 466.0 |
| 123 | 4355373 | 659.5 | 1611.5 | 755.9 | 594.2 | 280.1 | 1203.2 | 657.3 |
| 124 | 4382169 | 1136.8 | 39.3 | 1398.4 | 880.7 | 232.5 | 735.6 | 979.2 |
| 125 | 4400553 | 613.8 | 1442.0 | 750.1 | 518.2 | 302.6 | 1118.8 | 567.3 |
| 126 | 4400847 | 613.1 | 1405.4 | 897.9 | 578.3 | 363.1 | 1110.2 | 538.0 |
| 127 | 4402913 | 456.2 | 1352.4 | 588.0 | 452.5 | 221.7 | 883.4 | 430.6 |
| 128 | 4405414 | 534.5 | 1072.4 | 659.1 | 573.4 | 223.7 | 808.5 | 465.3 |
| 129 | 4425570 | 7939.2 | 8.7 | 3189.2 | 6100.9 | 393.9 | 3470.3 | 4751.6 |
| 130 | 4576933 | 428.1 | 811.0 | 568.0 | 444.9 | 253.4 | 654.0 | 423.0 |
| 131 | 4587198 | 543.3 | 963.7 | 557.5 | 486.1 | 233.3 | 700.2 | 486.4 |
| 132 | 4587495 | 631.1 | 1261.4 | 651.1 | 537.6 | 231.8 | 914.8 | 587.4 |
| 133 | 4752954 | 563.2 | 1294.7 | 650.9 | 537.6 | 230.7 | 950.3 | 554.1 |
| 134 | 4753616 | 539.0 | 1431.8 | 707.2 | 507.9 | 343.5 | 963.8 | 515.3 |
| 135 | 4754176 | 578.5 | 1480.2 | 723.0 | 586.9 | 297.5 | 1001.7 | 523.9 |
| 136 | 4754652 | 609.7 | 1406.1 | 706.1 | 560.8 | 355.0 | 929.2 | 559.5 |
| 137 | 4754878 | 632.4 | 1389.6 | 738.0 | 544.3 | 337.7 | 908.1 | 595.3 |
| 138 | 4755307 | 580.7 | 1512.1 | 738.7 | 475.1 | 294.5 | 1001.9 | 564.9 |
| 139 | 4755608 | 414.9 | 951.9 | 461.7 | 381.2 | 143.4 | 699.8 | 406.7 |
| 140 | 5159548 | 881.2 | 12.7 | 236.3 | 106.4 | 62.3 | 66.0 | 558.3 |
| 141 | 5219733 | 912.2 | 1442.4 | 534.7 | 541.4 | 635.0 | 879.4 | 910.6 |
| 142 | 5233092 | 662.5 | 1203.9 | 746.0 | 614.7 | 306.3 | 819.3 | 610.2 |
| 143 | 5236558 | 491.5 | 1006.2 | 606.4 | 488.4 | 220.0 | 777.4 | 445.1 |
| 144 | 5236864 | 471.7 | 781.1 | 525.1 | 490.1 | 214.4 | 540.0 | 380.3 |
| 145 | 5236985 | 487.4 | 762.7 | 493.8 | 454.9 | 237.7 | 499.6 | 409.2 |
| 146 | 5268881 | 679.5 | 29.4 | 247.1 | 185.2 | 60.9 | 150.2 | 566.6 |
| 147 | 5270523 | 434.3 | 693.4 | 476.1 | 374.1 | 211.7 | 496.6 | 377.5 |
| 148 | 5275584 | 632.2 | 1362.5 | 740.9 | 609.0 | 386.2 | 944.8 | 619.3 |
| 149 | 5276703 | 465.9 | 1107.3 | 905.5 | 558.8 | 241.1 | 742.3 | 452.2 |
| 150 | 5291283 | 439.0 | 915.8 | 578.8 | 470.8 | 255.2 | 623.9 | 443.2 |
| 151 | 5300407 | 536.5 | 1074.0 | 588.7 | 466.1 | 254.4 | 837.3 | 528.0 |
| 152 | 5300796 | 654.3 | 1528.1 | 733.0 | 577.7 | 269.5 | 961.0 | 644.1 |
| 153 | 5301027 | 521.8 | 1146.9 | 568.7 | 524.4 | 225.8 | 799.6 | 498.9 |
| 154 | 5305066 | 596.3 | 1400.2 | 761.5 | 579.3 | 320.6 | 870.8 | 596.4 |
| 155 | 5330676 | 557.9 | 1243.0 | 762.2 | 521.6 | 332.2 | 947.2 | 521.0 |
| 156 | 5331461 | 598.2 | 1245.1 | 941.0 | 679.1 | 373.4 | 919.2 | 548.3 |
| 157 | 5331638 | 611.9 | 1201.4 | 852.4 | 648.7 | 398.2 | 794.7 | 540.9 |
| 158 | 5352651 | 427.5 | 707.7 | 483.5 | 440.1 | 218.4 | 490.2 | 381.7 |
| 159 | 5352994 | 525.2 | 1181.7 | 681.5 | 637.8 | 203.9 | 882.8 | 482.3 |
| 160 | 5403392 | 3058.5 | 6709.5 | 3102.8 | 3161.8 | 4584.5 | 3222.5 | 2620.0 |
| 161 | 5414219 | 967.4 | 62.9 | 508.3 | 620.9 | 186.0 | 644.1 | 1090.7 |
| 162 | 5515082 | 369.2 | 632.3 | 186.3 | 225.1 | 165.8 | 301.8 | 347.1 |
| 163 | 5996355 | 536.5 | 1225.5 | 824.3 | 550.2 | 292.3 | 796.0 | 504.9 |
| 164 | 5996882 | 416.3 | 982.0 | 826.9 | 513.5 | 184.6 | 677.8 | 450.1 |
| 165 | 5998097 | 509.8 | 1009.1 | 746.2 | 555.3 | 300.6 | 698.2 | 520.2 |
| 166 | 5999094 | 567.7 | 1448.3 | 910.4 | 678.5 | 249.7 | 1114.7 | 518.7 |
| 167 | 6082366 | 10477.0 | 23.2 | 1291.5 | 4067.5 | 120.7 | 1507.1 | 6109.5 |
| 168 | 6093032 | 3285.2 | 16.5 | 205.1 | 333.9 | 83.0 | 269.2 | 2756.2 |
| 169 | 6199144 | 472.3 | 607.7 | 468.8 | 364.8 | 355.0 | 517.5 | 432.8 |

^a^Site Location refers to the genomic position of the base in the center of a given ChIP peak.
