# Supplementary material for: Promoter selectivity of the RhlR quorum-sensing transcription factor receptor in Pseudomonas aeruginosa is coordinated by distinct and overlapping dependencies on C4-homoserine lactone and PqsE
Source: PLoS Genet. 2023 Dec 8;19(12):e1010900. doi: 10.1371/journal.pgen.1010900 (PMC10732425; doi:10.1371/journal.pgen.1010900)
Supplement: S2 Table — Expression values were determined using Rockhopper [69] with data from Simanek et al. 2022 [29]. Q-values represent the probability of a false discovery of differentially expressed genes. (DOCX) [file pgen.1010900.s005.docx]

|  | A | B | C | D | E | F | G | H | I | J | K | L | M | N |
| --- | --- | --- | --- | --- | --- | --- | --- | --- | --- | --- | --- | --- | --- | --- |
| 1 | **Associated Binding Site^a^** | **Gene Start^b^** | **Gene End^c^** | **Strand^d^** | **Name** | **Synonym** | **Product** | **Expression WT** | **Expression Δ*rhlR*** | **Expression Δ*rhlI*** | **Expression Δ*pqsE*** | **Expression *pqsE-* NI** | **Expression *pqsE* (D73A)** | **qValue WT vs Δ*rhlR*** |
| 2 | - | 483 | 2027 | + | *dnaA* | *PA14_00010* | chromosomal replication initiation protein | 257 | 240 | 252 | 245 | 233 | 229 | 1 |
| 3 | - | 2056 | 3159 | + | *dnaN* | *PA14_00020* | DNA polymerase III subunit beta | 194 | 256 | 197 | 192 | 202 | 190 | 1 |
| 4 | - | 3169 | 4278 | + | *recF* | *PA14_00030* | recombination protein F | 251 | 223 | 304 | 214 | 235 | 217 | 1 |
| 5 | - | 4275 | 6695 | + | *gyrB* | *PA14_00050* | DNA gyrase subunit B | 244 | 282 | 219 | 216 | 257 | 222 | 1 |
| 6 | - | 7791 | 7018 | - | *-* | *PA14_00060* | acyltransferase | 39 | 36 | 39 | 32 | 33 | 35 | 1 |
| 7 | - | 8339 | 7803 | - | *-* | *PA14_00070* | D,D-heptose 1,7-bisphosphate phosphatase | 41 | 32 | 52 | 33 | 35 | 35 | 1 |
| 8 | - | 8671 | 10377 | + | *-* | *PA14_00080* | hypothetical protein | 110 | 85 | 117 | 119 | 97 | 110 | 1 |
| 9 | - | 12488 | 10434 | - | *glyS* | *PA14_00090* | glycyl-tRNA synthetase subunit beta | 67 | 95 | 68 | 68 | 77 | 50 | 1 |
| 10 | - | 13435 | 12488 | - | *glyQ* | *PA14_00100* | glycyl-tRNA synthetase subunit alpha | 45 | 60 | 42 | 47 | 51 | 33 | 1 |
| 11 | - | 13540 | 14091 | + | *tag* | *PA14_00110* | DNA-3-methyladenine glycosidase I | 16 | 16 | 16 | 14 | 16 | 13 | 1 |
| 12 | - | 14236 | 15123 | + | *-* | *PA14_00120* | lipid A biosynthesis lauroyl acyltransferase | 51 | 50 | 62 | 55 | 63 | 52 | 1 |
| 13 | - | 15208 | 15474 | + | *-* | *PA14_00130* | hypothetical protein | 44 | 47 | 59 | 45 | 47 | 44 | 1 |
| 14 | - | 15621 | 16274 | + | *-* | *PA14_00140* | hypothetical protein | 11 | 14 | 13 | 10 | 9 | 9 | 1 |
| 15 | - | 16608 | 16336 | - | *-* | *PA14_00150* | hypothetical protein | 6 | 9 | 8 | 5 | 6 | 4 | 1 |
| 16 | - | 17240 | 16923 | - | *-* | *PA14_00160* | hypothetical protein | 130 | 123 | 141 | 124 | 132 | 139 | 1 |
| 17 | - | 18762 | 17389 | - | *trkA* | *PA14_00170* | potassium transporter peripheral membrane protein | 87 | 85 | 81 | 73 | 83 | 70 | 1 |
| 18 | - | 20093 | 18789 | - | *-* | *PA14_00180* | tRNA and rRNA cytosine-C5-methylases | 67 | 68 | 68 | 54 | 62 | 53 | 1 |
| 19 | - | 21034 | 20090 | - | *fmt* | *PA14_00190* | methionyl-tRNA formyltransferase | 70 | 58 | 66 | 50 | 63 | 52 | 1 |
| 20 | - | 21594 | 21088 | - | *def* | *PA14_00200* | peptide deformylase | 342 | 301 | 343 | 220 | 259 | 288 | 1 |
| 21 | - | 21733 | 22758 | + | *-* | *PA14_00210* | lysin domain-containing protein | 71 | 95 | 76 | 94 | 85 | 66 | 1 |
| 22 | - | 22893 | 23981 | + | *-* | *PA14_00230* | Rossmann fold nucleotide-binding protein | 9 | 13 | 9 | 12 | 10 | 9 | 1 |
| 23 | - | 24022 | 24579 | + | *-* | *PA14_00240* | hypothetical protein | 71 | 81 | 74 | 78 | 69 | 70 | 1 |
| 24 | - | 25566 | 24589 | - | *qor* | *PA14_00250* | quinone oxidoreductase | 73 | 85 | 67 | 77 | 73 | 64 | 1 |
| 25 | - | 25757 | 26674 | + | *hemF* | *PA14_00280* | coproporphyrinogen III oxidase | 81 | 95 | 74 | 111 | 192 | 149 | 1 |
| 26 | - | 26732 | 27556 | + | *aroE* | *PA14_00290* | shikimate 5-dehydrogenase | 74 | 98 | 81 | 106 | 146 | 121 | 1 |
| 27 | - | 27667 | 28653 | + | *plcB* | *PA14_00300* | phospholipase C, PlcB | 80 | 132 | 110 | 139 | 115 | 100 | 1 |
| 28 | - | 28634 | 29920 | + | *-* | *PA14_00310* | peptidyl-prolyl isomerase | 194 | 304 | 286 | 370 | 262 | 238 | 1 |
| 29 | - | 29917 | 30519 | + | *-* | *PA14_00320* | hypothetical protein | 112 | 201 | 148 | 221 | 157 | 149 | 0.87182855 |
| 30 | - | 32076 | 30523 | - | *-* | *PA14_00340* | sulfate transporter | 36 | 61 | 49 | 82 | 57 | 57 | 0.853730745 |
| 31 | - | 33004 | 32081 | - | *-* | *PA14_00360* | hypothetical protein | 8 | 7 | 7 | 9 | 7 | 7 | 1 |
| 32 | - | 34532 | 33021 | - | *betC* | *PA14_00380* | choline sulfatase | 21 | 19 | 23 | 22 | 17 | 19 | 1 |
| 33 | - | 34645 | 35559 | + | *-* | *PA14_00400* | LysR family transcriptional regulator | 18 | 19 | 18 | 16 | 16 | 15 | 1 |
| 34 | - | 35570 | 35947 | + | *-* | *PA14_00410* | dioxygenase | 131 | 118 | 109 | 97 | 112 | 103 | 1 |
| 35 | - | 36291 | 35926 | - | *-* | *PA14_00420* | histidine phosphotransfer domain-containing protein | 51 | 51 | 51 | 41 | 45 | 39 | 1 |
| 36 | - | 36922 | 36299 | - | *-* | *PA14_00430* | two-component response regulator | 31 | 23 | 27 | 24 | 24 | 23 | 1 |
| 37 | - | 37914 | 37108 | - | *trpA* | *PA14_00440* | tryptophan synthase subunit alpha | 55 | 49 | 42 | 43 | 45 | 43 | 1 |
| 38 | - | 39119 | 37911 | - | *trpB* | *PA14_00450* | tryptophan synthase subunit beta | 80 | 64 | 63 | 63 | 64 | 63 | 1 |
| 39 | - | 39223 | 40110 | + | *trpI* | *PA14_00460* | transcriptional regulator TrpI | 19 | 17 | 16 | 15 | 14 | 14 | 1 |
| 40 | - | 40211 | 40426 | + | *-* | *PA14_00470* | hypothetical protein | 773 | 957 | 1090 | 933 | 1030 | 1009 | 1 |
| 41 | - | 40453 | 40609 | ? | *-* | predicted RNA | - | 1520 | 2020 | 1667 | 1909 | 1651 | 1921 | 1 |
| 42 | - | 40610 | 40837 | + | *-* | *PA14_00480* | hypothetical protein | 2080 | 2747 | 2724 | 3329 | 2669 | 3108 | 1 |
| 43 | - | 41133 | 42821 | + | *-* | *PA14_00490* | hemolysin activation/secretion protein | 26 | 25 | 28 | 26 | 25 | 21 | 1 |
| 44 | - | 42934 | 53265 | + | *-* | *PA14_00510* | hemagglutinin | 36 | 29 | 31 | 25 | 30 | 24 | 1 |
| 45 | - | 54577 | 54699 | + | *-* | *PA14_00520* | hypothetical protein | 32 | 27 | 22 | 31 | 43 | 39 | 1 |
| 46 | - | 54909 | 55304 | + | *-* | *PA14_00530* | hypothetical protein | 86 | 77 | 83 | 107 | 70 | 76 | 1 |
| 47 | - | 56984 | 55575 | - | *-* | *PA14_00550* | hypothetical protein | 18 | 16 | 17 | 14 | 17 | 14 | 1 |
| 48 | - | 57148 | 58476 | + | *exoT* | *PA14_00560* | exoenzyme T | 176 | 174 | 208 | 194 | 168 | 183 | 1 |
| 49 | - | 59018 | 59704 | + | *-* | *PA14_00570* | lipoprotein | 12 | 17 | 13 | 10 | 14 | 8 | 1 |
| 50 | - | 59735 | 60088 | + | *-* | *PA14_00580* | lipoprotein | 16 | 24 | 17 | 14 | 26 | 12 | 1 |
| 51 | - | 60241 | 60750 | + | *-* | *PA14_00590* | lipoprotein | 17 | 19 | 16 | 13 | 18 | 11 | 1 |
| 52 | - | 61148 | 60765 | - | *-* | *PA14_00600* | transcriptional regulator | 9 | 15 | 12 | 9 | 14 | 6 | 1 |
| 53 | - | 63091 | 61430 | - | *-* | *PA14_00620* | hypothetical protein | 6 | 12 | 8 | 12 | 11 | 9 | 0.258262081 |
| 54 | - | 63701 | 63841 | + | *-* | *PA14_00630* | hypothetical protein | 131 | 82 | 103 | 136 | 115 | 161 | 1 |
| 55 | 64211 | 64665 | 66497 | + | *phzH* | *PA14_00640* | potential phenazine-modifying enzyme | 63 | 10 | 10 | 20 | 20 | 57 | 3.40517E-17 |
| 56 | - | 66978 | 66550 | - | *-* | *PA14_00650* | hypothetical protein | 105 | 83 | 96 | 135 | 110 | 209 | 1 |
| 57 | - | 67735 | 67187 | - | *-* | *PA14_00660* | RNA 2'-phosphotransferase-like protein | 17 | 16 | 12 | 12 | 20 | 12 | 1 |
| 58 | - | 68280 | 67774 | - | *-* | *PA14_00670* | hypothetical protein | 45 | 52 | 34 | 38 | 40 | 32 | 1 |
| 59 | - | 69266 | 68346 | - | *-* | *PA14_00680* | LysR family transcriptional regulator | 10 | 10 | 9 | 10 | 10 | 9 | 1 |
| 60 | - | 69374 | 70261 | + | *-* | *PA14_00690* | hypothetical protein | 10 | 6 | 8 | 8 | 7 | 9 | 0.41668339 |
| 61 | - | 70324 | 71028 | + | *-* | *PA14_00700* | hypothetical protein | 7 | 7 | 7 | 8 | 6 | 8 | 1 |
| 62 | - | 71112 | 71567 | + | *osmC* | *PA14_00710* | osmotically inducible protein OsmC | 131 | 178 | 140 | 165 | 194 | 180 | 1 |
| 63 | - | 71678 | 71911 | + | *-* | *PA14_00720* | hypothetical protein | 93 | 118 | 105 | 107 | 148 | 145 | 1 |
| 64 | - | 72360 | 71923 | - | *-* | *PA14_00730* | hypothetical protein | 141 | 115 | 151 | 125 | 134 | 126 | 1 |
| 65 | - | 72833 | 72417 | - | *-* | *PA14_00740* | lipoprotein | 24 | 28 | 31 | 32 | 29 | 24 | 1 |
| 66 | - | 72925 | 74052 | + | *-* | *PA14_00750* | hypothetical protein | 75 | 69 | 68 | 68 | 64 | 70 | 1 |
| 67 | - | 75043 | 74060 | - | *-* | *PA14_00760* | hypothetical protein | 82 | 70 | 65 | 73 | 65 | 63 | 1 |
| 68 | - | 75741 | 75076 | - | *-* | *PA14_00770* | hypothetical protein | 48 | 50 | 48 | 49 | 51 | 46 | 1 |
| 69 | - | 76276 | 75734 | - | *-* | *PA14_00780* | hypothetical protein | 82 | 81 | 78 | 67 | 71 | 71 | 1 |
| 70 | - | 76354 | 78399 | + | *prlC* | *PA14_00790* | oligopeptidase A | 216 | 174 | 156 | 142 | 166 | 186 | 1 |
| 71 | - | 78396 | 78671 | + | *-* | *PA14_00800* | hypothetical protein | 117 | 123 | 81 | 92 | 107 | 102 | 1 |
| 72 | - | 78760 | 79818 | + | *-* | *PA14_00810* | DNA repair photolyase | 18 | 17 | 17 | 18 | 19 | 22 | 1 |
| 73 | - | 80963 | 80049 | - | *-* | *PA14_00820* | hypothetical protein | 130 | 136 | 79 | 46 | 122 | 73 | 1 |
| 74 | - | 82737 | 81025 | - | *-* | *PA14_00830* | hypothetical protein | 63 | 51 | 46 | 41 | 54 | 48 | 1 |
| 75 | - | 83929 | 82730 | - | *-* | *PA14_00850* | hypothetical protein | 32 | 20 | 21 | 14 | 24 | 18 | 1 |
| 76 | - | 84648 | 83929 | - | *-* | *PA14_00860* | ABC transporter ATP-binding protein | 40 | 24 | 21 | 16 | 28 | 24 | 0.925024982 |
| 77 | - | 87743 | 84645 | - | *ppkA* | *PA14_00875* | serine/threonine protein kinase PpkA | 58 | 40 | 36 | 33 | 54 | 45 | 1 |
| 78 | - | 88479 | 87751 | - | *-* | *PA14_00890* | phosphoprotein phosphatase | 59 | 38 | 44 | 27 | 54 | 45 | 1 |
| 79 | - | 89169 | 88489 | - | *-* | *PA14_00900* | hypothetical protein | 59 | 40 | 40 | 28 | 50 | 46 | 1 |
| 80 | - | 92474 | 89166 | - | *-* | *PA14_00910* | hypothetical protein | 62 | 40 | 40 | 36 | 48 | 45 | 1 |
| 81 | - | 94045 | 92696 | - | *-* | *PA14_00925* | hypothetical protein | 81 | 54 | 62 | 54 | 60 | 54 | 1 |
| 82 | - | 95386 | 94052 | - | *-* | *PA14_00940* | hypothetical protein | 60 | 47 | 54 | 46 | 51 | 45 | 1 |
| 83 | - | 95866 | 95402 | - | *-* | *PA14_00960* | lipoprotein | 37 | 32 | 27 | 24 | 30 | 24 | 1 |
| 84 | - | 96453 | 95911 | - | *-* | *PA14_00970* | hypothetical protein | 95 | 90 | 78 | 74 | 70 | 81 | 1 |

|  | A | B | C | D | E | F | G | H | I | J | K | L | M | N |
| --- | --- | --- | --- | --- | --- | --- | --- | --- | --- | --- | --- | --- | --- | --- |
| 85 | - | 97409 | 96450 | - | *-* | *PA14_00980* | hypothetical protein | 79 | 71 | 68 | 82 | 81 | 80 | 1 |
| 86 | - | 97777 | 98811 | + | *-* | *PA14_00990* | hypothetical protein | 42 | 25 | 33 | 22 | 24 | 19 | 1 |
| 87 | - | 98900 | 99418 | + | *-* | *PA14_01010* | hypothetical protein | 186 | 137 | 126 | 117 | 195 | 107 | 1 |
| 88 | - | 99431 | 100927 | + | *-* | *PA14_01020* | hypothetical protein | 103 | 85 | 70 | 55 | 94 | 57 | 1 |
| 89 | - | 101003 | 101491 | + | *-* | *PA14_01030* | hypothetical protein | 324 | 239 | 180 | 116 | 280 | 158 | 1 |
| 90 | - | 101659 | 102504 | + | *-* | *PA14_01040* | secretion protein | 64 | 38 | 44 | 28 | 47 | 34 | 1 |
| 91 | - | 102506 | 103015 | + | *-* | *PA14_01060* | hypothetical protein | 24 | 18 | 19 | 16 | 23 | 17 | 1 |
| 92 | - | 103012 | 104871 | + | *-* | *PA14_01070* | hypothetical protein | 24 | 19 | 18 | 13 | 22 | 15 | 1 |
| 93 | - | 104835 | 105881 | + | *-* | *PA14_01080* | hypothetical protein | 27 | 16 | 17 | 12 | 27 | 14 | 0.803084159 |
| 94 | - | 105874 | 108582 | + | *-* | *PA14_01100* | ClpA/B-type chaperone | 59 | 37 | 37 | 29 | 57 | 35 | 1 |
| 95 | - | 108629 | 110560 | + | *-* | *PA14_01110* | hypothetical protein | 43 | 27 | 24 | 19 | 37 | 23 | 1 |
| 96 | - | 110959 | 110675 | - | *-* | *PA14_01120* | hypothetical protein | 50 | 53 | 37 | 36 | 57 | 35 | 1 |
| 97 | - | 111205 | 110972 | - | *-* | *PA14_01130* | hypothetical protein | 72 | 69 | 65 | 61 | 95 | 69 | 1 |
| 98 | - | 112514 | 111195 | - | *-* | *PA14_01140* | hypothetical protein | 27 | 23 | 24 | 19 | 23 | 16 | 1 |
| 99 | - | 113027 | 112593 | - | *-* | *PA14_01150* | hypothetical protein | 28 | 27 | 24 | 28 | 27 | 24 | 1 |
| 100 | - | 113281 | 115506 | + | *-* | *PA14_01160* | hypothetical protein | 83 | 70 | 87 | 89 | 79 | 75 | 1 |
| 101 | - | 115534 | 115983 | + | *-* | *PA14_01170* | hypothetical protein | 44 | 34 | 33 | 31 | 34 | 34 | 1 |
| 102 | - | 115913 | 117112 | + | *-* | *PA14_01180* | hypothetical protein | 24 | 20 | 22 | 20 | 25 | 19 | 1 |
| 103 | - | 117109 | 118146 | + | *-* | *PA14_01190* | 3-oxoacyl-ACP synthase | 28 | 23 | 24 | 20 | 26 | 19 | 1 |
| 104 | - | 118146 | 119237 | + | *-* | *PA14_01200* | hypothetical protein | 43 | 33 | 38 | 33 | 41 | 34 | 1 |
| 105 | - | 119248 | 120168 | + | *-* | *PA14_01220* | hypothetical protein | 81 | 57 | 63 | 53 | 70 | 61 | 1 |
| 106 | - | 120178 | 121410 | + | *-* | *PA14_01230* | hypothetical protein | 37 | 27 | 27 | 23 | 27 | 24 | 1 |
| 107 | - | 121786 | 122514 | + | *-* | *PA14_01240* | carbonic anhydrase | 103 | 107 | 86 | 80 | 98 | 88 | 1 |
| 108 | - | 122725 | 124296 | + | *-* | *PA14_01250* | sulfate transporter | 17 | 16 | 19 | 21 | 18 | 16 | 1 |
| 109 | - | 124433 | 125029 | + | *-* | *PA14_01270* | hypothetical protein | 13 | 15 | 19 | 15 | 14 | 12 | 1 |
| 110 | - | 125293 | 126417 | + | *coxB* | *PA14_01290* | cytochrome c oxidase subunit II | 133 | 95 | 185 | 117 | 68 | 106 | 1 |
| 111 | - | 126427 | 128019 | + | *coxA* | *PA14_01300* | cytochrome c oxidase subunit I | 73 | 49 | 68 | 71 | 42 | 75 | 1 |
| 112 | - | 128030 | 128584 | + | *-* | *PA14_01310* | cytochrome C oxidase assembly protein | 45 | 37 | 58 | 68 | 36 | 68 | 1 |
| 113 | - | 128595 | 129482 | + | *coIII* | *PA14_01320* | cytochrome c oxidase subunit III | 161 | 166 | 209 | 207 | 164 | 238 | 1 |
| 114 | - | 129707 | 129498 | - | *-* | *PA14_01330* | hypothetical protein | 432 | 489 | 604 | 646 | 666 | 715 | 1 |
| 115 | - | 129723 | 130517 | + | *-* | *PA14_01340* | hypothetical protein | 31 | 24 | 40 | 33 | 23 | 32 | 1 |
| 116 | - | 130492 | 131070 | + | *-* | *PA14_01350* | hypothetical protein | 18 | 18 | 24 | 25 | 14 | 26 | 1 |
| 117 | - | 131135 | 132208 | + | *-* | *PA14_01360* | hypothetical protein | 18 | 15 | 16 | 21 | 12 | 21 | 1 |
| 118 | - | 132234 | 133148 | + | *-* | *PA14_01380* | protoheme IX farnesyltransferase | 40 | 39 | 44 | 48 | 40 | 54 | 1 |
| 119 | - | 133174 | 133809 | + | *-* | *PA14_01390* | hypothetical protein | 106 | 73 | 101 | 87 | 104 | 105 | 1 |
| 120 | - | 134301 | 133849 | - | *-* | *PA14_01400* | hypothetical protein | 33 | 37 | 35 | 30 | 36 | 28 | 1 |
| 121 | - | 134433 | 134906 | + | *-* | *PA14_01410* | hypothetical protein | 75 | 88 | 82 | 91 | 77 | 89 | 1 |
| 122 | - | 135162 | 135890 | + | *-* | *PA14_01430* | short chain dehydrogenase | 13 | 10 | 10 | 11 | 10 | 12 | 1 |
| 123 | - | 135915 | 136502 | + | *-* | *PA14_01440* | hypothetical protein | 15 | 17 | 11 | 12 | 13 | 14 | 1 |
| 124 | - | 136732 | 138081 | + | *-* | *PA14_01460* | C4-dicarboxylate transporter DctA | 10 | 14 | 9 | 10 | 13 | 10 | 1 |
| 125 | - | 138130 | 138816 | + | *-* | *PA14_01470* | transcriptional regulator | 68 | 58 | 50 | 58 | 60 | 60 | 1 |
| 126 | - | 138917 | 139651 | + | *-* | *PA14_01480* | hypothetical protein | 22 | 25 | 18 | 20 | 18 | 19 | 1 |
| 127 | 139733 | 139831 | 140241 | + | *-* | *PA14_01490* | hemolysin | 2956 | 160 | 1024 | 2108 | 1401 | 3762 | 2.0605E-123 |
| 128 | - | 141181 | 140273 | - | *-* | *PA14_01500* | transcriptional regulator | 189 | 30 | 58 | 127 | 84 | 196 | 2.46944E-13 |
| 129 | - | 141762 | 141481 | - | *-* | *PA14_01510* | hypothetical protein | 11 | 8 | 8 | 7 | 7 | 7 | 1 |
| 130 | - | 141986 | 141759 | - | *-* | *PA14_01520* | hypothetical protein | 10 | 8 | 9 | 9 | 9 | 10 | 1 |
| 131 | - | 142785 | 142162 | - | *-* | *PA14_01540* | hypothetical protein | 16 | 17 | 13 | 8 | 14 | 9 | 1 |
| 132 | - | 142886 | 143386 | + | *-* | *PA14_01550* | lipoprotein | 25 | 31 | 27 | 24 | 27 | 19 | 1 |
| 133 | - | 143458 | 143799 | + | *-* | *PA14_01560* | hypothetical protein | 98 | 73 | 154 | 58 | 48 | 59 | 1 |
| 134 | - | 145308 | 143881 | - | *gabP* | *PA14_01580* | gamma-aminobutyrate permease | 39 | 55 | 47 | 60 | 55 | 66 | 1 |
| 135 | - | 146970 | 145477 | - | *-* | *PA14_01600* | aldehyde dehydrogenase | 263 | 323 | 268 | 377 | 335 | 401 | 1 |
| 136 | - | 147341 | 147054 | - | *-* | *PA14_01610* | hypothetical protein | 96 | 171 | 173 | 290 | 220 | 343 | 0.605197743 |
| 137 | - | 148687 | 147341 | - | *aptA* | *PA14_01620* | beta alanine--pyruvate transaminase | 161 | 266 | 260 | 396 | 349 | 569 | 1 |
| 138 | - | 148823 | 149740 | + | *-* | *PA14_01640* | LysR family transcriptional regulator | 17 | 20 | 19 | 22 | 18 | 23 | 1 |
| 139 | - | 151223 | 149853 | - | *-* | *PA14_01660* | guanine deaminase | 52 | 45 | 45 | 48 | 50 | 51 | 1 |
| 140 | - | 152381 | 153952 | + | *-* | *PA14_01670* | ABC transporter ATP-binding protein | 12 | 10 | 13 | 13 | 11 | 12 | 1 |
| 141 | - | 153952 | 155049 | + | *-* | *PA14_01680* | ABC transporter permease | 4 | 4 | 3 | 4 | 5 | 4 | 1 |
| 142 | - | 155072 | 156052 | + | *-* | *PA14_01690* | ABC transporter permease | 16 | 18 | 18 | 15 | 16 | 15 | 1 |
| 143 | - | 156164 | 156727 | + | *ahpC* | *PA14_01710* | alkyl hydroperoxide reductase | 1247 | 649 | 297 | 276 | 440 | 1483 | 1 |
| 144 | - | 156872 | 158437 | + | *ahpF* | *PA14_01720* | alkyl hydroperoxide reductase | 288 | 98 | 98 | 59 | 74 | 280 | 0.579361359 |
| 145 | - | 159413 | 158517 | - | *-* | *PA14_01730* | hypothetical protein | 562 | 683 | 744 | 382 | 542 | 498 | 1 |
| 146 | - | 159868 | 161217 | + | *-* | *PA14_01750* | hydroxydechloroatrazine ethylaminohydrolase | 19 | 31 | 24 | 29 | 30 | 22 | 0.952168061 |
| 147 | - | 161387 | 162376 | + | *nuh* | *PA14_01760* | nonspecific ribonucleoside hydrolase | 79 | 97 | 98 | 103 | 90 | 88 | 1 |
| 148 | - | 163261 | 162413 | - | *-* | *PA14_01770* | nucleoside-binding outer membrane protein | 35 | 56 | 47 | 59 | 47 | 41 | 0.944453713 |
| 149 | - | 163922 | 163296 | - | *-* | *PA14_01780* | nucleoside 2-deoxyribosyltransferase | 85 | 136 | 133 | 158 | 116 | 117 | 0.940967938 |
| 150 | - | 164590 | 164072 | - | *-* | *PA14_01790* | nucleoside 2-deoxyribosyltransferase | 23 | 26 | 28 | 23 | 23 | 22 | 1 |
| 151 | - | 164828 | 165925 | + | *-* | *PA14_01800* | hypothetical protein | 5 | 7 | 6 | 7 | 4 | 5 | 1 |
| 152 | - | 165998 | 166960 | + | *-* | *PA14_01810* | oxidoreductase | 23 | 21 | 22 | 22 | 21 | 23 | 1 |
| 153 | - | 167065 | 168015 | + | *-* | *PA14_01830* | adenosine deaminase | 33 | 33 | 36 | 34 | 26 | 29 | 1 |
| 154 | - | 168212 | 168757 | + | *-* | *PA14_01840* | RNA polymerase ECF-subfamily sigma-70 factor | 17 | 22 | 21 | 44 | 29 | 18 | 1 |
| 155 | - | 168754 | 169749 | + | *-* | *PA14_01860* | transmembrane sensor | 11 | 15 | 12 | 18 | 15 | 8 | 1 |
| 156 | - | 169897 | 172284 | + | *-* | *PA14_01870* | TonB-dependent receptor | 7 | 6 | 7 | 9 | 7 | 5 | 1 |
| 157 | - | 172662 | 173489 | + | *pcaQ* | *PA14_01890* | transcriptional regulator PcaQ | 39 | 38 | 44 | 32 | 37 | 38 | 1 |
| 158 | - | 173624 | 174343 | + | *pcaH* | *PA14_01900* | protocatechuate 3,4-dioxygenase subunit beta | 10 | 11 | 11 | 11 | 9 | 10 | 1 |
| 159 | - | 174354 | 174959 | + | *pcaG* | *PA14_01910* | protocatechuate 3,4-dioxygenase subunit alpha | 11 | 13 | 14 | 12 | 12 | 14 | 1 |
| 160 | - | 175175 | 176014 | + | *pcaR* | *PA14_01930* | transcriptional regulator PcaR | 14 | 14 | 14 | 15 | 13 | 16 | 1 |
| 161 | - | 176167 | 177318 | + | *-* | *PA14_01940* | RND efflux membrane fusion protein | 97 | 113 | 124 | 155 | 113 | 106 | 1 |
| 162 | - | 177315 | 178385 | + | *-* | *PA14_01960* | RND efflux membrane fusion protein | 84 | 124 | 114 | 145 | 115 | 107 | 1 |
| 163 | - | 178382 | 181429 | + | *-* | *PA14_01970* | RND efflux transporter | 120 | 178 | 173 | 207 | 180 | 173 | 1 |
| 164 | - | 181628 | 182566 | + | *-* | *PA14_01980* | LysR family transcriptional regulator | 45 | 52 | 51 | 49 | 53 | 51 | 1 |
| 165 | - | 182682 | 182867 | + | *-* | *PA14_01990* | hypothetical protein | 31 | 33 | 29 | 29 | 26 | 28 | 1 |
| 166 | - | 183147 | 183299 | + | *-* | *PA14_02010* | hypothetical protein | 14 | 6 | 7 | 6 | 8 | 5 | 0.25264025 |
| 167 | - | 183454 | 184788 | + | *-* | *PA14_02020* | outer membrane porin | 32 | 34 | 28 | 26 | 30 | 25 | 1 |
| 168 | - | 185614 | 184817 | - | *-* | *PA14_02030* | AraC family transcriptional regulator | 19 | 19 | 14 | 17 | 21 | 17 | 1 |

|  | A | B | C | D | E | F | G | H | I | J | K | L | M | N |
| --- | --- | --- | --- | --- | --- | --- | --- | --- | --- | --- | --- | --- | --- | --- |
| 169 | - | 185692 | 187308 | + | *-* | *PA14_02050* | gamma-glutamyltranspeptidase | 30 | 21 | 31 | 18 | 22 | 24 | 1 |
| 170 | - | 187980 | 188816 | + | *-* | *PA14_02060* | hypothetical protein | 5 | 7 | 6 | 5 | 5 | 5 | 1 |
| 171 | - | 189058 | 190464 | + | *-* | *PA14_02070* | transporter | 10 | 13 | 10 | 10 | 9 | 9 | 1 |
| 172 | - | 190557 | 191222 | + | *-* | *PA14_02090* | TetR family transcriptional regulator | 39 | 49 | 39 | 29 | 36 | 26 | 1 |
| 173 | - | 191228 | 191818 | + | *-* | *PA14_02100* | hypothetical protein | 116 | 130 | 100 | 105 | 127 | 105 | 1 |
| 174 | - | 192532 | 191825 | - | *-* | *PA14_02110* | diguanylate cyclase | 43 | 40 | 43 | 44 | 46 | 44 | 1 |
| 175 | - | 193039 | 192659 | - | *-* | *PA14_02130* | hypothetical protein | 10 | 9 | 10 | 8 | 8 | 8 | 1 |
| 176 | - | 193608 | 193066 | - | *-* | *PA14_02140* | hypothetical protein | 6 | 6 | 6 | 7 | 5 | 3 | 1 |
| 177 | - | 195608 | 193617 | - | *-* | *PA14_02150* | hypothetical protein | 11 | 9 | 11 | 11 | 8 | 9 | 1 |
| 178 | - | 196920 | 195871 | - | *cheB* | *PA14_02180* | chemotaxis-specific methylesterase | 29 | 28 | 36 | 40 | 31 | 48 | 1 |
| 179 | - | 197542 | 196940 | - | *-* | *PA14_02190* | hypothetical protein | 47 | 41 | 55 | 52 | 56 | 70 | 1 |
| 180 | - | 198390 | 197548 | - | *-* | *PA14_02200* | chemotaxis protein methyltransferase | 83 | 88 | 108 | 104 | 102 | 146 | 1 |
| 181 | - | 200499 | 198460 | - | *-* | *PA14_02220* | chemotaxis transducer | 406 | 421 | 485 | 568 | 490 | 659 | 1 |
| 182 | - | 201020 | 200535 | - | *cheW* | *PA14_02230* | purine-binding chemotaxis protein | 316 | 318 | 387 | 473 | 376 | 504 | 1 |
| 183 | - | 202926 | 201007 | - | *cheA* | *PA14_02250* | two-component sensor | 252 | 225 | 295 | 300 | 261 | 324 | 1 |
| 184 | - | 203319 | 202954 | - | *-* | *PA14_02260* | two-component response regulator | 753 | 756 | 1089 | 880 | 863 | 997 | 1 |
| 185 | - | 204689 | 203517 | - | *-* | *PA14_02270* | chemotaxis transducer | 106 | 96 | 133 | 137 | 101 | 121 | 1 |
| 186 | - | 205815 | 204880 | - | *-* | *PA14_02290* | LysR family transcriptional regulator | 42 | 38 | 36 | 44 | 40 | 38 | 1 |
| 187 | - | 205932 | 206684 | + | *fabG* | *PA14_02300* | 3-ketoacyl-ACP reductase | 16 | 16 | 13 | 15 | 17 | 11 | 1 |
| 188 | - | 208394 | 206784 | - | *atsA* | *PA14_02310* | arylsulfatase | 7 | 8 | 6 | 6 | 7 | 6 | 1 |
| 189 | - | 209321 | 208482 | - | *-* | *PA14_02330* | ABC transporter ATP-binding protein | 8 | 6 | 6 | 6 | 6 | 6 | 1 |
| 190 | - | 210934 | 209318 | - | *-* | *PA14_02340* | ABC transporter permease | 6 | 6 | 4 | 6 | 5 | 5 | 1 |
| 191 | - | 211201 | 212262 | + | *-* | *PA14_02360* | ABC transporter substrate-binding protein | 10 | 10 | 10 | 10 | 11 | 12 | 1 |
| 192 | - | 213643 | 212285 | - | *-* | *PA14_02370* | porin | 14 | 15 | 15 | 14 | 13 | 14 | 1 |
| 193 | - | 213891 | 214616 | + | *-* | *PA14_02380* | acid phosphatase | 8 | 9 | 9 | 12 | 8 | 8 | 1 |
| 194 | - | 215557 | 214640 | - | *-* | *PA14_02390* | transcriptional regulator | 8 | 14 | 11 | 12 | 10 | 10 | 0.956796655 |
| 195 | - | 215908 | 218280 | + | *-* | *PA14_02410* | TonB-dependent receptor | 7 | 9 | 8 | 10 | 7 | 8 | 1 |
| 196 | - | 218321 | 219223 | + | *-* | *PA14_02420* | hypothetical protein | 4 | 3 | 4 | 3 | 4 | 3 | 1 |
| 197 | - | 219291 | 220190 | + | *-* | *PA14_02435* | hypothetical protein | 5 | 5 | 6 | 6 | 5 | 5 | 1 |
| 198 | - | 220837 | 221955 | + | *-* | *PA14_02450* | NAD(P) transhydrogenase subunit alpha part 1 | 56 | 59 | 71 | 67 | 62 | 73 | 1 |
| 199 | - | 222031 | 222339 | + | *-* | *PA14_02460* | NAD(P) transhydrogenase subunit alpha part 2 | 27 | 28 | 33 | 37 | 34 | 44 | 1 |
| 200 | - | 222339 | 223775 | + | *pntB* | *PA14_02470* | pyridine nucleotide transhydrogenase subunit beta | 30 | 36 | 34 | 36 | 33 | 45 | 1 |
| 201 | - | 224119 | 224931 | + | *tonB2* | *PA14_02490* | hypothetical protein | 11 | 9 | 9 | 5 | 10 | 14 | 1 |
| 202 | - | 224960 | 225679 | + | *exbB1* | *PA14_02500* | transport protein ExbB | 17 | 16 | 18 | 17 | 19 | 29 | 1 |
| 203 | - | 225681 | 226082 | + | *exbD1* | *PA14_02510* | transport protein ExbD | 76 | 54 | 45 | 23 | 63 | 133 | 1 |
| 204 | - | 226475 | 226263 | - | *-* | *PA14_02520* | hypothetical protein | 215 | 189 | 144 | 75 | 268 | 504 | 1 |
| 205 | - | 226691 | 227272 | + | *-* | *PA14_02530* | hypothetical protein | 34 | 68 | 45 | 27 | 45 | 55 | 0.317658547 |
| 206 | - | 227383 | 229047 | + | *mdcA* | *PA14_02550* | malonate decarboxylase subunit alpha | 78 | 116 | 90 | 68 | 92 | 96 | 1 |
| 207 | - | 229047 | 229928 | + | *-* | *PA14_02560* | triphosphoribosyl-dephospho-CoA synthase | 37 | 70 | 60 | 43 | 62 | 80 | 0.559494168 |
| 208 | - | 229930 | 230229 | + | *mdcC* | *PA14_02570* | malonate decarboxylase subunit delta | 23 | 57 | 45 | 30 | 56 | 61 | 0.044254247 |
| 209 | - | 230222 | 231085 | + | *mdcD* | *PA14_02580* | malonate decarboxylase subunit beta | 38 | 82 | 66 | 49 | 75 | 84 | 0.320506198 |
| 210 | - | 231082 | 231888 | + | *mdcE* | *PA14_02590* | malonate decarboxylase subunit gamma | 28 | 56 | 47 | 40 | 60 | 64 | 0.343217685 |
| 211 | - | 231904 | 232596 | + | *-* | *PA14_02610* | phosphoribosyl-dephospho-CoA transferase | 31 | 42 | 57 | 31 | 41 | 39 | 1 |
| 212 | - | 232593 | 233525 | + | *-* | *PA14_02620* | epsilon subunit of malonate decarboxylase | 48 | 55 | 63 | 44 | 54 | 51 | 1 |
| 213 | - | 233582 | 233986 | + | *-* | *PA14_02630* | malonate carrier protein | 20 | 25 | 27 | 21 | 23 | 22 | 1 |
| 214 | - | 233992 | 234756 | + | *-* | *PA14_02640* | malonate transporter subunit MadM | 18 | 25 | 24 | 24 | 25 | 25 | 1 |
| 215 | - | 235770 | 234841 | - | *-* | *PA14_02650* | malonate utilization transcriptional regulator | 21 | 20 | 20 | 22 | 21 | 18 | 1 |
| 216 | - | 237019 | 236099 | - | *-* | *PA14_02660* | LysR family transcriptional regulator | 22 | 19 | 21 | 22 | 22 | 23 | 1 |
| 217 | - | 237370 | 238860 | + | *-* | *PA14_02680* | aldehyde dehydrogenase | 4 | 4 | 4 | 6 | 4 | 5 | 1 |
| 218 | - | 238918 | 240351 | + | *-* | *PA14_02690* | amino acid permease | 12 | 11 | 12 | 16 | 10 | 12 | 1 |
| 219 | - | 240382 | 241764 | + | *-* | *PA14_02700* | aminotransferase | 6 | 7 | 5 | 6 | 6 | 8 | 1 |
| 220 | - | 241928 | 242986 | + | *-* | *PA14_02720* | hypothetical protein | 2 | 2 | 2 | 2 | 2 | 1 | 1 |
| 221 | - | 243945 | 243064 | - | *-* | *PA14_02730* | dihydrodipicolinate synthetase | 17 | 10 | 10 | 11 | 8 | 8 | 0.527092266 |
| 222 | - | 244787 | 244005 | - | *-* | *PA14_02740* | class II aldolase/adducin domain-containing protein | 39 | 29 | 34 | 40 | 30 | 34 | 1 |
| 223 | - | 244942 | 245481 | + | *-* | *PA14_02750* | transcriptional regulator | 43 | 41 | 39 | 35 | 41 | 38 | 1 |
| 224 | - | 245632 | 246483 | + | *-* | *PA14_02760* | CoA transferase, subunit A | 5 | 6 | 5 | 4 | 5 | 6 | 1 |
| 225 | - | 246480 | 247262 | + | *-* | *PA14_02770* | CoA transferase subunit B | 8 | 8 | 9 | 8 | 6 | 7 | 1 |
| 226 | - | 247259 | 248464 | + | *pcaF* | *PA14_02790* | beta-ketoadipyl CoA thiolase | 16 | 15 | 12 | 14 | 10 | 12 | 1 |
| 227 | - | 248614 | 249912 | + | *pcaT* | *PA14_02810* | dicarboxylic acid transporter PcaT | 31 | 25 | 28 | 28 | 28 | 27 | 1 |
| 228 | - | 249935 | 251314 | + | *pcaB* | *PA14_02830* | 3-carboxy-cis,cis-muconate cycloisomerase | 64 | 62 | 59 | 59 | 61 | 60 | 1 |
| 229 | - | 251329 | 252120 | + | *pcaD* | *PA14_02840* | beta-ketoadipate enol-lactone hydrolase | 46 | 49 | 41 | 38 | 50 | 40 | 1 |
| 230 | - | 252131 | 252532 | + | *pcaC* | *PA14_02850* | gamma-carboxymuconolactone decarboxylase | 49 | 67 | 47 | 59 | 65 | 72 | 1 |
| 231 | - | 252660 | 253601 | + | *-* | *PA14_02870* | transcriptional regulator | 52 | 44 | 42 | 48 | 44 | 47 | 1 |
| 232 | - | 253792 | 254625 | + | *-* | *PA14_02890* | hypothetical protein | 6 | 6 | 6 | 6 | 6 | 5 | 1 |
| 233 | - | 256110 | 254764 | - | *pcaK* | *PA14_02900* | 4-hydroxybenzoate transporter PcaK | 2 | 2 | 3 | 2 | 2 | 2 | 1 |
| 234 | - | 256416 | 257195 | + | *-* | *PA14_02910* | IclR family transcriptional regulator | 52 | 15 | 26 | 32 | 19 | 42 | 9.68945E-05 |
| 235 | - | 257439 | 258482 | + | *-* | *PA14_02930* | oxidoreductase | 9 | 7 | 8 | 7 | 7 | 7 | 1 |
| 236 | - | 258505 | 259320 | + | *-* | *PA14_02960* | hypothetical protein | 11 | 12 | 13 | 13 | 11 | 12 | 1 |
| 237 | - | 259470 | 260348 | + | *-* | *PA14_02970* | hypothetical protein | 12 | 12 | 15 | 11 | 11 | 9 | 1 |
| 238 | - | 261639 | 260374 | - | *-* | *PA14_02980* | porin | 14 | 13 | 15 | 15 | 13 | 15 | 1 |
| 239 | - | 263030 | 261705 | - | *-* | *PA14_02990* | MFS transporter | 4 | 5 | 5 | 4 | 5 | 5 | 1 |
| 240 | - | 263577 | 265481 | + | *-* | *PA14_03000* | hypothetical protein | 8 | 8 | 8 | 8 | 7 | 8 | 1 |
| 241 | - | 265574 | 266242 | + | *-* | *PA14_03010* | TetR family transcriptional regulator | 35 | 24 | 27 | 23 | 22 | 32 | 1 |
| 242 | - | 267136 | 266282 | - | *-* | *PA14_03020* | shikimate 5-dehydrogenase | 9 | 5 | 5 | 4 | 6 | 6 | 0.43335915 |
| 243 | - | 267579 | 267133 | - | *aroQ2* | *PA14_03030* | 3-dehydroquinate dehydratase | 5 | 5 | 6 | 4 | 4 | 5 | 1 |
| 244 | - | 269201 | 267696 | - | *-* | *PA14_03040* | MFS transporter | 20 | 20 | 19 | 17 | 20 | 14 | 1 |
| 245 | - | 270560 | 269376 | - | *pobA* | *PA14_03050* | 4-hydroxybenzoate 3-monooxygenase | 9 | 6 | 6 | 6 | 6 | 7 | 1 |
| 246 | - | 270738 | 271604 | + | *-* | *PA14_03070* | transcriptional regulator | 28 | 39 | 30 | 25 | 32 | 29 | 1 |
| 247 | - | 272047 | 271601 | - | *-* | *PA14_03080* | acetyltransferase | 80 | 46 | 33 | 45 | 39 | 93 | 0.810957915 |
| 248 | - | 272559 | 272125 | - | *-* | *PA14_03090* | hypothetical protein | 423 | 121 | 124 | 144 | 129 | 377 | 0.015343712 |
| 249 | - | 273365 | 272724 | - | *-* | *PA14_03100* | hypothetical protein | 8 | 7 | 8 | 7 | 6 | 5 | 1 |
| 250 | - | 273920 | 273633 | - | *-* | *PA14_03110* | hypothetical protein | 5 | 4 | 5 | 3 | 4 | 4 | 1 |
| 251 | - | 274488 | 274012 | - | *-* | *PA14_03120* | transcriptional regulator | 35 | 28 | 30 | 31 | 24 | 30 | 1 |
| 252 | - | 276019 | 274529 | - | *-* | *PA14_03130* | hypothetical protein | 56 | 48 | 48 | 57 | 49 | 46 | 1 |

|  | A | B | C | D | E | F | G | H | I | J | K | L | M | N |
| --- | --- | --- | --- | --- | --- | --- | --- | --- | --- | --- | --- | --- | --- | --- |
| 253 | - | 276839 | 276153 | - | *-* | *PA14_03150* | hypothetical protein | 113 | 111 | 115 | 123 | 113 | 100 | 1 |
| 254 | - | 277935 | 277003 | - | *-* | *PA14_03160* | hypothetical protein | 110 | 106 | 137 | 142 | 109 | 137 | 1 |
| 255 | - | 278510 | 279133 | + | *-* | *PA14_03163* | hypothetical protein | 715 | 666 | 627 | 614 | 540 | 645 | 1 |
| 256 | - | 279133 | 279531 | + | *-* | *PA14_03166* | hypothetical protein | 119 | 111 | 119 | 116 | 155 | 140 | 1 |
| 257 | - | 280557 | 279718 | - | *-* | *PA14_03170* | hypothetical protein | 46 | 46 | 38 | 42 | 48 | 39 | 1 |
| 258 | - | 280853 | 280590 | - | *-* | *PA14_03180* | hypothetical protein | 108 | 124 | 97 | 118 | 137 | 112 | 1 |
| 259 | - | 282415 | 280973 | - | *-* | *PA14_03190* | hypothetical protein | 87 | 79 | 76 | 80 | 86 | 79 | 1 |
| 260 | - | 284715 | 282565 | - | *-* | *PA14_03200* | hypothetical protein | 31 | 30 | 28 | 26 | 35 | 26 | 1 |
| 261 | - | 285209 | 284712 | - | *-* | *PA14_03210* | hypothetical protein | 24 | 20 | 22 | 18 | 27 | 19 | 1 |
| 262 | - | 288272 | 285213 | - | *-* | *PA14_03220* | hypothetical protein | 31 | 26 | 24 | 23 | 28 | 21 | 1 |
| 263 | - | 288972 | 288454 | - | *hcpC* | *PA14_03240* | secreted protein Hcp | 33 | 19 | 18 | 20 | 21 | 22 | 0.580848337 |
| 264 | - | 291568 | 293184 | + | *-* | *PA14_03250* | hypothetical protein | 70 | 67 | 63 | 58 | 64 | 56 | 1 |
| 265 | - | 293187 | 293936 | + | *-* | *PA14_03265* | hypothetical protein | 82 | 82 | 66 | 65 | 86 | 69 | 1 |
| 266 | - | 294101 | 297238 | + | *-* | *PA14_03270* | hypothetical protein | 101 | 94 | 77 | 81 | 98 | 85 | 1 |
| 267 | - | 297222 | 298367 | + | *-* | *PA14_03285* | hypothetical protein | 63 | 57 | 50 | 51 | 62 | 55 | 1 |
| 268 | - | 298567 | 298875 | + | *-* | *PA14_03290* | hypothetical protein | 461 | 431 | 441 | 441 | 406 | 406 | 1 |
| 269 | - | 298872 | 299717 | + | *-* | *PA14_03300* | hypothetical protein | 123 | 111 | 115 | 119 | 115 | 111 | 1 |
| 270 | - | 300333 | 301931 | + | *-* | *PA14_03310* | hypothetical protein | 41 | 47 | 45 | 46 | 46 | 44 | 1 |
| 271 | - | 303804 | 302683 | - | *-* | *PA14_03320* | hypothetical protein | 80 | 78 | 66 | 70 | 85 | 69 | 1 |
| 272 | - | 304676 | 303810 | - | *-* | *PA14_03330* | hypothetical protein | 26 | 30 | 24 | 22 | 34 | 25 | 1 |
| 273 | - | 305599 | 304673 | - | *-* | *PA14_03340* | hypothetical protein | 41 | 33 | 37 | 34 | 41 | 34 | 1 |
| 274 | - | 306903 | 305950 | - | *-* | *PA14_03350* | hypothetical protein | 165 | 185 | 128 | 148 | 182 | 151 | 1 |
| 275 | - | 307666 | 306896 | - | *-* | *PA14_03360* | hypothetical protein | 174 | 193 | 138 | 133 | 171 | 136 | 1 |
| 276 | - | 308983 | 307985 | - | *-* | *PA14_03370* | hypothetical protein | 88 | 88 | 79 | 83 | 101 | 79 | 1 |
| 277 | - | 309749 | 311728 | + | *-* | *PA14_03380* | hypothetical protein | 65 | 59 | 60 | 66 | 62 | 60 | 1 |
| 278 | - | 312174 | 311665 | - | *-* | *PA14_03390* | hypothetical protein | 11 | 13 | 12 | 9 | 11 | 9 | 1 |
| 279 | - | 312499 | 312167 | - | *-* | *PA14_03400* | hypothetical protein | 3 | 6 | 5 | 3 | 4 | 2 | 1 |
| 280 | - | 313034 | 312961 | - | *-* | *PA14_03410* | Arg tRNA | 18 | 14 | 15 | 8 | 12 | 13 | 1 |
| 281 | - | 313638 | 313222 | - | *-* | *PA14_03420* | hypothetical protein | 44 | 34 | 42 | 39 | 43 | 35 | 1 |
| 282 | - | 313663 | 315114 | + | *gabD* | *PA14_03430* | succinate-semialdehyde dehydrogenase I | 158 | 188 | 140 | 164 | 153 | 162 | 1 |
| 283 | - | 315359 | 316639 | + | *gabT* | *PA14_03450* | 4-aminobutyrate aminotransferase | 174 | 219 | 122 | 164 | 187 | 184 | 1 |
| 284 | - | 316965 | 318164 | + | *-* | *PA14_03470* | hypothetical protein | 75 | 81 | 76 | 80 | 82 | 69 | 1 |
| 285 | - | 319753 | 318332 | - | *-* | *PA14_03480* | GntR family transcriptional regulator | 15 | 20 | 16 | 18 | 19 | 17 | 1 |
| 286 | - | 319880 | 320317 | + | *-* | *PA14_03490* | hypothetical protein | 78 | 56 | 42 | 52 | 46 | 102 | 1 |
| 287 | - | 320329 | 320736 | + | *-* | *PA14_03510* | hypothetical protein | 98 | 72 | 52 | 74 | 83 | 145 | 1 |
| 288 | - | 320770 | 321054 | + | *-* | *PA14_03520* | hypothetical protein | 127 | 63 | 62 | 91 | 83 | 183 | 0.321350893 |
| 289 | - | 321983 | 321051 | - | *-* | *PA14_03530* | transcriptional regulator | 57 | 30 | 32 | 45 | 38 | 90 | 0.8338713 |
| 290 | - | 323247 | 322033 | - | *-* | *PA14_03550* | MFS transporter | 7 | 5 | 6 | 6 | 4 | 6 | 1 |
| 291 | - | 324180 | 323410 | - | *-* | *PA14_03560* | hypothetical protein | 5 | 5 | 4 | 4 | 6 | 4 | 1 |
| 292 | - | 324975 | 324289 | - | *-* | *PA14_03580* | transcriptional regulator | 44 | 35 | 41 | 32 | 31 | 37 | 1 |
| 293 | - | 325051 | 325566 | + | *-* | *PA14_03590* | hypothetical protein | 81 | 47 | 67 | 53 | 45 | 75 | 1 |
| 294 | - | 326364 | 325606 | - | *-* | *PA14_03610* | Zn-dependent protease with chaperone function | 31 | 20 | 29 | 23 | 23 | 35 | 1 |
| 295 | - | 327288 | 326536 | - | *-* | *PA14_03620* | hypothetical protein | 9 | 7 | 8 | 8 | 8 | 8 | 1 |
| 296 | - | 327382 | 328080 | + | *-* | *PA14_03630* | ArsR family transcriptional regulator | 39 | 34 | 37 | 32 | 33 | 31 | 1 |
| 297 | - | 329082 | 328093 | - | *cysA* | *PA14_03650* | sulfate transport protein CysA | 40 | 35 | 14 | 15 | 26 | 15 | 1 |
| 298 | - | 329955 | 329086 | - | *cysW* | *PA14_03670* | sulfate transport protein CysW | 33 | 28 | 14 | 17 | 25 | 12 | 1 |
| 299 | - | 330784 | 329966 | - | *cysT* | *PA14_03680* | sulfate transport protein CysT | 19 | 18 | 9 | 10 | 11 | 10 | 1 |
| 300 | - | 331944 | 330946 | - | *sbp* | *PA14_03700* | sulfate-binding protein | 14 | 35 | 12 | 10 | 19 | 10 | 0.070186394 |
| 301 | - | 332303 | 332121 | - | *-* | *PA14_03710* | hypothetical protein | 40 | 105 | 49 | 42 | 54 | 37 | 0.022359242 |
| 302 | - | 334749 | 332467 | - | *-* | *PA14_03720* | sensory box GGDEF domain-containing protein | 23 | 25 | 22 | 23 | 23 | 20 | 1 |
| 303 | - | 334920 | 336098 | + | *-* | *PA14_03730* | fatty acid desaturase | 215 | 181 | 203 | 132 | 153 | 140 | 1 |
| 304 | - | 336327 | 337712 | + | *-* | *PA14_03760* | sodium:solute symporter | 7 | 6 | 6 | 6 | 8 | 6 | 1 |
| 305 | - | 337768 | 338724 | + | *speB1* | *PA14_03770* | agmatinase | 5 | 7 | 5 | 7 | 5 | 4 | 1 |
| 306 | - | 338777 | 339739 | + | *-* | *PA14_03780* | transcriptional regulator | 34 | 29 | 31 | 30 | 29 | 24 | 1 |
| 307 | - | 339852 | 340823 | + | *-* | *PA14_03790* | sensory box GGDEF domain-containing protein | 106 | 112 | 143 | 146 | 98 | 107 | 1 |
| 308 | - | 341437 | 342828 | + | *oprE* | *PA14_03800* | anaerobically-induced outer membrane porin OprE precursor | 169 | 253 | 124 | 120 | 186 | 134 | 1 |
| 309 | - | 344032 | 342926 | - | *aguA* | *PA14_03810* | agmatine deiminase | 45 | 55 | 35 | 32 | 47 | 38 | 1 |
| 310 | - | 344996 | 344118 | - | *aguB* | *PA14_03830* | N-carbamoylputrescine amidohydrolase | 11 | 12 | 11 | 8 | 9 | 9 | 1 |
| 311 | - | 345824 | 345159 | - | *aguR* | *PA14_03840* | transcriptional regulator AguR | 17 | 19 | 20 | 20 | 19 | 20 | 1 |
| 312 | - | 346948 | 345887 | - | *-* | *PA14_03855* | periplasmic polyamine binding protein | 38 | 39 | 46 | 34 | 31 | 30 | 1 |
| 313 | - | 348580 | 347204 | - | *-* | *PA14_03860* | glutamine synthetase | 167 | 187 | 157 | 181 | 149 | 173 | 1 |
| 314 | - | 348859 | 349611 | + | *spuA* | *PA14_03870* | glutamine amidotransferase | 92 | 94 | 101 | 81 | 79 | 85 | 1 |
| 315 | - | 349652 | 351010 | + | *spuB* | *PA14_03880* | glutamine synthetase | 107 | 136 | 95 | 130 | 101 | 104 | 1 |
| 316 | - | 351076 | 352446 | + | *spuC* | *PA14_03900* | aminotransferase | 327 | 398 | 274 | 335 | 314 | 289 | 1 |
| 317 | - | 352562 | 353665 | + | *spuD* | *PA14_03920* | polyamine transport protein | 272 | 320 | 206 | 249 | 278 | 248 | 1 |
| 318 | - | 354084 | 355181 | + | *spuE* | *PA14_03930* | polyamine transport protein | 117 | 130 | 108 | 106 | 111 | 108 | 1 |
| 319 | - | 355236 | 356390 | + | *spuF* | *PA14_03940* | polyamine transport protein PotG | 136 | 181 | 117 | 154 | 169 | 148 | 1 |
| 320 | - | 356417 | 357298 | + | *spuG* | *PA14_03950* | polyamine transport protein PotH | 119 | 142 | 99 | 122 | 134 | 126 | 1 |
| 321 | - | 357381 | 358250 | + | *spuH* | *PA14_03960* | polyamine transport protein PotI | 191 | 187 | 149 | 161 | 175 | 163 | 1 |
| 322 | - | 360835 | 358448 | - | *-* | *PA14_03980* | hypothetical protein | 24 | 27 | 21 | 21 | 25 | 22 | 1 |
| 323 | - | 360946 | 361998 | + | *-* | *PA14_04000* | transcriptional regulator | 52 | 68 | 57 | 53 | 67 | 55 | 1 |
| 324 | - | 362480 | 361977 | - | *-* | *PA14_04010* | hypothetical protein | 169 | 241 | 194 | 141 | 183 | 159 | 1 |
| 325 | - | 363195 | 364214 | + | *-* | *PA14_04030* | hypothetical protein | 43 | 43 | 37 | 37 | 34 | 31 | 1 |
| 326 | - | 363196 | 362585 | - | *-* | *PA14_04020* | hypothetical protein | 50 | 44 | 56 | 52 | 42 | 43 | 1 |
| 327 | - | 364986 | 364234 | - | *-* | *PA14_04040* | hypothetical protein | 73 | 63 | 68 | 60 | 55 | 57 | 1 |
| 328 | - | 365035 | 365733 | + | *-* | *PA14_04050* | proline hydroxylase | 54 | 54 | 65 | 62 | 52 | 56 | 1 |
| 329 | - | 366309 | 365755 | - | *-* | *PA14_04060* | hypothetical protein | 7 | 5 | 9 | 7 | 5 | 5 | 1 |
| 330 | - | 366575 | 367069 | + | *-* | *PA14_04070* | hypothetical protein | 149 | 154 | 184 | 195 | 141 | 149 | 1 |
| 331 | - | 367766 | 367074 | - | *-* | *PA14_04080* | ABC transporter permease | 61 | 68 | 60 | 71 | 62 | 62 | 1 |
| 332 | - | 368608 | 367838 | - | *-* | *PA14_04090* | ABC transporter substrate-binding protein | 51 | 56 | 55 | 50 | 65 | 54 | 1 |
| 333 | - | 368901 | 369338 | + | *-* | *PA14_04100* | hypothetical protein | 185 | 141 | 120 | 153 | 153 | 212 | 1 |
| 334 | - | 370625 | 369396 | - | *serA* | *PA14_04110* | D-3-phosphoglycerate dehydrogenase | 105 | 119 | 94 | 88 | 103 | 88 | 1 |
| 335 | - | 370829 | 372223 | + | *-* | *PA14_04140* | hypothetical protein | 88 | 92 | 97 | 85 | 91 | 75 | 1 |
| 336 | - | 372315 | 372980 | + | *-* | *PA14_04150* | hypothetical protein | 216 | 226 | 186 | 155 | 167 | 180 | 1 |

|  | A | B | C | D | E | F | G | H | I | J | K | L | M | N |
| --- | --- | --- | --- | --- | --- | --- | --- | --- | --- | --- | --- | --- | --- | --- |
| 337 | - | 373079 | 374068 | + | *-* | *PA14_04160* | transcriptional regulator | 57 | 58 | 58 | 60 | 59 | 57 | 1 |
| 338 | - | 374152 | 374502 | + | *-* | *PA14_04180* | hypothetical protein | 19 | 27 | 32 | 17 | 12 | 8 | 1 |
| 339 | - | 375617 | 374583 | - | *-* | *PA14_04190* | acetylpolyamine aminohydrolase | 18 | 17 | 20 | 19 | 17 | 18 | 1 |
| 340 | - | 377027 | 375633 | - | *-* | *PA14_04210* | transporter | 10 | 9 | 11 | 10 | 9 | 9 | 1 |
| 341 | - | 378491 | 377448 | - | *-* | *PA14_04220* | ABC transporter substrate-binding protein | 12 | 12 | 12 | 13 | 10 | 11 | 1 |
| 342 | - | 379327 | 378539 | - | *-* | *PA14_04230* | ABC transporter permease | 13 | 15 | 14 | 17 | 12 | 15 | 1 |
| 343 | - | 380253 | 379324 | - | *-* | *PA14_04240* | ABC transporter permease | 42 | 41 | 44 | 49 | 44 | 50 | 1 |
| 344 | - | 381299 | 380250 | - | *-* | *PA14_04250* | ABC transporter ATP-binding protein | 5 | 4 | 4 | 4 | 5 | 4 | 1 |
| 345 | - | 381627 | 382592 | + | *-* | *PA14_04270* | transcriptional regulator | 38 | 34 | 43 | 42 | 36 | 40 | 1 |
| 346 | - | 384574 | 382631 | - | *-* | *PA14_04290* | hypothetical protein | 37 | 41 | 40 | 42 | 39 | 35 | 1 |
| 347 | - | 385207 | 384875 | - | *-* | *PA14_04300* | hypothetical protein | 199 | 271 | 222 | 214 | 190 | 231 | 1 |
| 348 | - | 386001 | 385330 | - | *rpiA* | *PA14_04310* | ribose-5-phosphate isomerase A | 71 | 62 | 87 | 59 | 49 | 53 | 1 |
| 349 | - | 386260 | 387774 | + | *ilvA1* | *PA14_04320* | threonine dehydratase | 64 | 62 | 67 | 56 | 63 | 48 | 1 |
| 350 | - | 387894 | 388361 | + | *-* | *PA14_04330* | hypothetical protein | 213 | 198 | 252 | 215 | 194 | 197 | 1 |
| 351 | - | 388412 | 389683 | + | *-* | *PA14_04340* | hypothetical protein | 68 | 55 | 68 | 61 | 63 | 63 | 1 |
| 352 | - | 389680 | 390147 | + | *-* | *PA14_04350* | integral membrane protein | 96 | 100 | 94 | 107 | 104 | 93 | 1 |
| 353 | - | 391361 | 390120 | - | *-* | *PA14_04370* | MFS transporter | 45 | 40 | 45 | 35 | 37 | 40 | 1 |
| 354 | - | 392064 | 391411 | - | *-* | *PA14_04380* | hypothetical protein | 54 | 44 | 55 | 46 | 43 | 37 | 1 |
| 355 | - | 392268 | 392747 | + | *ygdP* | *PA14_04390* | dinucleoside polyphosphate hydrolase | 402 | 373 | 413 | 418 | 416 | 379 | 1 |
| 356 | - | 392770 | 395049 | + | *ptsP* | *PA14_04410* | phosphoenolpyruvate-protein phosphotransferase PtsP | 405 | 427 | 364 | 411 | 376 | 392 | 1 |
| 357 | - | 395075 | 396205 | + | *-* | *PA14_04420* | hypothetical protein | 51 | 47 | 52 | 50 | 41 | 41 | 1 |
| 358 | - | 396964 | 396209 | - | *-* | *PA14_04430* | hypothetical protein | 44 | 41 | 49 | 43 | 39 | 39 | 1 |
| 359 | - | 397086 | 397889 | + | *-* | *PA14_04440* | permease | 27 | 21 | 30 | 28 | 24 | 24 | 1 |
| 360 | - | 397899 | 398699 | + | *lgt* | *PA14_04460* | prolipoprotein diacylglyceryl transferase | 55 | 73 | 60 | 60 | 65 | 49 | 1 |
| 361 | - | 398905 | 399699 | + | *thyA* | *PA14_04480* | thymidylate synthase | 66 | 65 | 75 | 68 | 71 | 67 | 1 |
| 362 | - | 400523 | 399726 | - | *-* | *PA14_04490* | hypothetical protein | 73 | 73 | 77 | 69 | 82 | 74 | 1 |
| 363 | - | 401937 | 400558 | - | *-* | *PA14_04510* | hypothetical protein | 104 | 116 | 102 | 123 | 118 | 120 | 1 |
| 364 | - | 403315 | 401930 | - | *-* | *PA14_04520* | hypothetical protein | 68 | 60 | 67 | 76 | 64 | 68 | 1 |
| 365 | - | 403507 | 403869 | + | *-* | *PA14_04530* | hypothetical protein | 53 | 55 | 50 | 44 | 48 | 41 | 1 |
| 366 | - | 405057 | 403906 | - | *glpQ* | *PA14_04550* | glycerophosphoryl diester phosphodiesterase, periplasmic | 19 | 21 | 20 | 21 | 21 | 19 | 1 |
| 367 | - | 405267 | 406361 | + | *-* | *PA14_04560* | hypothetical protein | 18 | 20 | 21 | 21 | 17 | 17 | 1 |
| 368 | - | 406358 | 407398 | + | *-* | *PA14_04570* | hypothetical protein | 14 | 14 | 15 | 15 | 14 | 14 | 1 |
| 369 | - | 407469 | 407975 | + | *folA* | *PA14_04580* | dihydrofolate reductase | 22 | 33 | 28 | 23 | 27 | 23 | 1 |
| 370 | - | 408464 | 407991 | - | *-* | *PA14_04590* | hypothetical protein | 51 | 70 | 64 | 45 | 60 | 46 | 1 |
| 371 | - | 409988 | 408603 | - | *-* | *PA14_04610* | transporter | 11 | 12 | 15 | 10 | 11 | 8 | 1 |
| 372 | - | 412056 | 410218 | - | *ilvD* | *PA14_04630* | dihydroxy-acid dehydratase | 102 | 118 | 101 | 87 | 112 | 99 | 1 |
| 373 | - | 413580 | 412384 | - | *-* | *PA14_04640* | hypothetical protein | 59 | 52 | 69 | 55 | 55 | 60 | 1 |
| 374 | - | 414192 | 413653 | - | *pfpI* | *PA14_04650* | protease PfpI | 110 | 149 | 114 | 97 | 142 | 115 | 1 |
| 375 | - | 414409 | 415233 | + | *-* | *PA14_04660* | hypothetical protein | 28 | 23 | 33 | 19 | 20 | 18 | 1 |
| 376 | - | 415292 | 416104 | + | *mutM* | *PA14_04670* | formamidopyrimidine-DNA glycosylase | 62 | 56 | 60 | 55 | 58 | 45 | 1 |
| 377 | - | 416181 | 416759 | + | *-* | *PA14_04680* | hypothetical protein | 24 | 24 | 24 | 25 | 22 | 21 | 1 |
| 378 | - | 416842 | 417186 | + | *-* | *PA14_04690* | hypothetical protein | 136 | 161 | 184 | 98 | 131 | 123 | 1 |
| 379 | - | 417456 | 418442 | + | *-* | *PA14_04700* | hypothetical protein | 80 | 83 | 79 | 84 | 87 | 80 | 1 |
| 380 | - | 418986 | 418555 | - | *-* | *PA14_04710* | hypothetical protein | 76 | 78 | 80 | 59 | 79 | 64 | 1 |
| 381 | - | 420871 | 419138 | - | *-* | *PA14_04730* | gamma-glutamyltranspeptidase | 57 | 50 | 62 | 57 | 47 | 48 | 1 |
| 382 | - | 421250 | 420999 | - | *fdx1* | *PA14_04750* | ferredoxin (4Fe-4S) | 74 | 63 | 80 | 53 | 54 | 48 | 1 |
| 383 | - | 421850 | 421371 | - | *coaD* | *PA14_04760* | phosphopantetheine adenylyltransferase | 28 | 23 | 30 | 26 | 21 | 29 | 1 |
| 384 | - | 423594 | 421999 | - | *-* | *PA14_04780* | oxidoreductase | 33 | 27 | 27 | 36 | 27 | 38 | 1 |
| 385 | - | 424198 | 423650 | - | *-* | *PA14_04790* | hypothetical protein | 42 | 43 | 42 | 57 | 39 | 58 | 1 |
| 386 | - | 425683 | 424253 | - | *-* | *PA14_04810* | aldehyde dehydrogenase | 73 | 64 | 65 | 91 | 61 | 94 | 1 |
| 387 | - | 425972 | 426619 | + | *-* | *PA14_04820* | TetR family transcriptional regulator | 66 | 66 | 75 | 76 | 64 | 70 | 1 |
| 388 | - | 427014 | 426604 | - | *-* | *PA14_04830* | acetyltransferase | 207 | 211 | 181 | 209 | 254 | 229 | 1 |
| 389 | - | 428075 | 427077 | - | *-* | *PA14_04840* | hypothetical protein | 65 | 47 | 54 | 52 | 56 | 52 | 1 |
| 390 | - | 428402 | 428112 | - | *-* | *PA14_04850* | hypothetical protein | 83 | 77 | 82 | 75 | 93 | 85 | 1 |
| 391 | - | 429277 | 428681 | - | *-* | *PA14_04860* | methyltransferase | 98 | 107 | 85 | 95 | 101 | 83 | 1 |
| 392 | - | 430764 | 429277 | - | *-* | *PA14_04870* | hypothetical protein | 93 | 118 | 94 | 96 | 114 | 95 | 1 |
| 393 | - | 432154 | 430757 | - | *-* | *PA14_04890* | zinc protease | 53 | 50 | 56 | 46 | 50 | 50 | 1 |
| 394 | - | 432275 | 433630 | + | *ftsY* | *PA14_04900* | signal recognition particle receptor FtsY | 152 | 127 | 165 | 153 | 145 | 133 | 1 |
| 395 | - | 433627 | 434298 | + | *ftsE* | *PA14_04910* | cell division ATP-binding protein FtsE | 181 | 197 | 197 | 211 | 209 | 184 | 1 |
| 396 | - | 434298 | 435305 | + | *ftsX* | *PA14_04920* | cell division protein FtsX | 203 | 182 | 198 | 212 | 209 | 180 | 1 |
| 397 | - | 435419 | 436273 | + | *rpoH* | *PA14_04930* | RNA polymerase factor sigma-32 | 677 | 557 | 773 | 497 | 455 | 473 | 1 |
| 398 | - | 436338 | 436943 | + | *-* | *PA14_04940* | hypothetical protein | 97 | 105 | 101 | 108 | 114 | 87 | 1 |
| 399 | - | 437679 | 436948 | - | *mtgA* | *PA14_04950* | monofunctional biosynthetic peptidoglycan transglycosylase | 43 | 45 | 52 | 45 | 42 | 36 | 1 |
| 400 | - | 437716 | 438093 | + | *-* | *PA14_04960* | hypothetical protein | 41 | 51 | 41 | 53 | 41 | 40 | 1 |
| 401 | - | 438196 | 438396 | + | *-* | *PA14_04970* | sulfur carrier protein ThiS | 33 | 26 | 31 | 28 | 22 | 20 | 1 |
| 402 | - | 438455 | 439252 | + | *thiG* | *PA14_04980* | thiazole synthase | 34 | 39 | 34 | 24 | 24 | 22 | 1 |
| 403 | - | 439406 | 440077 | + | *trmB* | *PA14_05000* | tRNA (guanine-N(7)-)-methyltransferase | 37 | 32 | 43 | 33 | 33 | 25 | 1 |
| 404 | - | 440240 | 441586 | + | *-* | *PA14_05010* | hypothetical protein | 31 | 34 | 37 | 43 | 31 | 35 | 1 |
| 405 | - | 441856 | 441599 | - | *-* | *PA14_05020* | hypothetical protein | 22 | 29 | 27 | 25 | 26 | 36 | 1 |
| 406 | - | 442241 | 441918 | - | *-* | *PA14_05030* | hypothetical protein | 14 | 15 | 19 | 10 | 10 | 12 | 1 |
| 407 | - | 443476 | 442322 | - | *-* | *PA14_05040* | coproporphyrinogen III oxidase | 20 | 15 | 23 | 17 | 14 | 16 | 1 |
| 408 | - | 444190 | 443597 | - | *-* | *PA14_05050* | deoxyribonucleotide triphosphate pyrophosphatase | 226 | 240 | 238 | 201 | 225 | 218 | 1 |
| 409 | - | 444606 | 444187 | - | *-* | *PA14_05060* | hypothetical protein | 320 | 340 | 412 | 274 | 301 | 316 | 1 |
| 410 | - | 445358 | 444738 | - | *-* | *PA14_05070* | methionine biosynthesis protein | 21 | 28 | 34 | 21 | 24 | 23 | 1 |
| 411 | - | 446505 | 445366 | - | *metX* | *PA14_05080* | homoserine O-acetyltransferase | 18 | 17 | 21 | 19 | 16 | 16 | 1 |
| 412 | - | 448561 | 446594 | - | *-* | *PA14_05110* | hypothetical protein | 51 | 75 | 58 | 68 | 52 | 46 | 1 |
| 413 | - | 448839 | 448669 | - | *-* | *PA14_05120* | hypothetical protein | 42 | 44 | 34 | 26 | 39 | 36 | 1 |
| 414 | - | 449555 | 448962 | - | *-* | *PA14_05130* | hypothetical protein | 99 | 111 | 91 | 80 | 101 | 85 | 1 |
| 415 | - | 450387 | 449566 | - | *proC* | *PA14_05150* | pyrroline-5-carboxylate reductase | 186 | 192 | 182 | 149 | 151 | 151 | 1 |
| 416 | - | 451091 | 450399 | - | *-* | *PA14_05160* | hypothetical protein | 86 | 78 | 80 | 58 | 66 | 61 | 1 |
| 417 | - | 451306 | 452340 | + | *pilT* | *PA14_05180* | twitching motility protein PilT | 123 | 122 | 146 | 147 | 133 | 136 | 1 |
| 418 | - | 452518 | 453666 | + | *pilU* | *PA14_05190* | twitching motility protein PilU | 252 | 218 | 307 | 283 | 213 | 246 | 1 |
| 419 | - | 454573 | 453674 | - | *-* | *PA14_05200* | cation efflux system protein | 76 | 78 | 96 | 95 | 78 | 87 | 1 |
| 420 | - | 454728 | 455132 | + | *-* | *PA14_05210* | hypothetical protein | 31 | 46 | 30 | 20 | 24 | 33 | 1 |

|  | A | B | C | D | E | F | G | H | I | J | K | L | M | N |
| --- | --- | --- | --- | --- | --- | --- | --- | --- | --- | --- | --- | --- | --- | --- |
| 421 | - | 455375 | 456748 | + | *-* | *PA14_05220* | cystathionine beta-synthase | 119 | 154 | 101 | 101 | 144 | 106 | 1 |
| 422 | - | 456745 | 457929 | + | *-* | *PA14_05230* | cystathionine gamma-lyase | 111 | 151 | 82 | 86 | 131 | 94 | 1 |
| 423 | - | 459428 | 458157 | - | *pyrC* | *PA14_05250* | dihydroorotase | 100 | 107 | 88 | 85 | 102 | 85 | 1 |
| 424 | - | 460429 | 459425 | - | *pyrB* | *PA14_05260* | aspartate carbamoyltransferase | 139 | 136 | 131 | 119 | 131 | 115 | 1 |
| 425 | - | 460965 | 460453 | - | *pyrR* | *PA14_05270* | bifunctional pyrimidine regulatory protein PyrR/uracil phosphoribosyltransferase | 123 | 136 | 130 | 96 | 110 | 119 | 1 |
| 426 | - | 461511 | 461077 | - | *yqgF* | *PA14_05280* | Holliday junction resolvase-like protein | 67 | 68 | 63 | 62 | 64 | 57 | 1 |
| 427 | - | 462080 | 461511 | - | *-* | *PA14_05290* | hypothetical protein | 63 | 73 | 68 | 58 | 57 | 59 | 1 |
| 428 | - | 463088 | 462129 | - | *-* | *PA14_05300* | TonB domain-containing protein | 37 | 37 | 37 | 40 | 31 | 32 | 1 |
| 429 | - | 464122 | 463169 | - | *gshB* | *PA14_05310* | glutathione synthetase | 316 | 416 | 483 | 436 | 339 | 282 | 1 |
| 430 | - | 464377 | 464784 | + | *pilG* | *PA14_05320* | twitching motility protein PilG | 418 | 400 | 545 | 438 | 469 | 543 | 1 |
| 431 | - | 464831 | 465196 | + | *pilH* | *PA14_05330* | twitching motility protein PilH | 191 | 206 | 257 | 212 | 214 | 229 | 1 |
| 432 | - | 465247 | 465783 | + | *pilI* | *PA14_05340* | twitching motility protein PilI | 150 | 146 | 202 | 162 | 166 | 156 | 1 |
| 433 | - | 465868 | 467916 | + | *pilJ* | *PA14_05360* | twitching motility protein PilJ | 288 | 377 | 313 | 299 | 333 | 285 | 1 |
| 434 | - | 467977 | 468852 | + | *pilK* | *PA14_05380* | methyltransferase PilK | 199 | 220 | 218 | 253 | 241 | 219 | 1 |
| 435 | - | 468864 | 476294 | + | *chpA* | *PA14_05390* | ChpA | 339 | 383 | 350 | 374 | 402 | 364 | 1 |
| 436 | - | 476287 | 477318 | + | *-* | *PA14_05400* | methylesterase | 274 | 299 | 297 | 277 | 350 | 304 | 1 |
| 437 | - | 477315 | 477821 | + | *-* | *PA14_05410* | chemotaxis protein | 131 | 182 | 143 | 161 | 197 | 160 | 1 |
| 438 | - | 477829 | 478623 | + | *-* | *PA14_05420* | transcriptional regulator | 51 | 62 | 56 | 55 | 67 | 55 | 1 |
| 439 | - | 478700 | 479311 | + | *-* | *PA14_05430* | chemotaxis protein | 15 | 18 | 20 | 17 | 19 | 14 | 1 |
| 440 | - | 480734 | 479319 | - | *-* | *PA14_05440* | hypothetical protein | 21 | 26 | 20 | 22 | 24 | 17 | 1 |
| 441 | - | 481491 | 480769 | - | *-* | *PA14_05450* | 16S ribosomal RNA methyltransferase RsmE | 33 | 31 | 38 | 24 | 26 | 24 | 1 |
| 442 | - | 482987 | 481584 | - | *bioA* | *PA14_05460* | adenosylmethionine-8-amino-7-oxononanoate aminotransferase | 29 | 32 | 25 | 29 | 29 | 27 | 1 |
| 443 | - | 483161 | 484651 | + | *-* | *PA14_05480* | hypothetical protein | 63 | 68 | 63 | 61 | 60 | 49 | 1 |
| 444 | - | 484832 | 485401 | + | *-* | *PA14_05500* | hypothetical protein | 111 | 62 | 108 | 55 | 71 | 67 | 0.956402005 |
| 445 | - | 485413 | 485988 | + | *-* | *PA14_05510* | hypothetical protein | 316 | 488 | 235 | 201 | 302 | 259 | 1 |
| 446 | - | 486500 | 486057 | - | *mexR* | *PA14_05520* | multidrug resistance operon repressor MexR | 215 | 205 | 251 | 350 | 229 | 271 | 1 |
| 447 | - | 486775 | 487926 | + | *mexA* | *PA14_05530* | RND multidrug efflux membrane fusion protein MexA | 264 | 212 | 230 | 200 | 179 | 195 | 1 |
| 448 | - | 487942 | 491082 | + | *mexB* | *PA14_05540* | RND multidrug efflux transporter MexB | 499 | 401 | 467 | 420 | 394 | 387 | 1 |
| 449 | - | 491084 | 492541 | + | *oprM* | *PA14_05550* | major intrinsic multiple antibiotic resistance efflux outer membrane protein OprM precursor | 599 | 504 | 695 | 446 | 415 | 415 | 1 |
| 450 | - | 494556 | 492637 | - | *-* | *PA14_05560* | ATP-dependent RNA helicase | 238 | 184 | 221 | 171 | 165 | 186 | 1 |
| 451 | - | 495877 | 494807 | - | *-* | *PA14_05580* | hypothetical protein | 124 | 105 | 118 | 97 | 92 | 118 | 1 |
| 452 | - | 496819 | 495947 | - | *metF* | *PA14_05590* | 5,10-methylenetetrahydrofolate reductase | 192 | 111 | 131 | 111 | 98 | 155 | 1 |
| 453 | - | 497416 | 496862 | - | *-* | *PA14_05600* | hypothetical protein | 169 | 98 | 101 | 78 | 80 | 121 | 1 |
| 454 | - | 498866 | 497457 | - | *sahH* | *PA14_05620* | S-adenosyl-L-homocysteine hydrolase | 471 | 305 | 282 | 264 | 257 | 392 | 1 |
| 455 | - | 499155 | 499589 | + | *-* | *PA14_05630* | hypothetical protein | 20 | 16 | 20 | 18 | 15 | 13 | 1 |
| 456 | - | 499715 | 501907 | + | *-* | *PA14_05640* | hypothetical protein | 23 | 18 | 22 | 20 | 17 | 17 | 1 |
| 457 | - | 501918 | 503396 | + | *-* | *PA14_05650* | hypothetical protein | 12 | 9 | 12 | 9 | 10 | 8 | 1 |
| 458 | - | 503481 | 504101 | + | *-* | *PA14_05660* | transcriptional regulator | 203 | 133 | 156 | 171 | 187 | 272 | 1 |
| 459 | - | 505409 | 504138 | - | *codA* | *PA14_05690* | cytosine deaminase | 55 | 55 | 53 | 56 | 63 | 60 | 1 |
| 460 | - | 506649 | 505399 | - | *codB* | *PA14_05700* | cytosine permease | 14 | 13 | 12 | 12 | 14 | 12 | 1 |
| 461 | - | 508108 | 506831 | - | *-* | *PA14_05740* | dihydropyrimidine dehydrogenase | 21 | 7 | 17 | 9 | 6 | 20 | 0.003564822 |
| 462 | - | 509472 | 508105 | - | *-* | *PA14_05750* | oxidoreductase | 25 | 13 | 22 | 16 | 11 | 24 | 0.391278558 |
| 463 | - | 511006 | 509567 | - | *dhT* | *PA14_05770* | phenylhydantoinase | 46 | 17 | 34 | 18 | 11 | 30 | 0.053355548 |
| 464 | - | 511229 | 511113 | - | *-* | *PA14_05775* | hypothetical protein | 1 | 1 | 0 | 0 | 0 | 0 | 1 |
| 465 | - | 511622 | 513112 | + | *-* | *PA14_05790* | transporter | 10 | 6 | 9 | 8 | 7 | 11 | 1 |
| 466 | - | 513171 | 514454 | + | *amaB* | *PA14_05810* | allantoate amidohydrolase | 56 | 39 | 63 | 43 | 38 | 65 | 1 |
| 467 | - | 515878 | 514655 | - | *-* | *PA14_05820* | hypothetical protein | 79 | 116 | 84 | 92 | 107 | 122 | 1 |
| 468 | - | 517179 | 515998 | - | *gcdH* | *PA14_05840* | glutaryl-CoA dehydrogenase | 219 | 365 | 262 | 213 | 325 | 313 | 1 |
| 469 | - | 517400 | 518308 | + | *-* | *PA14_05850* | LysR family transcriptional regulator | 33 | 32 | 31 | 34 | 30 | 31 | 1 |
| 470 | - | 518460 | 518864 | + | *-* | *PA14_05860* | hypothetical protein | 271 | 244 | 135 | 142 | 206 | 281 | 1 |
| 471 | - | 520529 | 518907 | - | *-* | *PA14_05870* | phosphate transporter | 17 | 15 | 13 | 12 | 14 | 18 | 1 |
| 472 | - | 520909 | 522240 | + | *-* | *PA14_05880* | membrane-bound protease | 225 | 214 | 518 | 274 | 287 | 315 | 1 |
| 473 | - | 522237 | 523031 | + | *-* | *PA14_05890* | stomatin-like protein | 28 | 34 | 45 | 34 | 37 | 44 | 1 |
| 474 | - | 523139 | 523870 | + | *-* | *PA14_05910* | periplasmic transport system | 42 | 43 | 49 | 46 | 44 | 45 | 1 |
| 475 | - | 526068 | 523867 | - | *-* | *PA14_05920* | hypothetical protein | 36 | 38 | 42 | 43 | 38 | 37 | 1 |
| 476 | - | 527706 | 526330 | - | *dbpA* | *PA14_05950* | ATP-dependent RNA helicase DbpA | 41 | 39 | 46 | 34 | 33 | 34 | 1 |
| 477 | - | 528054 | 528263 | + | *-* | *PA14_05960* | cold-shock protein | 1537 | 1697 | 1142 | 976 | 1077 | 994 | 1 |
| 478 | - | 528414 | 528938 | + | *-* | *PA14_05970* | hypothetical protein | 96 | 90 | 106 | 90 | 89 | 85 | 1 |
| 479 | - | 530474 | 529041 | - | *-* | *PA14_05990* | EmrB/QacA family drug resistance transporter | 51 | 50 | 55 | 50 | 49 | 43 | 1 |
| 480 | - | 531095 | 533647 | + | *-* | *PA14_06000* | ClpA/B protease ATP binding subunit | 539 | 578 | 757 | 555 | 456 | 565 | 1 |
| 481 | - | 533749 | 534327 | + | *-* | *PA14_06010* | hypothetical protein | 534 | 674 | 639 | 601 | 635 | 674 | 1 |
| 482 | - | 535279 | 535983 | + | *-* | *PA14_06040* | hypothetical protein | 82 | 70 | 75 | 59 | 75 | 70 | 1 |
| 483 | - | 535331 | 534444 | - | *-* | *PA14_06030* | acyltransferase | 129 | 136 | 158 | 143 | 136 | 138 | 1 |
| 484 | - | 536068 | 536757 | + | *creB* | *PA14_06060* | DNA-binding response regulator CreB | 22 | 17 | 20 | 17 | 18 | 18 | 1 |
| 485 | - | 536757 | 538181 | + | *creC* | *PA14_06070* | sensory histidine kinase CreC | 32 | 30 | 31 | 29 | 32 | 25 | 1 |
| 486 | - | 538283 | 539641 | + | *creD* | *PA14_06080* | hypothetical protein | 18 | 16 | 16 | 15 | 14 | 15 | 1 |
| 487 | - | 539691 | 539993 | + | *-* | *PA14_06090* | hypothetical protein | 13 | 13 | 13 | 15 | 13 | 11 | 1 |
| 488 | - | 540758 | 540138 | - | *-* | *PA14_06120* | hypothetical protein | 37 | 27 | 23 | 25 | 32 | 46 | 1 |
| 489 | - | 541727 | 540777 | - | *-* | *PA14_06130* | hypothetical protein | 144 | 82 | 93 | 86 | 93 | 129 | 1 |
| 490 | - | 541813 | 542670 | + | *-* | *PA14_06150* | hypothetical protein | 142 | 149 | 138 | 125 | 156 | 134 | 1 |
| 491 | - | 545236 | 542843 | - | *-* | *PA14_06160* | hydroxamate-type ferrisiderophore receptor | 20 | 23 | 24 | 37 | 29 | 22 | 1 |
| 492 | - | 546311 | 545340 | - | *-* | *PA14_06170* | transmembrane sensor | 9 | 28 | 14 | 90 | 53 | 17 | 0.002852411 |
| 493 | - | 546826 | 546308 | - | *-* | *PA14_06180* | RNA polymerase sigma factor | 10 | 58 | 19 | 235 | 178 | 30 | 5.65402E-21 |
| 494 | - | 546995 | 547744 | + | *-* | *PA14_06190* | glutathione S-transferase | 23 | 30 | 17 | 17 | 23 | 18 | 1 |
| 495 | - | 547884 | 548288 | + | *-* | *PA14_06200* | hypothetical protein | 2 | 2 | 2 | 2 | 2 | 1 | 1 |
| 496 | - | 548338 | 548907 | + | *-* | *PA14_06210* | transcriptional regulator | 22 | 21 | 16 | 19 | 19 | 17 | 1 |
| 497 | - | 550668 | 548941 | - | *-* | *PA14_06230* | permease | 40 | 36 | 36 | 48 | 38 | 45 | 1 |
| 498 | - | 551942 | 551016 | - | *-* | *PA14_06240* | LysR family transcriptional regulator | 20 | 17 | 20 | 17 | 18 | 18 | 1 |
| 499 | - | 552030 | 552506 | + | *-* | *PA14_06250* | GNAT family acetyltransferase | 41 | 43 | 43 | 42 | 45 | 42 | 1 |
| 500 | - | 553523 | 552585 | - | *-* | *PA14_06260* | LysR family transcriptional regulator | 61 | 56 | 54 | 56 | 49 | 51 | 1 |
| 501 | - | 553627 | 554424 | + | *-* | *PA14_06270* | hydrolase | 61 | 64 | 75 | 63 | 57 | 58 | 1 |
| 502 | - | 554467 | 554910 | + | *-* | *PA14_06280* | hypothetical protein | 48 | 47 | 56 | 67 | 45 | 50 | 1 |
| 503 | - | 557135 | 554958 | - | *glcB* | *PA14_06290* | malate synthase G | 228 | 182 | 148 | 145 | 144 | 142 | 1 |
| 504 | - | 557442 | 557885 | + | *-* | *PA14_06300* | GNAT family acetyltransferase | 133 | 106 | 130 | 124 | 113 | 129 | 1 |

|  | A | B | C | D | E | F | G | H | I | J | K | L | M | N |
| --- | --- | --- | --- | --- | --- | --- | --- | --- | --- | --- | --- | --- | --- | --- |
| 505 | - | 557917 | 558432 | + | *-* | *PA14_06310* | ACT domain-containing protein | 202 | 198 | 266 | 265 | 192 | 263 | 1 |
| 506 | 560072 | 560017 | 559121 | - | *-* | *PA14_06320* | hypothetical protein | 26 | 34 | 41 | 35 | 28 | 23 | 1 |
| 507 | - | 561194 | 560220 | - | *-* | *PA14_06330* | serine/threonine protein kinase | 25 | 25 | 27 | 20 | 21 | 21 | 1 |
| 508 | - | 562014 | 561256 | - | *-* | *PA14_06340* | molybdenum transport regulator | 49 | 37 | 43 | 32 | 44 | 37 | 1 |
| 509 | - | 562402 | 562082 | - | *-* | *PA14_06350* | hypothetical protein | 43 | 39 | 44 | 48 | 42 | 39 | 1 |
| 510 | - | 563169 | 562444 | - | *-* | *PA14_06360* | phosphoribosyl transferase | 18 | 13 | 12 | 12 | 15 | 13 | 1 |
| 511 | - | 563601 | 563308 | - | *-* | *PA14_06390* | hypothetical protein | 166 | 149 | 197 | 157 | 190 | 169 | 1 |
| 512 | - | 564581 | 563655 | - | *-* | *PA14_06400* | LysR family transcriptional regulator | 17 | 15 | 14 | 12 | 15 | 13 | 1 |
| 513 | - | 564699 | 565457 | + | *-* | *PA14_06420* | hypothetical protein | 29 | 18 | 16 | 18 | 17 | 18 | 1 |
| 514 | - | 565534 | 565782 | + | *-* | *PA14_06430* | hypothetical protein | 25 | 13 | 8 | 11 | 10 | 7 | 0.148048673 |
| 515 | - | 565793 | 567169 | + | *-* | *PA14_06450* | acetyl-CoA carboxylase biotin carboxylase subunit | 31 | 13 | 10 | 11 | 12 | 9 | 0.11934908 |
| 516 | - | 567171 | 568049 | + | *-* | *PA14_06460* | hypothetical protein | 40 | 19 | 14 | 16 | 19 | 17 | 0.215275599 |
| 517 | - | 568039 | 569016 | + | *-* | *PA14_06480* | hydrolase | 54 | 22 | 24 | 25 | 23 | 22 | 0.140519471 |
| 518 | - | 569617 | 570675 | + | *bioB* | *PA14_06500* | biotin synthase | 89 | 181 | 50 | 70 | 128 | 81 | 0.843937152 |
| 519 | - | 570781 | 571986 | + | *bioF* | *PA14_06510* | 8-amino-7-oxononanoate synthase | 49 | 63 | 36 | 35 | 48 | 45 | 1 |
| 520 | - | 571979 | 572701 | + | *-* | *PA14_06530* | biotin biosynthesis protein bioH | 82 | 90 | 59 | 60 | 72 | 75 | 1 |
| 521 | - | 572694 | 573518 | + | *-* | *PA14_06540* | biotin synthesis protein BioC | 66 | 71 | 40 | 47 | 63 | 58 | 1 |
| 522 | - | 573522 | 574208 | + | *bioD* | *PA14_06570* | dithiobiotin synthetase | 76 | 89 | 60 | 62 | 79 | 83 | 1 |
| 523 | - | 574317 | 574547 | + | *-* | *PA14_06580* | hypothetical protein | 268 | 283 | 380 | 231 | 271 | 262 | 1 |
| 524 | - | 574888 | 576693 | + | *-* | *PA14_06600* | acyl-CoA dehydrogenase | 326 | 263 | 364 | 222 | 277 | 333 | 1 |
| 525 | - | 576906 | 578702 | + | *-* | *PA14_06620* | acyl-CoA dehydrogenase | 21 | 21 | 17 | 17 | 17 | 17 | 1 |
| 526 | - | 578977 | 580755 | + | *-* | *PA14_06640* | acyl-CoA dehydrogenase | 24 | 23 | 19 | 22 | 25 | 22 | 1 |
| 527 | - | 582569 | 581088 | - | *nirN* | *PA14_06650* | c-type cytochrome | 100 | 177 | 265 | 304 | 428 | 258 | 1 |
| 528 | - | 583399 | 582560 | - | *nirE* | *PA14_06660* | uroporphyrin-III c-methyltransferase | 106 | 253 | 261 | 340 | 538 | 308 | 0.391278558 |
| 529 | - | 584573 | 583410 | - | *nirJ* | *PA14_06670* | heme d1 biosynthesis protein NirJ | 116 | 280 | 256 | 389 | 558 | 361 | 0.723542393 |
| 530 | - | 585082 | 584567 | - | *nirH* | *PA14_06680* | hypothetical protein | 114 | 266 | 246 | 362 | 489 | 318 | 0.232924335 |
| 531 | - | 585500 | 585057 | - | *nirG* | *PA14_06690* | transcriptional regulator | 89 | 223 | 183 | 324 | 424 | 280 | 0.115616918 |
| 532 | - | 586017 | 585493 | - | *nirL* | *PA14_06700* | heme d1 biosynthesis protein NirL | 65 | 180 | 136 | 193 | 275 | 179 | 0.036442662 |
| 533 | - | 586466 | 586014 | - | *-* | *PA14_06710* | transcriptional regulator | 118 | 312 | 256 | 361 | 476 | 342 | 0.063886935 |
| 534 | - | 587653 | 586475 | - | *nirF* | *PA14_06720* | heme d1 biosynthesis protein NirF | 148 | 361 | 283 | 450 | 598 | 453 | 0.791445601 |
| 535 | - | 588009 | 587650 | - | *nirC* | *PA14_06730* | c-type cytochrome | 109 | 306 | 171 | 265 | 366 | 240 | 0.034724611 |
| 536 | - | 588320 | 588006 | - | *nirM* | *PA14_06740* | cytochrome c-551 | 196 | 810 | 447 | 565 | 890 | 535 | 3.65613E-05 |
| 537 | - | 588321 | 588369 | ? | *-* | predicted RNA | - | 283 | 1331 | 774 | 890 | 1411 | 1049 | 1.97712E-09 |
| 538 | - | 590077 | 588371 | - | *nirS* | *PA14_06750* | nitrite reductase | 286 | 1211 | 715 | 927 | 1254 | 898 | 0.434308967 |
| 539 | - | 590293 | 591075 | + | *nirQ* | *PA14_06770* | regulatory protein NirQ | 89 | 217 | 64 | 220 | 343 | 280 | 0.242362109 |
| 540 | - | 591110 | 591637 | + | *-* | *PA14_06790* | cytochrome c oxidase subunit | 22 | 65 | 11 | 81 | 134 | 112 | 0.010140343 |
| 541 | - | 591645 | 591902 | + | *-* | *PA14_06800* | hypothetical protein | 18 | 35 | 9 | 61 | 96 | 74 | 0.52741753 |
| 542 | - | 591992 | 592432 | + | *norC* | *PA14_06810* | nitric-oxide reductase subunit C | 266 | 702 | 47 | 1090 | 2368 | 1275 | 0.432571835 |
| 543 | - | 592432 | 593829 | + | *norB* | *PA14_06830* | nitric-oxide reductase subunit B | 266 | 554 | 101 | 1082 | 2050 | 1455 | 1 |
| 544 | - | 593831 | 595669 | + | *-* | *PA14_06840* | dinitrification protein NorD | 141 | 306 | 152 | 627 | 1150 | 1003 | 1 |
| 545 | - | 595870 | 595676 | - | *-* | *PA14_06860* | hypothetical protein | 510 | 764 | 472 | 1113 | 1036 | 948 | 1 |
| 546 | - | 596637 | 595954 | - | *dnr* | *PA14_06870* | transcriptional regulator Dnr | 73 | 60 | 85 | 95 | 154 | 160 | 1 |
| 547 | - | 596840 | 596963 | + | *-* | *PA14_06875* | rsmYregulatoryRNA | 135 | 141 | 221 | 90 | 54 | 48 | 1 |
| 548 | - | 597868 | 596990 | - | *-* | *PA14_06880* | LysR family transcriptional regulator | 10 | 11 | 13 | 8 | 9 | 8 | 1 |
| 549 | - | 597970 | 598671 | + | *-* | *PA14_06890* | hypothetical protein | 302 | 116 | 260 | 111 | 164 | 114 | 0.499266146 |
| 550 | - | 598668 | 598904 | + | *-* | *PA14_06900* | hypothetical protein | 118 | 52 | 93 | 43 | 61 | 51 | 0.081032313 |
| 551 | - | 598897 | 600078 | + | *-* | *PA14_06920* | class III pyridoxal phosphate-dependent aminotransferase | 104 | 42 | 84 | 37 | 57 | 41 | 0.321350893 |
| 552 | - | 600078 | 600794 | + | *-* | *PA14_06930* | glutamine amidotransferase | 67 | 35 | 60 | 37 | 37 | 37 | 0.828318293 |
| 553 | - | 601375 | 600869 | - | *-* | *PA14_06940* | hypothetical protein | 44 | 32 | 45 | 46 | 40 | 36 | 1 |
| 554 | - | 601418 | 602908 | + | *-* | *PA14_06950* | LuxR family transcriptional regulator | 44 | 42 | 42 | 42 | 39 | 38 | 1 |
| 555 | - | 603247 | 604536 | + | *-* | *PA14_06960* | hypothetical protein | 48 | 48 | 31 | 106 | 93 | 328 | 1 |
| 556 | - | 604554 | 605108 | + | *-* | *PA14_06970* | Cro/CI family transcriptional regulator | 57 | 45 | 51 | 82 | 65 | 152 | 1 |
| 557 | - | 606244 | 605219 | - | *-* | *PA14_06980* | hypothetical protein | 122 | 119 | 126 | 107 | 119 | 85 | 1 |
| 558 | - | 606856 | 606248 | - | *-* | *PA14_06990* | hypothetical protein | 95 | 89 | 89 | 58 | 78 | 53 | 1 |
| 559 | - | 607453 | 606944 | - | *dsbB* | *PA14_07000* | disulfide bond formation protein | 24 | 20 | 22 | 14 | 11 | 10 | 1 |
| 560 | - | 607682 | 608512 | + | *-* | *PA14_07010* | hypothetical protein | 12 | 10 | 12 | 14 | 15 | 9 | 1 |
| 561 | - | 608582 | 608968 | + | *-* | *PA14_07020* | hypothetical protein | 25 | 32 | 30 | 30 | 26 | 28 | 1 |
| 562 | - | 609082 | 609540 | + | *-* | *PA14_07030* | cytochrome c' | 153 | 189 | 176 | 206 | 170 | 134 | 1 |
| 563 | - | 610151 | 609732 | - | *-* | *PA14_07040* | hypothetical protein | 104 | 120 | 134 | 106 | 104 | 93 | 1 |
| 564 | - | 610401 | 611369 | + | *-* | *PA14_07050* | hypothetical protein | 75 | 71 | 88 | 95 | 81 | 91 | 1 |
| 565 | - | 612116 | 611373 | - | *-* | *PA14_07060* | hypothetical protein | 35 | 42 | 52 | 55 | 42 | 53 | 1 |
| 566 | - | 612321 | 613625 | + | *-* | *PA14_07070* | hypothetical protein | 393 | 272 | 491 | 212 | 171 | 170 | 1 |
| 567 | - | 614872 | 613682 | - | *metK* | *PA14_07090* | S-adenosylmethionine synthetase | 401 | 159 | 147 | 115 | 130 | 280 | 0.981513735 |
| 568 | - | 615889 | 614888 | - | *-* | *PA14_07110* | ArsR family transcriptional regulator | 435 | 114 | 249 | 154 | 117 | 340 | 0.05276977 |
| 569 | - | 616135 | 618132 | + | *tktA* | *PA14_07130* | transketolase | 191 | 192 | 183 | 167 | 174 | 165 | 1 |
| 570 | - | 618132 | 619196 | + | *-* | *PA14_07140* | hypothetical protein | 120 | 112 | 118 | 109 | 119 | 108 | 1 |
| 571 | - | 619974 | 619177 | - | *-* | *PA14_07150* | hypothetical protein | 44 | 40 | 51 | 45 | 46 | 43 | 1 |
| 572 | - | 620189 | 621250 | + | *epd* | *PA14_07170* | D-erythrose 4-phosphate dehydrogenase | 37 | 29 | 37 | 29 | 27 | 30 | 1 |
| 573 | - | 621256 | 622419 | + | *pgk* | *PA14_07190* | phosphoglycerate kinase | 225 | 234 | 210 | 202 | 250 | 173 | 1 |
| 574 | - | 622492 | 622692 | + | *-* | *PA14_07200* | hypothetical protein | 477 | 516 | 543 | 667 | 508 | 549 | 1 |
| 575 | - | 622852 | 623193 | + | *-* | *PA14_07210* | hypothetical protein | 150 | 141 | 178 | 174 | 191 | 182 | 1 |
| 576 | - | 623313 | 624377 | + | *fda* | *PA14_07230* | fructose-1,6-bisphosphate aldolase | 192 | 248 | 188 | 187 | 250 | 190 | 1 |
| 577 | - | 624443 | 624925 | + | *-* | *PA14_07240* | hypothetical protein | 41 | 57 | 46 | 45 | 49 | 45 | 1 |
| 578 | - | 625582 | 626349 | + | *-* | *PA14_07260* | hypothetical protein | 74 | 74 | 75 | 82 | 73 | 61 | 1 |
| 579 | - | 625583 | 624927 | - | *-* | *PA14_07250* | hypothetical protein | 15 | 27 | 21 | 26 | 25 | 25 | 0.723249369 |
| 580 | - | 627524 | 626346 | - | *-* | *PA14_07280* | hypothetical protein | 54 | 52 | 57 | 59 | 55 | 52 | 1 |
| 581 | - | 627637 | 628125 | + | *-* | *PA14_07290* | hypothetical protein | 35 | 27 | 43 | 29 | 27 | 33 | 1 |
| 582 | - | 628151 | 629305 | + | *-* | *PA14_07300* | hypothetical protein | 26 | 21 | 23 | 19 | 21 | 21 | 1 |
| 583 | - | 629981 | 629307 | - | *-* | *PA14_07310* | hydrolase | 28 | 19 | 25 | 23 | 24 | 17 | 1 |
| 584 | - | 630463 | 630110 | - | *-* | *PA14_07330* | hypothetical protein | 27 | 25 | 20 | 19 | 23 | 18 | 1 |
| 585 | - | 631552 | 630641 | - | *-* | *PA14_07340* | transcriptional regulator | 17 | 15 | 14 | 12 | 12 | 12 | 1 |
| 586 | - | 631670 | 632008 | + | *-* | *PA14_07355* | hypothetical protein | 69 | 89 | 68 | 79 | 75 | 83 | 1 |
| 587 | - | 632513 | 631998 | - | *-* | *PA14_07360* | hypothetical protein | 63 | 79 | 69 | 74 | 69 | 66 | 1 |
| 588 | - | 632701 | 632859 | + | *-* | *PA14_07370* | hypothetical protein | 179 | 226 | 176 | 191 | 214 | 185 | 1 |

|  | A | B | C | D | E | F | G | H | I | J | K | L | M | N |
| --- | --- | --- | --- | --- | --- | --- | --- | --- | --- | --- | --- | --- | --- | --- |
| 589 | - | 633327 | 632869 | - | *-* | *PA14_07380* | hypothetical protein | 77 | 95 | 69 | 91 | 87 | 79 | 1 |
| 590 | - | 633813 | 633343 | - | *-* | *PA14_07400* | hypothetical protein | 39 | 37 | 31 | 32 | 28 | 30 | 1 |
| 591 | - | 634174 | 633827 | - | *-* | *PA14_07410* | hypothetical protein | 15 | 13 | 13 | 15 | 15 | 11 | 1 |
| 592 | - | 634778 | 634164 | - | *-* | *PA14_07420* | hypothetical protein | 36 | 34 | 33 | 33 | 33 | 31 | 1 |
| 593 | - | 637740 | 634969 | - | *-* | *PA14_07430* | hypothetical protein | 106 | 199 | 97 | 144 | 158 | 141 | 1 |
| 594 | - | 638644 | 638309 | - | *-* | *PA14_07440* | hypothetical protein | 19 | 23 | 20 | 24 | 18 | 22 | 1 |
| 595 | - | 639858 | 638737 | - | *-* | *PA14_07450* | hypothetical protein | 21 | 22 | 20 | 22 | 22 | 18 | 1 |
| 596 | - | 640278 | 640117 | - | *-* | *PA14_07460* | hypothetical protein | 24 | 30 | 30 | 24 | 23 | 25 | 1 |
| 597 | - | 640483 | 640410 | - | *-* | *PA14_07470* | Met tRNA | 57 | 49 | 49 | 30 | 26 | 40 | 1 |
| 598 | - | 642266 | 640581 | - | *-* | *PA14_07480* | reverse transcriptase | 383 | 258 | 176 | 164 | 220 | 258 | 1 |
| 599 | - | 647084 | 643347 | - | *-* | *PA14_07500* | hypothetical protein | 32 | 33 | 35 | 45 | 39 | 52 | 1 |
| 600 | - | 649044 | 647191 | - | *rpoD* | *PA14_07520* | RNA polymerase sigma factor RpoD | 666 | 623 | 774 | 518 | 589 | 559 | 1 |
| 601 | - | 651118 | 649124 | - | *dnaG* | *PA14_07530* | DNA primase | 80 | 76 | 85 | 79 | 83 | 65 | 1 |
| 602 | - | 651650 | 651201 | - | *-* | *PA14_07550* | hypothetical protein | 31 | 27 | 37 | 24 | 19 | 15 | 1 |
| 603 | - | 651935 | 651720 | - | *rpsU* | *PA14_07560* | 30S ribosomal protein S21 | 80 | 78 | 86 | 64 | 44 | 36 | 1 |
| 604 | - | 652136 | 653161 | + | *gcp* | *PA14_07570* | DNA-binding/iron metalloprotein/AP endonuclease | 82 | 68 | 91 | 80 | 65 | 63 | 1 |
| 605 | - | 653809 | 653240 | - | *-* | *PA14_07580* | glycerol-3-phosphate acyltransferase PlsY | 64 | 58 | 73 | 65 | 62 | 53 | 1 |
| 606 | - | 653893 | 654246 | + | *folB* | *PA14_07590* | dihydroneopterin aldolase | 60 | 49 | 67 | 62 | 48 | 56 | 1 |
| 607 | - | 654237 | 654779 | + | *-* | *PA14_07600* | 2-amino-4-hydroxy-6-hydroxymethyldihydropteridine pyrophosphokinase | 30 | 27 | 34 | 27 | 28 | 28 | 1 |
| 608 | - | 655984 | 654752 | - | *cca* | *PA14_07620* | multifunctional tRNA nucleotidyl transferase/2'3'-cyclic phosphodiesterase/2'nucleotidase/phosphatase | 24 | 24 | 23 | 22 | 26 | 22 | 1 |
| 609 | - | 656534 | 656028 | - | *-* | *PA14_07630* | hypothetical protein | 11 | 14 | 14 | 18 | 16 | 16 | 1 |
| 610 | - | 658182 | 656629 | - | *-* | *PA14_07650* | SpoVR family protein | 241 | 286 | 243 | 316 | 283 | 405 | 1 |
| 611 | - | 659450 | 658179 | - | *-* | *PA14_07660* | hypothetical protein | 297 | 332 | 295 | 352 | 339 | 445 | 1 |
| 612 | - | 661473 | 659551 | - | *-* | *PA14_07680* | hypothetical protein | 588 | 610 | 591 | 611 | 618 | 747 | 1 |
| 613 | - | 662084 | 661752 | - | *glpE* | *PA14_07690* | thiosulfate sulfurtransferase | 55 | 58 | 52 | 57 | 57 | 65 | 1 |
| 614 | - | 662979 | 662128 | - | *apaH* | *PA14_07700* | diadenosine tetraphosphatase | 86 | 89 | 85 | 68 | 86 | 82 | 1 |
| 615 | - | 663359 | 662979 | - | *apaG* | *PA14_07710* | ApaG protein | 137 | 138 | 133 | 120 | 135 | 143 | 1 |
| 616 | - | 664202 | 663396 | - | *ksgA* | *PA14_07730* | dimethyladenosine transferase | 35 | 29 | 38 | 34 | 36 | 29 | 1 |
| 617 | - | 665304 | 664318 | - | *pdxA* | *PA14_07740* | 4-hydroxythreonine-4-phosphate dehydrogenase | 77 | 90 | 75 | 87 | 91 | 81 | 1 |
| 618 | - | 666593 | 665301 | - | *surA* | *PA14_07760* | peptidyl-prolyl cis-trans isomerase SurA | 267 | 299 | 242 | 273 | 301 | 236 | 1 |
| 619 | - | 669348 | 666574 | - | *ostA* | *PA14_07770* | organic solvent tolerance protein OstA | 253 | 270 | 250 | 262 | 273 | 227 | 1 |
| 620 | - | 669475 | 670491 | + | *-* | *PA14_07780* | hypothetical protein | 179 | 156 | 186 | 152 | 187 | 196 | 1 |
| 621 | - | 670488 | 671162 | + | *-* | *PA14_07790* | nucleotidyl transferase | 190 | 198 | 216 | 188 | 205 | 213 | 1 |
| 622 | - | 671164 | 671922 | + | *-* | *PA14_07800* | hypothetical protein | 192 | 188 | 249 | 229 | 239 | 245 | 1 |
| 623 | - | 672984 | 671923 | - | *-* | *PA14_07810* | hypothetical protein | 50 | 53 | 55 | 65 | 49 | 52 | 1 |
| 624 | - | 673136 | 675529 | + | *-* | *PA14_07820* | histidine kinase | 56 | 50 | 56 | 58 | 50 | 46 | 1 |
| 625 | - | 675575 | 676207 | + | *-* | *PA14_07840* | two-component response regulator | 116 | 99 | 108 | 113 | 99 | 96 | 1 |
| 626 | - | 676336 | 677370 | + | *-* | *PA14_07850* | ABC transporter substrate-binding protein | 41 | 73 | 44 | 50 | 53 | 38 | 0.718815586 |
| 627 | - | 677604 | 678713 | + | *-* | *PA14_07860* | ABC transporter ATP-binding protein | 26 | 44 | 29 | 28 | 26 | 22 | 0.76759159 |
| 628 | - | 678769 | 679815 | + | *-* | *PA14_07870* | ABC transporter substrate-binding protein | 61 | 79 | 61 | 57 | 45 | 47 | 1 |
| 629 | - | 679930 | 681177 | + | *-* | *PA14_07890* | ABC transporter permease | 20 | 25 | 20 | 19 | 18 | 18 | 1 |
| 630 | - | 681283 | 682113 | + | *-* | *PA14_07900* | ABC transporter permease | 13 | 15 | 12 | 12 | 11 | 9 | 1 |
| 631 | - | 682237 | 682911 | + | *rpe* | *PA14_07910* | ribulose-phosphate 3-epimerase | 42 | 30 | 36 | 31 | 32 | 26 | 1 |
| 632 | - | 682911 | 683729 | + | *-* | *PA14_07930* | phosphoglycolate phosphatase | 40 | 37 | 40 | 37 | 39 | 31 | 1 |
| 633 | - | 683802 | 685280 | + | *trpE* | *PA14_07940* | anthranilate synthase component I | 108 | 83 | 99 | 98 | 92 | 97 | 1 |
| 634 | - | 685781 | 685467 | - | *prtN* | *PA14_07950* | transcriptional regulator PrtN | 124 | 186 | 138 | 202 | 186 | 166 | 1 |
| 635 | - | 686651 | 685881 | - | *prtR* | *PA14_07960* | transcriptional regulator PrtR | 60 | 67 | 58 | 59 | 53 | 52 | 1 |
| 636 | - | 687109 | 687309 | + | *-* | *PA14_07970* | hypothetical protein | 86 | 152 | 89 | 130 | 123 | 87 | 0.580848337 |
| 637 | - | 687357 | 687716 | + | *-* | *PA14_07980* | hypothetical protein | 69 | 136 | 76 | 114 | 125 | 75 | 0.333226212 |
| 638 | - | 688080 | 688529 | + | *-* | *PA14_07990* | hypothetical protein | 139 | 217 | 141 | 286 | 331 | 157 | 1 |
| 639 | - | 688551 | 689066 | + | *-* | *PA14_08000* | hypothetical protein | 284 | 402 | 309 | 506 | 556 | 320 | 1 |
| 640 | - | 689063 | 689620 | + | *-* | *PA14_08010* | hypothetical protein | 190 | 379 | 232 | 428 | 501 | 241 | 0.986282904 |
| 641 | - | 689773 | 690099 | + | *-* | *PA14_08020* | bacteriophage protein | 174 | 442 | 245 | 486 | 543 | 246 | 0.10997121 |
| 642 | - | 690096 | 690983 | + | *-* | *PA14_08030* | phage baseplate assembly protein | 309 | 592 | 336 | 765 | 904 | 415 | 1 |
| 643 | - | 690976 | 691509 | + | *-* | *PA14_08040* | phage tail protein | 371 | 672 | 390 | 825 | 988 | 469 | 1 |
| 644 | - | 691511 | 693586 | + | *-* | *PA14_08050* | tail fiber protein | 393 | 749 | 468 | 904 | 1049 | 535 | 1 |
| 645 | - | 693583 | 694041 | + | *-* | *PA14_08060* | tail fiber assembly protein | 200 | 533 | 329 | 645 | 746 | 348 | 0.221092106 |
| 646 | - | 694084 | 695244 | + | *-* | *PA14_08070* | phage tail sheath protein | 665 | 1247 | 798 | 1180 | 1409 | 814 | 1 |
| 647 | - | 695257 | 695760 | + | *-* | *PA14_08090* | phage tail tube protein | 647 | 1219 | 767 | 1157 | 1369 | 784 | 1 |
| 648 | - | 695775 | 696119 | + | *-* | *PA14_08100* | hypothetical protein | 479 | 854 | 514 | 827 | 976 | 533 | 1 |
| 649 | - | 696088 | 696198 | + | *-* | *PA14_08110* | hypothetical protein | 337 | 648 | 372 | 723 | 851 | 392 | 0.549291923 |
| 650 | - | 696279 | 696288 | ? | *-* | predicted RNA | - | 335 | 696 | 386 | 734 | 858 | 393 | 0.214768135 |
| 651 | - | 696289 | 698526 | + | *-* | *PA14_08120* | tail length determinator protein | 384 | 702 | 456 | 782 | 937 | 442 | 1 |
| 652 | - | 698536 | 699408 | + | *-* | *PA14_08130* | hypothetical protein | 329 | 558 | 351 | 622 | 757 | 347 | 1 |
| 653 | - | 699383 | 699589 | + | *-* | *PA14_08140* | hypothetical protein | 246 | 486 | 294 | 524 | 644 | 272 | 0.480861202 |
| 654 | - | 699647 | 700636 | + | *-* | *PA14_08150* | phage late control gene D protein | 228 | 436 | 252 | 481 | 624 | 281 | 1 |
| 655 | - | 700669 | 701298 | + | *-* | *PA14_08160* | lytic enzyme | 193 | 305 | 207 | 350 | 425 | 214 | 1 |
| 656 | - | 701295 | 701657 | + | *-* | *PA14_08180* | hypothetical protein | 266 | 337 | 222 | 383 | 475 | 258 | 1 |
| 657 | - | 701654 | 701911 | + | *-* | *PA14_08190* | hypothetical protein | 198 | 316 | 184 | 367 | 443 | 216 | 0.934854092 |
| 658 | - | 701926 | 702213 | + | *-* | *PA14_08200* | hypothetical protein | 176 | 343 | 201 | 289 | 339 | 174 | 0.503207228 |
| 659 | - | 702227 | 702721 | + | *-* | *PA14_08210* | hypothetical protein | 480 | 863 | 516 | 757 | 869 | 553 | 1 |
| 660 | - | 702733 | 703080 | + | *-* | *PA14_08220* | hypothetical protein | 442 | 823 | 540 | 797 | 974 | 601 | 1 |
| 661 | - | 703092 | 703109 | ? | *-* | predicted RNA | - | 542 | 817 | 499 | 811 | 1008 | 607 | 1 |
| 662 | - | 703110 | 703364 | + | *-* | *PA14_08230* | hypothetical protein | 600 | 938 | 570 | 968 | 1180 | 683 | 1 |
| 663 | - | 703411 | 705246 | + | *-* | *PA14_08240* | hypothetical protein | 257 | 519 | 310 | 581 | 700 | 342 | 1 |
| 664 | - | 705239 | 705580 | + | *-* | *PA14_08250* | hypothetical protein | 352 | 667 | 398 | 897 | 1072 | 499 | 1 |
| 665 | - | 705588 | 706283 | + | *-* | *PA14_08260* | minor tail protein L | 208 | 341 | 217 | 369 | 442 | 236 | 1 |
| 666 | - | 706286 | 707056 | + | *-* | *PA14_08270* | hypothetical protein | 252 | 392 | 257 | 451 | 555 | 286 | 1 |
| 667 | - | 707111 | 707713 | + | *-* | *PA14_08280* | bacteriophage protein | 211 | 384 | 231 | 461 | 560 | 264 | 1 |
| 668 | - | 707772 | 711431 | + | *-* | *PA14_08300* | phage-related protein, tail component | 208 | 343 | 211 | 370 | 469 | 252 | 1 |
| 669 | - | 712461 | 713513 | + | *-* | *PA14_08310* | hypothetical protein | 162 | 312 | 191 | 274 | 338 | 221 | 1 |
| 670 | - | 713513 | 713815 | + | *-* | *PA14_08320* | hypothetical protein | 221 | 348 | 249 | 342 | 435 | 314 | 1 |
| 671 | - | 713812 | 714042 | + | *-* | *PA14_08330* | hypothetical protein | 115 | 212 | 164 | 179 | 234 | 148 | 0.499266146 |
| 672 | - | 714461 | 715066 | + | *trpG* | *PA14_08340* | anthranilate synthase component II | 132 | 117 | 119 | 147 | 141 | 109 | 1 |

|  | A | B | C | D | E | F | G | H | I | J | K | L | M | N |
| --- | --- | --- | --- | --- | --- | --- | --- | --- | --- | --- | --- | --- | --- | --- |
| 673 | - | 715068 | 716117 | + | *trpD* | *PA14_08350* | anthranilate phosphoribosyltransferase | 154 | 147 | 159 | 166 | 149 | 156 | 1 |
| 674 | - | 716114 | 716950 | + | *trpC* | *PA14_08360* | indole-3-glycerol-phosphate synthase | 107 | 109 | 83 | 99 | 110 | 92 | 1 |
| 675 | - | 717656 | 717012 | - | *vfr* | *PA14_08370* | cAMP-regulatory protein | 163 | 202 | 144 | 155 | 186 | 129 | 1 |
| 676 | - | 717928 | 718350 | + | *-* | *PA14_08380* | hypothetical protein | 96 | 85 | 101 | 103 | 100 | 88 | 1 |
| 677 | - | 718670 | 719464 | + | *speD* | *PA14_08390* | S-adenosylmethionine decarboxylase | 32 | 33 | 30 | 33 | 35 | 28 | 1 |
| 678 | - | 720166 | 719519 | - | *-* | *PA14_08400* | hypothetical protein | 114 | 108 | 125 | 97 | 127 | 120 | 1 |
| 679 | - | 720604 | 720266 | - | *-* | *PA14_08420* | HIT family protein | 139 | 146 | 175 | 192 | 181 | 224 | 1 |
| 680 | - | 720683 | 722164 | + | *-* | *PA14_08430* | ATPase | 101 | 116 | 109 | 120 | 108 | 103 | 1 |
| 681 | - | 723004 | 722204 | - | *-* | *PA14_08440* | short chain alcohol dehydrogenase | 45 | 50 | 42 | 43 | 51 | 37 | 1 |
| 682 | - | 724147 | 723065 | - | *-* | *PA14_08450* | hypothetical protein | 35 | 43 | 29 | 33 | 45 | 29 | 1 |
| 683 | - | 725237 | 724269 | - | *-* | *PA14_08460* | hypothetical protein | 48 | 64 | 39 | 53 | 62 | 46 | 1 |
| 684 | - | 725676 | 725254 | - | *-* | *PA14_08470* | hypothetical protein | 22 | 26 | 19 | 21 | 25 | 19 | 1 |
| 685 | - | 725967 | 727001 | + | *argC* | *PA14_08480* | N-acetyl-gamma-glutamyl-phosphate reductase | 107 | 89 | 104 | 105 | 92 | 93 | 1 |
| 686 | - | 727001 | 727720 | + | *-* | *PA14_08490* | hypothetical protein | 65 | 72 | 69 | 68 | 72 | 51 | 1 |
| 687 | - | 727721 | 728143 | + | *-* | *PA14_08500* | hypothetical protein | 128 | 139 | 152 | 127 | 134 | 120 | 1 |
| 688 | - | 728221 | 728571 | + | *-* | *PA14_08510* | iron-sulfur cluster insertion protein ErpA | 189 | 186 | 121 | 231 | 405 | 288 | 1 |
| 689 | - | 729716 | 728625 | - | *anmK* | *PA14_08520* | anhydro-N-acetylmuramic acid kinase | 86 | 99 | 87 | 100 | 98 | 82 | 1 |
| 690 | - | 731062 | 729719 | - | *-* | *PA14_08540* | hypothetical protein | 125 | 130 | 119 | 140 | 111 | 108 | 1 |
| 691 | - | 731347 | 732546 | + | *tyrZ* | *PA14_08560* | tyrosyl-tRNA synthetase | 61 | 64 | 67 | 58 | 62 | 50 | 1 |
| 692 | - | 733095 | 734620 | + | *-* | *PA14_08570* | 16S ribosomal RNA | 237 | 360 | 900 | 310 | 553 | 68 | 1 |
| 693 | - | 734686 | 734759 | + | *-* | *PA14_08580* | Ile tRNA | 710 | 1071 | 928 | 568 | 435 | 216 | 1 |
| 694 | - | 734791 | 734863 | + | *-* | *PA14_08590* | Ala tRNA | 696 | 956 | 932 | 643 | 489 | 240 | 1 |
| 695 | - | 735093 | 737983 | + | *-* | *PA14_08600* | 23S ribosomal RNA | 612 | 872 | 726 | 418 | 753 | 121 | 1 |
| 696 | - | 738120 | 738251 | + | *-* | *PA14_08610* | 5S ribosomal RNA | 11 | 11 | 27 | 11 | 5 | 2 | 1 |
| 697 | - | 738405 | 739343 | + | *birA* | *PA14_08620* | biotin--protein ligase | 50 | 51 | 54 | 56 | 51 | 42 | 1 |
| 698 | - | 739340 | 740086 | + | *-* | *PA14_08630* | pantothenate kinase | 52 | 51 | 49 | 48 | 47 | 42 | 1 |
| 699 | - | 740095 | 740799 | + | *-* | *PA14_08640* | hypothetical protein | 64 | 68 | 72 | 77 | 72 | 61 | 1 |
| 700 | - | 740947 | 741028 | + | *-* | *PA14_08650* | Tyr tRNA | 409 | 188 | 348 | 211 | 145 | 145 | 0.134543655 |
| 701 | - | 741058 | 741128 | + | *-* | *PA14_08660* | Gly tRNA | 415 | 195 | 381 | 262 | 173 | 179 | 0.14959202 |
| 702 | - | 741151 | 741223 | + | *-* | *PA14_08670* | Thr tRNA | 198 | 124 | 206 | 152 | 99 | 167 | 0.771447696 |
| 703 | - | 741311 | 742504 | + | *tufB* | *PA14_08680* | elongation factor Tu | 1616 | 2292 | 1236 | 1010 | 1620 | 947 | 1 |
| 704 | - | 742560 | 742632 | + | *-* | *PA14_08690* | Trp tRNA | 93 | 146 | 114 | 72 | 68 | 47 | 1 |
| 705 | - | 742681 | 743049 | + | *secE* | *PA14_08695* | preprotein translocase subunit SecE | 283 | 249 | 305 | 207 | 200 | 158 | 1 |
| 706 | - | 743059 | 743592 | + | *nusG* | *PA14_08710* | transcription antitermination protein NusG | 518 | 477 | 571 | 386 | 423 | 328 | 1 |
| 707 | - | 743709 | 744140 | + | *rplK* | *PA14_08720* | 50S ribosomal protein L11 | 559 | 889 | 447 | 358 | 611 | 307 | 1 |
| 708 | - | 744140 | 744835 | + | *rplA* | *PA14_08730* | 50S ribosomal protein L1 | 568 | 893 | 465 | 394 | 679 | 324 | 1 |
| 709 | - | 745022 | 745033 | ? | *-* | predicted RNA | - | 522 | 790 | 483 | 376 | 673 | 324 | 1 |
| 710 | - | 745034 | 745534 | + | *rplJ* | *PA14_08740* | 50S ribosomal protein L10 | 427 | 611 | 320 | 241 | 422 | 219 | 1 |
| 711 | - | 745613 | 745981 | + | *rplL* | *PA14_08750* | 50S ribosomal protein L7/L12 | 638 | 1049 | 557 | 402 | 597 | 300 | 1 |
| 712 | - | 746203 | 750276 | + | *rpoB* | *PA14_08760* | DNA-directed RNA polymerase subunit beta | 693 | 754 | 556 | 495 | 620 | 437 | 1 |
| 713 | - | 750342 | 754541 | + | *rpoC* | *PA14_08780* | DNA-directed RNA polymerase subunit beta' | 863 | 950 | 603 | 558 | 738 | 533 | 1 |
| 714 | 754615 | 754693 | 755064 | + | *rpsL* | *PA14_08790* | 30S ribosomal protein S12 | 507 | 858 | 453 | 370 | 395 | 271 | 1 |
| 715 | 754615 | 755164 | 755634 | + | *rpsG* | *PA14_08810* | 30S ribosomal protein S7 | 789 | 1192 | 736 | 646 | 865 | 528 | 1 |
| 716 | 754615 | 755665 | 757785 | + | *fusA1* | *PA14_08820* | elongation factor G | 1032 | 1350 | 864 | 775 | 953 | 611 | 1 |
| 717 | 754615 | 757816 | 759009 | + | *tufA* | *PA14_08830* | elongation factor Tu | 1996 | 2750 | 1478 | 1221 | 1985 | 1143 | 1 |
| 718 | - | 759165 | 759476 | + | *rpsJ* | *PA14_08840* | 30S ribosomal protein S10 | 474 | 753 | 498 | 416 | 369 | 276 | 1 |
| 719 | - | 759559 | 760194 | + | *rplC* | *PA14_08850* | 50S ribosomal protein L3 | 653 | 929 | 746 | 531 | 657 | 405 | 1 |
| 720 | - | 760208 | 760810 | + | *rplD* | *PA14_08860* | 50S ribosomal protein L4 | 626 | 925 | 621 | 487 | 653 | 366 | 1 |
| 721 | - | 760807 | 761106 | + | *rplW* | *PA14_08870* | 50S ribosomal protein L23 | 328 | 655 | 309 | 255 | 319 | 191 | 0.912927393 |
| 722 | - | 761118 | 761939 | + | *rplB* | *PA14_08880* | 50S ribosomal protein L2 | 603 | 806 | 494 | 459 | 653 | 346 | 1 |
| 723 | - | 761956 | 762231 | + | *rpsS* | *PA14_08890* | 30S ribosomal protein S19 | 534 | 833 | 433 | 393 | 564 | 302 | 1 |
| 724 | - | 762244 | 762576 | + | *rplV* | *PA14_08900* | 50S ribosomal protein L22 | 630 | 840 | 524 | 461 | 575 | 363 | 1 |
| 725 | - | 762589 | 763275 | + | *rpsC* | *PA14_08910* | 30S ribosomal protein S3 | 682 | 875 | 568 | 402 | 594 | 360 | 1 |
| 726 | - | 763287 | 763700 | + | *rplP* | *PA14_08920* | 50S ribosomal protein L16 | 615 | 804 | 471 | 317 | 557 | 302 | 1 |
| 727 | - | 763700 | 763891 | + | *rpmC* | *PA14_08930* | 50S ribosomal protein L29 | 786 | 933 | 543 | 424 | 762 | 372 | 1 |
| 728 | - | 763894 | 764160 | + | *rpsQ* | *PA14_08940* | 30S ribosomal protein S17 | 438 | 611 | 304 | 230 | 392 | 215 | 1 |
| 729 | - | 764184 | 764552 | + | *rplN* | *PA14_08950* | 50S ribosomal protein L14 | 880 | 1131 | 655 | 564 | 908 | 488 | 1 |
| 730 | - | 764565 | 764879 | + | *rplX* | *PA14_08960* | 50S ribosomal protein L24 | 525 | 677 | 351 | 274 | 391 | 233 | 1 |
| 731 | - | 764899 | 765438 | + | *rplE* | *PA14_08970* | 50S ribosomal protein L5 | 864 | 942 | 597 | 479 | 739 | 465 | 1 |
| 732 | - | 765452 | 765757 | + | *rpsN* | *PA14_08980* | 30S ribosomal protein S14 | 900 | 1116 | 665 | 390 | 638 | 394 | 1 |
| 733 | - | 765762 | 765784 | ? | *-* | predicted RNA | - | 430 | 589 | 361 | 153 | 210 | 151 | 1 |
| 734 | - | 765947 | 766339 | + | *rpsH* | *PA14_08990* | 30S ribosomal protein S8 | 377 | 402 | 352 | 288 | 291 | 219 | 1 |
| 735 | - | 766351 | 766884 | + | *rplF* | *PA14_09000* | 50S ribosomal protein L6 | 573 | 686 | 537 | 515 | 566 | 405 | 1 |
| 736 | - | 766895 | 767245 | + | *rplR* | *PA14_09010* | 50S ribosomal protein L18 | 744 | 1027 | 730 | 636 | 683 | 466 | 1 |
| 737 | - | 767249 | 767749 | + | *rpsE* | *PA14_09020* | 30S ribosomal protein S5 | 1418 | 1910 | 1362 | 1400 | 1696 | 1071 | 1 |
| 738 | - | 767752 | 767928 | + | *rpmD* | *PA14_09030* | 50S ribosomal protein L30 | 1094 | 1443 | 890 | 883 | 1004 | 664 | 1 |
| 739 | - | 767932 | 768366 | + | *rplO* | *PA14_09040* | 50S ribosomal protein L15 | 1189 | 1506 | 941 | 924 | 1102 | 694 | 1 |
| 740 | - | 768367 | 769695 | + | *secY* | *PA14_09050* | preprotein translocase subunit SecY | 946 | 1024 | 832 | 729 | 839 | 563 | 1 |
| 741 | - | 769724 | 769840 | + | *rpmJ* | *PA14_09070* | 50S ribosomal protein L36 | 1672 | 1985 | 1811 | 1465 | 1394 | 1119 | 1 |
| 742 | - | 769850 | 769902 | ? | *-* | predicted RNA | - | 1195 | 1431 | 1095 | 827 | 889 | 677 | 1 |
| 743 | - | 769971 | 770327 | + | *rpsM* | *PA14_09080* | 30S ribosomal protein S13 | 762 | 831 | 602 | 496 | 608 | 467 | 1 |
| 744 | - | 770346 | 770735 | + | *rpsK* | *PA14_09090* | 30S ribosomal protein S11 | 524 | 766 | 415 | 343 | 459 | 321 | 1 |
| 745 | - | 770752 | 771372 | + | *rpsD* | *PA14_09100* | 30S ribosomal protein S4 | 609 | 884 | 448 | 412 | 501 | 369 | 1 |
| 746 | - | 771395 | 772396 | + | *rpoA* | *PA14_09115* | DNA-directed RNA polymerase subunit alpha | 1067 | 1192 | 952 | 814 | 835 | 628 | 1 |
| 747 | - | 772440 | 772829 | + | *rplQ* | *PA14_09130* | 50S ribosomal protein L17 | 468 | 674 | 326 | 296 | 385 | 282 | 1 |
| 748 | - | 773110 | 774558 | + | *katA* | *PA14_09150* | catalase | 460 | 464 | 341 | 182 | 326 | 565 | 1 |
| 749 | - | 774689 | 775153 | + | *bfrA* | *PA14_09160* | bacterioferritin | 164 | 193 | 135 | 196 | 221 | 182 | 1 |
| 750 | - | 778062 | 775225 | - | *uvrA* | *PA14_09180* | excinuclease ABC subunit A | 70 | 87 | 77 | 69 | 81 | 61 | 1 |
| 751 | - | 778276 | 779664 | + | *-* | *PA14_09195* | major facilitator transporter | 52 | 43 | 54 | 47 | 44 | 37 | 1 |
| 752 | - | 779681 | 780178 | + | *ssb* | *PA14_09200* | single-stranded DNA-binding protein | 168 | 214 | 152 | 151 | 175 | 135 | 1 |
| 753 | - | 781697 | 780267 | - | *pchA* | *PA14_09210* | salicylate biosynthesis isochorismate synthase | 86 | 227 | 85 | 251 | 249 | 207 | 0.460197005 |
| 754 | - | 781999 | 781694 | - | *pchB* | *PA14_09220* | isochorismate-pyruvate lyase | 74 | 255 | 61 | 287 | 254 | 217 | 0.000294392 |
| 755 | - | 782754 | 781999 | - | *pchC* | *PA14_09230* | pyochelin biosynthetic protein PchC | 35 | 97 | 33 | 176 | 134 | 96 | 0.01946313 |
| 756 | - | 784394 | 782751 | - | *pchD* | *PA14_09240* | pyochelin biosynthesis protein PchD | 40 | 132 | 42 | 294 | 194 | 118 | 0.007836446 |

|  | A | B | C | D | E | F | G | H | I | J | K | L | M | N |
| --- | --- | --- | --- | --- | --- | --- | --- | --- | --- | --- | --- | --- | --- | --- |
| 757 | - | 784624 | 785514 | + | *pchR* | *PA14_09260* | transcriptional regulator PchR | 19 | 54 | 23 | 62 | 50 | 33 | 0.007773202 |
| 758 | - | 785708 | 790024 | + | *pchE* | *PA14_09270* | dihydroaeruginoic acid synthetase | 93 | 238 | 99 | 295 | 291 | 193 | 1 |
| 759 | - | 790021 | 795450 | + | *pchF* | *PA14_09280* | pyochelin synthetase | 119 | 233 | 111 | 192 | 242 | 210 | 1 |
| 760 | - | 795447 | 796496 | + | *pchG* | *PA14_09290* | pyochelin biosynthetic protein PchG | 153 | 255 | 120 | 203 | 253 | 257 | 1 |
| 761 | - | 796493 | 798205 | + | *-* | *PA14_09300* | ABC transporter ATP-binding protein | 97 | 147 | 72 | 117 | 159 | 145 | 1 |
| 762 | - | 798202 | 799926 | + | *-* | *PA14_09320* | ABC transporter ATP-binding protein | 173 | 223 | 129 | 171 | 239 | 216 | 1 |
| 763 | - | 800018 | 802180 | + | *fptA* | *PA14_09340* | Fe(III)-pyochelin outer membrane receptor | 101 | 329 | 102 | 665 | 468 | 190 | 0.401171332 |
| 764 | - | 802180 | 802461 | + | *-* | *PA14_09350* | hypothetical protein | 153 | 314 | 162 | 561 | 477 | 256 | 0.395977757 |
| 765 | - | 802793 | 803968 | + | *-* | *PA14_09370* | hypothetical protein | 59 | 92 | 45 | 145 | 154 | 78 | 1 |
| 766 | - | 803961 | 805205 | + | *-* | *PA14_09380* | transporter | 110 | 137 | 56 | 137 | 170 | 123 | 1 |
| 767 | - | 806518 | 805310 | - | *phzS* | *PA14_09400* | hypothetical protein | 943 | 51 | 51 | 63 | 97 | 678 | 5.8118E-125 |
| 768 | 813529 | 807503 | 806856 | - | *phzG1* | *PA14_09410* | pyrodoxamine 5'-phosphate oxidase | 907 | 10 | 30 | 58 | 70 | 705 | 0 |
| 769 | 813529 | 808362 | 807526 | - | *phzF1* | *PA14_09420* | phenazine biosynthesis protein | 899 | 10 | 27 | 57 | 72 | 708 | 0 |
| 770 | 813529 | 810259 | 808376 | - | *phzE1* | *PA14_09440* | phenazine biosynthesis protein PhzE | 1250 | 15 | 44 | 94 | 98 | 969 | 0 |
| 771 | 813529 | 810879 | 810256 | - | *phzD1* | *PA14_09450* | phenazine biosynthesis protein PhzD | 968 | 6 | 21 | 69 | 82 | 748 | 0 |
| 772 | 812427 | 812093 | 810876 | - | *phzC1* | *PA14_09460* | phenazine biosynthesis protein PhzC | 1244 | 8 | 39 | 59 | 72 | 832 | 0 |
| 773 | 812427 | 812617 | 812129 | - | *phzB1* | *PA14_09470* | phenazine biosynthesis protein | 528 | 1 | 11 | 17 | 43 | 415 | 0 |
| 774 | 813529 | 813135 | 812647 | - | *phzA1* | *PA14_09480* | phenazine biosynthesis protein | 123 | 0 | 7 | 7 | 6 | 95 | 0 |
| 775 | 813529 | 813831 | 814835 | + | *phzM* | *PA14_09490* | phenazine-specific methyltransferase | 284 | 14 | 40 | 33 | 33 | 358 | 2.1957E-299 |
| 776 | - | 816309 | 814846 | - | *opmD* | *PA14_09500* | outer membrane protein | 488 | 22 | 35 | 30 | 31 | 605 | 8.6398E-244 |
| 777 | - | 819395 | 816306 | - | *mexI* | *PA14_09520* | RND efflux transporter | 340 | 17 | 16 | 17 | 17 | 366 | 3.1109E-142 |
| 778 | - | 820520 | 819408 | - | *mexH* | *PA14_09530* | RND efflux membrane fusion protein | 286 | 14 | 11 | 12 | 11 | 301 | 2.0372E-284 |
| 779 | - | 820974 | 820528 | - | *mexG* | *PA14_09540* | hypothetical protein | 330 | 22 | 15 | 18 | 16 | 315 | 1.4046E-178 |
| 780 | - | 822791 | 821625 | - | *-* | *PA14_09550* | hypothetical protein | 48 | 39 | 39 | 37 | 48 | 42 | 1 |
| 781 | - | 823725 | 822871 | - | *-* | *PA14_09570* | LysR family transcriptional regulator | 25 | 22 | 20 | 21 | 22 | 28 | 1 |
| 782 | - | 823833 | 824888 | + | *-* | *PA14_09580* | hypothetical protein | 32 | 38 | 26 | 23 | 35 | 33 | 1 |
| 783 | - | 824984 | 826024 | + | *ddlA* | *PA14_09600* | D-alanine--D-alanine ligase | 190 | 149 | 147 | 130 | 138 | 189 | 1 |
| 784 | - | 826930 | 826064 | - | *-* | *PA14_09610* | hypothetical protein | 65 | 58 | 57 | 32 | 54 | 52 | 1 |
| 785 | - | 828919 | 827138 | - | *-* | *PA14_09630* | acyl-CoA dehydrogenase | 154 | 118 | 120 | 59 | 88 | 96 | 1 |
| 786 | - | 830659 | 829037 | - | *-* | *PA14_09660* | acyl-CoA synthetase | 105 | 97 | 92 | 50 | 78 | 65 | 1 |
| 787 | - | 830905 | 833181 | + | *-* | *PA14_09680* | two-component sensor | 71 | 70 | 62 | 41 | 69 | 58 | 1 |
| 788 | - | 833184 | 833828 | + | *-* | *PA14_09690* | two-component response regulator | 109 | 109 | 100 | 81 | 117 | 121 | 1 |
| 789 | - | 835038 | 833833 | - | *-* | *PA14_09700* | monooxygenase | 57 | 73 | 52 | 46 | 68 | 58 | 1 |
| 790 | - | 836696 | 835206 | - | *-* | *PA14_09710* | aldehyde dehydrogenase | 7 | 6 | 5 | 5 | 6 | 6 | 1 |
| 791 | - | 837600 | 836689 | - | *-* | *PA14_09730* | dihydrodipicolinate synthase | 6 | 6 | 5 | 3 | 5 | 5 | 1 |
| 792 | - | 838993 | 837665 | - | *-* | *PA14_09740* | MFS transporter | 7 | 7 | 7 | 7 | 6 | 7 | 1 |
| 793 | - | 840448 | 839129 | - | *-* | *PA14_09750* | oxidoreductase | 70 | 64 | 85 | 74 | 60 | 62 | 1 |
| 794 | - | 841270 | 840542 | - | *-* | *PA14_09760* | GntR family transcriptional regulator | 22 | 20 | 22 | 19 | 17 | 18 | 1 |
| 795 | - | 841579 | 842601 | + | *-* | *PA14_09770* | AraC family transcriptional regulator | 37 | 37 | 33 | 37 | 41 | 38 | 1 |
| 796 | - | 842627 | 843094 | + | *-* | *PA14_09780* | hypothetical protein | 36 | 40 | 39 | 29 | 36 | 37 | 1 |
| 797 | - | 843736 | 843098 | - | *-* | *PA14_09790* | transcriptional regulator | 54 | 72 | 53 | 52 | 58 | 52 | 1 |
| 798 | - | 844483 | 843764 | - | *-* | *PA14_09810* | hypothetical protein | 26 | 22 | 23 | 22 | 20 | 18 | 1 |
| 799 | - | 844784 | 846427 | + | *-* | *PA14_09820* | acetolactate synthase | 84 | 81 | 68 | 73 | 84 | 76 | 1 |
| 800 | - | 847765 | 846470 | - | *-* | *PA14_09850* | porin | 40 | 31 | 31 | 29 | 32 | 36 | 1 |
| 801 | - | 848746 | 848003 | - | *-* | *PA14_09870* | hypothetical protein | 5 | 5 | 6 | 5 | 3 | 4 | 1 |
| 802 | - | 849247 | 848846 | - | *-* | *PA14_09880* | hypothetical protein | 4 | 4 | 4 | 5 | 5 | 4 | 1 |
| 803 | - | 849390 | 849671 | + | *ppiC2* | *PA14_09890* | peptidyl-prolyl cis-trans isomerase C2 | 230 | 336 | 233 | 214 | 268 | 196 | 1 |
| 804 | - | 851380 | 849992 | - | *prpL* | *PA14_09900* | Pvds-regulated endoprotease, lysyl class | 93 | 141 | 93 | 141 | 125 | 148 | 1 |
| 805 | - | 852836 | 851913 | - | *-* | *PA14_09910* | transcriptional regulator | 36 | 55 | 44 | 63 | 51 | 57 | 1 |
| 806 | - | 852977 | 853375 | + | *-* | *PA14_09920* | translation initiation inhibitor | 4 | 5 | 2 | 3 | 3 | 3 | 1 |
| 807 | - | 854207 | 853407 | - | *-* | *PA14_09930* | exonuclease III | 9 | 12 | 11 | 12 | 12 | 9 | 1 |
| 808 | - | 854793 | 854230 | - | *-* | *PA14_09940* | protease | 19 | 32 | 32 | 28 | 27 | 22 | 1 |
| 809 | - | 855861 | 854929 | - | *-* | *PA14_09950* | oxidoreductase | 17 | 13 | 15 | 20 | 18 | 16 | 1 |
| 810 | - | 855942 | 856370 | + | *-* | *PA14_09960* | hypothetical protein | 12 | 13 | 14 | 13 | 12 | 9 | 1 |
| 811 | - | 858843 | 856435 | - | *fpvB* | *PA14_09970* | type I ferripyoverdine receptor, FpvB | 27 | 28 | 28 | 40 | 26 | 29 | 1 |
| 812 | - | 859186 | 860004 | + | *dkgB* | *PA14_09980* | 2,5-diketo-D-gluconate reductase B | 3 | 3 | 2 | 6 | 3 | 2 | 1 |
| 813 | - | 860512 | 860054 | - | *-* | *PA14_09990* | acetyltransferase | 3 | 4 | 3 | 4 | 4 | 6 | 1 |
| 814 | - | 860644 | 862134 | + | *-* | *PA14_10010* | transcriptional regulator | 28 | 27 | 22 | 23 | 26 | 24 | 1 |
| 815 | - | 862476 | 862150 | - | *-* | *PA14_10020* | hypothetical protein | 61 | 76 | 53 | 52 | 74 | 66 | 1 |
| 816 | - | 864247 | 862538 | - | *-* | *PA14_10040* | amidase | 66 | 86 | 61 | 54 | 75 | 56 | 1 |
| 817 | - | 864931 | 864434 | - | *-* | *PA14_10050* | hypothetical protein | 3 | 4 | 3 | 4 | 4 | 4 | 1 |
| 818 | - | 865931 | 864945 | - | *-* | *PA14_10070* | zinc-dependent oxidoreductase | 6 | 5 | 4 | 5 | 6 | 6 | 1 |
| 819 | - | 866321 | 865944 | - | *-* | *PA14_10080* | hypothetical protein | 24 | 23 | 22 | 33 | 21 | 26 | 1 |
| 820 | - | 866440 | 867351 | + | *-* | *PA14_10090* | LysR family transcriptional regulator | 46 | 40 | 47 | 41 | 44 | 45 | 1 |
| 821 | - | 868546 | 867362 | - | *-* | *PA14_10110* | transporter | 10 | 10 | 10 | 11 | 8 | 12 | 1 |
| 822 | - | 869571 | 868642 | - | *-* | *PA14_10120* | LysR family transcriptional regulator | 24 | 21 | 24 | 21 | 18 | 19 | 1 |
| 823 | - | 869711 | 870427 | + | *-* | *PA14_10130* | short chain dehydrogenase | 33 | 29 | 31 | 33 | 31 | 30 | 1 |
| 824 | - | 871468 | 870437 | - | *fepG* | *PA14_10140* | ferric enterobactin transport protein FepG | 16 | 13 | 13 | 19 | 14 | 11 | 1 |
| 825 | - | 872487 | 871465 | - | *fepD* | *PA14_10160* | ferric enterobactin transport protein FepD | 2 | 4 | 2 | 11 | 6 | 1 | 0.829483661 |
| 826 | - | 873423 | 872518 | - | *fepB* | *PA14_10170* | iron-enterobactin transporter periplasmic binding protein | 5 | 12 | 6 | 49 | 20 | 5 | 0.059785741 |
| 827 | - | 873549 | 874346 | + | *fepC* | *PA14_10180* | ferric enterobactin transport protein FepC | 5 | 10 | 4 | 14 | 12 | 7 | 0.760328835 |
| 828 | - | 875141 | 874353 | - | *-* | *PA14_10190* | transcriptional regulator | 23 | 26 | 22 | 25 | 23 | 20 | 1 |
| 829 | - | 875397 | 877481 | + | *-* | *PA14_10200* | TonB-dependent receptor protein | 7 | 11 | 7 | 19 | 11 | 8 | 1 |
| 830 | - | 877549 | 878856 | + | *-* | *PA14_10210* | oxidoreductase | 3 | 5 | 3 | 6 | 5 | 4 | 1 |
| 831 | - | 879293 | 879961 | + | *-* | *PA14_10220* | SH3 domain-containing protein | 9 | 7 | 13 | 7 | 9 | 5 | 1 |
| 832 | - | 881136 | 880045 | - | *adh* | *PA14_10230* | 2,3-butanediol dehydrogenase | 5 | 6 | 6 | 5 | 6 | 5 | 1 |
| 833 | - | 882262 | 881150 | - | *-* | *PA14_10240* | branched-chain alpha-keto acid dehydrogenase subunit E2 | 10 | 9 | 8 | 11 | 9 | 11 | 1 |
| 834 | - | 883278 | 882259 | - | *acoB* | *PA14_10250* | acetoin catabolism protein AcoB | 11 | 12 | 11 | 14 | 11 | 10 | 1 |
| 835 | - | 884285 | 883311 | - | *-* | *PA14_10260* | dehydrogenase E1 component | 27 | 25 | 29 | 43 | 30 | 32 | 1 |
| 836 | - | 885348 | 884308 | - | *-* | *PA14_10270* | hypothetical protein | 12 | 12 | 12 | 11 | 12 | 10 | 1 |
| 837 | - | 886145 | 885345 | - | *-* | *PA14_10280* | short-chain dehydrogenase | 8 | 9 | 9 | 7 | 8 | 10 | 1 |
| 838 | - | 886477 | 888354 | + | *acoR* | *PA14_10290* | transcriptional regulator AcoR | 68 | 79 | 81 | 82 | 73 | 96 | 1 |
| 839 | 895433 | 888559 | 889170 | + | *-* | *PA14_10300* | efflux protein | 15 | 12 | 12 | 13 | 12 | 17 | 1 |
| 840 | 895433 | 890047 | 889157 | - | *-* | *PA14_10320* | transcriptional regulator | 33 | 27 | 23 | 24 | 23 | 28 | 1 |

|  | A | B | C | D | E | F | G | H | I | J | K | L | M | N |
| --- | --- | --- | --- | --- | --- | --- | --- | --- | --- | --- | --- | --- | --- | --- |
| 841 | 895433 | 891558 | 890143 | - | *-* | *PA14_10330* | outer membrane protein | 28 | 6 | 6 | 20 | 15 | 37 | 3.37386E-14 |
| 842 | 895433 | 893717 | 891558 | - | *-* | *PA14_10340* | toxin transporter | 38 | 7 | 10 | 24 | 16 | 37 | 6.42463E-16 |
| 843 | 895433 | 894983 | 893727 | - | *-* | *PA14_10350* | secretion protein | 84 | 7 | 13 | 41 | 30 | 78 | 1.8387E-103 |
| 844 | 895433 | 895379 | 895080 | - | *-* | *PA14_10360* | hypothetical protein | 6793 | 234 | 525 | 3591 | 3008 | 10535 | 0 |
| 845 | - | 895384 | 895404 | ? | *-* | predicted RNA | - | 1121 | 33 | 42 | 324 | 234 | 1288 | 0 |
| 846 | - | 897481 | 895688 | - | *-* | *PA14_10370* | hypothetical protein | 11 | 6 | 6 | 7 | 5 | 10 | 0.452603163 |
| 847 | - | 897912 | 897613 | - | *-* | *PA14_10380* | hypothetical protein | 32 | 26 | 11 | 17 | 21 | 37 | 1 |
| 848 | - | 898251 | 898045 | - | *-* | *PA14_10400* | hypothetical protein | 1 | 0 | 0 | 1 | 0 | 1 | 3.0938E-17 |
| 849 | - | 898562 | 898269 | - | *-* | *PA14_10410* | hypothetical protein | 0 | 0 | 0 | 0 | 0 | 0 | 0.013995908 |
| 850 | - | 898671 | 899909 | + | *tyrS* | *PA14_10420* | tyrosyl-tRNA synthetase | 5 | 6 | 4 | 6 | 5 | 4 | 1 |
| 851 | - | 901194 | 899938 | - | *-* | *PA14_10440* | porin | 15 | 11 | 14 | 15 | 12 | 13 | 1 |
| 852 | - | 901559 | 902767 | + | *-* | *PA14_10470* | MFS transporter | 60 | 36 | 53 | 50 | 43 | 45 | 1 |
| 853 | - | 902853 | 903275 | + | *-* | *PA14_10480* | transcriptional regulator | 69 | 52 | 65 | 66 | 57 | 58 | 1 |
| 854 | - | 903533 | 903291 | - | *-* | *PA14_10490* | hypothetical protein | 331 | 279 | 304 | 347 | 333 | 302 | 1 |
| 855 | - | 905044 | 903617 | - | *-* | *PA14_10500* | cbb3-type cytochrome c oxidase subunit I | 624 | 653 | 556 | 611 | 750 | 522 | 1 |
| 856 | - | 906723 | 905308 | - | *-* | *PA14_10530* | GntR family transcriptional regulator | 895 | 854 | 805 | 1222 | 1038 | 972 | 1 |
| 857 | - | 906810 | 906820 | ? | *-* | predicted RNA | - | 689 | 713 | 542 | 814 | 811 | 737 | 1 |
| 858 | - | 908542 | 906821 | - | *-* | *PA14_10540* | iron-sulfur cluster-binding protein | 305 | 369 | 253 | 379 | 451 | 312 | 1 |
| 859 | - | 908648 | 910321 | + | *-* | *PA14_10550* | sulfite or nitrite reductas | 615 | 556 | 458 | 711 | 689 | 676 | 1 |
| 860 | - | 910318 | 910806 | + | *-* | *PA14_10560* | hypothetical protein | 899 | 769 | 695 | 1053 | 932 | 977 | 1 |
| 861 | - | 911694 | 910888 | - | *-* | *PA14_10570* | 2,4-dihydroxyhept-2-ene-1,7-dioic acid aldolase | 341 | 261 | 280 | 387 | 352 | 401 | 1 |
| 862 | - | 912510 | 911707 | - | *hpcG* | *PA14_10590* | 2-oxo-hepta-3-ene-1,7-dioic acid hydratase | 124 | 98 | 113 | 145 | 132 | 151 | 1 |
| 863 | - | 913832 | 912528 | - | *-* | *PA14_10600* | MFS transporter | 50 | 39 | 50 | 68 | 70 | 79 | 1 |
| 864 | - | 914317 | 913925 | - | *hpcD* | *PA14_10610* | 5-carboxymethyl-2-hydroxymuconate isomerase | 39 | 33 | 38 | 41 | 32 | 34 | 1 |
| 865 | - | 915251 | 914328 | - | *hpcB* | *PA14_10620* | homoprotocatechuate 2,3-dioxygenase | 21 | 20 | 22 | 28 | 20 | 20 | 1 |
| 866 | - | 916843 | 915383 | - | *hpcC* | *PA14_10630* | 5-carboxy-2-hydroxymuconate semialdehyde dehydrogenase | 12 | 11 | 11 | 13 | 9 | 13 | 1 |
| 867 | - | 917619 | 916840 | - | *-* | *PA14_10640* | hypothetical protein | 7 | 6 | 6 | 8 | 7 | 7 | 1 |
| 868 | - | 918289 | 917630 | - | *-* | *PA14_10650* | hypothetical protein | 7 | 7 | 9 | 7 | 5 | 6 | 1 |
| 869 | - | 918509 | 919420 | + | *-* | *PA14_10660* | transcriptional regulator | 13 | 14 | 14 | 14 | 12 | 14 | 1 |
| 870 | - | 919472 | 920275 | + | *aph* | *PA14_10670* | aminoglycoside 3'-phosphotransferase type IIB | 17 | 20 | 22 | 22 | 18 | 17 | 1 |
| 871 | - | 920968 | 920399 | - | *-* | *PA14_10680* | hypothetical protein | 52 | 48 | 56 | 57 | 55 | 58 | 1 |
| 872 | - | 923232 | 921046 | - | *-* | *PA14_10700* | bacteriophytochrome | 151 | 184 | 194 | 212 | 185 | 209 | 1 |
| 873 | - | 923847 | 923260 | - | *-* | *PA14_10710* | hypothetical protein | 216 | 236 | 327 | 390 | 266 | 284 | 1 |
| 874 | - | 925350 | 923965 | - | *-* | *PA14_10730* | hypothetical protein | 132 | 111 | 137 | 120 | 135 | 146 | 1 |
| 875 | - | 925770 | 926279 | + | *-* | *PA14_10740* | spermidine acetyltransferase | 36 | 42 | 32 | 30 | 36 | 40 | 1 |
| 876 | - | 926433 | 927623 | + | *-* | *PA14_10750* | sugar efflux transporter | 11 | 7 | 13 | 10 | 10 | 8 | 0.543961598 |
| 877 | - | 927751 | 932004 | + | *-* | *PA14_10770* | sensor/response regulator hybrid | 121 | 112 | 149 | 183 | 131 | 168 | 1 |
| 878 | - | 932566 | 932168 | - | *-* | *PA14_10780* | hypothetical protein | 65 | 77 | 91 | 86 | 82 | 78 | 1 |
| 879 | - | 933824 | 932631 | - | *ampC* | *PA14_10790* | beta-lactamase | 13 | 14 | 14 | 14 | 13 | 14 | 1 |
| 880 | - | 933973 | 934863 | + | *ampR* | *PA14_10800* | transcriptional regulator AmpR | 42 | 43 | 43 | 56 | 47 | 46 | 1 |
| 881 | - | 936666 | 935422 | - | *-* | *PA14_10820* | HDIG domain-containing protein | 133 | 121 | 154 | 105 | 138 | 146 | 1 |
| 882 | - | 937824 | 936949 | - | *-* | *PA14_10830* | LysR family transcriptional regulator | 74 | 73 | 78 | 65 | 79 | 73 | 1 |
| 883 | - | 937983 | 938735 | + | *-* | *PA14_10840* | dehydrogenase | 38 | 39 | 36 | 34 | 45 | 36 | 1 |
| 884 | - | 940431 | 938752 | - | *-* | *PA14_10850* | dehydrogenase | 26 | 29 | 24 | 27 | 27 | 25 | 1 |
| 885 | - | 941890 | 940586 | - | *-* | *PA14_10870* | hypothetical protein | 11 | 9 | 9 | 13 | 7 | 9 | 1 |
| 886 | - | 942700 | 941975 | - | *-* | *PA14_10890* | short-chain dehydrogenase | 9 | 11 | 12 | 14 | 10 | 10 | 1 |
| 887 | - | 943773 | 942715 | - | *-* | *PA14_10900* | alcohol dehydrogenase | 3 | 3 | 3 | 5 | 3 | 2 | 1 |
| 888 | - | 945029 | 943770 | - | *-* | *PA14_10910* | major facilitator transporter | 3 | 4 | 3 | 3 | 3 | 2 | 1 |
| 889 | - | 945633 | 945100 | - | *-* | *PA14_10920* | hypothetical protein | 3 | 3 | 4 | 5 | 3 | 4 | 1 |
| 890 | - | 945954 | 946913 | + | *-* | *PA14_10940* | AraC family transcriptional regulator | 25 | 26 | 26 | 28 | 27 | 30 | 1 |
| 891 | - | 946961 | 947377 | + | *-* | *PA14_10950* | hypothetical protein | 10 | 11 | 6 | 10 | 7 | 12 | 1 |
| 892 | - | 948499 | 947387 | - | *-* | *PA14_10960* | ferredoxin oxidoreductase subunit | 10 | 9 | 8 | 10 | 9 | 11 | 1 |
| 893 | - | 949493 | 948528 | - | *-* | *PA14_10970* | flavodoxin reductase | 10 | 8 | 10 | 10 | 9 | 10 | 1 |
| 894 | - | 949604 | 950458 | + | *-* | *PA14_10980* | hypothetical protein | 11 | 9 | 11 | 9 | 10 | 11 | 1 |
| 895 | - | 951014 | 950502 | - | *hpaC* | *PA14_10990* | 4-hydroxyphenylacetate 3-monooxygenase small subunit | 4 | 5 | 3 | 5 | 4 | 4 | 1 |
| 896 | - | 952606 | 951044 | - | *hpaA* | *PA14_11000* | 4-hydroxyphenylacetate 3-monooxygenase large chain | 5 | 6 | 6 | 6 | 6 | 6 | 1 |
| 897 | - | 953062 | 952772 | - | *-* | *PA14_11010* | hypothetical protein | 16 | 27 | 15 | 33 | 33 | 21 | 1 |
| 898 | - | 954126 | 953365 | - | *-* | *PA14_11020* | 3-ketoacyl-ACP reductase | 16 | 17 | 17 | 21 | 13 | 17 | 1 |
| 899 | - | 955508 | 954123 | - | *-* | *PA14_11030* | aminotransferase | 5 | 7 | 6 | 8 | 5 | 7 | 1 |
| 900 | - | 956341 | 955517 | - | *-* | *PA14_11050* | hypothetical protein | 1 | 2 | 1 | 2 | 2 | 2 | 0.129649142 |
| 901 | - | 956812 | 957381 | + | *cupB1* | *PA14_11060* | fimbrial subunit CupB1 | 12 | 9 | 8 | 6 | 11 | 10 | 1 |
| 902 | - | 957412 | 958158 | + | *cupB2* | *PA14_11070* | chaperone CupB2 | 1 | 1 | 2 | 1 | 1 | 1 | 0.842469071 |
| 903 | - | 958480 | 961014 | + | *cupB3* | *PA14_11080* | usher CupB3 | 16 | 20 | 21 | 24 | 21 | 25 | 1 |
| 904 | - | 961011 | 961751 | + | *cupB4* | *PA14_11090* | chaperone CupB4 | 2 | 2 | 2 | 1 | 1 | 1 | 1 |
| 905 | - | 961839 | 964895 | + | *cupB5* | *PA14_11100* | adhesive protein CupB5 | 5 | 6 | 5 | 6 | 6 | 5 | 1 |
| 906 | - | 964995 | 966140 | + | *cupB6* | *PA14_11110* | fimbrial protein cupB6 | 6 | 6 | 6 | 6 | 6 | 6 | 1 |
| 907 | - | 966785 | 967429 | + | *-* | *PA14_11120* | response regulator | 40 | 46 | 35 | 47 | 50 | 56 | 1 |
| 908 | - | 968133 | 967444 | - | *-* | *PA14_11130* | short chain dehydrogenase | 135 | 70 | 78 | 97 | 88 | 178 | 0.785036578 |
| 909 | - | 971198 | 968223 | - | *-* | *PA14_11140* | nonribosomal peptide synthetase | 109 | 38 | 42 | 68 | 45 | 141 | 0.452109894 |
| 910 | - | 971615 | 971409 | - | *-* | *PA14_11150* | transcriptional regulator | 7 | 11 | 7 | 6 | 9 | 9 | 1 |
| 911 | - | 971986 | 971612 | - | *-* | *PA14_11160* | hypothetical protein | 5 | 7 | 5 | 5 | 8 | 6 | 1 |
| 912 | - | 972093 | 972929 | + | *-* | *PA14_11170* | hypothetical protein | 78 | 80 | 70 | 78 | 79 | 68 | 1 |
| 913 | - | 973594 | 972926 | - | *-* | *PA14_11180* | transcriptional regulator | 22 | 25 | 22 | 23 | 19 | 21 | 1 |
| 914 | - | 973823 | 975310 | + | *-* | *PA14_11190* | aldehyde dehydrogenase | 8 | 12 | 8 | 14 | 12 | 10 | 1 |
| 915 | - | 975501 | 976991 | + | *-* | *PA14_11210* | amino acid permease | 6 | 8 | 7 | 8 | 8 | 7 | 1 |
| 916 | - | 976966 | 977544 | + | *-* | *PA14_11230* | hypothetical protein | 7 | 6 | 9 | 12 | 8 | 8 | 1 |
| 917 | - | 977705 | 978655 | + | *-* | *PA14_11240* | DNA-binding transcriptional activator FeaR | 29 | 28 | 25 | 34 | 24 | 33 | 1 |
| 918 | - | 978924 | 979808 | + | *-* | *PA14_11250* | hypothetical protein | 83 | 71 | 80 | 66 | 73 | 64 | 1 |
| 919 | - | 979801 | 980730 | + | *-* | *PA14_11260* | epimerase | 192 | 205 | 190 | 189 | 204 | 178 | 1 |
| 920 | - | 981869 | 981171 | - | *oprG* | *PA14_11270* | outer membrane protein OprG precursor | 1993 | 2754 | 2204 | 947 | 1496 | 1484 | 1 |
| 921 | - | 982535 | 982017 | - | *-* | *PA14_11280* | hypothetical protein | 29 | 30 | 32 | 36 | 32 | 32 | 1 |
| 922 | - | 983818 | 982553 | - | *-* | *PA14_11290* | permease | 29 | 24 | 25 | 27 | 25 | 30 | 1 |
| 923 | - | 984525 | 983821 | - | *-* | *PA14_11310* | ABC transporter ATP-binding protein | 17 | 13 | 17 | 15 | 14 | 14 | 1 |
| 924 | - | 985204 | 984614 | - | *-* | *PA14_11320* | hypothetical protein | 24 | 17 | 23 | 24 | 31 | 43 | 1 |

|  | A | B | C | D | E | F | G | H | I | J | K | L | M | N |
| --- | --- | --- | --- | --- | --- | --- | --- | --- | --- | --- | --- | --- | --- | --- |
| 925 | - | 985337 | 985681 | + | *-* | *PA14_11330* | hypothetical protein | 73 | 60 | 72 | 63 | 63 | 59 | 1 |
| 926 | - | 986587 | 985718 | - | *-* | *PA14_11340* | thioredoxin | 135 | 126 | 178 | 79 | 114 | 92 | 1 |
| 927 | - | 987321 | 986659 | - | *-* | *PA14_11350* | hypothetical protein | 34 | 29 | 37 | 30 | 28 | 28 | 1 |
| 928 | - | 987788 | 987318 | - | *-* | *PA14_11370* | lipoprotein | 21 | 21 | 23 | 20 | 21 | 21 | 1 |
| 929 | - | 987974 | 988438 | + | *nrdR* | *PA14_11380* | transcriptional regulator NrdR | 67 | 54 | 67 | 50 | 47 | 47 | 1 |
| 930 | - | 988435 | 989556 | + | *ribD* | *PA14_11400* | riboflavin-specific deaminase/reductase | 310 | 265 | 313 | 318 | 267 | 259 | 1 |
| 931 | - | 989984 | 990643 | + | *ribC* | *PA14_11410* | riboflavin synthase subunit alpha | 49 | 42 | 48 | 35 | 39 | 41 | 1 |
| 932 | - | 990670 | 991767 | + | *ribB* | *PA14_11420* | bifunctional 3,4-dihydroxy-2-butanone 4-phosphate synthase/GTP cyclohydrolase II-like protein | 55 | 56 | 55 | 40 | 56 | 44 | 1 |
| 933 | - | 991885 | 992361 | + | *ribH* | *PA14_11430* | 6,7-dimethyl-8-ribityllumazine synthase | 134 | 143 | 160 | 132 | 116 | 109 | 1 |
| 934 | - | 992358 | 992837 | + | *nusB* | *PA14_11450* | transcription antitermination protein NusB | 142 | 171 | 168 | 131 | 133 | 109 | 1 |
| 935 | - | 992856 | 993824 | + | *thiL* | *PA14_11460* | thiamine monophosphate kinase | 72 | 64 | 78 | 55 | 67 | 48 | 1 |
| 936 | - | 993817 | 994332 | + | *pgpA* | *PA14_11470* | phosphatidylglycerophosphatase A | 74 | 69 | 94 | 70 | 78 | 64 | 1 |
| 937 | - | 994341 | 995087 | + | *-* | *PA14_11480* | hypothetical protein | 93 | 79 | 119 | 105 | 98 | 98 | 1 |
| 938 | - | 995090 | 995734 | + | *-* | *PA14_11490* | hypothetical protein | 104 | 100 | 142 | 107 | 109 | 103 | 1 |
| 939 | - | 995865 | 996482 | + | *ribA* | *PA14_11510* | GTP cyclohydrolase II | 86 | 65 | 95 | 62 | 66 | 71 | 1 |
| 940 | - | 996479 | 996898 | + | *-* | *PA14_11520* | hypothetical protein | 56 | 54 | 63 | 41 | 52 | 44 | 1 |
| 941 | - | 996898 | 997695 | + | *-* | *PA14_11530* | hypothetical protein | 58 | 48 | 63 | 51 | 51 | 48 | 1 |
| 942 | - | 999702 | 997819 | - | *dxs* | *PA14_11550* | 1-deoxy-D-xylulose-5-phosphate synthase | 120 | 112 | 122 | 112 | 111 | 99 | 1 |
| 943 | - | 1000698 | 999811 | - | *ispA* | *PA14_11560* | geranyltranstransferase | 60 | 54 | 67 | 70 | 59 | 62 | 1 |
| 944 | - | 1000937 | 1000695 | - | *xseB* | *PA14_11570* | exodeoxyribonuclease VII small subunit | 34 | 26 | 40 | 30 | 29 | 28 | 1 |
| 945 | - | 1001095 | 1002264 | + | *-* | *PA14_11580* | hypothetical protein | 79 | 73 | 89 | 93 | 70 | 82 | 1 |
| 946 | - | 1003503 | 1002496 | - | *-* | *PA14_11590* | hypothetical protein | 24 | 24 | 24 | 27 | 21 | 28 | 1 |
| 947 | - | 1005307 | 1003505 | - | *-* | *PA14_11600* | ABC transporter | 47 | 45 | 49 | 52 | 42 | 50 | 1 |
| 948 | - | 1006051 | 1005317 | - | *-* | *PA14_11610* | ABC transporter permease | 6 | 5 | 8 | 8 | 7 | 6 | 1 |
| 949 | - | 1006974 | 1006048 | - | *-* | *PA14_11620* | ABC transporter | 7 | 7 | 9 | 9 | 6 | 8 | 1 |
| 950 | - | 1009679 | 1007379 | - | *-* | *PA14_11630* | two-component sensor | 31 | 36 | 36 | 38 | 38 | 35 | 1 |
| 951 | - | 1010699 | 1009698 | - | *-* | *PA14_11650* | hypothetical protein | 36 | 39 | 36 | 32 | 29 | 26 | 1 |
| 952 | - | 1011554 | 1010865 | - | *aqpZ* | *PA14_11660* | aquaporin Z | 9 | 9 | 8 | 8 | 7 | 7 | 1 |
| 953 | - | 1012170 | 1011901 | - | *-* | *PA14_11670* | hypothetical protein | 12 | 11 | 11 | 14 | 8 | 12 | 1 |
| 954 | - | 1012365 | 1013081 | + | *-* | *PA14_11680* | two-component regulator | 87 | 73 | 95 | 71 | 63 | 70 | 1 |
| 955 | - | 1013699 | 1013172 | - | *ppa* | *PA14_11690* | inorganic pyrophosphatase | 89 | 127 | 81 | 54 | 79 | 49 | 1 |
| 956 | - | 1014609 | 1013779 | - | *-* | *PA14_11700* | hypothetical protein | 26 | 24 | 34 | 22 | 22 | 21 | 1 |
| 957 | - | 1015277 | 1014612 | - | *-* | *PA14_11720* | hypothetical protein | 31 | 24 | 36 | 20 | 21 | 17 | 1 |
| 958 | - | 1015471 | 1017054 | + | *-* | *PA14_11730* | protein kinase | 24 | 22 | 24 | 23 | 22 | 22 | 1 |
| 959 | - | 1017840 | 1016947 | - | *-* | *PA14_11740* | hypothetical protein | 64 | 73 | 71 | 64 | 73 | 81 | 1 |
| 960 | - | 1018351 | 1017890 | - | *-* | *PA14_11750* | acetyltransferase | 125 | 119 | 111 | 92 | 111 | 120 | 1 |
| 961 | - | 1019336 | 1018515 | - | *-* | *PA14_11760* | ethanolamine ammonia-lyase small subunit | 51 | 58 | 58 | 67 | 47 | 63 | 1 |
| 962 | - | 1020741 | 1019347 | - | *eutB* | *PA14_11770* | ethanolamine ammonia-lyase large subunit | 54 | 69 | 74 | 73 | 58 | 73 | 1 |
| 963 | - | 1022248 | 1020800 | - | *-* | *PA14_11790* | amino acid transporter | 22 | 29 | 33 | 29 | 23 | 24 | 1 |
| 964 | - | 1023978 | 1022458 | - | *-* | *PA14_11810* | aldehyde dehydrogenase | 157 | 285 | 203 | 174 | 177 | 162 | 1 |
| 965 | - | 1024317 | 1026248 | + | *-* | *PA14_11830* | transcriptional regulator | 101 | 108 | 94 | 103 | 91 | 90 | 1 |
| 966 | - | 1026406 | 1027761 | + | *mpl* | *PA14_11845* | UDP-N-acetylmuramate:L-alanyl-gamma-D-glutamyl-meso-diaminopimelate ligase | 117 | 111 | 114 | 114 | 117 | 114 | 1 |
| 967 | - | 1027770 | 1028399 | + | *-* | *PA14_11860* | aromatic acid decarboxylase | 160 | 170 | 166 | 152 | 163 | 148 | 1 |
| 968 | - | 1028401 | 1028676 | + | *-* | *PA14_11880* | hypothetical protein | 157 | 176 | 188 | 165 | 166 | 185 | 1 |
| 969 | - | 1029337 | 1028696 | - | *-* | *PA14_11890* | hypothetical protein | 264 | 309 | 366 | 298 | 364 | 386 | 1 |
| 970 | - | 1031123 | 1029384 | - | *-* | *PA14_11900* | hypothetical protein | 39 | 35 | 39 | 36 | 31 | 32 | 1 |
| 971 | - | 1031769 | 1031314 | - | *-* | *PA14_11910* | hypothetical protein | 78 | 97 | 73 | 82 | 105 | 89 | 1 |
| 972 | - | 1031915 | 1032325 | + | *-* | *PA14_11920* | hypothetical protein | 43 | 38 | 48 | 39 | 35 | 29 | 1 |
| 973 | - | 1032325 | 1033035 | + | *-* | *PA14_11930* | hypothetical protein | 80 | 68 | 88 | 78 | 70 | 61 | 1 |
| 974 | - | 1033055 | 1033648 | + | *-* | *PA14_11940* | hypothetical protein | 248 | 239 | 278 | 289 | 264 | 257 | 1 |
| 975 | - | 1034972 | 1033659 | - | *-* | *PA14_11960* | hypothetical protein | 79 | 75 | 79 | 77 | 75 | 72 | 1 |
| 976 | - | 1035688 | 1034969 | - | *-* | *PA14_11970* | 3-methyladenine DNA glycosylase | 67 | 45 | 63 | 58 | 51 | 51 | 1 |
| 977 | - | 1035752 | 1036609 | + | *-* | *PA14_11980* | hypothetical protein | 23 | 21 | 24 | 27 | 22 | 23 | 1 |
| 978 | - | 1037840 | 1036617 | - | *-* | *PA14_11990* | hydrolase | 25 | 26 | 31 | 31 | 25 | 34 | 1 |
| 979 | - | 1038138 | 1039403 | + | *proA* | *PA14_12010* | gamma-glutamyl phosphate reductase | 117 | 114 | 129 | 120 | 97 | 90 | 1 |
| 980 | - | 1039403 | 1040047 | + | *nadD* | *PA14_12020* | nicotinic acid mononucleotide adenylyltransferase | 229 | 225 | 285 | 248 | 221 | 189 | 1 |
| 981 | - | 1040070 | 1040426 | + | *-* | *PA14_12030* | hypothetical protein | 275 | 365 | 360 | 284 | 286 | 276 | 1 |
| 982 | - | 1040434 | 1040901 | + | *-* | *PA14_12050* | rRNA large subunit methyltransferase | 206 | 188 | 253 | 181 | 181 | 165 | 1 |
| 983 | - | 1040914 | 1042854 | + | *pbpA* | *PA14_12060* | penicillin-binding protein 2 | 73 | 64 | 84 | 68 | 72 | 60 | 1 |
| 984 | - | 1042886 | 1043989 | + | *rodA* | *PA14_12070* | rod shape-determining protein | 74 | 65 | 82 | 81 | 79 | 67 | 1 |
| 985 | - | 1043998 | 1045020 | + | *sltB1* | *PA14_12080* | soluble lytic transglycosylase B | 179 | 126 | 174 | 185 | 182 | 165 | 1 |
| 986 | - | 1045017 | 1046042 | + | *-* | *PA14_12090* | RlpA family lipoprotein | 301 | 277 | 327 | 319 | 279 | 272 | 1 |
| 987 | - | 1046108 | 1047268 | + | *dacC* | *PA14_12100* | D-ala-D-ala-carboxypeptidase | 368 | 402 | 355 | 361 | 380 | 332 | 1 |
| 988 | - | 1047339 | 1047620 | + | *-* | *PA14_12110* | hypothetical protein | 324 | 354 | 336 | 285 | 306 | 282 | 1 |
| 989 | - | 1047620 | 1048273 | + | *lipB* | *PA14_12120* | lipoate-protein ligase B | 260 | 229 | 253 | 237 | 225 | 197 | 1 |
| 990 | - | 1048270 | 1049253 | + | *lis* | *PA14_12130* | lipoyl synthase | 523 | 453 | 542 | 508 | 544 | 525 | 1 |
| 991 | - | 1050291 | 1049398 | - | *-* | *PA14_12140* | transcriptional regulator | 40 | 35 | 42 | 40 | 39 | 44 | 1 |
| 992 | - | 1050399 | 1051289 | + | *-* | *PA14_12150* | epoxide hydrolase | 9 | 9 | 9 | 11 | 10 | 9 | 1 |
| 993 | - | 1052838 | 1051492 | - | *-* | *PA14_12160* | murein transglycosylase | 57 | 62 | 63 | 52 | 65 | 54 | 1 |
| 994 | - | 1053067 | 1053285 | + | *-* | *PA14_12170* | hypothetical protein | 9 | 9 | 13 | 10 | 10 | 10 | 1 |
| 995 | - | 1053553 | 1053401 | - | *-* | *PA14_12180* | hypothetical protein | 7 | 7 | 9 | 7 | 9 | 7 | 1 |
| 996 | - | 1054682 | 1053645 | - | *holA* | *PA14_12200* | DNA polymerase III subunit delta | 65 | 68 | 53 | 47 | 59 | 52 | 1 |
| 997 | - | 1055343 | 1054720 | - | *-* | *PA14_12210* | hypothetical protein | 137 | 148 | 112 | 112 | 147 | 108 | 1 |
| 998 | - | 1058031 | 1055410 | - | *leuS* | *PA14_12230* | leucyl-tRNA synthetase | 192 | 205 | 182 | 175 | 185 | 164 | 1 |
| 999 | - | 1058202 | 1058633 | + | *-* | *PA14_12260* | hypothetical protein | 434 | 361 | 586 | 420 | 394 | 424 | 1 |
| 1000 | - | 1058706 | 1059467 | + | *-* | *PA14_12270* | hypothetical protein | 62 | 54 | 61 | 57 | 56 | 54 | 1 |
| 1001 | - | 1061052 | 1059517 | - | *lnt* | *PA14_12280* | apolipoprotein N-acyltransferase | 41 | 44 | 33 | 36 | 36 | 29 | 1 |
| 1002 | - | 1062077 | 1061238 | - | *-* | *PA14_12300* | hypothetical protein | 147 | 150 | 142 | 114 | 126 | 125 | 1 |
| 1003 | - | 1062556 | 1062074 | - | *-* | *PA14_12310* | metalloprotease | 165 | 172 | 191 | 171 | 218 | 186 | 1 |
| 1004 | - | 1063568 | 1062546 | - | *-* | *PA14_12330* | hypothetical protein | 299 | 308 | 308 | 248 | 280 | 290 | 1 |
| 1005 | - | 1065077 | 1063737 | - | *-* | *PA14_12350* | (dimethylallyl)adenosine tRNA methylthiotransferase | 62 | 61 | 70 | 67 | 57 | 51 | 1 |
| 1006 | - | 1065206 | 1065538 | + | *-* | *PA14_12360* | hypothetical protein | 30 | 27 | 38 | 28 | 28 | 31 | 1 |
| 1007 | - | 1066102 | 1065554 | - | *-* | *PA14_12370* | hypothetical protein | 244 | 243 | 309 | 213 | 283 | 249 | 1 |
| 1008 | - | 1067594 | 1066311 | - | *hemL* | *PA14_12390* | glutamate-1-semialdehyde aminotransferase | 123 | 131 | 106 | 95 | 115 | 89 | 1 |

|  | A | B | C | D | E | F | G | H | I | J | K | L | M | N |
| --- | --- | --- | --- | --- | --- | --- | --- | --- | --- | --- | --- | --- | --- | --- |
| 1009 | - | 1068261 | 1067632 | - | *thiE* | *PA14_12400* | thiamine-phosphate pyrophosphorylase | 49 | 50 | 41 | 44 | 38 | 36 | 1 |
| 1010 | - | 1069069 | 1068272 | - | *thiD* | *PA14_12410* | phosphomethylpyrimidine kinase | 96 | 66 | 83 | 71 | 70 | 67 | 1 |
| 1011 | - | 1071822 | 1072469 | + | *-* | *PA14_12440* | AcrR family transcriptional regulator | 170 | 136 | 145 | 145 | 185 | 225 | 1 |
| 1012 | - | 1072466 | 1074115 | + | *-* | *PA14_12450* | acyl-CoA dehydrogenase | 126 | 126 | 112 | 120 | 178 | 194 | 1 |
| 1013 | - | 1074127 | 1074558 | + | *-* | *PA14_12470* | hypothetical protein | 71 | 61 | 79 | 64 | 81 | 91 | 1 |
| 1014 | - | 1074613 | 1076112 | + | *-* | *PA14_12490* | AMP nucleosidase | 75 | 81 | 65 | 62 | 77 | 70 | 1 |
| 1015 | - | 1077872 | 1076787 | - | *-* | *PA14_12530* | hypothetical protein | 56 | 55 | 52 | 43 | 52 | 47 | 1 |
| 1016 | - | 1078011 | 1078580 | + | *-* | *PA14_12540* | pseudouridine synthase | 29 | 30 | 27 | 25 | 23 | 18 | 1 |
| 1017 | - | 1079006 | 1078614 | - | *-* | *PA14_12550* | hypothetical protein | 31 | 23 | 27 | 20 | 24 | 20 | 1 |
| 1018 | - | 1079316 | 1079107 | - | *-* | *PA14_12560* | hypothetical protein | 160 | 78 | 199 | 126 | 131 | 162 | 0.243629287 |
| 1019 | - | 1080027 | 1079518 | - | *-* | *PA14_12570* | transcription regulator AsnC | 20 | 34 | 26 | 23 | 22 | 22 | 1 |
| 1020 | - | 1081194 | 1080409 | - | *-* | *PA14_12590* | hypothetical protein | 63 | 74 | 69 | 112 | 51 | 49 | 1 |
| 1021 | - | 1082047 | 1081151 | - | *-* | *PA14_12610* | transporter | 35 | 41 | 35 | 46 | 34 | 30 | 1 |
| 1022 | - | 1082552 | 1082136 | - | *-* | *PA14_12620* | hypothetical protein | 40 | 43 | 33 | 26 | 40 | 31 | 1 |
| 1023 | - | 1082719 | 1085211 | + | *-* | *PA14_12630* | ATP-dependent helicase | 66 | 67 | 66 | 60 | 59 | 49 | 1 |
| 1024 | - | 1085629 | 1085222 | - | *-* | *PA14_12640* | hypothetical protein | 30 | 38 | 33 | 36 | 37 | 37 | 1 |
| 1025 | - | 1086387 | 1085653 | - | *-* | *PA14_12650* | hypothetical protein | 11 | 9 | 13 | 14 | 9 | 8 | 1 |
| 1026 | - | 1087616 | 1086477 | - | *-* | *PA14_12670* | hypothetical protein | 39 | 30 | 40 | 39 | 34 | 39 | 1 |
| 1027 | - | 1087678 | 1088514 | + | *-* | *PA14_12680* | short chain dehydrogenase | 119 | 117 | 144 | 141 | 140 | 149 | 1 |
| 1028 | - | 1088955 | 1088521 | - | *-* | *PA14_12690* | hypothetical protein | 51 | 60 | 75 | 62 | 50 | 52 | 1 |
| 1029 | - | 1089678 | 1089022 | - | *-* | *PA14_12700* | hypothetical protein | 23 | 21 | 26 | 27 | 19 | 21 | 1 |
| 1030 | - | 1090856 | 1089762 | - | *-* | *PA14_12710* | hypothetical protein | 15 | 16 | 15 | 15 | 15 | 12 | 1 |
| 1031 | - | 1091595 | 1091014 | - | *-* | *PA14_12730* | hypothetical protein | 10 | 6 | 13 | 7 | 5 | 8 | 0.499266146 |
| 1032 | - | 1091836 | 1092411 | + | *-* | *PA14_12740* | hypothetical protein | 284 | 240 | 461 | 155 | 232 | 158 | 1 |
| 1033 | - | 1092477 | 1092980 | + | *-* | *PA14_12750* | hypothetical protein | 79 | 66 | 108 | 52 | 71 | 57 | 1 |
| 1034 | - | 1093058 | 1094407 | + | *-* | *PA14_12760* | ATP-dependent RNA helicase | 61 | 60 | 63 | 58 | 57 | 49 | 1 |
| 1035 | - | 1095605 | 1094430 | - | *-* | *PA14_12770* | hypothetical protein | 78 | 59 | 74 | 62 | 53 | 53 | 1 |
| 1036 | - | 1096593 | 1095964 | - | *-* | *PA14_12780* | two-component response regulator | 94 | 73 | 106 | 71 | 82 | 70 | 1 |
| 1037 | - | 1096875 | 1098053 | + | *-* | *PA14_12810* | two-component response regulator | 22 | 25 | 23 | 22 | 21 | 23 | 1 |
| 1038 | - | 1098155 | 1101793 | + | *-* | *PA14_12820* | two-component sensor | 10 | 10 | 11 | 10 | 10 | 10 | 1 |
| 1039 | - | 1102008 | 1102598 | + | *-* | *PA14_12840* | acetyltransferase | 367 | 345 | 443 | 364 | 385 | 440 | 1 |
| 1040 | - | 1102625 | 1103203 | + | *-* | *PA14_12850* | acetyltransferase | 116 | 103 | 112 | 91 | 98 | 99 | 1 |
| 1041 | - | 1103206 | 1104801 | + | *-* | *PA14_12860* | hypothetical protein | 93 | 79 | 87 | 75 | 85 | 88 | 1 |
| 1042 | - | 1104835 | 1105704 | + | *tesB* | *PA14_12870* | acyl-CoA thioesterase | 53 | 44 | 50 | 41 | 49 | 46 | 1 |
| 1043 | - | 1105701 | 1106297 | + | *-* | *PA14_12890* | hypothetical protein | 55 | 68 | 63 | 56 | 64 | 62 | 1 |
| 1044 | - | 1106702 | 1106421 | - | *-* | *PA14_12900* | DNA binding protein | 205 | 517 | 218 | 177 | 212 | 193 | 0.11934908 |
| 1045 | - | 1107072 | 1107251 | + | *-* | *PA14_12910* | hypothetical protein | 15 | 11 | 6 | 6 | 10 | 9 | 1 |
| 1046 | - | 1107458 | 1108471 | + | *-* | *PA14_12920* | taurine ABC transporter periplasmic protein | 10 | 12 | 11 | 11 | 8 | 10 | 1 |
| 1047 | - | 1108535 | 1109326 | + | *-* | *PA14_12940* | taurine ABC transporter ATP-binding protein | 6 | 7 | 7 | 7 | 6 | 7 | 1 |
| 1048 | - | 1109316 | 1110134 | + | *-* | *PA14_12960* | taurine ABC transporter permease | 6 | 5 | 5 | 6 | 4 | 4 | 1 |
| 1049 | - | 1110241 | 1111074 | + | *tauD* | *PA14_12970* | taurine dioxygenase | 12 | 10 | 11 | 12 | 9 | 10 | 1 |
| 1050 | - | 1111237 | 1113273 | + | *-* | *PA14_12980* | hypothetical protein | 68 | 88 | 75 | 110 | 84 | 74 | 1 |
| 1051 | - | 1115342 | 1113381 | - | *-* | *PA14_12990* | choline transporter | 85 | 59 | 72 | 70 | 72 | 78 | 1 |
| 1052 | - | 1116506 | 1115532 | - | *-* | *PA14_13000* | transcriptional regulator | 11 | 15 | 10 | 10 | 12 | 13 | 1 |
| 1053 | - | 1117461 | 1116682 | - | *-* | *PA14_13010* | hypothetical protein | 15 | 50 | 15 | 12 | 23 | 14 | 0.000919853 |
| 1054 | - | 1117899 | 1119365 | + | *cioA* | *PA14_13030* | CioA, cyanide insensitive terminal oxidase | 227 | 165 | 136 | 273 | 277 | 227 | 1 |
| 1055 | - | 1119369 | 1120376 | + | *cioB* | *PA14_13040* | CioB, cyanide insensitive terminal oxidase | 310 | 208 | 197 | 321 | 311 | 295 | 1 |
| 1056 | - | 1120390 | 1120560 | + | *-* | *PA14_13050* | hypothetical protein | 102 | 81 | 65 | 113 | 121 | 108 | 1 |
| 1057 | - | 1121623 | 1120835 | - | *-* | *PA14_13060* | transcriptional regulator | 18 | 22 | 17 | 17 | 19 | 18 | 1 |
| 1058 | - | 1121725 | 1122906 | + | *-* | *PA14_13070* | MFS family transporter | 12 | 11 | 14 | 16 | 11 | 10 | 1 |
| 1059 | - | 1124138 | 1122963 | - | *-* | *PA14_13090* | acyl-CoA thiolase | 35 | 39 | 31 | 33 | 31 | 29 | 1 |
| 1060 | - | 1124320 | 1126002 | + | *-* | *PA14_13110* | long-chain-fatty-acid--CoA ligase | 41 | 51 | 46 | 66 | 46 | 62 | 1 |
| 1061 | - | 1126214 | 1128139 | + | *-* | *PA14_13130* | hypothetical protein | 70 | 91 | 71 | 129 | 79 | 116 | 1 |
| 1062 | - | 1128200 | 1129567 | + | *-* | *PA14_13140* | hypothetical protein | 61 | 92 | 62 | 122 | 78 | 109 | 1 |
| 1063 | - | 1129901 | 1132621 | + | *-* | *PA14_13150* | transcriptional regulator | 132 | 142 | 136 | 164 | 129 | 154 | 1 |
| 1064 | - | 1135031 | 1132653 | - | *-* | *PA14_13170* | metal transporting P-type ATPase | 42 | 61 | 29 | 67 | 69 | 55 | 1 |
| 1065 | - | 1135535 | 1136926 | + | *-* | *PA14_13190* | hypothetical protein | 206 | 211 | 187 | 185 | 207 | 264 | 1 |
| 1066 | - | 1137043 | 1137819 | + | *-* | *PA14_13200* | hypothetical protein | 33 | 29 | 32 | 35 | 34 | 52 | 1 |
| 1067 | - | 1138011 | 1137829 | - | *-* | *PA14_13210* | hypothetical protein | 73 | 43 | 53 | 98 | 83 | 166 | 0.43335915 |
| 1068 | - | 1138602 | 1138174 | - | *-* | *PA14_13220* | protein-tyrosine-phosphatase | 26 | 18 | 23 | 16 | 17 | 19 | 1 |
| 1069 | - | 1138820 | 1139302 | + | *moaC* | *PA14_13230* | molybdenum cofactor biosynthesis protein MoaC | 59 | 61 | 68 | 91 | 58 | 233 | 1 |
| 1070 | - | 1139299 | 1139550 | + | *moaD* | *PA14_13240* | molybdopterin converting factor, small subunit | 99 | 92 | 105 | 136 | 139 | 350 | 1 |
| 1071 | - | 1139555 | 1140007 | + | *moaE* | *PA14_13250* | molybdopterin converting factor, large subunit | 53 | 51 | 47 | 60 | 64 | 141 | 1 |
| 1072 | - | 1140109 | 1140666 | + | *moaB1* | *PA14_13260* | MoaB1 | 7 | 25 | 11 | 125 | 98 | 417 | 1.45127E-05 |
| 1073 | - | 1140663 | 1141886 | + | *moeA1* | *PA14_13280* | molybdenum cofactor biosynthetic protein A1 | 7 | 27 | 9 | 30 | 55 | 185 | 4.73358E-05 |
| 1074 | - | 1142001 | 1142996 | + | *-* | *PA14_13290* | protease | 41 | 63 | 51 | 78 | 91 | 103 | 1 |
| 1075 | - | 1143008 | 1143898 | + | *-* | *PA14_13300* | hypothetical protein | 49 | 102 | 71 | 96 | 139 | 101 | 0.391278558 |
| 1076 | - | 1143892 | 1144407 | + | *-* | *PA14_13320* | hypothetical protein | 44 | 88 | 71 | 83 | 138 | 86 | 0.328493033 |
| 1077 | - | 1144659 | 1146221 | + | *-* | *PA14_13330* | hypothetical protein | 9 | 10 | 11 | 13 | 11 | 11 | 1 |
| 1078 | - | 1146258 | 1148600 | + | *-* | *PA14_13340* | extracellular nuclease | 12 | 7 | 7 | 9 | 7 | 12 | 0.711763148 |
| 1079 | - | 1149641 | 1148922 | - | *-* | *PA14_13350* | hypothetical protein | 142 | 217 | 164 | 165 | 250 | 149 | 1 |
| 1080 | - | 1150423 | 1149638 | - | *-* | *PA14_13360* | hypothetical protein | 140 | 165 | 148 | 143 | 193 | 115 | 1 |
| 1081 | - | 1150803 | 1150420 | - | *-* | *PA14_13370* | hypothetical protein | 159 | 180 | 174 | 190 | 219 | 129 | 1 |
| 1082 | - | 1151323 | 1150796 | - | *-* | *PA14_13380* | hypothetical protein | 110 | 130 | 125 | 121 | 137 | 89 | 1 |
| 1083 | - | 1151715 | 1151320 | - | *-* | *PA14_13390* | hypothetical protein | 112 | 133 | 137 | 154 | 112 | 99 | 1 |
| 1084 | - | 1153683 | 1152100 | - | *prfC* | *PA14_13410* | peptide chain release factor 3 | 47 | 51 | 63 | 52 | 48 | 37 | 1 |
| 1085 | - | 1153904 | 1154365 | + | *-* | *PA14_13420* | hypothetical protein | 40 | 46 | 42 | 35 | 47 | 35 | 1 |
| 1086 | - | 1156819 | 1154465 | - | *fecA* | *PA14_13430* | Fe(III) dicitrate transport protein FecA | 16 | 17 | 17 | 19 | 20 | 14 | 1 |
| 1087 | - | 1157867 | 1156914 | - | *-* | *PA14_13450* | transmembrane sensor | 18 | 29 | 25 | 67 | 43 | 20 | 0.934705131 |
| 1088 | - | 1158373 | 1157864 | - | *-* | *PA14_13460* | RNA polymerase sigma factor | 7 | 31 | 10 | 80 | 70 | 12 | 3.18925E-09 |
| 1089 | - | 1159355 | 1158474 | - | *-* | *PA14_13470* | AraC family transcriptional regulator | 21 | 19 | 21 | 23 | 19 | 19 | 1 |
| 1090 | - | 1159481 | 1160383 | + | *-* | *PA14_13490* | hypothetical protein | 15 | 16 | 14 | 16 | 14 | 12 | 1 |
| 1091 | - | 1161378 | 1160401 | - | *-* | *PA14_13500* | 2-hydroxyacid dehydrogenase | 22 | 25 | 21 | 20 | 24 | 18 | 1 |
| 1092 | - | 1162426 | 1161473 | - | *-* | *PA14_13510* | LysR family transcriptional regulator | 31 | 31 | 32 | 28 | 31 | 31 | 1 |

|  | A | B | C | D | E | F | G | H | I | J | K | L | M | N |
| --- | --- | --- | --- | --- | --- | --- | --- | --- | --- | --- | --- | --- | --- | --- |
| 1093 | - | 1162672 | 1164162 | + | *-* | *PA14_13520* | outer membrane protein | 20 | 13 | 17 | 15 | 13 | 15 | 1 |
| 1094 | - | 1164159 | 1166354 | + | *-* | *PA14_13530* | outer membrane protein | 12 | 10 | 10 | 10 | 10 | 10 | 1 |
| 1095 | - | 1166581 | 1167489 | + | *-* | *PA14_13560* | hypothetical protein | 11 | 8 | 10 | 8 | 13 | 9 | 1 |
| 1096 | - | 1168780 | 1167617 | - | *-* | *PA14_13580* | ABC transporter ATP-binding protein | 64 | 87 | 73 | 71 | 98 | 81 | 1 |
| 1097 | - | 1169455 | 1168793 | - | *-* | *PA14_13590* | ABC transporter permease | 63 | 65 | 67 | 63 | 84 | 84 | 1 |
| 1098 | - | 1170390 | 1169455 | - | *-* | *PA14_13600* | ABC transporter substrate-binding protein | 50 | 51 | 51 | 53 | 66 | 57 | 1 |
| 1099 | - | 1171112 | 1170390 | - | *-* | *PA14_13610* | ABC transporter permease | 63 | 48 | 61 | 51 | 64 | 60 | 1 |
| 1100 | - | 1171554 | 1172828 | + | *nhaP* | *PA14_13620* | Na+/H+ antiporter NhaP | 41 | 38 | 38 | 40 | 36 | 35 | 1 |
| 1101 | - | 1173132 | 1172887 | - | *-* | *PA14_13630* | hypothetical protein | 43 | 51 | 54 | 52 | 55 | 53 | 1 |
| 1102 | - | 1174062 | 1173163 | - | *-* | *PA14_13650* | hypothetical protein | 31 | 30 | 26 | 24 | 26 | 25 | 1 |
| 1103 | - | 1174823 | 1174167 | - | *-* | *PA14_13660* | hypothetical protein | 3 | 6 | 6 | 4 | 4 | 4 | 1 |
| 1104 | - | 1175304 | 1174918 | - | *-* | *PA14_13670* | hypothetical protein | 9 | 9 | 11 | 9 | 9 | 8 | 1 |
| 1105 | - | 1176155 | 1175325 | - | *-* | *PA14_13680* | short chain dehydrogenase | 29 | 23 | 30 | 26 | 28 | 29 | 1 |
| 1106 | - | 1176992 | 1176243 | - | *-* | *PA14_13690* | methyltransferase | 35 | 43 | 31 | 38 | 43 | 39 | 1 |
| 1107 | - | 1177472 | 1177008 | - | *-* | *PA14_13710* | hypothetical protein | 33 | 32 | 31 | 32 | 38 | 37 | 1 |
| 1108 | - | 1178033 | 1177638 | - | *-* | *PA14_13720* | hypothetical protein | 128 | 135 | 125 | 168 | 223 | 172 | 1 |
| 1109 | - | 1178777 | 1178118 | - | *narL* | *PA14_13730* | transcriptional regulator NarL | 235 | 203 | 299 | 138 | 148 | 249 | 1 |
| 1110 | - | 1180642 | 1178774 | - | *narX* | *PA14_13740* | two-component sensor NarX | 59 | 50 | 67 | 49 | 50 | 60 | 1 |
| 1111 | - | 1180817 | 1182112 | + | *narK1* | *PA14_13750* | nitrite extrusion protein 1 | 17 | 59 | 18 | 80 | 79 | 316 | 0.000376823 |
| 1112 | - | 1182125 | 1183531 | + | *narK2* | *PA14_13770* | nitrite extrusion protein 2 | 10 | 49 | 13 | 17 | 67 | 102 | 1.71783E-09 |
| 1113 | - | 1183607 | 1187392 | + | *narG* | *PA14_13780* | respiratory nitrate reductase alpha subun | 21 | 60 | 23 | 19 | 82 | 50 | 0.066717397 |
| 1114 | - | 1187404 | 1188945 | + | *narH* | *PA14_13800* | respiratory nitrate reductase beta subuni | 25 | 76 | 27 | 18 | 98 | 35 | 0.016650042 |
| 1115 | - | 1188951 | 1189691 | + | *narJ* | *PA14_13810* | respiratory nitrate reductase delta chain | 13 | 51 | 12 | 8 | 51 | 15 | 2.63546E-06 |
| 1116 | - | 1189694 | 1190377 | + | *narI* | *PA14_13830* | respiratory nitrate reductase gamma chain | 15 | 49 | 12 | 8 | 51 | 15 | 0.000417016 |
| 1117 | - | 1190432 | 1191250 | + | *-* | *PA14_13840* | peptidyl-prolyl cis-trans isomerase, PpiC-type | 19 | 54 | 20 | 13 | 54 | 22 | 0.006997432 |
| 1118 | - | 1191307 | 1192296 | + | *moaA* | *PA14_13850* | molybdenum cofactor biosynthesis protein A | 20 | 34 | 17 | 15 | 35 | 17 | 0.65533104 |
| 1119 | - | 1192482 | 1192303 | - | *-* | *PA14_13860* | hypothetical protein | 14 | 19 | 13 | 14 | 19 | 13 | 1 |
| 1120 | - | 1193000 | 1192617 | - | *-* | *PA14_13870* | hypothetical protein | 13 | 13 | 16 | 19 | 10 | 12 | 1 |
| 1121 | - | 1193138 | 1193043 | - | *-* | *PA14_13880* | hypothetical protein | 7 | 8 | 7 | 8 | 6 | 4 | 1 |
| 1122 | - | 1194201 | 1193191 | - | *-* | *PA14_13890* | integrase | 8 | 8 | 6 | 6 | 7 | 6 | 1 |
| 1123 | - | 1195465 | 1195623 | + | *-* | *PA14_13920* | hypothetical protein | 5 | 5 | 7 | 5 | 4 | 7 | 1 |
| 1124 | - | 1196245 | 1197924 | + | *-* | *PA14_13940* | S-type pyocin protein | 70 | 90 | 66 | 73 | 91 | 74 | 1 |
| 1125 | - | 1197924 | 1198262 | + | *-* | *PA14_13950* | hypothetical protein | 112 | 133 | 98 | 74 | 130 | 100 | 1 |
| 1126 | - | 1198426 | 1198334 | - | *-* | *PA14_13960* | hypothetical protein | 96 | 90 | 86 | 75 | 108 | 80 | 1 |
| 1127 | - | 1198694 | 1198431 | - | *-* | *PA14_13970* | hypothetical protein | 43 | 36 | 38 | 43 | 46 | 43 | 1 |
| 1128 | - | 1199242 | 1200033 | + | *-* | *PA14_13990* | amino acid ABC transporter | 35 | 43 | 30 | 90 | 40 | 42 | 1 |
| 1129 | - | 1200689 | 1200057 | - | *-* | *PA14_14000* | hypothetical protein | 27 | 29 | 27 | 59 | 25 | 26 | 1 |
| 1130 | - | 1201879 | 1200752 | - | *-* | *PA14_14010* | amino acid oxidase | 19 | 23 | 18 | 43 | 20 | 18 | 1 |
| 1131 | - | 1202849 | 1201902 | - | *-* | *PA14_14020* | hypothetical protein | 26 | 29 | 25 | 48 | 26 | 25 | 1 |
| 1132 | - | 1204533 | 1203010 | - | *rhl* | *PA14_14040* | ATP-dependent RNA helicase RhlB | 103 | 97 | 94 | 95 | 88 | 84 | 1 |
| 1133 | - | 1204817 | 1206715 | + | *-* | *PA14_14060* | AMP-binding protein | 51 | 69 | 53 | 64 | 62 | 57 | 1 |
| 1134 | - | 1207378 | 1206731 | - | *-* | *PA14_14080* | carboxylesterase | 62 | 74 | 64 | 69 | 74 | 62 | 1 |
| 1135 | - | 1207659 | 1208684 | + | *-* | *PA14_14100* | amino acid-binding protein | 178 | 147 | 211 | 248 | 167 | 220 | 1 |
| 1136 | - | 1208798 | 1209514 | + | *pcs* | *PA14_14110* | phosphatidylserine synthase | 108 | 80 | 107 | 75 | 88 | 92 | 1 |
| 1137 | - | 1209585 | 1210067 | + | *-* | *PA14_14130* | hypothetical protein | 68 | 55 | 66 | 66 | 67 | 69 | 1 |
| 1138 | - | 1210134 | 1210835 | + | *-* | *PA14_14140* | hypothetical protein | 50 | 46 | 50 | 52 | 46 | 47 | 1 |
| 1139 | - | 1210828 | 1211142 | + | *-* | *PA14_14150* | GIY-YIG nuclease superfamily protein | 64 | 56 | 72 | 72 | 63 | 63 | 1 |
| 1140 | - | 1211171 | 1211860 | + | *-* | *PA14_14160* | acetyltransferase | 53 | 51 | 49 | 60 | 60 | 59 | 1 |
| 1141 | - | 1211981 | 1212916 | + | *-* | *PA14_14170* | hypothetical protein | 73 | 75 | 75 | 64 | 73 | 67 | 1 |
| 1142 | - | 1212920 | 1213672 | + | *-* | *PA14_14200* | hypothetical protein | 54 | 54 | 53 | 55 | 59 | 57 | 1 |
| 1143 | - | 1213761 | 1214672 | + | *-* | *PA14_14210* | hypothetical protein | 33 | 28 | 26 | 30 | 34 | 25 | 1 |
| 1144 | - | 1215719 | 1214715 | - | *-* | *PA14_14220* | nucleoid-associated protein NdpA | 83 | 78 | 81 | 86 | 85 | 86 | 1 |
| 1145 | - | 1217167 | 1215812 | - | *-* | *PA14_14230* | hypothetical protein | 58 | 60 | 52 | 50 | 61 | 58 | 1 |
| 1146 | - | 1217711 | 1217241 | - | *-* | *PA14_14250* | hypothetical protein | 36 | 34 | 38 | 34 | 32 | 38 | 1 |
| 1147 | - | 1217815 | 1218357 | + | *-* | *PA14_14270* | isochorismatase family hydrolase | 380 | 340 | 403 | 355 | 352 | 459 | 1 |
| 1148 | - | 1219243 | 1218347 | - | *-* | *PA14_14280* | LysR family transcriptional regulator | 80 | 77 | 87 | 88 | 84 | 95 | 1 |
| 1149 | - | 1219367 | 1219978 | + | *-* | *PA14_14290* | hypothetical protein | 28 | 30 | 31 | 34 | 27 | 31 | 1 |
| 1150 | - | 1221262 | 1220135 | - | *-* | *PA14_14300* | zinc-binding oxidoreductase | 11 | 9 | 10 | 11 | 10 | 10 | 1 |
| 1151 | - | 1221495 | 1221193 | - | *-* | *PA14_14310* | transcriptional regulator | 14 | 14 | 15 | 15 | 10 | 10 | 1 |
| 1152 | - | 1221589 | 1222182 | + | *-* | *PA14_14320* | hypothetical protein | 12 | 4 | 6 | 6 | 4 | 13 | 0.001057312 |
| 1153 | - | 1222824 | 1222474 | - | *-* | *PA14_14330* | chaperone | 13 | 17 | 14 | 13 | 11 | 14 | 1 |
| 1154 | - | 1224022 | 1223012 | - | *-* | *PA14_14340* | SAM-dependent methyltransferase | 22 | 20 | 25 | 19 | 21 | 27 | 1 |
| 1155 | - | 1225946 | 1224114 | - | *-* | *PA14_14360* | sodium:sulfate symporter | 98 | 84 | 129 | 52 | 62 | 109 | 1 |
| 1156 | - | 1226859 | 1226065 | - | *-* | *PA14_14370* | ABC-transporter ATP-binding component | 70 | 82 | 54 | 58 | 68 | 56 | 1 |
| 1157 | - | 1227752 | 1226862 | - | *-* | *PA14_14380* | ABC transporter permease | 24 | 30 | 20 | 21 | 25 | 17 | 1 |
| 1158 | - | 1228813 | 1227836 | - | *-* | *PA14_14390* | ABC-type transport protein, periplasmic c | 73 | 122 | 61 | 62 | 86 | 61 | 1 |
| 1159 | - | 1228936 | 1229070 | + | *-* | *PA14_14400* | hypothetical protein | 5 | 2 | 4 | 4 | 3 | 3 | 0.56521933 |
| 1160 | - | 1229163 | 1229642 | + | *-* | *PA14_14420* | hypothetical protein | 14 | 17 | 11 | 12 | 15 | 9 | 1 |
| 1161 | - | 1231207 | 1231584 | + | *-* | *PA14_14430* | hypothetical protein | 36 | 47 | 39 | 43 | 37 | 31 | 1 |
| 1162 | - | 1234433 | 1231581 | - | *valS* | *PA14_14440* | valyl-tRNA synthetase | 76 | 88 | 80 | 79 | 85 | 65 | 1 |
| 1163 | - | 1234922 | 1234554 | - | *-* | *PA14_14450* | hypothetical protein | 41 | 42 | 42 | 37 | 50 | 43 | 1 |
| 1164 | - | 1235362 | 1234934 | - | *holC* | *PA14_14460* | DNA polymerase III subunit chi | 88 | 92 | 76 | 73 | 71 | 68 | 1 |
| 1165 | - | 1236846 | 1235359 | - | *pepA* | *PA14_14470* | leucyl aminopeptidase | 118 | 129 | 103 | 97 | 106 | 93 | 1 |
| 1166 | - | 1237183 | 1237995 | + | *-* | *PA14_14480* | transcriptional regulator | 57 | 44 | 56 | 60 | 48 | 50 | 1 |
| 1167 | - | 1238079 | 1239002 | + | *-* | *PA14_14490* | hydrolase | 29 | 22 | 22 | 27 | 20 | 24 | 1 |
| 1168 | - | 1239195 | 1240313 | + | *-* | *PA14_14500* | hypothetical protein | 71 | 43 | 72 | 60 | 55 | 59 | 1 |
| 1169 | - | 1240306 | 1241373 | + | *-* | *PA14_14510* | hypothetical protein | 90 | 85 | 87 | 97 | 84 | 76 | 1 |
| 1170 | - | 1242002 | 1241505 | - | *-* | *PA14_14520* | hypothetical protein | 26 | 23 | 28 | 25 | 24 | 26 | 1 |
| 1171 | - | 1242132 | 1243703 | + | *-* | *PA14_14530* | hypothetical protein | 45 | 40 | 56 | 64 | 46 | 52 | 1 |
| 1172 | - | 1243940 | 1244128 | + | *-* | *PA14_14540* | hypothetical protein | 325 | 640 | 387 | 515 | 488 | 522 | 0.580848337 |
| 1173 | - | 1244088 | 1244495 | + | *-* | *PA14_14550* | hypothetical protein | 1239 | 2105 | 1599 | 2551 | 2280 | 2463 | 1 |
| 1174 | - | 1244649 | 1244524 | - | *-* | *PA14_14560* | hypothetical protein | 106 | 118 | 110 | 142 | 132 | 103 | 1 |
| 1175 | - | 1244826 | 1244743 | - | *-* | *PA14_14570* | Leu tRNA | 15 | 9 | 10 | 5 | 8 | 8 | 0.760328835 |
| 1176 | - | 1244929 | 1245972 | + | *queA* | *PA14_14590* | S-adenosylmethionine--tRNA ribosyltransferase-isomerase | 54 | 52 | 54 | 56 | 48 | 42 | 1 |

|  | A | B | C | D | E | F | G | H | I | J | K | L | M | N |
| --- | --- | --- | --- | --- | --- | --- | --- | --- | --- | --- | --- | --- | --- | --- |
| 1177 | - | 1245985 | 1247103 | + | *tgt* | *PA14_14600* | queuine tRNA-ribosyltransferase | 64 | 66 | 77 | 60 | 64 | 54 | 1 |
| 1178 | - | 1247147 | 1247485 | + | *yajC* | *PA14_14610* | preprotein translocase subunit YajC | 74 | 95 | 71 | 50 | 56 | 42 | 1 |
| 1179 | - | 1247545 | 1249407 | + | *secD* | *PA14_14630* | preprotein translocase subunit SecD | 99 | 130 | 105 | 90 | 124 | 69 | 1 |
| 1180 | - | 1249418 | 1250338 | + | *secF* | *PA14_14650* | preprotein translocase subunit SecF | 168 | 209 | 157 | 121 | 187 | 105 | 1 |
| 1181 | - | 1250522 | 1251070 | + | *-* | *PA14_14660* | hypothetical protein | 1065 | 1016 | 1310 | 1071 | 1249 | 1239 | 1 |
| 1182 | - | 1251990 | 1251175 | - | *-* | *PA14_14680* | extragenic suppressor protein SuhB | 48 | 49 | 66 | 57 | 42 | 39 | 1 |
| 1183 | - | 1252142 | 1252915 | + | *-* | *PA14_14690* | methyltransferase | 62 | 42 | 52 | 45 | 43 | 49 | 1 |
| 1184 | - | 1252915 | 1253691 | + | *cysE* | *PA14_14700* | serine O-acetyltransferase | 241 | 212 | 215 | 212 | 210 | 200 | 1 |
| 1185 | - | 1253836 | 1254327 | + | *-* | *PA14_14710* | Rrf2 family protein | 56 | 150 | 47 | 223 | 263 | 76 | 0.033575691 |
| 1186 | - | 1254357 | 1255571 | + | *iscS* | *PA14_14730* | cysteine desulfurase | 244 | 367 | 136 | 591 | 674 | 363 | 1 |
| 1187 | - | 1255607 | 1255993 | + | *-* | *PA14_14740* | scaffold protein | 383 | 393 | 209 | 635 | 727 | 554 | 1 |
| 1188 | - | 1255994 | 1256014 | ? | *-* | predicted RNA | - | 412 | 402 | 200 | 735 | 861 | 643 | 1 |
| 1189 | - | 1256021 | 1256344 | + | *-* | *PA14_14750* | iron-binding protein IscA | 423 | 386 | 222 | 719 | 872 | 639 | 1 |
| 1190 | - | 1256352 | 1256873 | + | *hscB* | *PA14_14770* | co-chaperone HscB | 194 | 158 | 115 | 286 | 397 | 267 | 1 |
| 1191 | - | 1256916 | 1258775 | + | *hscA* | *PA14_14780* | chaperone protein HscA | 229 | 155 | 138 | 271 | 370 | 298 | 1 |
| 1192 | - | 1258782 | 1259120 | + | *fdx2* | *PA14_14800* | ferredoxin 2Fe-2S | 251 | 152 | 127 | 260 | 332 | 339 | 1 |
| 1193 | - | 1259146 | 1259346 | + | *-* | *PA14_14810* | hypothetical protein | 382 | 293 | 315 | 379 | 507 | 534 | 1 |
| 1194 | - | 1259590 | 1260021 | + | *ndk* | *PA14_14820* | nucleoside diphosphate kinase | 363 | 458 | 332 | 247 | 247 | 266 | 1 |
| 1195 | - | 1260046 | 1261185 | + | *-* | *PA14_14830* | hypothetical protein | 283 | 256 | 254 | 240 | 225 | 220 | 1 |
| 1196 | - | 1261203 | 1261961 | + | *pilF* | *PA14_14850* | type 4 fimbrial biogenesis protein PilF | 216 | 200 | 224 | 270 | 233 | 234 | 1 |
| 1197 | - | 1261958 | 1263001 | + | *-* | *PA14_14860* | hypothetical protein | 232 | 224 | 227 | 251 | 230 | 234 | 1 |
| 1198 | - | 1262998 | 1264113 | + | *ispG* | *PA14_14880* | 4-hydroxy-3-methylbut-2-en-1-yl diphosphate synthase | 414 | 372 | 401 | 392 | 363 | 394 | 1 |
| 1199 | - | 1264132 | 1265421 | + | *hisS* | *PA14_14890* | histidyl-tRNA synthetase | 345 | 324 | 345 | 321 | 318 | 345 | 1 |
| 1200 | - | 1265448 | 1266092 | + | *-* | *PA14_14900* | hypothetical protein | 580 | 528 | 570 | 558 | 572 | 573 | 1 |
| 1201 | - | 1266094 | 1267236 | + | *-* | *PA14_14910* | hypothetical protein | 480 | 441 | 455 | 459 | 448 | 454 | 1 |
| 1202 | - | 1267314 | 1268795 | + | *engA* | *PA14_14930* | GTP-binding protein EngA | 170 | 149 | 185 | 178 | 167 | 184 | 1 |
| 1203 | - | 1269092 | 1270240 | + | *-* | *PA14_14940* | aminotransferase | 30 | 28 | 29 | 25 | 32 | 25 | 1 |
| 1204 | - | 1270228 | 1271022 | + | *-* | *PA14_14960* | hypothetical protein | 164 | 163 | 151 | 154 | 147 | 156 | 1 |
| 1205 | - | 1271156 | 1271743 | + | *-* | *PA14_14975* | hypothetical protein | 331 | 284 | 357 | 401 | 365 | 385 | 1 |
| 1206 | - | 1272782 | 1271832 | - | *-* | *PA14_14990* | oxidoreductase | 84 | 82 | 82 | 78 | 84 | 74 | 1 |
| 1207 | - | 1272963 | 1273418 | + | *-* | *PA14_15000* | hypothetical protein | 38 | 36 | 39 | 45 | 36 | 39 | 1 |
| 1208 | - | 1273472 | 1273804 | + | *-* | *PA14_15020* | hypothetical protein | 27 | 31 | 34 | 26 | 26 | 23 | 1 |
| 1209 | - | 1275652 | 1273874 | - | *leuA* | *PA14_15030* | 2-isopropylmalate synthase | 104 | 109 | 92 | 86 | 95 | 97 | 1 |
| 1210 | - | 1276025 | 1276486 | + | *-* | *PA14_15050* | hypothetical protein | 230 | 208 | 268 | 288 | 230 | 185 | 1 |
| 1211 | - | 1276607 | 1278778 | + | *oprC* | *PA14_15070* | outer membrane copper receptor OprC | 192 | 234 | 171 | 219 | 230 | 166 | 1 |
| 1212 | - | 1278843 | 1280258 | + | *-* | *PA14_15080* | hypothetical protein | 263 | 240 | 251 | 277 | 275 | 245 | 1 |
| 1213 | - | 1280671 | 1280276 | - | *-* | *PA14_15090* | hypothetical protein | 178 | 192 | 188 | 195 | 200 | 192 | 1 |
| 1214 | - | 1281606 | 1280758 | - | *-* | *PA14_15100* | hypothetical protein | 82 | 85 | 107 | 111 | 95 | 91 | 1 |
| 1215 | - | 1281778 | 1282167 | + | *-* | *PA14_15110* | hypothetical protein | 222 | 203 | 218 | 235 | 202 | 244 | 1 |
| 1216 | - | 1282296 | 1282772 | + | *-* | *PA14_15120* | hypothetical protein | 154 | 112 | 111 | 180 | 148 | 202 | 1 |
| 1217 | - | 1282829 | 1283401 | + | *-* | *PA14_15130* | hypothetical protein | 291 | 267 | 287 | 447 | 337 | 412 | 1 |
| 1218 | - | 1284065 | 1283436 | - | *-* | *PA14_15140* | hypothetical protein | 301 | 208 | 241 | 405 | 330 | 389 | 1 |
| 1219 | - | 1284186 | 1285139 | + | *-* | *PA14_15150* | transcriptional regulator | 70 | 62 | 70 | 79 | 65 | 67 | 1 |
| 1220 | - | 1285843 | 1285136 | - | *-* | *PA14_15160* | hypothetical protein | 27 | 24 | 23 | 20 | 22 | 24 | 1 |
| 1221 | - | 1287182 | 1285902 | - | *-* | *PA14_15180* | transporter | 17 | 15 | 19 | 18 | 19 | 19 | 1 |
| 1222 | - | 1287673 | 1287179 | - | *-* | *PA14_15190* | hypothetical protein | 15 | 12 | 12 | 13 | 14 | 14 | 1 |
| 1223 | - | 1288786 | 1287746 | - | *-* | *PA14_15200* | hypothetical protein | 12 | 13 | 11 | 11 | 13 | 10 | 1 |
| 1224 | - | 1289873 | 1288938 | - | *-* | *PA14_15210* | LysR family transcriptional regulator | 32 | 30 | 29 | 27 | 22 | 22 | 1 |
| 1225 | - | 1291259 | 1289880 | - | *xseA* | *PA14_15230* | exodeoxyribonuclease VII large subunit | 54 | 47 | 48 | 43 | 41 | 37 | 1 |
| 1226 | - | 1292204 | 1291296 | - | *-* | *PA14_15240* | LysR family transcriptional regulator | 6 | 6 | 6 | 6 | 5 | 5 | 1 |
| 1227 | - | 1292312 | 1293094 | + | *-* | *PA14_15250* | hypothetical protein | 8 | 7 | 7 | 7 | 8 | 7 | 1 |
| 1228 | - | 1294221 | 1293079 | - | *-* | *PA14_15260* | acetylpolyamine aminohydrolase | 3 | 3 | 3 | 2 | 2 | 2 | 1 |
| 1229 | - | 1295408 | 1294251 | - | *-* | *PA14_15270* | hypothetical protein | 3 | 3 | 4 | 4 | 3 | 3 | 1 |
| 1230 | - | 1296317 | 1295421 | - | *-* | *PA14_15280* | hypothetical protein | 9 | 7 | 6 | 10 | 6 | 5 | 1 |
| 1231 | - | 1297429 | 1296452 | - | *-* | *PA14_15290* | transcriptional regulator | 20 | 20 | 21 | 24 | 17 | 22 | 1 |
| 1232 | - | 1297693 | 1299162 | + | *guaB* | *PA14_15310* | inosine 5'-monophosphate dehydrogenase | 192 | 234 | 241 | 175 | 179 | 128 | 1 |
| 1233 | - | 1299242 | 1300819 | + | *guaA* | *PA14_15340* | GMP synthase | 182 | 206 | 195 | 176 | 185 | 123 | 1 |
| 1234 | - | 1301126 | 1302328 | + | *-* | *PA14_15350* | integrase | 152 | 163 | 158 | 147 | 128 | 127 | 1 |
| 1235 | - | 1303723 | 1302743 | - | *-* | *PA14_15360* | hypothetical protein | 173 | 162 | 161 | 136 | 153 | 161 | 1 |
| 1236 | - | 1304052 | 1304930 | + | *repA* | *PA14_15380* | replicative helicase, RepA | 8 | 5 | 9 | 7 | 6 | 7 | 0.791445601 |
| 1237 | - | 1304084 | 1303857 | - | *-* | *PA14_15370* | hypothetical protein | 21 | 29 | 21 | 23 | 22 | 19 | 1 |
| 1238 | - | 1304902 | 1305789 | + | *-* | *PA14_15400* | replication protein, RepC | 25 | 21 | 25 | 27 | 18 | 22 | 1 |
| 1239 | - | 1306970 | 1306410 | - | *-* | *PA14_15430* | resolvase, essential for transposition | 17 | 17 | 17 | 17 | 11 | 14 | 1 |
| 1240 | - | 1308090 | 1307101 | - | *-* | *PA14_15435* | hypothetical protein | 37 | 38 | 33 | 37 | 35 | 35 | 1 |
| 1241 | - | 1308323 | 1308087 | - | *merE* | *PA14_15445* | mercury resistance protein | 38 | 36 | 32 | 39 | 33 | 35 | 1 |
| 1242 | - | 1308685 | 1308320 | - | *merD* | *PA14_15450* | transcriptional regulator MerD | 43 | 48 | 41 | 50 | 36 | 40 | 1 |
| 1243 | - | 1310388 | 1308703 | - | *merA* | *PA14_15460* | mercuric reductase | 46 | 50 | 42 | 50 | 40 | 40 | 1 |
| 1244 | - | 1310735 | 1310460 | - | *merP* | *PA14_15470* | periplasmic mercuric ion binding protein, MerP | 75 | 79 | 66 | 80 | 59 | 53 | 1 |
| 1245 | - | 1311098 | 1310748 | - | *merT* | *PA14_15475* | mercuric transport protein | 33 | 42 | 30 | 42 | 32 | 29 | 1 |
| 1246 | - | 1311170 | 1311604 | + | *merR* | *PA14_15480* | transcriptional regulator MerR | 168 | 195 | 167 | 151 | 126 | 113 | 1 |
| 1247 | - | 1311858 | 1312034 | + | *-* | *PA14_15490* | hypothetical protein | 12 | 12 | 13 | 12 | 10 | 10 | 1 |
| 1248 | - | 1312074 | 1312361 | + | *-* | *PA14_15500* | oriT-binding protein, TraK | 27 | 23 | 21 | 19 | 19 | 27 | 1 |
| 1249 | - | 1312655 | 1313026 | + | *traJ* | *PA14_15510* | conjugal transfer relaxosome component TraJ | 23 | 22 | 24 | 27 | 19 | 23 | 1 |
| 1250 | - | 1313291 | 1314076 | + | *trbJ* | *PA14_15520* | conjugal transfer protein TrbJ | 16 | 15 | 17 | 17 | 14 | 11 | 1 |
| 1251 | - | 1314094 | 1314369 | + | *-* | *PA14_15530* | entry/exclusion protein TrbK | 42 | 27 | 48 | 15 | 11 | 23 | 0.615940863 |
| 1252 | - | 1314366 | 1315988 | + | *-* | *PA14_15540* | mating pair formation protein TrbL | 45 | 35 | 51 | 27 | 24 | 35 | 1 |
| 1253 | - | 1316812 | 1316504 | - | *-* | *PA14_15560* | hypothetical protein | 89 | 100 | 84 | 74 | 70 | 64 | 1 |
| 1254 | - | 1317148 | 1317348 | + | *-* | *PA14_15570* | hypothetical protein | 66 | 79 | 82 | 68 | 81 | 61 | 1 |
| 1255 | - | 1317407 | 1320187 | + | *-* | *PA14_15580* | Type II restriction enzyme, methylase subunit | 133 | 140 | 123 | 143 | 149 | 126 | 1 |
| 1256 | - | 1321218 | 1323335 | + | *-* | *PA14_15590* | hypothetical protein | 196 | 223 | 178 | 203 | 205 | 199 | 1 |
| 1257 | - | 1323328 | 1324512 | + | *-* | *PA14_15600* | hypothetical protein | 252 | 276 | 230 | 265 | 269 | 268 | 1 |
| 1258 | - | 1324805 | 1327411 | + | *-* | *PA14_15610* | hypothetical protein | 234 | 253 | 221 | 240 | 226 | 248 | 1 |
| 1259 | - | 1328012 | 1328974 | + | *-* | *PA14_15620* | oxidoreductase | 9 | 9 | 9 | 8 | 7 | 5 | 1 |
| 1260 | - | 1328895 | 1330193 | + | *-* | *PA14_15630* | hypothetical protein | 17 | 17 | 20 | 21 | 18 | 16 | 1 |

|  | A | B | C | D | E | F | G | H | I | J | K | L | M | N |
| --- | --- | --- | --- | --- | --- | --- | --- | --- | --- | --- | --- | --- | --- | --- |
| 1261 | - | 1331201 | 1330245 | - | *-* | *PA14_15650* | hypothetical protein | 31 | 27 | 26 | 27 | 22 | 23 | 1 |
| 1262 | - | 1331350 | 1332345 | + | *-* | *PA14_15660* | oxidoreductase | 21 | 18 | 20 | 18 | 15 | 16 | 1 |
| 1263 | - | 1332509 | 1333900 | + | *-* | *PA14_15670* | metallo-oxidoreductase | 59 | 69 | 76 | 70 | 88 | 71 | 1 |
| 1264 | - | 1333891 | 1334439 | + | *-* | *PA14_15680* | hypothetical protein | 64 | 84 | 94 | 109 | 105 | 94 | 1 |
| 1265 | - | 1334828 | 1336084 | + | *-* | *PA14_15700* | amino acid permease | 14 | 11 | 13 | 11 | 11 | 10 | 1 |
| 1266 | - | 1336210 | 1336794 | + | *-* | *PA14_15710* | hypothetical protein | 39 | 43 | 43 | 35 | 30 | 31 | 1 |
| 1267 | - | 1338163 | 1336808 | - | *-* | *PA14_15720* | transglycosylase | 51 | 47 | 52 | 52 | 47 | 47 | 1 |
| 1268 | - | 1338564 | 1342460 | + | *purL* | *PA14_15740* | phosphoribosylformylglycinamidine synthase | 147 | 141 | 140 | 134 | 135 | 112 | 1 |
| 1269 | - | 1342467 | 1342745 | + | *-* | *PA14_15750* | hypothetical protein | 142 | 181 | 236 | 230 | 198 | 229 | 1 |
| 1270 | - | 1342925 | 1343242 | + | *-* | *PA14_15770* | hypothetical protein | 175 | 172 | 213 | 175 | 137 | 139 | 1 |
| 1271 | - | 1345156 | 1343444 | - | *-* | *PA14_15780* | PTS system N-acetylglucosamine-specific IIBC component | 30 | 30 | 30 | 26 | 29 | 31 | 1 |
| 1272 | - | 1347709 | 1345181 | - | *-* | *PA14_15790* | phosphoenolpyruvate-protein phosphotransferase | 61 | 54 | 56 | 55 | 54 | 52 | 1 |
| 1273 | - | 1348748 | 1347726 | - | *-* | *PA14_15810* | aminotransferase | 20 | 25 | 20 | 19 | 23 | 20 | 1 |
| 1274 | - | 1349836 | 1348745 | - | *-* | *PA14_15820* | N-acetylglucosamine-6-phosphate deacetylase | 11 | 16 | 12 | 13 | 14 | 13 | 1 |
| 1275 | - | 1350596 | 1349853 | - | *-* | *PA14_15830* | GntR family transcriptional regulator | 9 | 9 | 10 | 11 | 10 | 8 | 1 |
| 1276 | - | 1351339 | 1350839 | - | *-* | *PA14_15840* | hypothetical protein | 69 | 59 | 79 | 56 | 56 | 62 | 1 |
| 1277 | - | 1351956 | 1351402 | - | *-* | *PA14_15850* | hypothetical protein | 43 | 40 | 48 | 36 | 39 | 43 | 1 |
| 1278 | - | 1352097 | 1352708 | + | *-* | *PA14_15860* | hypothetical protein | 109 | 112 | 119 | 85 | 109 | 105 | 1 |
| 1279 | - | 1352746 | 1353270 | + | *-* | *PA14_15870* | hypothetical protein | 462 | 435 | 463 | 496 | 449 | 506 | 1 |
| 1280 | - | 1353267 | 1353461 | + | *-* | *PA14_15880* | hypothetical protein | 155 | 154 | 141 | 141 | 156 | 128 | 1 |
| 1281 | - | 1353499 | 1354680 | + | *purT* | *PA14_15890* | phosphoribosylglycinamide formyltransferase 2 | 127 | 125 | 108 | 103 | 117 | 111 | 1 |
| 1282 | - | 1354682 | 1355431 | + | *-* | *PA14_15910* | hypothetical protein | 221 | 174 | 210 | 210 | 214 | 207 | 1 |
| 1283 | - | 1356752 | 1355439 | - | *-* | *PA14_15920* | major facilitator transporter | 13 | 12 | 15 | 15 | 14 | 13 | 1 |
| 1284 | - | 1358348 | 1357056 | - | *-* | *PA14_15930* | hemolysin | 35 | 33 | 37 | 43 | 36 | 35 | 1 |
| 1285 | - | 1359161 | 1358361 | - | *-* | *PA14_15940* | hypothetical protein | 41 | 30 | 43 | 36 | 30 | 34 | 1 |
| 1286 | - | 1359462 | 1360835 | + | *ffh* | *PA14_15960* | signal recognition particle protein Ffh | 132 | 125 | 146 | 120 | 109 | 97 | 1 |
| 1287 | - | 1361045 | 1361296 | + | *rpsP* | *PA14_15970* | 30S ribosomal protein S16 | 548 | 746 | 775 | 573 | 490 | 343 | 1 |
| 1288 | - | 1361312 | 1361839 | + | *rimM* | *PA14_15980* | 16S rRNA-processing protein RimM | 528 | 630 | 702 | 541 | 431 | 311 | 1 |
| 1289 | - | 1361846 | 1362604 | + | *trmD* | *PA14_15990* | tRNA (guanine-N(1)-)-methyltransferase | 272 | 319 | 393 | 300 | 266 | 199 | 1 |
| 1290 | - | 1362646 | 1362996 | + | *rplS* | *PA14_16000* | 50S ribosomal protein L19 | 322 | 453 | 317 | 183 | 256 | 185 | 1 |
| 1291 | - | 1363148 | 1363573 | + | *-* | *PA14_16010* | hypothetical protein | 40 | 45 | 48 | 45 | 40 | 39 | 1 |
| 1292 | - | 1364329 | 1363643 | - | *-* | *PA14_16020* | hypothetical protein | 64 | 117 | 89 | 68 | 77 | 70 | 0.641536103 |
| 1293 | - | 1366277 | 1364442 | - | *-* | *PA14_16030* | sodium/hydrogen antiporter | 35 | 30 | 34 | 35 | 33 | 31 | 1 |
| 1294 | - | 1366492 | 1367388 | + | *xerD* | *PA14_16040* | site-specific tyrosine recombinase XerD | 33 | 23 | 26 | 22 | 23 | 19 | 1 |
| 1295 | - | 1367515 | 1368243 | + | *dsbC* | *PA14_16050* | thiol:disulfide interchange protein DsbC | 110 | 108 | 115 | 91 | 118 | 93 | 1 |
| 1296 | - | 1368457 | 1369761 | + | *hom* | *PA14_16070* | homoserine dehydrogenase | 123 | 122 | 121 | 106 | 124 | 104 | 1 |
| 1297 | - | 1369814 | 1371223 | + | *thrC* | *PA14_16090* | threonine synthase | 96 | 114 | 84 | 80 | 89 | 83 | 1 |
| 1298 | 1372370 | 1372497 | 1371277 | - | *-* | *PA14_16100* | hypothetical protein | 55 | 36 | 35 | 38 | 31 | 50 | 1 |
| 1299 | 1372370 | 1372594 | 1372878 | + | *-* | *PA14_16110* | hypothetical protein | 50 | 38 | 44 | 68 | 47 | 69 | 1 |
| 1300 | - | 1374294 | 1373065 | - | *-* | *PA14_16130* | hypothetical protein | 51 | 41 | 48 | 50 | 39 | 49 | 1 |
| 1301 | - | 1374776 | 1375219 | + | *-* | *PA14_16140* | hypothetical protein | 112 | 104 | 94 | 84 | 122 | 84 | 1 |
| 1302 | - | 1375234 | 1375929 | + | *-* | *PA14_16150* | hypothetical protein | 110 | 104 | 79 | 78 | 106 | 81 | 1 |
| 1303 | - | 1375985 | 1376626 | + | *-* | *PA14_16160* | hypothetical protein | 34 | 29 | 23 | 20 | 32 | 19 | 1 |
| 1304 | - | 1376662 | 1378728 | + | *-* | *PA14_16180* | hypothetical protein | 83 | 68 | 58 | 48 | 73 | 57 | 1 |
| 1305 | - | 1378878 | 1384118 | + | *-* | *PA14_16190* | hypothetical protein | 65 | 49 | 43 | 39 | 54 | 45 | 1 |
| 1306 | - | 1384137 | 1384829 | + | *-* | *PA14_16200* | hypothetical protein | 47 | 38 | 30 | 26 | 41 | 37 | 1 |
| 1307 | - | 1384936 | 1385475 | + | *-* | *PA14_16210* | hypothetical protein | 19 | 13 | 21 | 16 | 16 | 14 | 1 |
| 1308 | - | 1385519 | 1387234 | + | *recJ* | *PA14_16220* | single-stranded-DNA-specific exonuclease RecJ | 28 | 24 | 27 | 27 | 27 | 25 | 1 |
| 1309 | 1387361 | 1387521 | 1389017 | + | *lasB* | *PA14_16250* | elastase LasB | 583 | 241 | 181 | 863 | 566 | 1089 | 1 |
| 1310 | - | 1389187 | 1390293 | + | *-* | *PA14_16260* | FMN oxidoreductase | 247 | 252 | 331 | 248 | 283 | 314 | 1 |
| 1311 | - | 1390481 | 1390831 | + | *-* | *PA14_16270* | hypothetical protein | 110 | 88 | 102 | 65 | 72 | 74 | 1 |
| 1312 | - | 1391485 | 1390844 | - | *-* | *PA14_16280* | transcriptional regulator | 291 | 232 | 252 | 121 | 208 | 209 | 1 |
| 1313 | - | 1391688 | 1392113 | + | *-* | *PA14_16290* | hypothetical protein | 272 | 85 | 176 | 88 | 99 | 86 | 0.012540033 |
| 1314 | - | 1392121 | 1392282 | + | *-* | *PA14_16300* | hypothetical protein | 137 | 50 | 95 | 54 | 59 | 46 | 0.000861338 |
| 1315 | - | 1392430 | 1393671 | + | *-* | *PA14_16310* | MFS permease | 34 | 9 | 14 | 10 | 7 | 31 | 2.90451E-05 |
| 1316 | - | 1394099 | 1393758 | - | *-* | *PA14_16320* | peptidyl-prolyl cis-trans isomerase, FkbP-type | 23 | 35 | 21 | 24 | 29 | 22 | 1 |
| 1317 | - | 1394310 | 1396016 | + | *-* | *PA14_16330* | hypothetical protein | 61 | 73 | 64 | 56 | 61 | 52 | 1 |
| 1318 | - | 1396898 | 1396095 | - | *-* | *PA14_16340* | hypothetical protein | 31 | 29 | 29 | 24 | 24 | 25 | 1 |
| 1319 | - | 1397660 | 1397019 | - | *-* | *PA14_16350* | two-component response regulator | 9 | 7 | 10 | 11 | 8 | 7 | 1 |
| 1320 | - | 1399846 | 1397984 | - | *-* | *PA14_16360* | hypothetical protein | 19 | 19 | 20 | 20 | 18 | 19 | 1 |
| 1321 | - | 1400168 | 1400863 | + | *-* | *PA14_16370* | hypothetical protein | 193 | 237 | 205 | 162 | 211 | 172 | 1 |
| 1322 | - | 1401805 | 1400900 | - | *-* | *PA14_16380* | LysR family transcriptional regulator | 78 | 93 | 87 | 80 | 76 | 81 | 1 |
| 1323 | - | 1401977 | 1403650 | + | *-* | *PA14_16390* | GMC-type oxidoreductase | 64 | 100 | 84 | 137 | 84 | 96 | 1 |
| 1324 | - | 1403759 | 1405378 | + | *-* | *PA14_16410* | MFS transporter | 34 | 51 | 40 | 61 | 40 | 45 | 1 |
| 1325 | - | 1405645 | 1407273 | + | *wspA* | *PA14_16430* | chemotaxis transducer | 232 | 233 | 271 | 300 | 253 | 269 | 1 |
| 1326 | - | 1407278 | 1407793 | + | *-* | *PA14_16440* | hypothetical protein | 232 | 258 | 270 | 288 | 250 | 269 | 1 |
| 1327 | - | 1407790 | 1409058 | + | *wspC* | *PA14_16450* | methyltransferase | 145 | 136 | 149 | 160 | 136 | 155 | 1 |
| 1328 | - | 1409051 | 1409740 | + | *wspD* | *PA14_16460* | CheW domain-containing protein | 84 | 92 | 84 | 111 | 93 | 97 | 1 |
| 1329 | - | 1409737 | 1412046 | + | *-* | *PA14_16470* | chemotaxis sensor/effector fusion protein | 175 | 171 | 186 | 202 | 180 | 183 | 1 |
| 1330 | - | 1412043 | 1413050 | + | *wspF* | *PA14_16480* | chemotaxis-specific methylesterase | 207 | 195 | 239 | 238 | 218 | 239 | 1 |
| 1331 | - | 1413158 | 1414201 | + | *wspR* | *PA14_16500* | two-component response regulator | 177 | 176 | 189 | 217 | 171 | 218 | 1 |
| 1332 | - | 1414421 | 1415419 | + | *-* | *PA14_16510* | peptide chain release factor 2 | 131 | 135 | 147 | 145 | 127 | 133 | 1 |
| 1333 | - | 1415600 | 1417105 | + | *lysS* | *PA14_16530* | lysyl-tRNA synthetase | 62 | 84 | 74 | 56 | 66 | 50 | 1 |
| 1334 | - | 1417317 | 1418030 | + | *-* | *PA14_16550* | TetR family transcriptional regulator | 43 | 41 | 40 | 39 | 38 | 31 | 1 |
| 1335 | - | 1418127 | 1418678 | + | *-* | *PA14_16560* | lipoprotein | 162 | 127 | 156 | 178 | 144 | 156 | 1 |
| 1336 | - | 1418687 | 1419982 | + | *-* | *PA14_16580* | hypothetical protein | 94 | 103 | 91 | 116 | 117 | 112 | 1 |
| 1337 | - | 1420051 | 1420797 | + | *-* | *PA14_16590* | hypothetical protein | 140 | 135 | 119 | 113 | 129 | 125 | 1 |
| 1338 | - | 1420794 | 1421699 | + | *-* | *PA14_16600* | alpha/beta hydrolase | 123 | 123 | 119 | 111 | 133 | 117 | 1 |
| 1339 | - | 1421696 | 1422019 | + | *-* | *PA14_16610* | hypothetical protein | 123 | 136 | 111 | 126 | 114 | 126 | 1 |
| 1340 | - | 1422016 | 1422537 | + | *-* | *PA14_16620* | hypothetical protein | 140 | 127 | 144 | 129 | 157 | 130 | 1 |
| 1341 | - | 1423398 | 1422613 | - | *-* | *PA14_16630* | outer membrane protein, OmpA | 873 | 1210 | 912 | 806 | 1347 | 1018 | 1 |
| 1342 | - | 1423851 | 1423447 | - | *-* | *PA14_16640* | lipoprotein | 890 | 1240 | 1126 | 832 | 1318 | 1058 | 1 |
| 1343 | - | 1426481 | 1424253 | - | *-* | *PA14_16660* | metal-transporting P-type ATPase | 77 | 78 | 53 | 61 | 56 | 73 | 1 |
| 1344 | - | 1426595 | 1427065 | + | *-* | *PA14_16670* | transcriptional regulator CadR | 230 | 276 | 257 | 275 | 287 | 301 | 1 |

|  | A | B | C | D | E | F | G | H | I | J | K | L | M | N |
| --- | --- | --- | --- | --- | --- | --- | --- | --- | --- | --- | --- | --- | --- | --- |
| 1345 | - | 1427418 | 1427074 | - | *-* | *PA14_16680* | hypothetical protein | 378 | 460 | 497 | 447 | 427 | 607 | 1 |
| 1346 | - | 1427626 | 1430262 | + | *ppc* | *PA14_16690* | phosphoenolpyruvate carboxylase | 182 | 156 | 147 | 174 | 160 | 185 | 1 |
| 1347 | - | 1430423 | 1431070 | + | *adk* | *PA14_16700* | adenylate kinase | 206 | 188 | 189 | 158 | 168 | 168 | 1 |
| 1348 | - | 1431174 | 1431854 | + | *-* | *PA14_16710* | hypothetical protein | 36 | 39 | 43 | 31 | 34 | 29 | 1 |
| 1349 | - | 1431928 | 1432275 | + | *-* | *PA14_16720* | hypothetical protein | 77 | 70 | 107 | 70 | 68 | 75 | 1 |
| 1350 | - | 1432294 | 1433160 | + | *-* | *PA14_16730* | hypothetical protein | 40 | 26 | 44 | 30 | 33 | 33 | 1 |
| 1351 | - | 1433233 | 1434057 | + | *-* | *PA14_16740* | hypothetical protein | 24 | 27 | 24 | 26 | 22 | 24 | 1 |
| 1352 | - | 1434062 | 1434757 | + | *-* | *PA14_16750* | hypothetical protein | 16 | 18 | 18 | 22 | 17 | 19 | 1 |
| 1353 | - | 1434813 | 1435598 | + | *-* | *PA14_16770* | hypothetical protein | 34 | 30 | 38 | 29 | 27 | 24 | 1 |
| 1354 | - | 1435627 | 1436904 | + | *-* | *PA14_16780* | hypothetical protein | 47 | 97 | 69 | 71 | 78 | 51 | 0.490513327 |
| 1355 | - | 1437549 | 1436911 | - | *-* | *PA14_16790* | TetR family transcriptional regulator | 42 | 262 | 162 | 170 | 214 | 130 | 6.13255E-17 |
| 1356 | - | 1437645 | 1438748 | + | *-* | *PA14_16800* | efflux transmembrane protein | 15 | 186 | 92 | 94 | 94 | 43 | 2.97801E-94 |
| 1357 | - | 1438753 | 1441830 | + | *-* | *PA14_16820* | efflux transmembrane protein | 27 | 237 | 135 | 141 | 132 | 64 | 2.36603E-25 |
| 1358 | - | 1442553 | 1441873 | - | *-* | *PA14_16830* | hypothetical protein | 36 | 139 | 88 | 68 | 90 | 38 | 1.87587E-05 |
| 1359 | - | 1443088 | 1442690 | - | *-* | *PA14_16840* | lipoprotein | 54 | 80 | 39 | 38 | 63 | 46 | 1 |
| 1360 | - | 1445735 | 1443231 | - | *plsB* | *PA14_16860* | glycerol-3-phosphate acyltransferase | 40 | 43 | 41 | 38 | 41 | 33 | 1 |
| 1361 | - | 1446019 | 1446942 | + | *-* | *PA14_16870* | ABC transporter ATP-binding protein | 30 | 30 | 30 | 32 | 33 | 27 | 1 |
| 1362 | - | 1446939 | 1447673 | + | *-* | *PA14_16880* | ABC transporter permease | 24 | 26 | 22 | 25 | 29 | 19 | 1 |
| 1363 | - | 1447684 | 1449531 | + | *-* | *PA14_16890* | auxiliary component of ABC transporter | 38 | 43 | 33 | 34 | 39 | 29 | 1 |
| 1364 | - | 1449535 | 1450515 | + | *-* | *PA14_16910* | hypothetical protein | 106 | 110 | 116 | 111 | 110 | 93 | 1 |
| 1365 | - | 1450944 | 1450522 | - | *-* | *PA14_16920* | hypothetical protein | 150 | 147 | 185 | 150 | 141 | 148 | 1 |
| 1366 | - | 1452146 | 1450941 | - | *-* | *PA14_16930* | pyridoxal-phosphate dependent protein | 87 | 78 | 105 | 87 | 87 | 89 | 1 |
| 1367 | - | 1453275 | 1452241 | - | *dapD* | *PA14_16950* | tetrahydrodipicolinate succinylase | 104 | 110 | 91 | 85 | 90 | 80 | 1 |
| 1368 | - | 1453928 | 1453305 | - | *-* | *PA14_16960* | amino acid transporter LysE | 43 | 29 | 43 | 31 | 36 | 33 | 1 |
| 1369 | - | 1454288 | 1453941 | - | *-* | *PA14_16970* | arsenate reductase | 53 | 44 | 56 | 35 | 35 | 38 | 1 |
| 1370 | - | 1454591 | 1454944 | + | *-* | *PA14_16980* | hypothetical protein | 104 | 119 | 131 | 112 | 111 | 98 | 1 |
| 1371 | - | 1455252 | 1454959 | - | *-* | *PA14_16990* | hypothetical protein | 792 | 879 | 846 | 1050 | 1104 | 869 | 1 |
| 1372 | - | 1455933 | 1455574 | - | *-* | *PA14_17000* | hypothetical protein | 7 | 7 | 10 | 11 | 5 | 6 | 1 |
| 1373 | - | 1456171 | 1457916 | + | *-* | *PA14_17010* | Na(+)/H(+) exchanger protein | 78 | 54 | 63 | 52 | 50 | 53 | 1 |
| 1374 | - | 1459175 | 1457967 | - | *-* | *PA14_17030* | succinyldiaminopimelate transaminase | 86 | 72 | 72 | 75 | 63 | 69 | 1 |
| 1375 | - | 1461895 | 1459193 | - | *glnD* | *PA14_17040* | PII uridylyl-transferase | 59 | 58 | 64 | 65 | 57 | 62 | 1 |
| 1376 | - | 1462847 | 1462062 | - | *map* | *PA14_17050* | methionine aminopeptidase | 172 | 142 | 195 | 142 | 131 | 142 | 1 |
| 1377 | - | 1463112 | 1463852 | + | *rpsB* | *PA14_17060* | 30S ribosomal protein S2 | 278 | 331 | 278 | 189 | 259 | 153 | 1 |
| 1378 | - | 1463983 | 1464852 | + | *tsf* | *PA14_17070* | elongation factor Ts | 145 | 241 | 149 | 94 | 143 | 73 | 1 |
| 1379 | - | 1465051 | 1465788 | + | *pyrH* | *PA14_17080* | uridylate kinase | 256 | 281 | 277 | 266 | 267 | 175 | 1 |
| 1380 | - | 1465791 | 1466348 | + | *frr* | *PA14_17100* | ribosome recycling factor | 279 | 400 | 295 | 276 | 285 | 215 | 1 |
| 1381 | - | 1466364 | 1467119 | + | *uppS* | *PA14_17110* | UDP pyrophosphate synthetase | 155 | 178 | 170 | 136 | 155 | 110 | 1 |
| 1382 | - | 1467113 | 1467928 | + | *cdsA* | *PA14_17120* | phosphatidate cytidylyltransferase | 150 | 151 | 176 | 146 | 157 | 106 | 1 |
| 1383 | - | 1467925 | 1469115 | + | *dxr* | *PA14_17130* | 1-deoxy-D-xylulose 5-phosphate reductoisomerase | 201 | 204 | 207 | 187 | 190 | 146 | 1 |
| 1384 | - | 1469141 | 1470493 | + | *-* | *PA14_17140* | membrane-associated zinc metalloprotease | 181 | 150 | 198 | 177 | 178 | 146 | 1 |
| 1385 | - | 1470564 | 1472948 | + | *-* | *PA14_17150* | outer membrane antigen | 158 | 170 | 158 | 186 | 170 | 161 | 1 |
| 1386 | - | 1472999 | 1473505 | + | *-* | *PA14_17170* | hypothetical protein | 548 | 582 | 556 | 524 | 493 | 481 | 1 |
| 1387 | - | 1473505 | 1474566 | + | *lpxD* | *PA14_17180* | UDP-3-O-[3-hydroxymyristoyl] glucosamine N-acyltransferase | 292 | 279 | 264 | 231 | 264 | 234 | 1 |
| 1388 | - | 1474612 | 1475052 | + | *fabZ* | *PA14_17190* | (3R)-hydroxymyristoyl-ACP dehydratase | 197 | 154 | 199 | 180 | 174 | 178 | 1 |
| 1389 | - | 1475049 | 1475825 | + | *lpxA* | *PA14_17210* | UDP-N-acetylglucosamine acyltransferase | 250 | 236 | 266 | 225 | 237 | 220 | 1 |
| 1390 | - | 1475829 | 1476965 | + | *lpxB* | *PA14_17220* | lipid-A-disaccharide synthase | 87 | 85 | 84 | 80 | 94 | 71 | 1 |
| 1391 | - | 1476965 | 1477570 | + | *rnhB* | *PA14_17230* | ribonuclease HII | 125 | 124 | 126 | 112 | 134 | 98 | 1 |
| 1392 | - | 1477787 | 1479202 | + | *-* | *PA14_17250* | amino acid permease | 49 | 61 | 56 | 41 | 55 | 36 | 1 |
| 1393 | - | 1479332 | 1482853 | + | *dnaE* | *PA14_17260* | DNA polymerase III subunit alpha | 121 | 117 | 119 | 118 | 120 | 110 | 1 |
| 1394 | - | 1483003 | 1483953 | + | *accA* | *PA14_17270* | acetyl-CoA carboxylase carboxyltransferase subunit alpha | 125 | 121 | 126 | 96 | 103 | 93 | 1 |
| 1395 | - | 1484023 | 1485351 | + | *-* | *PA14_17280* | hypothetical protein | 80 | 75 | 81 | 73 | 76 | 60 | 1 |
| 1396 | - | 1485522 | 1487150 | + | *pyrG* | *PA14_17290* | CTP synthetase | 220 | 189 | 230 | 216 | 173 | 180 | 1 |
| 1397 | - | 1487153 | 1487998 | + | *kdsA* | *PA14_17310* | 2-dehydro-3-deoxyphosphooctonate aldolase | 266 | 261 | 266 | 267 | 236 | 230 | 1 |
| 1398 | - | 1488044 | 1489333 | + | *eno* | *PA14_17320* | phosphopyruvate hydratase | 349 | 337 | 326 | 297 | 305 | 264 | 1 |
| 1399 | - | 1489398 | 1489682 | + | *-* | *PA14_17330* | hypothetical protein | 188 | 163 | 182 | 154 | 161 | 168 | 1 |
| 1400 | - | 1489702 | 1490406 | + | *ispD* | *PA14_17340* | 2-C-methyl-D-erythritol 4-phosphate cytidylyltransferase | 49 | 31 | 49 | 28 | 35 | 40 | 1 |
| 1401 | - | 1490715 | 1490467 | - | *-* | *PA14_17350* | hypothetical protein | 45 | 52 | 54 | 40 | 43 | 42 | 1 |
| 1402 | - | 1491907 | 1490681 | - | *-* | *PA14_17370* | inner membrane protein | 51 | 38 | 61 | 36 | 32 | 43 | 1 |
| 1403 | - | 1492891 | 1491983 | - | *-* | *PA14_17380* | LysR family transcriptional regulator | 39 | 32 | 39 | 32 | 33 | 44 | 1 |
| 1404 | - | 1493023 | 1494135 | + | *adhC* | *PA14_17400* | alcohol dehydrogenase | 145 | 159 | 162 | 142 | 139 | 138 | 1 |
| 1405 | - | 1494189 | 1495040 | + | *-* | *PA14_17410* | esterase | 89 | 95 | 84 | 83 | 74 | 83 | 1 |
| 1406 | - | 1495111 | 1495584 | + | *ispF* | *PA14_17420* | 2-C-methyl-D-erythritol 2,4-cyclodiphosphate synthase | 113 | 113 | 127 | 121 | 109 | 100 | 1 |
| 1407 | - | 1495581 | 1496648 | + | *truD* | *PA14_17440* | tRNA pseudouridine synthase D | 83 | 81 | 87 | 84 | 81 | 72 | 1 |
| 1408 | - | 1496636 | 1497385 | + | *surE* | *PA14_17450* | stationary phase survival protein SurE | 106 | 101 | 103 | 109 | 112 | 97 | 1 |
| 1409 | - | 1497418 | 1498053 | + | *pcm* | *PA14_17460* | protein-L-isoaspartate O-methyltransferase | 249 | 256 | 255 | 298 | 296 | 233 | 1 |
| 1410 | - | 1498099 | 1498992 | + | *-* | *PA14_17470* | hypothetical protein | 1134 | 1168 | 1154 | 1376 | 1060 | 1279 | 1 |
| 1411 | - | 1499097 | 1500101 | + | *rpoS* | *PA14_17480* | RNA polymerase sigma factor RpoS | 641 | 827 | 492 | 742 | 707 | 818 | 1 |
| 1412 | - | 1500850 | 1500527 | - | *fdxA* | *PA14_17490* | ferredoxin I | 61 | 76 | 73 | 48 | 71 | 58 | 1 |
| 1413 | - | 1503484 | 1500917 | - | *mutS* | *PA14_17500* | DNA mismatch repair protein MutS | 62 | 60 | 69 | 54 | 54 | 49 | 1 |
| 1414 | - | 1504617 | 1503610 | - | *-* | *PA14_17510* | hypothetical protein | 31 | 24 | 34 | 32 | 26 | 23 | 1 |
| 1415 | - | 1504765 | 1505271 | + | *-* | *PA14_17520* | hypothetical protein | 138 | 143 | 147 | 142 | 123 | 119 | 1 |
| 1416 | - | 1505405 | 1506445 | + | *recA* | *PA14_17530* | recombinase A | 219 | 256 | 220 | 211 | 253 | 229 | 1 |
| 1417 | - | 1506451 | 1506912 | + | *recX* | *PA14_17540* | recombination regulator RecX | 96 | 109 | 91 | 90 | 89 | 92 | 1 |
| 1418 | - | 1508004 | 1506934 | - | *-* | *PA14_17550* | hypothetical protein | 184 | 192 | 185 | 149 | 156 | 184 | 1 |
| 1419 | - | 1509490 | 1508087 | - | *-* | *PA14_17570* | hypothetical protein | 230 | 193 | 235 | 126 | 138 | 193 | 1 |
| 1420 | - | 1509670 | 1512075 | + | *-* | *PA14_17580* | hypothetical protein | 212 | 197 | 256 | 141 | 167 | 164 | 1 |
| 1421 | - | 1512420 | 1512199 | - | *-* | *PA14_17590* | hypothetical protein | 99 | 99 | 131 | 95 | 89 | 90 | 1 |
| 1422 | - | 1512849 | 1512439 | - | *-* | *PA14_17600* | hypothetical protein | 99 | 96 | 117 | 85 | 93 | 82 | 1 |
| 1423 | - | 1514089 | 1513025 | - | *potD* | *PA14_17610* | polyamine ABC transporter | 7 | 7 | 8 | 8 | 7 | 6 | 1 |
| 1424 | - | 1514948 | 1514178 | - | *potC* | *PA14_17620* | polyamine transport protein PotC | 7 | 7 | 9 | 8 | 7 | 8 | 1 |
| 1425 | - | 1515834 | 1514941 | - | *potB* | *PA14_17630* | polyamine transport protein PotB | 5 | 5 | 6 | 5 | 5 | 4 | 1 |
| 1426 | - | 1516930 | 1515839 | - | *potA* | *PA14_17640* | polyamine transport protein PotA | 6 | 4 | 6 | 5 | 4 | 5 | 0.859738761 |
| 1427 | - | 1517189 | 1517905 | + | *-* | *PA14_17650* | hypothetical protein | 29 | 27 | 33 | 28 | 23 | 21 | 1 |
| 1428 | - | 1518835 | 1517909 | - | *-* | *PA14_17660* | hypothetical protein | 24 | 29 | 27 | 23 | 23 | 20 | 1 |

|  | A | B | C | D | E | F | G | H | I | J | K | L | M | N |
| --- | --- | --- | --- | --- | --- | --- | --- | --- | --- | --- | --- | --- | --- | --- |
| 1429 | - | 1518968 | 1519621 | + | *-* | *PA14_17670* | LuxR family transcriptional regulator | 62 | 49 | 55 | 48 | 53 | 48 | 1 |
| 1430 | - | 1519693 | 1520064 | + | *dgkA* | *PA14_17675* | diacylglycerol kinase | 25 | 23 | 20 | 20 | 20 | 18 | 1 |
| 1431 | - | 1521744 | 1520134 | - | *-* | *PA14_17690* | hypothetical protein | 42 | 43 | 36 | 36 | 40 | 36 | 1 |
| 1432 | - | 1521988 | 1522251 | + | *rpmE2* | *PA14_17700* | 50S ribosomal protein L31 | 22 | 38 | 23 | 64 | 51 | 34 | 0.818857487 |
| 1433 | - | 1522251 | 1522403 | + | *rpmJ* | *PA14_17710* | 50S ribosomal protein L36 | 35 | 51 | 28 | 75 | 58 | 42 | 1 |
| 1434 | - | 1523216 | 1522431 | - | *-* | *PA14_17720* | LuxR family transcriptional regulator | 32 | 30 | 30 | 37 | 30 | 25 | 1 |
| 1435 | - | 1523344 | 1524159 | + | *-* | *PA14_17730* | hypothetical protein | 18 | 16 | 20 | 19 | 20 | 16 | 1 |
| 1436 | - | 1524359 | 1525681 | + | *-* | *PA14_17740* | amino acid ABC transporter permease | 8 | 8 | 11 | 10 | 8 | 9 | 1 |
| 1437 | - | 1525771 | 1526844 | + | *-* | *PA14_17760* | methylated-DNA--protein-cysteine methyltransferase | 15 | 16 | 18 | 20 | 14 | 16 | 1 |
| 1438 | - | 1528250 | 1526841 | - | *-* | *PA14_17780* | major facilitator transporter | 11 | 9 | 11 | 11 | 11 | 9 | 1 |
| 1439 | - | 1529356 | 1528469 | - | *-* | *PA14_17790* | LysR family transcriptional regulator | 25 | 22 | 24 | 24 | 18 | 24 | 1 |
| 1440 | - | 1529470 | 1531197 | + | *-* | *PA14_17810* | acyl-CoA dehydrogenase | 10 | 8 | 10 | 10 | 7 | 8 | 1 |
| 1441 | - | 1531236 | 1532423 | + | *-* | *PA14_17820* | hypothetical protein | 5 | 3 | 5 | 5 | 3 | 4 | 0.568108676 |
| 1442 | - | 1532483 | 1533280 | + | *-* | *PA14_17850* | enoyl-CoA hydratase | 2 | 1 | 2 | 1 | 1 | 1 | 1 |
| 1443 | - | 1533277 | 1534809 | + | *-* | *PA14_17860* | 3-hydroxyacyl-CoA dehydrogenase | 10 | 10 | 8 | 10 | 8 | 8 | 1 |
| 1444 | - | 1534832 | 1536037 | + | *-* | *PA14_17880* | acetyl-CoA acetyltransferase | 6 | 6 | 7 | 6 | 5 | 4 | 1 |
| 1445 | - | 1536090 | 1537340 | + | *-* | *PA14_17890* | porin | 10 | 11 | 9 | 10 | 12 | 11 | 1 |
| 1446 | - | 1538266 | 1537346 | - | *metR* | *PA14_17900* | transcriptional regulator MetR | 89 | 61 | 81 | 59 | 93 | 113 | 1 |
| 1447 | - | 1538565 | 1539551 | + | *-* | *PA14_17910* | alpha/beta hydrolase | 9 | 10 | 10 | 10 | 7 | 9 | 1 |
| 1448 | - | 1539897 | 1539568 | - | *glpM* | *PA14_17920* | membrane protein GlpM | 15 | 16 | 11 | 9 | 9 | 12 | 1 |
| 1449 | - | 1541568 | 1540030 | - | *glpD* | *PA14_17930* | glycerol-3-phosphate dehydrogenase | 19 | 41 | 38 | 84 | 196 | 183 | 0.275790074 |
| 1450 | - | 1542602 | 1541847 | - | *glpR* | *PA14_17940* | glycerol-3-phosphate regulon repressor | 32 | 31 | 30 | 33 | 43 | 39 | 1 |
| 1451 | - | 1544325 | 1542808 | - | *glpK* | *PA14_17960* | glycerol kinase | 35 | 65 | 48 | 72 | 154 | 109 | 0.654227233 |
| 1452 | - | 1545204 | 1544365 | - | *glpF* | *PA14_17980* | glycerol uptake facilitator protein | 56 | 111 | 99 | 151 | 252 | 184 | 0.447575716 |
| 1453 | - | 1545469 | 1545939 | + | *-* | *PA14_17990* | hypothetical protein | 22 | 19 | 20 | 21 | 20 | 22 | 1 |
| 1454 | - | 1546003 | 1547487 | + | *-* | *PA14_18010* | glycerol kinase | 23 | 27 | 21 | 27 | 34 | 23 | 1 |
| 1455 | - | 1547683 | 1548468 | + | *-* | *PA14_18020* | PhzF family phenazine biosynthesis protein | 89 | 90 | 69 | 81 | 100 | 109 | 1 |
| 1456 | - | 1549283 | 1549459 | + | *-* | *PA14_18040* | hypothetical protein | 180 | 183 | 177 | 196 | 148 | 170 | 1 |
| 1457 | - | 1549993 | 1549571 | - | *-* | *PA14_18050* | hypothetical protein | 177 | 159 | 203 | 198 | 177 | 181 | 1 |
| 1458 | - | 1550622 | 1550092 | - | *-* | *PA14_18060* | hypothetical protein | 36 | 48 | 40 | 29 | 22 | 11 | 1 |
| 1459 | - | 1550805 | 1551002 | + | *-* | *PA14_18070* | periplasmic metal-binding protein | 158 | 312 | 151 | 257 | 222 | 167 | 0.410206905 |
| 1460 | - | 1551619 | 1550981 | - | *-* | *PA14_18080* | TetR family transcriptional regulator | 76 | 111 | 80 | 101 | 91 | 75 | 1 |
| 1461 | - | 1551985 | 1553163 | + | *-* | *PA14_18090* | major facilitator subfamily transporter protein | 68 | 73 | 116 | 48 | 47 | 38 | 1 |
| 1462 | - | 1553347 | 1553171 | - | *-* | *PA14_18100* | hypothetical protein | 93 | 113 | 149 | 72 | 59 | 58 | 1 |
| 1463 | - | 1554397 | 1553474 | - | *mmsR* | *PA14_18110* | transcriptional regulator MmsR | 22 | 18 | 20 | 21 | 17 | 17 | 1 |
| 1464 | - | 1554529 | 1556022 | + | *mmsA* | *PA14_18120* | methylmalonate-semialdehyde dehydrogenase | 160 | 254 | 258 | 506 | 249 | 413 | 1 |
| 1465 | - | 1556038 | 1556934 | + | *mmsB* | *PA14_18140* | 3-hydroxyisobutyrate dehydrogenase | 78 | 113 | 107 | 243 | 116 | 195 | 1 |
| 1466 | - | 1557036 | 1558922 | + | *-* | *PA14_18150* | acetyl-coa synthetase | 41 | 54 | 43 | 91 | 50 | 76 | 1 |
| 1467 | - | 1559993 | 1558980 | - | *-* | *PA14_18160* | oxidoreductase | 32 | 52 | 28 | 31 | 42 | 35 | 0.885197681 |
| 1468 | - | 1560308 | 1560015 | - | *-* | *PA14_18180* | hypothetical protein | 30 | 46 | 31 | 19 | 48 | 44 | 1 |
| 1469 | - | 1560404 | 1561324 | + | *-* | *PA14_18200* | LysR family transcriptional regulator | 25 | 24 | 21 | 18 | 21 | 19 | 1 |
| 1470 | - | 1562289 | 1561612 | - | *-* | *PA14_18210* | TatD family deoxyribonuclease | 33 | 34 | 34 | 31 | 32 | 28 | 1 |
| 1471 | - | 1563391 | 1562402 | - | *fruR* | *PA14_18230* | DNA-binding transcriptional regulator FruR | 39 | 36 | 40 | 32 | 39 | 31 | 1 |
| 1472 | - | 1563719 | 1566589 | + | *-* | *PA14_18250* | phosphotransferase system enzyme I | 16 | 52 | 42 | 23 | 50 | 32 | 0.007467257 |
| 1473 | - | 1566589 | 1567533 | + | *fruK* | *PA14_18260* | 1-phosphofructokinase | 20 | 66 | 49 | 26 | 63 | 44 | 0.001044072 |
| 1474 | - | 1567535 | 1569292 | + | *fruA* | *PA14_18275* | phosphotransferase system, fructose-specific IIBC component | 25 | 68 | 59 | 30 | 67 | 48 | 0.044138282 |
| 1475 | - | 1570839 | 1569445 | - | *-* | *PA14_18300* | nucleotide sugar dehydrogenase | 24 | 47 | 41 | 47 | 47 | 54 | 0.41668339 |
| 1476 | - | 1571249 | 1570836 | - | *-* | *PA14_18310* | hypothetical protein | 12 | 24 | 23 | 34 | 31 | 32 | 0.366758507 |
| 1477 | - | 1571593 | 1571246 | - | *-* | *PA14_18320* | hypothetical protein | 16 | 20 | 20 | 24 | 23 | 26 | 1 |
| 1478 | - | 1573239 | 1571590 | - | *arnT* | *PA14_18330* | 4-amino-4-deoxy-L-arabinose transferase | 8 | 11 | 12 | 16 | 14 | 16 | 1 |
| 1479 | - | 1574123 | 1573236 | - | *-* | *PA14_18340* | hypothetical protein | 12 | 21 | 19 | 21 | 20 | 21 | 1 |
| 1480 | - | 1576108 | 1574120 | - | *-* | *PA14_18350* | bifunctional UDP-glucuronic acid decarboxylase/UDP-4-amino-4-deoxy-L-arabinose formyltransferase | 20 | 31 | 32 | 34 | 32 | 40 | 1 |
| 1481 | - | 1577124 | 1576105 | - | *-* | *PA14_18360* | glycosyl transferase family protein | 14 | 22 | 23 | 24 | 22 | 26 | 1 |
| 1482 | - | 1578269 | 1577121 | - | *-* | *PA14_18370* | UDP-4-amino-4-deoxy-L-arabinose--oxoglutarate aminotransferase | 10 | 12 | 14 | 13 | 13 | 16 | 1 |
| 1483 | - | 1580102 | 1578657 | - | *algA* | *PA14_18380* | mannose-1-phosphate guanylyltransferase | 11 | 11 | 11 | 11 | 12 | 12 | 1 |
| 1484 | - | 1580949 | 1580299 | - | *algF* | *PA14_18410* | alginate o-acetyltransferase AlgF | 7 | 8 | 7 | 6 | 5 | 6 | 1 |
| 1485 | - | 1582197 | 1581022 | - | *algJ* | *PA14_18430* | alginate o-acetyltransferase AlgJ | 3 | 4 | 5 | 3 | 4 | 3 | 1 |
| 1486 | - | 1583774 | 1582212 | - | *algI* | *PA14_18450* | alginate o-acetyltransferase AlgI | 8 | 7 | 8 | 8 | 6 | 7 | 1 |
| 1487 | - | 1585119 | 1584016 | - | *algL* | *PA14_18470* | poly(beta-D-mannuronate) lyase | 5 | 5 | 5 | 5 | 6 | 6 | 1 |
| 1488 | - | 1586547 | 1585123 | - | *algX* | *PA14_18480* | alginate biosynthesis protein AlgX | 4 | 4 | 4 | 4 | 3 | 4 | 1 |
| 1489 | - | 1588191 | 1586560 | - | *algG* | *PA14_18500* | alginate-c5-mannuronan-epimerase AlgG | 68 | 67 | 67 | 64 | 58 | 61 | 1 |
| 1490 | - | 1589684 | 1588212 | - | *algE* | *PA14_18510* | alginate production outer membrane protein AlgE | 7 | 7 | 8 | 7 | 6 | 5 | 1 |
| 1491 | - | 1591108 | 1589681 | - | *algK* | *PA14_18520* | alginate biosynthetic protein AlgK | 8 | 8 | 10 | 9 | 7 | 6 | 1 |
| 1492 | - | 1592291 | 1591122 | - | *alg44* | *PA14_18550* | alginate biosynthesis protein Alg44 | 6 | 6 | 6 | 6 | 6 | 5 | 1 |
| 1493 | - | 1593867 | 1592374 | - | *alg8* | *PA14_18565* | alginate biosynthesis protein Alg8 | 17 | 14 | 17 | 16 | 15 | 17 | 1 |
| 1494 | - | 1595308 | 1593998 | - | *algD* | *PA14_18580* | GDP-mannose 6-dehydrogenase AlgD | 8 | 8 | 9 | 8 | 7 | 6 | 1 |
| 1495 | - | 1596990 | 1596211 | - | *-* | *PA14_18590* | hypothetical protein | 34 | 16 | 14 | 10 | 14 | 18 | 0.210107695 |
| 1496 | - | 1598179 | 1597097 | - | *-* | *PA14_18600* | ABC transporter ATP-binding protein | 53 | 47 | 49 | 36 | 49 | 44 | 1 |
| 1497 | - | 1599101 | 1598184 | - | *argF* | *PA14_18610* | ornithine carbamoyltransferase | 32 | 27 | 35 | 25 | 29 | 31 | 1 |
| 1498 | - | 1599380 | 1599847 | + | *-* | *PA14_18620* | hypothetical protein | 164 | 151 | 158 | 183 | 132 | 140 | 1 |
| 1499 | - | 1603228 | 1600241 | - | *-* | *PA14_18630* | serine protease | 48 | 70 | 55 | 64 | 62 | 64 | 1 |
| 1500 | - | 1603527 | 1605635 | + | *-* | *PA14_18640* | oxidoreductase | 14 | 14 | 13 | 16 | 15 | 16 | 1 |
| 1501 | - | 1605748 | 1606074 | + | *-* | *PA14_18650* | hypothetical protein | 198 | 283 | 130 | 156 | 291 | 263 | 1 |
| 1502 | - | 1606157 | 1607308 | + | *-* | *PA14_18660* | hypothetical protein | 46 | 36 | 41 | 38 | 46 | 47 | 1 |
| 1503 | - | 1607852 | 1607376 | - | *bfrB* | *PA14_18670* | bacterioferritin | 723 | 395 | 988 | 356 | 162 | 538 | 1 |
| 1504 | - | 1608283 | 1608062 | - | *-* | *PA14_18680* | hypothetical protein | 56 | 136 | 42 | 132 | 86 | 60 | 0.070160504 |
| 1505 | - | 1608543 | 1609145 | + | *-* | *PA14_18690* | peroxidase | 891 | 1221 | 725 | 549 | 837 | 607 | 1 |
| 1506 | - | 1609998 | 1609324 | - | *rnt* | *PA14_18700* | ribonuclease T | 92 | 139 | 111 | 125 | 134 | 101 | 1 |
| 1507 | - | 1611041 | 1609995 | - | *pyrC* | *PA14_18710* | dihydroorotase | 61 | 66 | 71 | 51 | 54 | 43 | 1 |
| 1508 | - | 1611174 | 1612139 | + | *-* | *PA14_18720* | OmpA family membrane protein | 96 | 87 | 114 | 84 | 103 | 101 | 1 |
| 1509 | - | 1612257 | 1613474 | + | *argG* | *PA14_18740* | argininosuccinate synthase | 83 | 70 | 86 | 61 | 65 | 59 | 1 |
| 1510 | - | 1613952 | 1613566 | - | *gloA1* | *PA14_18750* | lactoylglutathione lyase | 70 | 97 | 78 | 47 | 58 | 56 | 1 |
| 1511 | - | 1614329 | 1615486 | + | *-* | *PA14_18760* | RND efflux membrane fusion protein | 6 | 5 | 5 | 5 | 5 | 4 | 1 |
| 1512 | - | 1615483 | 1618644 | + | *-* | *PA14_18780* | RND efflux transporter | 8 | 8 | 6 | 7 | 7 | 6 | 1 |

|  | A | B | C | D | E | F | G | H | I | J | K | L | M | N |
| --- | --- | --- | --- | --- | --- | --- | --- | --- | --- | --- | --- | --- | --- | --- |
| 1513 | - | 1618641 | 1620116 | + | *-* | *PA14_18790* | outer membrane efflux protein | 5 | 5 | 4 | 5 | 5 | 5 | 1 |
| 1514 | 1620628 | 1620687 | 1620881 | + | *-* | *PA14_18800* | hypothetical protein | 35 | 8 | 9 | 32 | 27 | 57 | 3.95499E-10 |
| 1515 | 1621028 | 1621282 | 1622319 | + | *-* | *PA14_18810* | hypothetical protein | 4 | 7 | 3 | 3 | 6 | 5 | 0.630688527 |
| 1516 | 1621028 | 1622352 | 1623338 | + | *-* | *PA14_18820* | hypothetical protein | 3 | 6 | 2 | 5 | 5 | 4 | 1 |
| 1517 | 1621028 | 1623369 | 1624802 | + | *-* | *PA14_18830* | adenylosuccinate lyase | 11 | 11 | 15 | 15 | 10 | 13 | 1 |
| 1518 | 1621028 | 1624802 | 1626253 | + | *-* | *PA14_18850* | adenylosuccinate lyase | 7 | 7 | 6 | 9 | 5 | 6 | 1 |
| 1519 | 1621028 | 1626303 | 1627376 | + | *-* | *PA14_18860* | hypothetical protein | 28 | 33 | 27 | 35 | 27 | 28 | 1 |
| 1520 | - | 1627755 | 1627576 | - | *-* | *PA14_18870* | hypothetical protein | 80 | 132 | 70 | 67 | 43 | 53 | 0.903714641 |
| 1521 | - | 1628502 | 1627864 | - | *nth* | *PA14_18880* | endonuclease III | 35 | 49 | 43 | 36 | 43 | 33 | 1 |
| 1522 | - | 1629215 | 1628499 | - | *-* | *PA14_18890* | electron transport complex protein RsxE | 42 | 39 | 44 | 45 | 45 | 38 | 1 |
| 1523 | - | 1629852 | 1629208 | - | *-* | *PA14_18900* | hypothetical protein | 44 | 43 | 52 | 56 | 55 | 47 | 1 |
| 1524 | - | 1630886 | 1629852 | - | *-* | *PA14_18910* | hypothetical protein | 31 | 36 | 33 | 40 | 38 | 34 | 1 |
| 1525 | - | 1633213 | 1630889 | - | *-* | *PA14_18920* | electron transport complex protein RnfC | 42 | 47 | 46 | 47 | 45 | 37 | 1 |
| 1526 | - | 1633776 | 1633210 | - | *-* | *PA14_18930* | electron transport complex protein RnfB | 44 | 38 | 49 | 50 | 40 | 35 | 1 |
| 1527 | - | 1634357 | 1633773 | - | *-* | *PA14_18950* | Na(+)-translocating NADH-quinone reductase subunit E | 27 | 22 | 28 | 24 | 20 | 20 | 1 |
| 1528 | - | 1635233 | 1634535 | - | *-* | *PA14_18960* | hypothetical protein | 44 | 42 | 53 | 64 | 53 | 50 | 1 |
| 1529 | - | 1638973 | 1635662 | - | *pldA* | *PA14_18970* | phospholipase D | 34 | 28 | 24 | 28 | 32 | 27 | 1 |
| 1530 | - | 1641403 | 1638977 | - | *-* | *PA14_18985* | hypothetical protein | 20 | 19 | 15 | 18 | 22 | 15 | 1 |
| 1531 | - | 1642135 | 1641698 | - | *-* | *PA14_19010* | hypothetical protein | 26 | 22 | 20 | 18 | 17 | 16 | 1 |
| 1532 | - | 1643358 | 1642132 | - | *-* | *PA14_19020* | hypothetical protein | 31 | 27 | 23 | 24 | 29 | 23 | 1 |
| 1533 | - | 1644245 | 1643445 | - | *-* | *PA14_19030* | hypothetical protein | 66 | 62 | 65 | 51 | 56 | 40 | 1 |
| 1534 | - | 1646315 | 1644279 | - | *metG* | *PA14_19050* | methionyl-tRNA synthetase | 105 | 115 | 108 | 94 | 103 | 90 | 1 |
| 1535 | - | 1646446 | 1647540 | + | *-* | *PA14_19065* | hypothetical protein | 64 | 66 | 59 | 49 | 66 | 50 | 1 |
| 1536 | - | 1647642 | 1648208 | + | *dcd* | *PA14_19090* | deoxycytidine triphosphate deaminase | 58 | 46 | 49 | 46 | 43 | 42 | 1 |
| 1537 | 1648391 | 1648632 | 1649519 | + | *rhlA* | *PA14_19100* | rhamnosyltransferase chain A | 452 | 9 | 87 | 436 | 240 | 550 | 0 |
| 1538 | 1648391 | 1649585 | 1650865 | + | *rhlB* | *PA14_19110* | rhamnosyltransferase chain B | 217 | 18 | 37 | 255 | 124 | 301 | 3.12603E-64 |
| 1539 | - | 1650990 | 1651715 | + | *rhlR* | *PA14_19120* | transcriptional regulator RhlR | 707 | 1 | 615 | 994 | 731 | 898 | 0 |
| 1540 | 1651804 | 1651896 | 1652501 | + | *rhlI* | *PA14_19130* | autoinducer synthesis protein RhlI | 210 | 142 | 5 | 206 | 191 | 193 | 1 |
| 1541 | - | 1652656 | 1653462 | + | *pheC* | *PA14_19140* | cyclohexadienyl dehydratase | 45 | 39 | 141 | 48 | 40 | 42 | 1 |
| 1542 | - | 1653556 | 1654416 | + | *-* | *PA14_19150* | hypothetical protein | 10 | 12 | 25 | 11 | 13 | 10 | 1 |
| 1543 | - | 1654413 | 1655312 | + | *-* | *PA14_19160* | hypothetical protein | 18 | 26 | 30 | 23 | 22 | 17 | 1 |
| 1544 | - | 1655929 | 1655333 | - | *-* | *PA14_19170* | hypothetical protein | 47 | 61 | 68 | 49 | 43 | 42 | 1 |
| 1545 | - | 1656387 | 1658081 | + | *-* | *PA14_19190* | malate dehydrogenase | 93 | 137 | 93 | 85 | 111 | 71 | 1 |
| 1546 | - | 1658661 | 1658203 | - | *-* | *PA14_19205* | hypothetical protein | 47 | 47 | 47 | 42 | 44 | 35 | 1 |
| 1547 | - | 1659200 | 1658658 | - | *-* | *PA14_19210* | hypothetical protein | 34 | 39 | 32 | 27 | 36 | 24 | 1 |
| 1548 | - | 1660546 | 1659218 | - | *-* | *PA14_19230* | hypothetical protein | 26 | 32 | 26 | 22 | 29 | 20 | 1 |
| 1549 | - | 1662081 | 1660714 | - | *-* | *PA14_19270* | MFS transporter | 6 | 7 | 6 | 7 | 6 | 7 | 1 |
| 1550 | - | 1662267 | 1663607 | + | *-* | *PA14_19290* | ATP-dependent RNA helicase | 57 | 54 | 65 | 59 | 56 | 56 | 1 |
| 1551 | - | 1665444 | 1663729 | - | *-* | *PA14_19310* | hypothetical protein | 258 | 225 | 305 | 204 | 214 | 274 | 1 |
| 1552 | - | 1665661 | 1666986 | + | *-* | *PA14_19320* | hypothetical protein | 40 | 36 | 37 | 41 | 33 | 38 | 1 |
| 1553 | - | 1667345 | 1667115 | - | *-* | *PA14_19330* | hypothetical protein | 30 | 32 | 32 | 33 | 28 | 32 | 1 |
| 1554 | - | 1670152 | 1667396 | - | *-* | *PA14_19340* | sensor/response regulator hybrid | 22 | 21 | 25 | 28 | 21 | 29 | 1 |
| 1555 | - | 1671485 | 1670289 | - | *-* | *PA14_19350* | hypothetical protein | 67 | 100 | 91 | 114 | 125 | 130 | 1 |
| 1556 | - | 1673239 | 1671482 | - | *-* | *PA14_19360* | GNAT family acetyltransferase | 101 | 140 | 133 | 166 | 180 | 178 | 1 |
| 1557 | - | 1675049 | 1673280 | - | *-* | *PA14_19370* | asparagine synthetase | 262 | 240 | 278 | 202 | 263 | 278 | 1 |
| 1558 | - | 1675192 | 1675665 | + | *-* | *PA14_19380* | transcriptional regulator | 282 | 212 | 270 | 146 | 181 | 304 | 1 |
| 1559 | - | 1676673 | 1675921 | - | *-* | *PA14_19390* | hypothetical protein | 100 | 87 | 117 | 69 | 67 | 72 | 1 |
| 1560 | - | 1678738 | 1676774 | - | *mnmC* | *PA14_19400* | 5-methylaminomethyl-2-thiouridine methyltransferase | 38 | 32 | 32 | 34 | 29 | 25 | 1 |
| 1561 | - | 1678882 | 1680372 | + | *-* | *PA14_19410* | hypothetical protein | 100 | 101 | 97 | 93 | 96 | 98 | 1 |
| 1562 | - | 1680525 | 1681709 | + | *-* | *PA14_19430* | acyl-CoA thiolase | 26 | 24 | 21 | 20 | 29 | 27 | 1 |
| 1563 | - | 1681838 | 1682503 | + | *-* | *PA14_19450* | hypothetical protein | 19 | 21 | 18 | 14 | 18 | 13 | 1 |
| 1564 | - | 1684298 | 1682727 | - | *mqoA* | *PA14_19470* | malate:quinone oxidoreductase | 17 | 13 | 16 | 14 | 16 | 14 | 1 |
| 1565 | - | 1684710 | 1684501 | - | *-* | *PA14_19480* | hypothetical protein | 40 | 36 | 46 | 60 | 37 | 58 | 1 |
| 1566 | - | 1684916 | 1685554 | + | *-* | *PA14_19490* | antioxidant protein | 14 | 62 | 21 | 17 | 31 | 11 | 3.07409E-09 |
| 1567 | - | 1685805 | 1686809 | + | *-* | *PA14_19500* | hypothetical protein | 1 | 2 | 1 | 1 | 1 | 1 | 1 |
| 1568 | - | 1686813 | 1687637 | + | *-* | *PA14_19510* | ABC transporter permease | 3 | 5 | 3 | 4 | 3 | 3 | 1 |
| 1569 | - | 1687634 | 1688383 | + | *-* | *PA14_19520* | ABC transporter ATP-binding protein | 2 | 3 | 3 | 2 | 1 | 4 | 1 |
| 1570 | - | 1688537 | 1689130 | + | *-* | *PA14_19530* | NAD(P)H-dependent FMN reductase | 6 | 40 | 12 | 11 | 20 | 9 | 2.45612E-25 |
| 1571 | - | 1689249 | 1690220 | + | *-* | *PA14_19540* | hypothetical protein | 9 | 15 | 9 | 9 | 9 | 7 | 0.940015681 |
| 1572 | - | 1690298 | 1691446 | + | *ssuD* | *PA14_19560* | alkanesulfonate monooxygenase | 2 | 3 | 1 | 2 | 2 | 1 | 1 |
| 1573 | - | 1691467 | 1692255 | + | *-* | *PA14_19570* | ABC transporter permease | 3 | 3 | 2 | 2 | 2 | 1 | 1 |
| 1574 | - | 1692252 | 1693076 | + | *ssuB* | *PA14_19580* | aliphatic sulfonates transport ATP-binding subunit | 7 | 8 | 7 | 6 | 6 | 5 | 1 |
| 1575 | - | 1693117 | 1693332 | + | *-* | *PA14_19590* | molybdopterin-binding protein | 14 | 19 | 9 | 11 | 12 | 11 | 1 |
| 1576 | - | 1693850 | 1693512 | - | *-* | *PA14_19600* | hypothetical protein | 60 | 75 | 59 | 50 | 59 | 43 | 1 |
| 1577 | - | 1694213 | 1693902 | - | *-* | *PA14_19610* | hypothetical protein | 52 | 68 | 70 | 48 | 57 | 59 | 1 |
| 1578 | - | 1694713 | 1694342 | - | *folX* | *PA14_19620* | D-erythro-7,8-dihydroneopterin triphosphate 2'-epimerase | 102 | 127 | 81 | 106 | 152 | 137 | 1 |
| 1579 | - | 1695275 | 1694715 | - | *folE1* | *PA14_19630* | GTP cyclohydrolase I | 120 | 116 | 101 | 107 | 132 | 123 | 1 |
| 1580 | - | 1695997 | 1695293 | - | *-* | *PA14_19640* | short chain dehydrogenase | 34 | 35 | 30 | 26 | 43 | 39 | 1 |
| 1581 | - | 1696665 | 1696108 | - | *-* | *PA14_19650* | hypothetical protein | 5 | 5 | 6 | 6 | 5 | 4 | 1 |
| 1582 | - | 1696945 | 1697397 | + | *-* | *PA14_19660* | flavodoxin | 75 | 99 | 106 | 93 | 87 | 72 | 1 |
| 1583 | - | 1698429 | 1697536 | - | *-* | *PA14_19670* | LysR family transcriptional regulator | 23 | 27 | 24 | 22 | 20 | 21 | 1 |
| 1584 | - | 1698525 | 1698914 | + | *-* | *PA14_19680* | hypothetical protein | 27 | 25 | 34 | 38 | 19 | 40 | 1 |
| 1585 | - | 1698901 | 1699587 | + | *-* | *PA14_19690* | hypothetical protein | 33 | 28 | 41 | 44 | 25 | 47 | 1 |
| 1586 | - | 1699716 | 1700495 | + | *-* | *PA14_19700* | aldolase | 43 | 45 | 49 | 43 | 50 | 59 | 1 |
| 1587 | - | 1700492 | 1701388 | + | *-* | *PA14_19710* | alpha/beta hydrolase | 23 | 20 | 19 | 17 | 21 | 26 | 1 |
| 1588 | - | 1701487 | 1701780 | + | *-* | *PA14_19720* | hypothetical protein | 16 | 13 | 21 | 18 | 13 | 20 | 1 |
| 1589 | - | 1701896 | 1702807 | + | *-* | *PA14_19730* | oxidoreductase | 25 | 28 | 31 | 29 | 43 | 38 | 1 |
| 1590 | - | 1702924 | 1703694 | + | *-* | *PA14_19740* | enoyl-CoA hydratase | 135 | 130 | 163 | 176 | 138 | 147 | 1 |
| 1591 | - | 1704058 | 1703714 | - | *-* | *PA14_19750* | hypothetical protein | 58 | 67 | 75 | 91 | 69 | 72 | 1 |
| 1592 | - | 1705499 | 1704093 | - | *-* | *PA14_19770* | hypothetical protein | 16 | 18 | 18 | 20 | 16 | 22 | 1 |
| 1593 | - | 1706333 | 1705590 | - | *-* | *PA14_19800* | AraC family transcriptional regulator | 27 | 24 | 28 | 24 | 23 | 23 | 1 |
| 1594 | - | 1706618 | 1708375 | + | *-* | *PA14_19810* | hypothetical protein | 15 | 14 | 18 | 19 | 14 | 17 | 1 |
| 1595 | - | 1708432 | 1709802 | + | *-* | *PA14_19830* | hypothetical protein | 13 | 13 | 15 | 13 | 10 | 14 | 1 |
| 1596 | - | 1709952 | 1712435 | + | *-* | *PA14_19850* | transcriptional regulator | 9 | 8 | 8 | 9 | 9 | 11 | 1 |

|  | A | B | C | D | E | F | G | H | I | J | K | L | M | N |
| --- | --- | --- | --- | --- | --- | --- | --- | --- | --- | --- | --- | --- | --- | --- |
| 1597 | - | 1713254 | 1712442 | - | *-* | *PA14_19860* | hypothetical protein | 73 | 72 | 78 | 86 | 69 | 64 | 1 |
| 1598 | - | 1713500 | 1714525 | + | *ldh* | *PA14_19870* | leucine dehydrogenase | 278 | 334 | 374 | 314 | 330 | 352 | 1 |
| 1599 | - | 1714667 | 1715764 | + | *-* | *PA14_19900* | pyruvate dehydrogenase E1 component subunit alpha | 136 | 173 | 256 | 220 | 190 | 238 | 1 |
| 1600 | - | 1715757 | 1716758 | + | *-* | *PA14_19910* | pyruvate dehydrogenase E1 component, beta chain | 129 | 164 | 218 | 230 | 202 | 268 | 1 |
| 1601 | - | 1716776 | 1717888 | + | *-* | *PA14_19920* | branched-chain alpha-keto acid dehydrogenase subunit E2 | 56 | 81 | 96 | 106 | 83 | 126 | 1 |
| 1602 | - | 1718347 | 1717889 | - | *-* | *PA14_19930* | hypothetical protein | 23 | 40 | 40 | 53 | 43 | 60 | 0.882653101 |
| 1603 | - | 1718965 | 1718381 | - | *-* | *PA14_19940* | hypothetical protein | 61 | 83 | 90 | 93 | 88 | 92 | 1 |
| 1604 | - | 1719267 | 1719001 | - | *-* | *PA14_19950* | hypothetical protein | 271 | 325 | 326 | 380 | 354 | 338 | 1 |
| 1605 | - | 1719477 | 1719746 | + | *-* | *PA14_19960* | hypothetical protein | 15 | 17 | 12 | 27 | 13 | 14 | 1 |
| 1606 | - | 1719866 | 1720084 | + | *-* | *PA14_19970* | hypothetical protein | 4 | 13 | 3 | 13 | 8 | 2 | 9.20065E-07 |
| 1607 | - | 1720161 | 1720676 | + | *-* | *PA14_19990* | RNA polymerase ECF-subfamily sigma-70 factor | 5 | 9 | 4 | 19 | 10 | 5 | 1 |
| 1608 | - | 1720741 | 1721727 | + | *-* | *PA14_20000* | transmembrane sensor | 12 | 12 | 13 | 21 | 12 | 13 | 1 |
| 1609 | - | 1721937 | 1724612 | + | *hasR* | *PA14_20010* | heme uptake outer membrane receptor HasR | 8 | 9 | 8 | 11 | 8 | 8 | 1 |
| 1610 | - | 1724698 | 1725315 | + | *hasAp* | *PA14_20020* | heme acquisition protein HasAp | 46 | 43 | 53 | 51 | 34 | 39 | 1 |
| 1611 | - | 1725533 | 1727323 | + | *hasD* | *PA14_20030* | transport protein HasD | 3 | 2 | 3 | 2 | 1 | 2 | 1 |
| 1612 | - | 1727320 | 1728651 | + | *hasE* | *PA14_20040* | metalloprotease secretion protein | 3 | 2 | 4 | 2 | 2 | 2 | 0.925024982 |
| 1613 | - | 1728648 | 1730003 | + | *-* | *PA14_20050* | outer membrane protein | 6 | 6 | 7 | 7 | 7 | 7 | 1 |
| 1614 | - | 1730283 | 1729996 | - | *-* | *PA14_20060* | hypothetical protein | 21 | 19 | 26 | 22 | 19 | 18 | 1 |
| 1615 | - | 1730458 | 1730943 | + | *-* | *PA14_20070* | hypothetical protein | 14 | 14 | 13 | 15 | 13 | 12 | 1 |
| 1616 | - | 1731056 | 1732027 | + | *-* | *PA14_20080* | hypothetical protein | 20 | 18 | 19 | 16 | 15 | 13 | 1 |
| 1617 | - | 1732031 | 1733203 | + | *-* | *PA14_20100* | hypothetical protein | 27 | 27 | 25 | 26 | 23 | 23 | 1 |
| 1618 | - | 1733200 | 1734333 | + | *-* | *PA14_20110* | hypothetical protein | 25 | 25 | 23 | 27 | 21 | 22 | 1 |
| 1619 | - | 1734387 | 1734728 | + | *-* | *PA14_20120* | hypothetical protein | 129 | 145 | 125 | 148 | 135 | 136 | 1 |
| 1620 | 1735823 | 1735656 | 1734730 | - | *-* | *PA14_20130* | LysR family transcriptional regulator | 61 | 64 | 63 | 64 | 64 | 60 | 1 |
| 1621 | 1735823 | 1735930 | 1736706 | + | *fpr* | *PA14_20140* | ferredoxin--NADP+ reductase | 215 | 176 | 193 | 176 | 215 | 232 | 1 |
| 1622 | - | 1737325 | 1736789 | - | *nosL* | *PA14_20150* | NosL protein | 26 | 53 | 75 | 80 | 120 | 102 | 0.285762132 |
| 1623 | - | 1738169 | 1737342 | - | *nosY* | *PA14_20170* | NosY protein | 8 | 32 | 38 | 51 | 70 | 61 | 4.22828E-06 |
| 1624 | - | 1739074 | 1738160 | - | *nosF* | *PA14_20180* | NosF protein | 18 | 51 | 62 | 68 | 93 | 84 | 0.010969112 |
| 1625 | - | 1740357 | 1739071 | - | *nosD* | *PA14_20190* | copper ABC transporter periplasmic substrate-binding protein | 36 | 103 | 118 | 122 | 171 | 178 | 0.024246819 |
| 1626 | - | 1742264 | 1740354 | - | *nosZ* | *PA14_20200* | nitrous-oxide reductase | 49 | 214 | 147 | 188 | 254 | 264 | 0.000219048 |
| 1627 | - | 1744455 | 1742308 | - | *nosR* | *PA14_20230* | regulatory protein NosR | 60 | 88 | 63 | 164 | 237 | 190 | 1 |
| 1628 | - | 1745097 | 1744684 | - | *-* | *PA14_20240* | ring-cleaving dioxygenase | 4 | 4 | 4 | 4 | 4 | 3 | 1 |
| 1629 | - | 1745158 | 1745460 | + | *-* | *PA14_20250* | hypothetical protein | 6 | 7 | 9 | 8 | 9 | 7 | 1 |
| 1630 | - | 1746162 | 1745467 | - | *-* | *PA14_20260* | hypothetical protein | 8 | 9 | 9 | 9 | 8 | 9 | 1 |
| 1631 | - | 1746985 | 1746215 | - | *rhlG* | *PA14_20270* | beta-ketoacyl reductase | 6 | 7 | 8 | 8 | 7 | 7 | 1 |
| 1632 | - | 1747448 | 1747083 | - | *-* | *PA14_20280* | hypothetical protein | 15 | 25 | 20 | 28 | 31 | 25 | 0.844537532 |
| 1633 | - | 1747928 | 1747602 | - | *-* | *PA14_20290* | DNA binding-protein | 581 | 839 | 601 | 777 | 982 | 897 | 1 |
| 1634 | - | 1748289 | 1749125 | + | *phnC* | *PA14_20300* | ABC phosphonate transporter ATP-binding protein | 15 | 11 | 14 | 15 | 13 | 13 | 1 |
| 1635 | - | 1749178 | 1750182 | + | *phnD* | *PA14_20320* | phosphonate ABC transporter substrate-binding protein | 4 | 3 | 3 | 4 | 3 | 3 | 1 |
| 1636 | - | 1750250 | 1751044 | + | *phnE* | *PA14_20330* | phosphonate ABC transporter permease | 2 | 3 | 4 | 3 | 2 | 2 | 1 |
| 1637 | - | 1751065 | 1751787 | + | *phnF* | *PA14_20350* | transcriptional regulator | 5 | 6 | 6 | 6 | 6 | 6 | 1 |
| 1638 | - | 1751797 | 1752255 | + | *phnG* | *PA14_20360* | phosphonate metabolism protein PhnG | 42 | 31 | 42 | 37 | 30 | 33 | 1 |
| 1639 | - | 1752255 | 1752884 | + | *phnH* | *PA14_20370* | carbon-phosphorus lyase complex subunit | 28 | 24 | 26 | 22 | 15 | 19 | 1 |
| 1640 | - | 1752884 | 1753984 | + | *phnI* | *PA14_20380* | hypothetical protein | 9 | 9 | 10 | 9 | 9 | 10 | 1 |
| 1641 | - | 1753981 | 1754865 | + | *phnJ* | *PA14_20390* | hypothetical protein | 8 | 8 | 10 | 11 | 8 | 9 | 1 |
| 1642 | - | 1754862 | 1755677 | + | *phnK* | *PA14_20400* | phosphonate C-P lyase system protein PhnK | 8 | 8 | 8 | 9 | 8 | 10 | 1 |
| 1643 | - | 1755728 | 1756444 | + | *phnL* | *PA14_20420* | phosphonate ABC transporter ATPase | 5 | 6 | 7 | 5 | 6 | 6 | 1 |
| 1644 | - | 1756434 | 1757597 | + | *phnM* | *PA14_20430* | phosphonate metabolism protein | 19 | 19 | 22 | 29 | 19 | 23 | 1 |
| 1645 | - | 1757597 | 1758154 | + | *phnN* | *PA14_20440* | phosphonate transport ATP-binding protein | 96 | 92 | 107 | 121 | 102 | 108 | 1 |
| 1646 | - | 1758145 | 1758915 | + | *phnP* | *PA14_20450* | carbon-phosphorus lyase complex accessory protein | 49 | 36 | 47 | 47 | 46 | 48 | 1 |
| 1647 | - | 1759139 | 1758954 | - | *-* | *PA14_20460* | hypothetical protein | 180 | 105 | 140 | 139 | 131 | 120 | 0.87182855 |
| 1648 | - | 1759337 | 1759191 | - | *-* | *PA14_20470* | hypothetical protein | 182 | 114 | 135 | 139 | 124 | 133 | 1 |
| 1649 | - | 1759704 | 1759426 | - | *-* | *PA14_20480* | hypothetical protein | 67 | 51 | 46 | 47 | 47 | 52 | 1 |
| 1650 | - | 1760415 | 1759897 | - | *-* | *PA14_20491* | acetyltransferase | 17 | 15 | 16 | 16 | 14 | 17 | 1 |
| 1651 | - | 1760535 | 1760608 | + | *-* | *PA14_20500* | Arg tRNA | 30 | 30 | 28 | 25 | 22 | 27 | 1 |
| 1652 | - | 1762508 | 1763482 | + | *-* | *PA14_20510* | hypothetical protein | 29 | 38 | 27 | 56 | 45 | 26 | 1 |
| 1653 | - | 1763497 | 1766208 | + | *-* | *PA14_20520* | hypothetical protein | 41 | 46 | 37 | 70 | 56 | 38 | 1 |
| 1654 | - | 1766195 | 1766671 | + | *-* | *PA14_20530* | hypothetical protein | 27 | 40 | 25 | 61 | 55 | 25 | 1 |
| 1655 | - | 1768341 | 1768003 | - | *-* | *PA14_20550* | hypothetical protein | 9 | 5 | 6 | 5 | 4 | 3 | 0.738547973 |
| 1656 | - | 1768608 | 1769648 | + | *amiE* | *PA14_20560* | acylamide amidohydrolase | 78 | 103 | 85 | 86 | 66 | 72 | 1 |
| 1657 | - | 1769732 | 1770847 | + | *-* | *PA14_20570* | chaperone | 35 | 49 | 43 | 47 | 33 | 38 | 1 |
| 1658 | - | 1770873 | 1772030 | + | *amiC* | *PA14_20580* | aliphatic amidase expression-regulating protein | 46 | 51 | 48 | 59 | 41 | 45 | 1 |
| 1659 | - | 1772027 | 1772617 | + | *amiR* | *PA14_20590* | aliphatic amidase regulator | 60 | 71 | 61 | 82 | 62 | 79 | 1 |
| 1660 | - | 1773139 | 1773813 | + | *-* | *PA14_20600* | hypothetical protein | 74 | 32 | 43 | 42 | 30 | 61 | 0.061837823 |
| 1661 | 1774336 | 1774207 | 1773860 | - | *lecB* | *PA14_20610* | fucose-binding lectin PA-IIL | 393 | 69 | 176 | 137 | 96 | 449 | 7.47627E-12 |
| 1662 | 1774336 | 1774674 | 1775732 | + | *-* | *PA14_20620* | HlyD family secretion protein | 17 | 12 | 18 | 16 | 12 | 14 | 1 |
| 1663 | 1774336 | 1775755 | 1776765 | + | *-* | *PA14_20630* | hypothetical protein | 9 | 8 | 8 | 11 | 9 | 10 | 1 |
| 1664 | - | 1777648 | 1776770 | - | *-* | *PA14_20640* | hypothetical protein | 10 | 11 | 8 | 9 | 10 | 7 | 1 |
| 1665 | - | 1779051 | 1777705 | - | *dsdA* | *PA14_20650* | D-serine dehydratase | 33 | 34 | 31 | 34 | 31 | 36 | 1 |
| 1666 | - | 1779377 | 1780618 | + | *-* | *PA14_20670* | glutamine synthetase | 152 | 122 | 108 | 104 | 118 | 126 | 1 |
| 1667 | - | 1780802 | 1782106 | + | *-* | *PA14_20680* | MFS transporter | 44 | 41 | 40 | 47 | 38 | 44 | 1 |
| 1668 | - | 1782148 | 1782759 | + | *-* | *PA14_20690* | hypothetical protein | 160 | 140 | 161 | 160 | 128 | 161 | 1 |
| 1669 | - | 1783554 | 1782763 | - | *-* | *PA14_20700* | glycosyltransferase | 142 | 128 | 155 | 126 | 125 | 140 | 1 |
| 1670 | - | 1784053 | 1783583 | - | *flgN* | *PA14_20720* | hypothetical protein | 294 | 322 | 333 | 230 | 313 | 288 | 1 |
| 1671 | - | 1784431 | 1784108 | - | *flgM* | *PA14_20730* | hypothetical protein | 481 | 605 | 574 | 448 | 567 | 606 | 1 |
| 1672 | - | 1785274 | 1784576 | - | *flgA* | *PA14_20740* | flagellar basal body P-ring biosynthesis protein FlgA | 77 | 61 | 79 | 78 | 62 | 60 | 1 |
| 1673 | - | 1785406 | 1786338 | + | *-* | *PA14_20750* | chemotaxis protein | 963 | 897 | 1018 | 903 | 857 | 965 | 1 |
| 1674 | - | 1786415 | 1787239 | + | *-* | *PA14_20760* | chemotaxis protein methyltransferase | 463 | 460 | 512 | 579 | 546 | 602 | 1 |
| 1675 | - | 1787554 | 1787859 | + | *-* | *PA14_20770* | hypothetical protein | 279 | 381 | 413 | 478 | 440 | 499 | 1 |
| 1676 | - | 1787861 | 1789576 | + | *-* | *PA14_20780* | two-component response regulator | 170 | 208 | 230 | 345 | 236 | 285 | 1 |
| 1677 | - | 1789642 | 1789992 | + | *-* | *PA14_20800* | histidine phosphotransfer domain-containing protein | 106 | 91 | 115 | 142 | 101 | 124 | 1 |
| 1678 | - | 1790076 | 1792214 | + | *recQ* | *PA14_20810* | ATP-dependent DNA helicase RecQ | 94 | 83 | 104 | 91 | 74 | 70 | 1 |
| 1679 | - | 1792303 | 1793472 | + | *-* | *PA14_20820* | two-component response regulator | 63 | 78 | 74 | 88 | 75 | 73 | 1 |
| 1680 | - | 1793623 | 1794639 | + | *-* | *PA14_20840* | hypothetical protein | 70 | 76 | 72 | 84 | 77 | 93 | 1 |

|  | A | B | C | D | E | F | G | H | I | J | K | L | M | N |
| --- | --- | --- | --- | --- | --- | --- | --- | --- | --- | --- | --- | --- | --- | --- |
| 1681 | - | 1794757 | 1795191 | + | *-* | *PA14_20850* | MarR family transcriptional regulator | 83 | 95 | 92 | 82 | 91 | 109 | 1 |
| 1682 | - | 1795468 | 1797513 | + | *-* | *PA14_20860* | Tfp pilus assembly protein FimV | 54 | 48 | 54 | 59 | 47 | 69 | 1 |
| 1683 | - | 1799707 | 1797521 | - | *-* | *PA14_20870* | hypothetical protein | 20 | 23 | 25 | 27 | 23 | 30 | 1 |
| 1684 | - | 1799776 | 1800066 | + | *-* | *PA14_20880* | hypothetical protein | 32 | 16 | 26 | 19 | 27 | 24 | 0.047727227 |
| 1685 | - | 1800156 | 1801154 | + | *rfaD* | *PA14_20890* | ADP-L-glycero-D-manno-heptose-6-epimerase | 336 | 338 | 439 | 109 | 151 | 314 | 1 |
| 1686 | - | 1802331 | 1801165 | - | *-* | *PA14_20900* | MFS transporter | 247 | 58 | 77 | 76 | 147 | 123 | 0.001792166 |
| 1687 | - | 1803175 | 1802423 | - | *-* | *PA14_20920* | hypothetical protein | 208 | 12 | 22 | 64 | 156 | 111 | 3.476E-252 |
| 1688 | 1816906 | 1803532 | 1803293 | - | *-* | *PA14_20940* | acyl carrier protein | 247 | 13 | 35 | 99 | 206 | 156 | 0 |
| 1689 | 1816906 | 1804538 | 1803546 | - | *fabH2* | *PA14_20950* | 3-oxoacyl-ACP synthase | 268 | 11 | 33 | 82 | 187 | 127 | 0 |
| 1690 | 1816906 | 1804967 | 1804542 | - | *-* | *PA14_20960* | isomerase | 195 | 11 | 20 | 68 | 179 | 105 | 0 |
| 1691 | 1816906 | 1806220 | 1804964 | - | *cyp23* | *PA14_20970* | cytochrome P450 | 274 | 17 | 39 | 107 | 260 | 159 | 7.8332E-152 |
| 1692 | 1816906 | 1807127 | 1806213 | - | *-* | *PA14_20980* | short chain dehydrogenas | 451 | 16 | 56 | 165 | 359 | 239 | 0 |
| 1693 | 1816906 | 1808457 | 1807129 | - | *-* | *PA14_21000* | hypothetical protein | 262 | 17 | 44 | 107 | 213 | 155 | 2.0973E-123 |
| 1694 | 1816906 | 1809620 | 1808454 | - | *-* | *PA14_21010* | FAD-dependent monooxygenase | 246 | 10 | 35 | 90 | 185 | 127 | 0 |
| 1695 | 1816906 | 1816675 | 1809617 | - | *-* | *PA14_21020* | non-ribosomal peptide synthetase | 178 | 15 | 33 | 76 | 136 | 89 | 2.36197E-32 |
| 1696 | 1816906 | 1817157 | 1817762 | + | *-* | *PA14_21030* | ATP-dependent Clp protease proteolytic subunit | 572 | 132 | 259 | 618 | 630 | 537 | 0.002328387 |
| 1697 | - | 1818994 | 1818107 | - | *-* | *PA14_21040* | hypothetical protein | 38 | 14 | 24 | 50 | 41 | 38 | 0.011702045 |
| 1698 | - | 1820782 | 1819004 | - | *-* | *PA14_21050* | short chain dehydrogenase | 16 | 16 | 13 | 19 | 15 | 18 | 1 |
| 1699 | - | 1821654 | 1820779 | - | *-* | *PA14_21060* | hypothetical protein | 12 | 12 | 12 | 13 | 13 | 14 | 1 |
| 1700 | - | 1822080 | 1822778 | + | *-* | *PA14_21070* | MerR family transcriptional regulator | 38 | 27 | 34 | 33 | 27 | 28 | 1 |
| 1701 | - | 1823700 | 1822786 | - | *-* | *PA14_21080* | LysR family transcriptional activator | 16 | 15 | 14 | 17 | 12 | 16 | 1 |
| 1702 | - | 1823789 | 1824244 | + | *-* | *PA14_21090* | hypothetical protein | 4 | 4 | 4 | 5 | 5 | 7 | 1 |
| 1703 | - | 1824476 | 1826554 | + | *plcN* | *PA14_21110* | non-hemolytic phospholipase C | 6 | 6 | 6 | 7 | 5 | 5 | 1 |
| 1704 | - | 1827112 | 1826612 | - | *-* | *PA14_21120* | hypothetical protein | 11 | 10 | 10 | 9 | 10 | 10 | 1 |
| 1705 | - | 1827906 | 1827178 | - | *-* | *PA14_21130* | outer membrane lipoprotein | 25 | 18 | 22 | 20 | 21 | 17 | 1 |
| 1706 | - | 1828783 | 1828013 | - | *-* | *PA14_21140* | ABC transporter permease | 17 | 20 | 16 | 21 | 20 | 13 | 1 |
| 1707 | - | 1829613 | 1828780 | - | *-* | *PA14_21150* | ABC transporter permease | 12 | 16 | 14 | 12 | 15 | 11 | 1 |
| 1708 | - | 1830410 | 1829607 | - | *-* | *PA14_21160* | ABC transporter ATP-binding protein | 59 | 61 | 55 | 50 | 52 | 46 | 1 |
| 1709 | - | 1831413 | 1832303 | + | *-* | *PA14_21180* | 3-hydroxyisobutyrate dehydrogenase | 42 | 41 | 39 | 36 | 43 | 31 | 1 |
| 1710 | - | 1831414 | 1830407 | - | *-* | *PA14_21175* | hypothetical protein | 38 | 51 | 37 | 37 | 41 | 32 | 1 |
| 1711 | - | 1834664 | 1832307 | - | *-* | *PA14_21190* | hypothetical protein | 31 | 31 | 37 | 43 | 36 | 41 | 1 |
| 1712 | - | 1834819 | 1836474 | + | *-* | *PA14_21210* | hypothetical protein | 67 | 67 | 84 | 48 | 54 | 75 | 1 |
| 1713 | - | 1836998 | 1836543 | - | *-* | *PA14_21220* | hypothetical protein | 2414 | 2570 | 2646 | 1105 | 1165 | 2305 | 1 |
| 1714 | - | 1840133 | 1837281 | - | *hepA* | *PA14_21230* | ATP-dependent helicase HepA | 45 | 56 | 53 | 51 | 50 | 34 | 1 |
| 1715 | - | 1840369 | 1840677 | + | *-* | *PA14_21240* | hypothetical protein | 170 | 196 | 191 | 239 | 241 | 240 | 1 |
| 1716 | - | 1840731 | 1841333 | + | *alkB* | *PA14_21250* | hypothetical protein | 77 | 69 | 80 | 70 | 73 | 71 | 1 |
| 1717 | - | 1841740 | 1841853 | + | *-* | *PA14_21260* | hypothetical protein | 2674 | 1368 | 3463 | 1900 | 1433 | 1957 | 1 |
| 1718 | - | 1842061 | 1844055 | + | *-* | *PA14_21280* | hypothetical protein | 41 | 36 | 47 | 41 | 45 | 43 | 1 |
| 1719 | - | 1844273 | 1845130 | + | *-* | *PA14_21290* | hypothetical protein | 34 | 30 | 29 | 33 | 36 | 39 | 1 |
| 1720 | - | 1846286 | 1845111 | - | *-* | *PA14_21300* | MFS transporte | 12 | 13 | 13 | 13 | 13 | 16 | 1 |
| 1721 | - | 1846907 | 1846437 | - | *phaJ1* | *PA14_21310* | hypothetical protein | 53 | 84 | 52 | 56 | 79 | 53 | 1 |
| 1722 | - | 1847854 | 1846904 | - | *-* | *PA14_21320* | hypothetical protein | 26 | 25 | 26 | 20 | 22 | 19 | 1 |
| 1723 | - | 1848101 | 1849789 | + | *fadD2* | *PA14_21340* | long-chain-fatty-acid--CoA ligase | 64 | 79 | 66 | 108 | 85 | 84 | 1 |
| 1724 | - | 1850024 | 1851712 | + | *fadD1* | *PA14_21370* | long-chain-fatty-acid--CoA ligase | 99 | 118 | 93 | 90 | 93 | 78 | 1 |
| 1725 | - | 1851742 | 1852044 | + | *-* | *PA14_21380* | hypothetical protein | 17 | 18 | 22 | 37 | 20 | 24 | 1 |
| 1726 | - | 1852220 | 1856200 | + | *hrpA* | *PA14_21400* | ATP-dependent helicase | 61 | 56 | 60 | 57 | 55 | 52 | 1 |
| 1727 | - | 1857703 | 1856273 | - | *phoA* | *PA14_21410* | alkaline phosphatase | 7 | 7 | 8 | 7 | 6 | 5 | 1 |
| 1728 | - | 1857908 | 1858345 | + | *-* | *PA14_21440* | HIT family protein | 42 | 50 | 43 | 40 | 33 | 37 | 1 |
| 1729 | - | 1858574 | 1860640 | + | *-* | *PA14_21450* | hypothetical protein | 20 | 17 | 14 | 14 | 21 | 12 | 1 |
| 1730 | - | 1860650 | 1861465 | + | *-* | *PA14_21460* | hypothetical protein | 11 | 7 | 8 | 8 | 9 | 6 | 0.674979992 |
| 1731 | - | 1861405 | 1861983 | + | *-* | *PA14_21470* | hypothetical protein | 8 | 4 | 5 | 5 | 8 | 6 | 0.347673727 |
| 1732 | - | 1862506 | 1863060 | + | *-* | *PA14_21480* | hypothetical protein | 10 | 10 | 6 | 8 | 13 | 7 | 1 |
| 1733 | - | 1863075 | 1865723 | + | *-* | *PA14_21490* | hypothetical protein | 58 | 53 | 52 | 58 | 60 | 61 | 1 |
| 1734 | - | 1866175 | 1865762 | - | *-* | *PA14_21510* | hypothetical protein | 49 | 40 | 49 | 42 | 38 | 47 | 1 |
| 1735 | - | 1866255 | 1866758 | + | *-* | *PA14_21520* | hypothetical protein | 92 | 45 | 44 | 45 | 41 | 104 | 0.452603163 |
| 1736 | - | 1867277 | 1866762 | - | *-* | *PA14_21530* | ankyrin domain-containing protein | 179 | 31 | 34 | 34 | 28 | 168 | 6.87207E-17 |
| 1737 | - | 1867837 | 1868889 | + | *-* | *PA14_21540* | 3-oxoacyl-ACP synthase | 81 | 76 | 67 | 72 | 62 | 77 | 1 |
| 1738 | - | 1868892 | 1869503 | + | *-* | *PA14_21550* | RNA polymerase ECF-subfamily sigma-70 factor | 105 | 110 | 103 | 115 | 96 | 102 | 1 |
| 1739 | - | 1869497 | 1869736 | + | *-* | *PA14_21560* | hypothetical protein | 128 | 114 | 120 | 123 | 103 | 102 | 1 |
| 1740 | - | 1870142 | 1870456 | + | *-* | *PA14_21570* | hypothetical protein | 291 | 92 | 190 | 52 | 24 | 40 | 0.01350491 |
| 1741 | - | 1870475 | 1871329 | + | *-* | *PA14_21580* | hypothetical protein | 210 | 66 | 147 | 81 | 23 | 43 | 0.07173263 |
| 1742 | - | 1871326 | 1872060 | + | *-* | *PA14_21590* | hypothetical protein | 25 | 11 | 21 | 7 | 5 | 7 | 0.05276977 |
| 1743 | - | 1872063 | 1872653 | + | *-* | *PA14_21600* | hypothetical protein | 24 | 11 | 19 | 6 | 4 | 7 | 0.050479413 |
| 1744 | - | 1872900 | 1874216 | + | *oprO* | *PA14_21610* | pyrophosphate-specific outer membrane porin OprO precursor | 9 | 8 | 10 | 9 | 7 | 7 | 1 |
| 1745 | - | 1874675 | 1875997 | + | *oprP* | *PA14_21620* | phosphate-specific outer membrane porin OprP precursor | 3 | 3 | 3 | 5 | 4 | 3 | 1 |
| 1746 | - | 1876166 | 1876465 | + | *-* | *PA14_21630* | hypothetical protein | 564 | 549 | 995 | 416 | 366 | 698 | 1 |
| 1747 | - | 1876590 | 1877402 | + | *-* | *PA14_21640* | short chain dehydrogenase | 57 | 46 | 78 | 42 | 52 | 62 | 1 |
| 1748 | - | 1877635 | 1878057 | + | *-* | *PA14_21650* | hypothetical protein | 14 | 17 | 23 | 17 | 16 | 15 | 1 |
| 1749 | - | 1878054 | 1878383 | + | *-* | *PA14_21660* | hypothetical protein | 32 | 48 | 45 | 45 | 48 | 40 | 1 |
| 1750 | - | 1878700 | 1879299 | + | *-* | *PA14_21680* | hypothetical protein | 32 | 42 | 42 | 43 | 39 | 34 | 1 |
| 1751 | - | 1878749 | 1878462 | - | *-* | *PA14_21670* | hypothetical protein | 49 | 96 | 91 | 82 | 88 | 79 | 0.360825712 |
| 1752 | - | 1879368 | 1883714 | + | *-* | *PA14_21690* | ATP-dependent DNA helicase | 68 | 67 | 76 | 70 | 73 | 73 | 1 |
| 1753 | - | 1887201 | 1883722 | - | *-* | *PA14_21700* | two-component sensor | 108 | 70 | 92 | 71 | 66 | 68 | 1 |
| 1754 | - | 1887467 | 1888054 | + | *-* | *PA14_21710* | hypothetical protein | 123 | 89 | 119 | 96 | 85 | 81 | 1 |
| 1755 | - | 1888121 | 1888975 | + | *-* | *PA14_21720* | AraC family transcriptional regulator | 18 | 14 | 18 | 21 | 19 | 13 | 1 |
| 1756 | - | 1889072 | 1891237 | + | *-* | *PA14_21730* | TonB-dependent receptor | 10 | 15 | 17 | 16 | 15 | 8 | 1 |
| 1757 | - | 1891412 | 1893286 | + | *-* | *PA14_21750* | hypothetical protein | 41 | 33 | 37 | 35 | 36 | 30 | 1 |
| 1758 | - | 1893556 | 1893347 | - | *capB* | *PA14_21760* | cold acclimation protein B | 685 | 511 | 494 | 347 | 423 | 349 | 1 |
| 1759 | - | 1894085 | 1893771 | - | *-* | *PA14_21770* | DMT family permease | 29 | 26 | 32 | 32 | 22 | 28 | 1 |
| 1760 | - | 1895160 | 1894225 | - | *-* | *PA14_21780* | transporter | 15 | 17 | 16 | 18 | 14 | 15 | 1 |
| 1761 | - | 1896167 | 1895247 | - | *rdgC* | *PA14_21790* | recombination associated protein | 28 | 35 | 41 | 36 | 34 | 31 | 1 |
| 1762 | - | 1896332 | 1896404 | + | *-* | *PA14_21800* | Val tRNA | 151 | 90 | 168 | 141 | 86 | 58 | 0.382662007 |
| 1763 | - | 1896424 | 1896497 | + | *-* | *PA14_21810* | Asp tRNA | 184 | 119 | 182 | 155 | 107 | 71 | 0.853730745 |
| 1764 | - | 1896757 | 1897518 | + | *-* | *PA14_21820* | peptidyl-prolyl cis-trans isomerase, FkbP-type | 128 | 123 | 111 | 80 | 103 | 87 | 1 |

|  | A | B | C | D | E | F | G | H | I | J | K | L | M | N |
| --- | --- | --- | --- | --- | --- | --- | --- | --- | --- | --- | --- | --- | --- | --- |
| 1765 | - | 1897888 | 1898319 | + | *-* | *PA14_21830* | hypothetical protein | 403 | 310 | 435 | 431 | 326 | 364 | 1 |
| 1766 | - | 1899053 | 1898307 | - | *-* | *PA14_21840* | hypothetical protein | 171 | 166 | 202 | 179 | 136 | 158 | 1 |
| 1767 | - | 1899157 | 1899468 | + | *-* | *PA14_21850* | transcriptional regulator | 200 | 173 | 217 | 183 | 160 | 190 | 1 |
| 1768 | - | 1899826 | 1900305 | + | *-* | *PA14_21860* | hypothetical protein | 171 | 175 | 203 | 185 | 142 | 173 | 1 |
| 1769 | - | 1902124 | 1900319 | - | *-* | *PA14_21870* | EAL domain/GGDEF domain-containing protein | 23 | 23 | 28 | 28 | 21 | 24 | 1 |
| 1770 | - | 1904259 | 1902163 | - | *tsp* | *PA14_21880* | periplasmic tail-specific protease | 375 | 389 | 384 | 353 | 378 | 423 | 1 |
| 1771 | - | 1904389 | 1905351 | + | *-* | *PA14_21890* | oxidoreductase | 263 | 245 | 276 | 264 | 262 | 266 | 1 |
| 1772 | - | 1905996 | 1905418 | - | *-* | *PA14_21900* | HAD-superfamily hydrolase | 88 | 88 | 88 | 103 | 90 | 99 | 1 |
| 1773 | - | 1907063 | 1906068 | - | *-* | *PA14_21910* | ABC transporter ATP-binding protein | 20 | 20 | 20 | 26 | 20 | 24 | 1 |
| 1774 | - | 1907863 | 1907066 | - | *-* | *PA14_21920* | ABC transporter permease | 9 | 11 | 13 | 13 | 11 | 13 | 1 |
| 1775 | - | 1908689 | 1907850 | - | *-* | *PA14_21930* | ABC transporter permease | 12 | 11 | 11 | 13 | 9 | 13 | 1 |
| 1776 | - | 1909495 | 1908686 | - | *-* | *PA14_21940* | hypothetical protein | 15 | 16 | 16 | 18 | 12 | 16 | 1 |
| 1777 | - | 1910608 | 1909550 | - | *-* | *PA14_21960* | hypothetical protein | 37 | 40 | 41 | 43 | 36 | 48 | 1 |
| 1778 | - | 1910972 | 1911688 | + | *-* | *PA14_21970* | transcriptional regulator | 31 | 33 | 42 | 52 | 33 | 35 | 1 |
| 1779 | - | 1911754 | 1912305 | + | *-* | *PA14_21980* | hypothetical protein | 57 | 55 | 58 | 56 | 54 | 46 | 1 |
| 1780 | - | 1913637 | 1912348 | - | *-* | *PA14_21990* | aminopeptidase 2 | 61 | 59 | 57 | 49 | 50 | 52 | 1 |
| 1781 | - | 1914441 | 1913806 | - | *rluA* | *PA14_22000* | pseudouridine synthase | 18 | 21 | 22 | 27 | 26 | 23 | 1 |
| 1782 | - | 1914799 | 1914545 | - | *minE* | *PA14_22010* | cell division topological specificity factor MinE | 92 | 139 | 98 | 87 | 85 | 59 | 1 |
| 1783 | - | 1915611 | 1914796 | - | *minD* | *PA14_22020* | cell division inhibitor MinD | 102 | 107 | 99 | 100 | 97 | 86 | 1 |
| 1784 | - | 1916464 | 1915673 | - | *minC* | *PA14_22040* | septum formation inhibitor | 46 | 35 | 48 | 44 | 34 | 35 | 1 |
| 1785 | - | 1916624 | 1917562 | + | *htrB* | *PA14_22050* | lipid A biosynthesis lauroyl acyltransferase | 21 | 18 | 25 | 21 | 16 | 16 | 1 |
| 1786 | - | 1917630 | 1918799 | + | *-* | *PA14_22060* | hypothetical protein | 68 | 65 | 63 | 75 | 67 | 66 | 1 |
| 1787 | - | 1919495 | 1918863 | - | *-* | *PA14_22075* | pirin-related protein | 144 | 154 | 167 | 151 | 126 | 118 | 1 |
| 1788 | - | 1922695 | 1922000 | - | *-* | *PA14_22080* | resolvase | 147 | 115 | 102 | 75 | 99 | 97 | 1 |
| 1789 | 1924465 | 1924132 | 1923032 | - | *-* | *PA14_22090* | hypothetical protein | 43 | 45 | 42 | 46 | 36 | 38 | 1 |
| 1790 | - | 1927303 | 1926578 | - | *-* | *PA14_22100* | hypothetical protein | 5 | 4 | 3 | 4 | 5 | 5 | 1 |
| 1791 | - | 1928419 | 1927460 | - | *-* | *PA14_22110* | hypothetical protein | 5 | 5 | 5 | 5 | 6 | 4 | 1 |
| 1792 | - | 1931366 | 1929042 | - | *-* | *PA14_22120* | hypothetical protein | 5 | 3 | 4 | 5 | 3 | 4 | 1 |
| 1793 | - | 1932050 | 1931382 | - | *-* | *PA14_22130* | hypothetical protein | 1 | 0 | 1 | 1 | 0 | 0 | 0.002850335 |
| 1794 | - | 1933820 | 1933020 | - | *-* | *PA14_22140* | hypothetical protein | 5 | 4 | 4 | 5 | 5 | 5 | 1 |
| 1795 | - | 1934861 | 1934355 | - | *-* | *PA14_22160* | hypothetical protein | 0 | 0 | 0 | 0 | 0 | 1 | 1 |
| 1796 | - | 1935117 | 1934977 | - | *-* | *PA14_22180* | hypothetical protein | 8 | 8 | 7 | 7 | 6 | 4 | 1 |
| 1797 | - | 1935500 | 1935114 | - | *-* | *PA14_22190* | hypothetical protein | 14 | 14 | 11 | 12 | 13 | 14 | 1 |
| 1798 | - | 1935905 | 1935570 | - | *-* | *PA14_22210* | hypothetical protein | 6 | 5 | 4 | 5 | 5 | 6 | 1 |
| 1799 | - | 1936104 | 1935907 | - | *-* | *PA14_22220* | hypothetical protein | 7 | 7 | 4 | 3 | 6 | 5 | 1 |
| 1800 | - | 1936868 | 1936671 | - | *-* | *PA14_22230* | hypothetical protein | 5 | 6 | 2 | 3 | 2 | 2 | 1 |
| 1801 | - | 1937691 | 1937290 | - | *-* | *PA14_22240* | hypothetical protein | 6 | 6 | 4 | 10 | 7 | 7 | 1 |
| 1802 | - | 1938417 | 1937791 | - | *-* | *PA14_22250* | hypothetical protein | 0 | 1 | 2 | 1 | 0 | 1 | 1 |
| 1803 | - | 1938800 | 1938450 | - | *-* | *PA14_22260* | hypothetical protein | 2 | 1 | 0 | 1 | 1 | 1 | 1 |
| 1804 | - | 1941291 | 1939714 | - | *-* | *PA14_22270* | recombinase | 42 | 34 | 36 | 31 | 27 | 30 | 1 |
| 1805 | - | 1941585 | 1941322 | - | *-* | *PA14_22280* | pirin-related protein | 28 | 31 | 29 | 23 | 20 | 24 | 1 |
| 1806 | - | 1942528 | 1941725 | - | *vacJ* | *PA14_22290* | hypothetical protein | 63 | 62 | 58 | 82 | 70 | 70 | 1 |
| 1807 | - | 1943893 | 1942532 | - | *-* | *PA14_22310* | hypothetical protein | 45 | 39 | 42 | 53 | 39 | 53 | 1 |
| 1808 | - | 1944257 | 1944036 | - | *-* | *PA14_22320* | hypothetical protein | 22 | 7 | 8 | 13 | 9 | 76 | 0.001722686 |
| 1809 | - | 1944551 | 1945408 | + | *-* | *PA14_22330* | glycine betaine-binding protein | 19 | 15 | 16 | 20 | 14 | 22 | 1 |
| 1810 | - | 1945626 | 1945937 | + | *-* | *PA14_22340* | hypothetical protein | 142 | 133 | 94 | 85 | 63 | 81 | 1 |
| 1811 | - | 1945934 | 1947589 | + | *actP* | *PA14_22350* | acetate permease | 91 | 82 | 67 | 61 | 56 | 45 | 1 |
| 1812 | - | 1947679 | 1949478 | + | *-* | *PA14_22370* | hypothetical protein | 87 | 69 | 68 | 76 | 64 | 58 | 1 |
| 1813 | - | 1949475 | 1950101 | + | *-* | *PA14_22380* | DNA polymerase III subunit epsilon | 35 | 23 | 26 | 20 | 17 | 17 | 1 |
| 1814 | - | 1950210 | 1950368 | + | *-* | *PA14_22400* | hypothetical protein | 84 | 177 | 147 | 188 | 158 | 149 | 0.23199809 |
| 1815 | - | 1950645 | 1951769 | + | *-* | *PA14_22410* | hypothetical protein | 34 | 39 | 33 | 41 | 34 | 33 | 1 |
| 1816 | - | 1952068 | 1951802 | - | *-* | *PA14_22420* | hypothetical protein | 26 | 34 | 25 | 30 | 34 | 30 | 1 |
| 1817 | - | 1954182 | 1952350 | - | *-* | *PA14_22440* | ABC transporter ATP-binding protein/permease | 115 | 118 | 112 | 113 | 124 | 123 | 1 |
| 1818 | - | 1954808 | 1954245 | - | *ppiA* | *PA14_22450* | peptidyl-prolyl cis-trans isomerase A | 148 | 200 | 156 | 136 | 166 | 153 | 1 |
| 1819 | - | 1955617 | 1954790 | - | *-* | *PA14_22460* | alpha/beta hydrolase | 90 | 100 | 100 | 89 | 96 | 99 | 1 |
| 1820 | - | 1956553 | 1955624 | - | *-* | *PA14_22470* | LysR family transcriptional regulator | 105 | 99 | 105 | 95 | 105 | 104 | 1 |
| 1821 | - | 1956753 | 1957070 | + | *-* | *PA14_22480* | hypothetical protein | 141 | 187 | 171 | 156 | 147 | 139 | 1 |
| 1822 | - | 1957217 | 1957858 | + | *acpD* | *PA14_22490* | ACP phosphodieterase | 3 | 2 | 3 | 4 | 2 | 3 | 1 |
| 1823 | - | 1958847 | 1958113 | - | *-* | *PA14_22500* | protein-disulfide isomerase | 9 | 9 | 9 | 10 | 9 | 11 | 1 |
| 1824 | - | 1959333 | 1958941 | - | *-* | *PA14_22510* | hypothetical protein | 15 | 13 | 11 | 13 | 11 | 11 | 1 |
| 1825 | - | 1960494 | 1959439 | - | *-* | *PA14_22520* | hypothetical protein | 11 | 10 | 9 | 10 | 8 | 9 | 1 |
| 1826 | - | 1961041 | 1960424 | - | *-* | *PA14_22530* | glutathione S-transferase | 27 | 30 | 27 | 25 | 21 | 23 | 1 |
| 1827 | - | 1961671 | 1961117 | - | *-* | *PA14_22540* | flavodoxin | 47 | 44 | 44 | 46 | 44 | 50 | 1 |
| 1828 | - | 1961914 | 1962726 | + | *-* | *PA14_22550* | LysR family transcriptional regulator | 50 | 52 | 49 | 56 | 51 | 54 | 1 |
| 1829 | - | 1963696 | 1962803 | - | *-* | *PA14_22560* | permease | 58 | 53 | 55 | 51 | 50 | 40 | 1 |
| 1830 | - | 1964041 | 1963706 | - | *csaA* | *PA14_22570* | CsaA protein | 61 | 48 | 54 | 41 | 44 | 29 | 1 |
| 1831 | - | 1964870 | 1964106 | - | *-* | *PA14_22580* | AraC family transcriptional regulator | 35 | 28 | 34 | 28 | 24 | 26 | 1 |
| 1832 | - | 1965017 | 1965829 | + | *-* | *PA14_22590* | hypothetical protein | 7 | 7 | 7 | 8 | 7 | 5 | 1 |
| 1833 | - | 1965826 | 1966881 | + | *-* | *PA14_22600* | glycosyl transferase family protein | 14 | 19 | 21 | 20 | 15 | 14 | 1 |
| 1834 | - | 1968262 | 1966871 | - | *cyaB* | *PA14_22620* | hypothetical protein | 27 | 29 | 40 | 35 | 27 | 25 | 1 |
| 1835 | - | 1968796 | 1968479 | - | *-* | *PA14_22630* | hypothetical protein | 27 | 39 | 34 | 28 | 31 | 29 | 1 |
| 1836 | - | 1968939 | 1969952 | + | *-* | *PA14_22640* | AraC family transcriptional regulator | 88 | 89 | 93 | 89 | 75 | 80 | 1 |
| 1837 | - | 1970619 | 1969975 | - | *-* | *PA14_22650* | ABC transporter | 223 | 239 | 205 | 239 | 224 | 222 | 1 |
| 1838 | - | 1971554 | 1970616 | - | *-* | *PA14_22660* | hypothetical protein | 125 | 114 | 112 | 132 | 116 | 118 | 1 |
| 1839 | - | 1972350 | 1971556 | - | *-* | *PA14_22670* | ABC transporter ATP-binding protein | 140 | 120 | 140 | 153 | 143 | 127 | 1 |
| 1840 | - | 1973492 | 1972347 | - | *-* | *PA14_22680* | ABC transporter permease | 132 | 133 | 144 | 158 | 126 | 132 | 1 |
| 1841 | - | 1975013 | 1973559 | - | *trkH* | *PA14_22690* | potassium uptake protein TrkH | 24 | 21 | 25 | 25 | 20 | 26 | 1 |
| 1842 | - | 1975171 | 1975542 | + | *-* | *PA14_22700* | ferredoxin | 5 | 3 | 6 | 7 | 5 | 4 | 1 |
| 1843 | - | 1975607 | 1976167 | + | *-* | *PA14_22710* | hypothetical protein | 90 | 102 | 113 | 102 | 80 | 69 | 1 |
| 1844 | - | 1976624 | 1976148 | - | *-* | *PA14_22720* | hypothetical protein | 81 | 95 | 97 | 88 | 86 | 74 | 1 |
| 1845 | - | 1978005 | 1976668 | - | *-* | *PA14_22730* | two-component sensor | 43 | 43 | 50 | 47 | 40 | 38 | 1 |
| 1846 | - | 1978560 | 1978120 | - | *-* | *PA14_22740* | hypothetical protein | 49 | 115 | 61 | 86 | 18 | 24 | 0.085032199 |
| 1847 | - | 1979366 | 1978689 | - | *cpxR* | *PA14_22760* | two-component response regulator | 69 | 60 | 63 | 69 | 60 | 63 | 1 |
| 1848 | - | 1979745 | 1979377 | - | *-* | *PA14_22770* | hypothetical protein | 122 | 133 | 128 | 143 | 125 | 122 | 1 |

|  | A | B | C | D | E | F | G | H | I | J | K | L | M | N |
| --- | --- | --- | --- | --- | --- | --- | --- | --- | --- | --- | --- | --- | --- | --- |
| 1849 | - | 1980220 | 1979921 | - | *yciI* | *PA14_22780* | YciI-like protein | 114 | 135 | 128 | 136 | 126 | 120 | 1 |
| 1850 | - | 1980809 | 1980222 | - | *yciB* | *PA14_22800* | intracellular septation protein A | 106 | 83 | 111 | 86 | 85 | 89 | 1 |
| 1851 | - | 1981024 | 1981911 | + | *-* | *PA14_22820* | PHP domain-containing protein | 74 | 57 | 82 | 74 | 62 | 84 | 1 |
| 1852 | - | 1981908 | 1982537 | + | *-* | *PA14_22830* | SUA5/yciO/yrdC family:Sua5/YciO/YrdC/YwlC family protein | 91 | 95 | 97 | 110 | 97 | 118 | 1 |
| 1853 | - | 1982710 | 1983462 | + | *-* | *PA14_22840* | hypothetical protein | 71 | 68 | 79 | 65 | 60 | 67 | 1 |
| 1854 | - | 1983607 | 1984599 | + | *-* | *PA14_22860* | transcriptional regulator | 62 | 37 | 60 | 50 | 40 | 46 | 1 |
| 1855 | - | 1984681 | 1985211 | + | *-* | *PA14_22870* | hypothetical protein | 18 | 25 | 23 | 22 | 27 | 18 | 1 |
| 1856 | - | 1985296 | 1985508 | + | *-* | *PA14_22880* | Fe-S protein | 101 | 103 | 98 | 100 | 93 | 119 | 1 |
| 1857 | - | 1986519 | 1985515 | - | *gapA* | *PA14_22890* | glyceraldehyde-3-phosphate dehydrogenase | 108 | 191 | 149 | 190 | 164 | 145 | 1 |
| 1858 | - | 1986649 | 1988475 | + | *edd* | *PA14_22910* | phosphogluconate dehydratase | 64 | 180 | 88 | 155 | 195 | 89 | 0.321350893 |
| 1859 | - | 1988578 | 1989573 | + | *glk* | *PA14_22930* | glucokinase | 103 | 196 | 127 | 165 | 216 | 112 | 1 |
| 1860 | - | 1989608 | 1990336 | + | *gltR* | *PA14_22940* | two-component response regulator GltR | 51 | 116 | 62 | 85 | 113 | 58 | 0.260298623 |
| 1861 | - | 1990360 | 1991781 | + | *-* | *PA14_22960* | two-component sensor | 25 | 63 | 37 | 57 | 61 | 35 | 0.104266321 |
| 1862 | - | 1992306 | 1993568 | + | *-* | *PA14_22980* | sugar ABC transporter substrate-binding protein | 10 | 679 | 387 | 547 | 594 | 472 | 0 |
| 1863 | - | 1993669 | 1994601 | + | *-* | *PA14_22990* | ABC sugar transporter permease | 11 | 183 | 101 | 145 | 153 | 109 | 3.4879E-157 |
| 1864 | - | 1994594 | 1995439 | + | *-* | *PA14_23000* | ABC sugar transporter permease | 14 | 178 | 88 | 128 | 131 | 105 | 6.23215E-97 |
| 1865 | - | 1995472 | 1996632 | + | *gltK* | *PA14_23010* | ABC transporter ATP-binding protein | 9 | 504 | 263 | 373 | 404 | 311 | 0 |
| 1866 | - | 1996676 | 1998040 | + | *oprB* | *PA14_23030* | glucose/carbohydrate outer membrane porin OprB precursor | 65 | 926 | 502 | 712 | 719 | 599 | 5.54754E-82 |
| 1867 | - | 1998255 | 1999106 | + | *-* | *PA14_23050* | hypothetical protein | 335 | 353 | 399 | 444 | 356 | 368 | 1 |
| 1868 | - | 1999972 | 1999115 | - | *hexR* | *PA14_23060* | DNA-binding transcriptional regulator HexR | 60 | 68 | 64 | 77 | 64 | 58 | 1 |
| 1869 | - | 2000159 | 2001628 | + | *zwf* | *PA14_23070* | glucose-6-phosphate 1-dehydrogenase | 49 | 125 | 59 | 134 | 155 | 78 | 0.158443239 |
| 1870 | - | 2001615 | 2002331 | + | *pgl* | *PA14_23080* | 6-phosphogluconolactonase | 44 | 147 | 56 | 131 | 181 | 72 | 0.001697282 |
| 1871 | - | 2002349 | 2003011 | + | *-* | *PA14_23090* | keto-hydroxyglutarate-aldolase/keto-deoxy-phosphogluconate aldolase | 77 | 113 | 99 | 112 | 112 | 81 | 1 |
| 1872 | - | 2003538 | 2003101 | - | *-* | *PA14_23100* | hypothetical protein | 38 | 34 | 34 | 45 | 32 | 34 | 1 |
| 1873 | - | 2003686 | 2004846 | + | *-* | *PA14_23110* | hypothetical protein | 51 | 47 | 59 | 62 | 47 | 55 | 1 |
| 1874 | - | 2004939 | 2005316 | + | *-* | *PA14_23120* | hypothetical protein | 98 | 90 | 99 | 104 | 86 | 87 | 1 |
| 1875 | - | 2006346 | 2005423 | - | *-* | *PA14_23130* | sensory box GGDEF domain-containing protein | 57 | 45 | 52 | 46 | 41 | 44 | 1 |
| 1876 | - | 2007789 | 2006575 | - | *gltS* | *PA14_23160* | sodium/glutamate symporter | 5 | 6 | 5 | 5 | 4 | 4 | 1 |
| 1877 | - | 2008763 | 2007828 | - | *-* | *PA14_23170* | formimidoylglutamase | 22 | 22 | 25 | 25 | 17 | 21 | 1 |
| 1878 | - | 2009507 | 2008779 | - | *-* | *PA14_23190* | transcriptional regulator | 11 | 8 | 11 | 10 | 8 | 7 | 1 |
| 1879 | - | 2010406 | 2009666 | - | *-* | *PA14_23200* | short chain dehydrogenase | 89 | 86 | 94 | 85 | 82 | 84 | 1 |
| 1880 | - | 2011151 | 2010471 | - | *-* | *PA14_23210* | phosphoglycolate phosphatase | 130 | 132 | 143 | 130 | 127 | 116 | 1 |
| 1881 | - | 2011846 | 2011148 | - | *ubiG* | *PA14_23220* | 3-demethylubiquinone-9 3-methyltransferase | 202 | 165 | 222 | 174 | 158 | 171 | 1 |
| 1882 | - | 2013331 | 2011997 | - | *-* | *PA14_23240* | N-ethylammeline chlorohydrolase | 44 | 38 | 46 | 40 | 38 | 38 | 1 |
| 1883 | - | 2013441 | 2014517 | + | *mtnA* | *PA14_23250* | methylthioribose-1-phosphate isomerase | 64 | 58 | 68 | 68 | 63 | 53 | 1 |
| 1884 | - | 2014754 | 2017519 | + | *gyrA* | *PA14_23260* | DNA gyrase subunit A | 285 | 292 | 294 | 283 | 297 | 257 | 1 |
| 1885 | - | 2017607 | 2018692 | + | *serC* | *PA14_23270* | phosphoserine aminotransferase | 270 | 276 | 277 | 257 | 285 | 256 | 1 |
| 1886 | - | 2018692 | 2019789 | + | *pheA* | *PA14_23280* | chorismate mutase | 257 | 259 | 246 | 272 | 300 | 265 | 1 |
| 1887 | - | 2019858 | 2020967 | + | *hisC2* | *PA14_23290* | histidinol-phosphate aminotransferase | 117 | 99 | 109 | 88 | 121 | 93 | 1 |
| 1888 | - | 2020960 | 2023200 | + | *-* | *PA14_23310* | bifunctional cyclohexadienyl dehydrogenase/ 3-phosphoshikimate 1-carboxyvinyltransferase | 163 | 169 | 151 | 131 | 181 | 121 | 1 |
| 1889 | - | 2023200 | 2023889 | + | *cmk* | *PA14_23320* | cytidylate kinase | 166 | 187 | 167 | 137 | 180 | 143 | 1 |
| 1890 | - | 2024157 | 2025836 | + | *rpsA* | *PA14_23330* | 30S ribosomal protein S1 | 599 | 641 | 623 | 473 | 536 | 388 | 1 |
| 1891 | - | 2025973 | 2026257 | + | *ihfB* | *PA14_23340* | integration host factor subunit beta | 3265 | 3404 | 3944 | 4185 | 2514 | 2683 | 1 |
| 1892 | - | 2026291 | 2026590 | + | *orfA* | *PA14_23350* | hypothetical protein | 326 | 396 | 263 | 314 | 367 | 259 | 1 |
| 1893 | - | 2026846 | 2027928 | + | *wzz* | *PA14_23360* | O-antigen chain length regulator | 59 | 48 | 50 | 40 | 49 | 43 | 1 |
| 1894 | - | 2028084 | 2029220 | + | *orfK* | *PA14_23370* | UDP-N-acetylglucosamine 2-epimerase | 242 | 193 | 172 | 162 | 252 | 183 | 1 |
| 1895 | - | 2029301 | 2030569 | + | *orfH* | *PA14_23380* | UDP-N-acetyl-D-mannosaminuronate dehydrogenase | 159 | 219 | 129 | 124 | 165 | 121 | 1 |
| 1896 | - | 2030647 | 2031909 | + | *orfE* | *PA14_23390* | polysaccharide biosynthesis protein | 53 | 44 | 46 | 46 | 71 | 54 | 1 |
| 1897 | - | 2031887 | 2033236 | + | *-* | *PA14_23400* | hypothetical protein | 92 | 70 | 73 | 82 | 133 | 108 | 1 |
| 1898 | - | 2033233 | 2034075 | + | *orfJ* | *PA14_23410* | glycosyl transferase family protein | 90 | 98 | 70 | 76 | 112 | 88 | 1 |
| 1899 | - | 2034189 | 2036357 | + | *-* | *PA14_23420* | zinc-binding dehydrogenase | 214 | 267 | 149 | 164 | 254 | 173 | 1 |
| 1900 | - | 2036366 | 2038018 | + | *-* | *PA14_23430* | heparinase | 135 | 143 | 101 | 94 | 163 | 120 | 1 |
| 1901 | - | 2038087 | 2039199 | + | *orfL* | *PA14_23440* | group 1 glycosyl transferase | 129 | 145 | 87 | 99 | 166 | 125 | 1 |
| 1902 | - | 2039196 | 2040149 | + | *orfM* | *PA14_23450* | NAD dependent epimerase/dehydratase | 319 | 282 | 268 | 275 | 329 | 297 | 1 |
| 1903 | - | 2040149 | 2041165 | + | *orfN* | *PA14_23460* | group 4 glycosyl transferase | 79 | 59 | 60 | 63 | 105 | 74 | 1 |
| 1904 | - | 2041610 | 2043607 | + | *wbpM* | *PA14_23470* | nucleotide sugar epimerase/dehydratase WbpM | 340 | 273 | 287 | 345 | 299 | 272 | 1 |
| 1905 | - | 2043798 | 2044127 | + | *-* | *PA14_23480* | hypothetical protein | 7 | 9 | 10 | 8 | 9 | 10 | 1 |
| 1906 | - | 2044275 | 2044203 | - | *-* | *PA14_23490* | Asn tRNA | 7 | 7 | 7 | 6 | 3 | 5 | 1 |
| 1907 | - | 2045540 | 2044344 | - | *tyrB* | *PA14_23500* | aromatic amino acid aminotransferase | 83 | 71 | 83 | 65 | 63 | 58 | 1 |
| 1908 | - | 2045728 | 2047740 | + | *uvrB* | *PA14_23510* | excinuclease ABC subunit B | 64 | 63 | 61 | 61 | 52 | 51 | 1 |
| 1909 | - | 2049243 | 2047744 | - | *-* | *PA14_23520* | MFS transporter | 47 | 38 | 51 | 42 | 32 | 38 | 1 |
| 1910 | - | 2050360 | 2049293 | - | *-* | *PA14_23530* | secretion protein | 4 | 4 | 6 | 4 | 2 | 4 | 1 |
| 1911 | - | 2050430 | 2051350 | + | *act* | *PA14_23540* | transcriptional regulator | 17 | 12 | 16 | 17 | 16 | 17 | 1 |
| 1912 | - | 2051388 | 2052872 | + | *gltX* | *PA14_23560* | glutamyl-tRNA synthetase | 59 | 69 | 53 | 48 | 60 | 46 | 1 |
| 1913 | - | 2053367 | 2053439 | + | *-* | *PA14_23570* | Ala tRNA | 279 | 72 | 107 | 66 | 38 | 34 | 4.59105E-11 |
| 1914 | - | 2053489 | 2053561 | + | *-* | *PA14_23580* | Glu tRNA | 157 | 9 | 20 | 7 | 6 | 7 | 7.7397E-255 |
| 1915 | - | 2053723 | 2054265 | + | *-* | *PA14_23590* | transcriptional regulator | 8 | 7 | 7 | 7 | 6 | 9 | 1 |
| 1916 | - | 2054277 | 2055134 | + | *-* | *PA14_23610* | hydrolase | 14 | 16 | 18 | 19 | 15 | 16 | 1 |
| 1917 | - | 2055247 | 2055894 | + | *kdgA* | *PA14_23620* | aldolase | 26 | 31 | 31 | 30 | 27 | 24 | 1 |
| 1918 | - | 2055894 | 2056331 | + | *-* | *PA14_23630* | hypothetical protein | 25 | 30 | 23 | 24 | 31 | 22 | 1 |
| 1919 | - | 2056424 | 2057383 | + | *-* | *PA14_23640* | hypothetical protein | 40 | 38 | 45 | 40 | 39 | 33 | 1 |
| 1920 | - | 2058144 | 2057398 | - | *-* | *PA14_23650* | short chain dehydrogenase | 67 | 61 | 55 | 62 | 59 | 63 | 1 |
| 1921 | - | 2058964 | 2058161 | - | *-* | *PA14_23670* | hypothetical protein | 31 | 31 | 27 | 24 | 29 | 25 | 1 |
| 1922 | - | 2059122 | 2059571 | + | *ibpA* | *PA14_23680* | heat-shock protein IbpA | 643 | 618 | 685 | 278 | 387 | 319 | 1 |
| 1923 | - | 2060943 | 2059615 | - | *-* | *PA14_23690* | secreted protein | 15 | 14 | 18 | 13 | 13 | 13 | 1 |
| 1924 | - | 2061055 | 2061966 | + | *-* | *PA14_23700* | LysR family transcriptional regulator | 46 | 46 | 41 | 40 | 41 | 40 | 1 |
| 1925 | - | 2062046 | 2062423 | + | *-* | *PA14_23720* | translation initiation inhibitor | 156 | 174 | 156 | 197 | 168 | 228 | 1 |
| 1926 | - | 2063409 | 2062516 | - | *-* | *PA14_23730* | LysR family transcriptional regulator | 76 | 75 | 82 | 100 | 81 | 102 | 1 |
| 1927 | - | 2063572 | 2064996 | + | *leuC* | *PA14_23750* | isopropylmalate isomerase large subunit | 80 | 68 | 73 | 78 | 63 | 77 | 1 |
| 1928 | - | 2065008 | 2065646 | + | *leuD* | *PA14_23760* | isopropylmalate isomerase small subunit | 106 | 114 | 104 | 126 | 97 | 120 | 1 |
| 1929 | - | 2065916 | 2066479 | + | *-* | *PA14_23770* | biotin synthesis protein | 48 | 34 | 44 | 36 | 33 | 41 | 1 |
| 1930 | - | 2066538 | 2067620 | + | *leuB* | *PA14_23790* | 3-isopropylmalate dehydrogenase | 68 | 73 | 72 | 68 | 63 | 65 | 1 |
| 1931 | - | 2067690 | 2068802 | + | *asd* | *PA14_23800* | aspartate-semialdehyde dehydrogenase | 147 | 128 | 145 | 150 | 164 | 152 | 1 |
| 1932 | - | 2068904 | 2069914 | + | *-* | *PA14_23810* | aspartate-semialdehyde dehydrogenase | 57 | 50 | 67 | 54 | 51 | 51 | 1 |

|  | A | B | C | D | E | F | G | H | I | J | K | L | M | N |
| --- | --- | --- | --- | --- | --- | --- | --- | --- | --- | --- | --- | --- | --- | --- |
| 1933 | - | 2070083 | 2072857 | + | *fimV* | *PA14_23830* | pilus assembly protein | 375 | 434 | 422 | 372 | 347 | 371 | 1 |
| 1934 | - | 2072879 | 2073736 | + | *truA* | *PA14_23840* | tRNA pseudouridine synthase A | 108 | 111 | 120 | 103 | 93 | 99 | 1 |
| 1935 | - | 2073825 | 2074460 | + | *trpF* | *PA14_23850* | N-(5'-phosphoribosyl)anthranilate isomerase | 372 | 326 | 465 | 366 | 318 | 299 | 1 |
| 1936 | - | 2074729 | 2075601 | + | *accD* | *PA14_23860* | acetyl-CoA carboxylase subunit beta | 229 | 214 | 238 | 208 | 193 | 192 | 1 |
| 1937 | - | 2075598 | 2076887 | + | *folC* | *PA14_23880* | folylpolyglutamate synthetase | 167 | 179 | 167 | 162 | 147 | 131 | 1 |
| 1938 | - | 2076891 | 2077538 | + | *-* | *PA14_23890* | hypothetical protein | 125 | 128 | 125 | 135 | 129 | 115 | 1 |
| 1939 | - | 2077612 | 2078154 | + | *cvpA* | *PA14_23900* | hypothetical protein | 44 | 35 | 53 | 48 | 41 | 35 | 1 |
| 1940 | - | 2078376 | 2079881 | + | *purF* | *PA14_23920* | amidophosphoribosyltransferase | 151 | 150 | 154 | 128 | 131 | 119 | 1 |
| 1941 | - | 2079898 | 2081109 | + | *metZ* | *PA14_23930* | O-succinylhomoserine sulfhydrylase | 238 | 219 | 231 | 210 | 230 | 204 | 1 |
| 1942 | - | 2081106 | 2081873 | + | *-* | *PA14_23950* | oxidoreductase | 168 | 150 | 190 | 156 | 176 | 146 | 1 |
| 1943 | - | 2083936 | 2081960 | - | *xcpQ* | *PA14_23970* | general secretion pathway protein D | 234 | 243 | 219 | 293 | 257 | 275 | 1 |
| 1944 | - | 2084648 | 2083941 | - | *xcpP* | *PA14_23980* | secretion protein XcpP | 118 | 109 | 132 | 148 | 117 | 127 | 1 |
| 1945 | - | 2084868 | 2086376 | + | *xcpR* | *PA14_23990* | general secretion pathway protein E | 191 | 190 | 188 | 246 | 189 | 235 | 1 |
| 1946 | - | 2086376 | 2087593 | + | *xcpS* | *PA14_24010* | general secretion pathway protein F | 142 | 135 | 143 | 185 | 156 | 176 | 1 |
| 1947 | - | 2087598 | 2088044 | + | *xcpT* | *PA14_24020* | general secretion pathway protein G | 248 | 335 | 255 | 350 | 375 | 363 | 1 |
| 1948 | - | 2088051 | 2088569 | + | *xcpU* | *PA14_24040* | general secretion pathway outer membrane protein H precursor | 177 | 198 | 187 | 210 | 205 | 210 | 1 |
| 1949 | - | 2088566 | 2088955 | + | *xcpV* | *PA14_24050* | general secretion pathway protein I | 134 | 149 | 144 | 170 | 149 | 164 | 1 |
| 1950 | - | 2088952 | 2089665 | + | *xcpW* | *PA14_24060* | general secretion pathway protein J | 171 | 181 | 180 | 240 | 208 | 214 | 1 |
| 1951 | - | 2089662 | 2090663 | + | *xcpX* | *PA14_24070* | general secretion pathway protein K | 95 | 92 | 112 | 129 | 116 | 116 | 1 |
| 1952 | - | 2090660 | 2091808 | + | *xcpY* | *PA14_24080* | general secretion pathway protein L | 301 | 294 | 310 | 378 | 331 | 332 | 1 |
| 1953 | - | 2091810 | 2092334 | + | *xcpZ* | *PA14_24100* | general secretion pathway protein M | 182 | 177 | 189 | 226 | 204 | 184 | 1 |
| 1954 | - | 2092473 | 2092545 | + | *-* | *PA14_24110* | Val tRNA | 263 | 172 | 269 | 235 | 162 | 109 | 1 |
| 1955 | - | 2092565 | 2092638 | + | *-* | *PA14_24120* | Asp tRNA | 785 | 749 | 868 | 774 | 576 | 489 | 1 |
| 1956 | - | 2092756 | 2092829 | + | *-* | *PA14_24130* | Asp tRNA | 344 | 334 | 372 | 294 | 223 | 189 | 1 |
| 1957 | - | 2094157 | 2093120 | - | *-* | *PA14_24140* | AraC family transcriptional regulator | 37 | 41 | 39 | 40 | 32 | 31 | 1 |
| 1958 | - | 2095272 | 2094154 | - | *-* | *PA14_24150* | hypothetical protein | 37 | 33 | 41 | 35 | 29 | 32 | 1 |
| 1959 | - | 2095487 | 2097526 | + | *fadH1* | *PA14_24170* | 2,4-dienoyl-CoA reductase | 26 | 28 | 26 | 25 | 25 | 22 | 1 |
| 1960 | - | 2097863 | 2099302 | + | *-* | *PA14_24180* | hypothetical protein | 98 | 107 | 120 | 131 | 83 | 94 | 1 |
| 1961 | - | 2099307 | 2100239 | + | *-* | *PA14_24190* | 1-aminocyclopropane-1-carboxylate deaminase | 50 | 58 | 57 | 55 | 49 | 55 | 1 |
| 1962 | - | 2101205 | 2100240 | - | *-* | *PA14_24210* | hypothetical protein | 69 | 75 | 77 | 80 | 85 | 111 | 1 |
| 1963 | - | 2101330 | 2102217 | + | *ppnK* | *PA14_24220* | inorganic polyphosphate/ATP-NAD kinase | 92 | 75 | 94 | 72 | 77 | 74 | 1 |
| 1964 | - | 2102217 | 2103197 | + | *-* | *PA14_24230* | hypothetical protein | 92 | 90 | 86 | 91 | 75 | 84 | 1 |
| 1965 | - | 2103194 | 2104054 | + | *-* | *PA14_24240* | hypothetical protein | 151 | 136 | 145 | 154 | 144 | 136 | 1 |
| 1966 | - | 2104138 | 2104401 | + | *-* | *PA14_24245* | hypothetical protein | 175 | 151 | 184 | 125 | 148 | 143 | 1 |
| 1967 | - | 2104434 | 2105234 | + | *-* | *PA14_24260* | hypothetical protein | 119 | 113 | 127 | 111 | 107 | 110 | 1 |
| 1968 | - | 2105253 | 2107910 | + | *pepN* | *PA14_24270* | aminopeptidase | 163 | 175 | 153 | 151 | 160 | 157 | 1 |
| 1969 | - | 2108357 | 2110321 | + | *gbt* | *PA14_24290* | glycine betaine transmethylase | 45 | 59 | 47 | 43 | 45 | 40 | 1 |
| 1970 | - | 2110380 | 2111747 | + | *-* | *PA14_24300* | hypothetical protein | 37 | 52 | 38 | 31 | 42 | 28 | 1 |
| 1971 | - | 2111927 | 2113024 | + | *-* | *PA14_24310* | BNR/Asp-box repeat-containing protein | 48 | 78 | 66 | 80 | 70 | 61 | 0.931437202 |
| 1972 | - | 2113036 | 2115417 | + | *-* | *PA14_24330* | hypothetical protein | 44 | 76 | 57 | 75 | 68 | 60 | 1 |
| 1973 | - | 2116784 | 2115489 | - | *-* | *PA14_24340* | two-component sensor | 20 | 24 | 24 | 25 | 23 | 21 | 1 |
| 1974 | - | 2117452 | 2116781 | - | *-* | *PA14_24350* | two-component response regulator | 15 | 10 | 17 | 14 | 14 | 13 | 0.969261494 |
| 1975 | - | 2117776 | 2120700 | + | *-* | *PA14_24360* | hypothetical protein | 11 | 11 | 12 | 12 | 10 | 12 | 1 |
| 1976 | - | 2121786 | 2120707 | - | *-* | *PA14_24370* | hypothetical protein | 69 | 65 | 68 | 72 | 65 | 74 | 1 |
| 1977 | - | 2123471 | 2121840 | - | *-* | *PA14_24380* | hypothetical protein | 66 | 60 | 64 | 74 | 62 | 73 | 1 |
| 1978 | - | 2125228 | 2123468 | - | *-* | *PA14_24390* | hypothetical protein | 44 | 36 | 44 | 45 | 38 | 43 | 1 |
| 1979 | - | 2126247 | 2125225 | - | *-* | *PA14_24400* | von Willebrand factor A | 34 | 29 | 32 | 36 | 29 | 33 | 1 |
| 1980 | - | 2126734 | 2126240 | - | *-* | *PA14_24410* | hypothetical protein | 29 | 33 | 32 | 33 | 25 | 31 | 1 |
| 1981 | - | 2127669 | 2126731 | - | *-* | *PA14_24420* | hypothetical protein | 61 | 54 | 63 | 60 | 47 | 54 | 1 |
| 1982 | - | 2128660 | 2127680 | - | *-* | *PA14_24430* | hypothetical protein | 107 | 90 | 119 | 99 | 92 | 103 | 1 |
| 1983 | - | 2129153 | 2129770 | + | *-* | *PA14_24440* | lipoprotein | 68 | 46 | 71 | 67 | 50 | 56 | 1 |
| 1984 | - | 2130044 | 2134906 | + | *gdhB* | *PA14_24445* | NAD-dependent glutamate dehydrogenase | 365 | 542 | 349 | 386 | 452 | 377 | 1 |
| 1985 | - | 2135468 | 2138314 | + | *pelA* | *PA14_24480* | hypothetical protein | 14 | 6 | 8 | 7 | 5 | 6 | 0.23199809 |
| 1986 | - | 2138292 | 2141873 | + | *pelB* | *PA14_24490* | hypothetical protein | 14 | 5 | 5 | 5 | 5 | 5 | 0.044138282 |
| 1987 | - | 2141913 | 2142431 | + | *pelC* | *PA14_24500* | lipoprotein | 18 | 5 | 4 | 4 | 5 | 3 | 1.46581E-05 |
| 1988 | - | 2142437 | 2143804 | + | *pelD* | *PA14_24510* | hypothetical protein | 12 | 4 | 4 | 4 | 4 | 3 | 0.000626932 |
| 1989 | - | 2143782 | 2144771 | + | *pelE* | *PA14_24530* | hypothetical protein | 24 | 14 | 18 | 20 | 14 | 14 | 0.915523483 |
| 1990 | - | 2144768 | 2146291 | + | *pelF* | *PA14_24550* | hypothetical protein | 16 | 8 | 8 | 7 | 7 | 8 | 0.480861202 |
| 1991 | - | 2146293 | 2147663 | + | *pelG* | *PA14_24560* | hypothetical protein | 11 | 7 | 6 | 7 | 7 | 8 | 1 |
| 1992 | - | 2147934 | 2147704 | - | *-* | *PA14_24570* | hypothetical protein | 11 | 9 | 10 | 7 | 10 | 8 | 1 |
| 1993 | - | 2148060 | 2148536 | + | *-* | *PA14_24580* | hypothetical protein | 30 | 31 | 30 | 22 | 23 | 24 | 1 |
| 1994 | - | 2148574 | 2149050 | + | *-* | *PA14_24590* | hypothetical protein | 62 | 66 | 64 | 57 | 60 | 58 | 1 |
| 1995 | - | 2151071 | 2149167 | - | *-* | *PA14_24600* | carboxypeptidase | 30 | 32 | 31 | 23 | 30 | 23 | 1 |
| 1996 | - | 2152240 | 2151239 | - | *-* | *PA14_24610* | hydrolytic enzyme | 26 | 35 | 28 | 26 | 35 | 26 | 1 |
| 1997 | - | 2153286 | 2153567 | + | *-* | *PA14_24630* | hypothetical protein | 86 | 87 | 94 | 102 | 82 | 84 | 1 |
| 1998 | - | 2153292 | 2152312 | - | *-* | *PA14_24620* | hypothetical protein | 59 | 58 | 70 | 53 | 61 | 58 | 1 |
| 1999 | - | 2153624 | 2154652 | + | *pyrD* | *PA14_24640* | dihydroorotate dehydrogenase 2 | 71 | 59 | 94 | 71 | 55 | 59 | 1 |
| 2000 | - | 2154952 | 2154740 | - | *rmf* | *PA14_24650* | ribosome modulation factor | 1022 | 1426 | 992 | 1646 | 1141 | 1471 | 1 |
| 2001 | - | 2155414 | 2156517 | + | *-* | *PA14_24665* | hypothetical protein | 48 | 41 | 51 | 45 | 40 | 40 | 1 |
| 2002 | - | 2156561 | 2157592 | + | *-* | *PA14_24675* | hypothetical protein | 40 | 38 | 46 | 44 | 38 | 37 | 1 |
| 2003 | - | 2159092 | 2157662 | - | *-* | *PA14_24690* | D-alanyl-D-alanine carboxypeptidase | 79 | 68 | 80 | 66 | 72 | 70 | 1 |
| 2004 | - | 2159403 | 2159747 | + | *-* | *PA14_24700* | hypothetical protein | 77 | 100 | 62 | 49 | 92 | 55 | 1 |
| 2005 | - | 2160174 | 2160797 | + | *-* | *PA14_24710* | two-component response regulator | 9 | 11 | 11 | 8 | 11 | 11 | 1 |
| 2006 | - | 2160811 | 2163036 | + | *-* | *PA14_24720* | two-component sensor | 11 | 13 | 12 | 12 | 12 | 13 | 1 |
| 2007 | - | 2164378 | 2163047 | - | *-* | *PA14_24730* | deoxyguanosinetriphosphate triphosphohydrolase-like protein | 91 | 96 | 88 | 77 | 89 | 85 | 1 |
| 2008 | - | 2164818 | 2164495 | - | *-* | *PA14_24740* | hypothetical protein | 308 | 385 | 364 | 281 | 312 | 260 | 1 |
| 2009 | - | 2165212 | 2164832 | - | *-* | *PA14_24760* | hypothetical protein | 445 | 565 | 448 | 385 | 539 | 452 | 1 |
| 2010 | - | 2165545 | 2165216 | - | *-* | *PA14_24770* | hypothetical protein | 601 | 858 | 605 | 472 | 649 | 608 | 1 |
| 2011 | - | 2165841 | 2167223 | + | *-* | *PA14_24780* | transporter | 6 | 4 | 6 | 5 | 5 | 6 | 1 |
| 2012 | - | 2168673 | 2167408 | - | *-* | *PA14_24790* | outer membrane porin | 54 | 91 | 62 | 61 | 56 | 56 | 1 |
| 2013 | - | 2169708 | 2168842 | - | *-* | *PA14_24810* | hypothetical protein | 26 | 18 | 17 | 21 | 16 | 29 | 1 |
| 2014 | - | 2170640 | 2169708 | - | *-* | *PA14_24820* | hypothetical protein | 13 | 10 | 11 | 12 | 9 | 14 | 1 |
| 2015 | - | 2171253 | 2170657 | - | *-* | *PA14_24830* | glutathione S-transferase | 11 | 11 | 15 | 12 | 8 | 10 | 1 |
| 2016 | - | 2171419 | 2171976 | + | *-* | *PA14_24840* | transcriptional regulator | 44 | 35 | 49 | 35 | 33 | 36 | 1 |

|  | A | B | C | D | E | F | G | H | I | J | K | L | M | N |
| --- | --- | --- | --- | --- | --- | --- | --- | --- | --- | --- | --- | --- | --- | --- |
| 2017 | - | 2171983 | 2172261 | + | *-* | *PA14_24850* | hypothetical protein | 31 | 30 | 30 | 23 | 28 | 27 | 1 |
| 2018 | - | 2172628 | 2174031 | + | *snr1* | *PA14_24860* | cytochrome c Snr1 | 89 | 12 | 13 | 17 | 12 | 160 | 9.5063E-30 |
| 2019 | - | 2174165 | 2174092 | - | *-* | *PA14_24870* | Pro tRNA | 2 | 1 | 3 | 3 | 1 | 1 | 0.005569024 |
| 2020 | - | 2174540 | 2174319 | - | *-* | *PA14_24880* | lipoprotein | 574 | 671 | 479 | 418 | 537 | 533 | 1 |
| 2021 | - | 2175268 | 2174672 | - | *mobA* | *PA14_24890* | molybdopterin-guanine dinucleotide biosynthesis protein MobA | 31 | 25 | 31 | 30 | 25 | 32 | 1 |
| 2022 | - | 2175335 | 2175874 | + | *moaB2* | *PA14_24900* | molybdopterin biosynthetic protein B2 | 88 | 98 | 80 | 71 | 79 | 63 | 1 |
| 2023 | - | 2175871 | 2177088 | + | *moeA2* | *PA14_24910* | molybdenum cofactor biosynthesis protein A2 | 121 | 123 | 99 | 99 | 118 | 94 | 1 |
| 2024 | - | 2178148 | 2177117 | - | *-* | *PA14_24920* | transcriptional regulator | 35 | 34 | 35 | 36 | 34 | 29 | 1 |
| 2025 | - | 2178325 | 2179920 | + | *-* | *PA14_24940* | hypothetical protein | 35 | 35 | 36 | 37 | 39 | 36 | 1 |
| 2026 | - | 2180121 | 2181506 | + | *-* | *PA14_24950* | FAD-dependent glycerol-3-phosphate dehydrogenase | 41 | 37 | 38 | 38 | 38 | 41 | 1 |
| 2027 | - | 2181527 | 2183086 | + | *-* | *PA14_24960* | carbohydrate kinase | 48 | 45 | 50 | 51 | 49 | 54 | 1 |
| 2028 | - | 2183161 | 2184069 | + | *-* | *PA14_24970* | lipid kinase | 70 | 60 | 81 | 67 | 70 | 69 | 1 |
| 2029 | - | 2184172 | 2184978 | + | *-* | *PA14_24980* | hypothetical protein | 287 | 217 | 268 | 324 | 256 | 269 | 1 |
| 2030 | - | 2185408 | 2185022 | - | *-* | *PA14_24990* | hypothetical protein | 191 | 177 | 160 | 226 | 215 | 182 | 1 |
| 2031 | - | 2187393 | 2185465 | - | *-* | *PA14_25000* | lytic transglycosylase | 73 | 70 | 77 | 82 | 74 | 83 | 1 |
| 2032 | - | 2187619 | 2189541 | + | *-* | *PA14_25020* | ABC transporter ATP-binding protein | 59 | 62 | 74 | 60 | 57 | 50 | 1 |
| 2033 | - | 2189692 | 2190378 | + | *-* | *PA14_25030* | hypothetical protein | 52 | 53 | 54 | 42 | 67 | 45 | 1 |
| 2034 | - | 2190845 | 2190408 | - | *-* | *PA14_25040* | hypothetical protein | 183 | 184 | 178 | 163 | 206 | 184 | 1 |
| 2035 | - | 2191053 | 2191487 | + | *-* | *PA14_25050* | hypothetical protein | 14 | 16 | 19 | 14 | 15 | 16 | 1 |
| 2036 | - | 2192322 | 2191504 | - | *-* | *PA14_25060* | hypothetical protein | 12 | 18 | 17 | 13 | 12 | 14 | 1 |
| 2037 | - | 2192750 | 2194897 | + | *fadB* | *PA14_25080* | multifunctional fatty acid oxidation complex subunit alpha | 192 | 247 | 219 | 176 | 209 | 181 | 1 |
| 2038 | - | 2194928 | 2196103 | + | *fadA* | *PA14_25090* | 3-ketoacyl-CoA thiolase | 193 | 278 | 197 | 190 | 248 | 207 | 1 |
| 2039 | - | 2196186 | 2196560 | + | *-* | *PA14_25100* | hypothetical protein | 59 | 45 | 74 | 66 | 55 | 63 | 1 |
| 2040 | - | 2196651 | 2199257 | + | *topA* | *PA14_25110* | DNA topoisomerase I | 152 | 177 | 164 | 174 | 171 | 152 | 1 |
| 2041 | - | 2199364 | 2199882 | + | *-* | *PA14_25130* | hypothetical protein | 140 | 153 | 163 | 151 | 142 | 142 | 1 |
| 2042 | - | 2200088 | 2200321 | + | *-* | *PA14_25140* | hypothetical protein | 115 | 77 | 155 | 96 | 97 | 113 | 1 |
| 2043 | - | 2200828 | 2200343 | - | *-* | *PA14_25150* | hypothetical protein | 73 | 73 | 87 | 67 | 67 | 75 | 1 |
| 2044 | - | 2201454 | 2200840 | - | *lexA* | *PA14_25160* | LexA repressor | 73 | 72 | 78 | 78 | 76 | 80 | 1 |
| 2045 | - | 2201686 | 2202387 | + | *psrA* | *PA14_25180* | transcriptional regulator PsrA | 972 | 774 | 1094 | 918 | 890 | 922 | 1 |
| 2046 | - | 2202589 | 2203587 | + | *nagZ* | *PA14_25195* | beta-hexosaminidase | 57 | 47 | 56 | 48 | 47 | 46 | 1 |
| 2047 | - | 2203599 | 2204336 | + | *-* | *PA14_25210* | 5'-methylthioadenosine phosphorylase | 98 | 103 | 88 | 108 | 108 | 95 | 1 |
| 2048 | - | 2205012 | 2204383 | - | *-* | *PA14_25220* | hypothetical protein | 112 | 108 | 110 | 113 | 94 | 111 | 1 |
| 2049 | - | 2208469 | 2205023 | - | *mfd* | *PA14_25230* | transcription-repair coupling factor | 112 | 114 | 108 | 114 | 99 | 99 | 1 |
| 2050 | - | 2208690 | 2210075 | + | *-* | *PA14_25250* | glyceraldehyde-3-phosphate dehydrogenase | 252 | 251 | 267 | 187 | 188 | 158 | 1 |
| 2051 | - | 2210177 | 2211586 | + | *aroP1* | *PA14_25270* | aromatic amino acid transport protein AroP1 | 63 | 56 | 74 | 55 | 54 | 47 | 1 |
| 2052 | - | 2211845 | 2213182 | + | *nqrA* | *PA14_25280* | Na(+)-translocating NADH-quinone reductase subunit A | 119 | 136 | 147 | 154 | 141 | 122 | 1 |
| 2053 | - | 2213186 | 2214397 | + | *nqrB* | *PA14_25305* | Na(+)-translocating NADH-quinone reductase subunit B | 123 | 168 | 139 | 157 | 140 | 114 | 1 |
| 2054 | - | 2214390 | 2215175 | + | *nqrC* | *PA14_25320* | Na(+)-translocating NADH-quinone reductase subunit C | 153 | 184 | 151 | 147 | 127 | 121 | 1 |
| 2055 | - | 2215172 | 2215846 | + | *nqrD* | *PA14_25330* | Na(+)-translocating NADH-quinone reductase subunit D | 169 | 219 | 178 | 166 | 159 | 152 | 1 |
| 2056 | - | 2215846 | 2216454 | + | *nqrE* | *PA14_25340* | Na(+)-translocating NADH-quinone reductase subunit E | 169 | 188 | 166 | 155 | 154 | 150 | 1 |
| 2057 | - | 2216466 | 2217689 | + | *nqrF* | *PA14_25350* | Na(+)-translocating NADH-quinone reductase subunit F | 212 | 222 | 221 | 212 | 219 | 199 | 1 |
| 2058 | - | 2217682 | 2218710 | + | *-* | *PA14_25360* | thiamine biosynthesis lipoprotein | 225 | 223 | 232 | 239 | 235 | 223 | 1 |
| 2059 | - | 2218707 | 2218934 | + | *-* | *PA14_25370* | hypothetical protein | 210 | 274 | 228 | 278 | 243 | 247 | 1 |
| 2060 | - | 2219113 | 2220507 | + | *sth* | *PA14_25390* | soluble pyridine nucleotide transhydrogenase | 221 | 198 | 201 | 186 | 198 | 177 | 1 |
| 2061 | - | 2221272 | 2220550 | - | *-* | *PA14_25400* | phosphodiesterase | 118 | 121 | 104 | 103 | 102 | 99 | 1 |
| 2062 | - | 2221577 | 2221269 | - | *-* | *PA14_25410* | hypothetical protein | 37 | 40 | 40 | 30 | 28 | 28 | 1 |
| 2063 | - | 2222289 | 2223539 | + | *-* | *PA14_25430* | hypothetical protein | 68 | 43 | 73 | 53 | 52 | 52 | 1 |
| 2064 | - | 2222351 | 2221587 | - | *-* | *PA14_25420* | hypothetical protein | 44 | 34 | 49 | 39 | 34 | 40 | 1 |
| 2065 | - | 2223532 | 2224215 | + | *-* | *PA14_25440* | lipoprotein releasing system, ATP-binding protein | 127 | 122 | 127 | 111 | 128 | 104 | 1 |
| 2066 | - | 2224228 | 2225529 | + | *-* | *PA14_25450* | hypothetical protein | 46 | 37 | 44 | 32 | 42 | 36 | 1 |
| 2067 | - | 2226150 | 2225617 | - | *-* | *PA14_25470* | hypothetical protein | 31 | 28 | 30 | 18 | 21 | 21 | 1 |
| 2068 | - | 2226281 | 2228506 | + | *-* | *PA14_25480* | competence protein | 36 | 29 | 35 | 39 | 33 | 35 | 1 |
| 2069 | - | 2228579 | 2229214 | + | *-* | *PA14_25490* | tolQ-type transport protein | 67 | 58 | 70 | 61 | 53 | 55 | 1 |
| 2070 | - | 2229211 | 2229651 | + | *-* | *PA14_25500* | hypothetical protein | 169 | 191 | 185 | 182 | 169 | 140 | 1 |
| 2071 | - | 2229651 | 2230649 | + | *lpxK* | *PA14_25510* | tetraacyldisaccharide 4'-kinase | 89 | 95 | 101 | 92 | 90 | 69 | 1 |
| 2072 | - | 2230687 | 2230872 | + | *-* | *PA14_25520* | hypothetical protein | 93 | 109 | 103 | 97 | 85 | 100 | 1 |
| 2073 | - | 2230872 | 2231636 | + | *kdsB* | *PA14_25530* | 3-deoxy-manno-octulosonate cytidylyltransferase | 105 | 114 | 109 | 100 | 95 | 92 | 1 |
| 2074 | - | 2231636 | 2232100 | + | *ptpA* | *PA14_25540* | phosphotyrosine protein phosphatase | 130 | 127 | 132 | 124 | 123 | 119 | 1 |
| 2075 | - | 2232097 | 2233116 | + | *murB* | *PA14_25550* | UDP-N-acetylenolpyruvoylglucosamine reductase | 131 | 114 | 134 | 125 | 115 | 105 | 1 |
| 2076 | - | 2236447 | 2233274 | - | *rne* | *PA14_25560* | ribonuclease E | 155 | 172 | 147 | 147 | 151 | 132 | 1 |
| 2077 | - | 2237024 | 2237980 | + | *rluC* | *PA14_25580* | ribosomal large subunit pseudouridine synthase C | 305 | 228 | 356 | 292 | 323 | 320 | 1 |
| 2078 | - | 2237973 | 2238665 | + | *-* | *PA14_25590* | hydrolase | 262 | 227 | 308 | 272 | 273 | 273 | 1 |
| 2079 | - | 2238658 | 2239638 | + | *-* | *PA14_25600* | peptidase | 295 | 295 | 328 | 292 | 302 | 285 | 1 |
| 2080 | - | 2240299 | 2239721 | - | *-* | *PA14_25610* | Maf-like protein | 21 | 26 | 23 | 22 | 19 | 21 | 1 |
| 2081 | - | 2240409 | 2240945 | + | *-* | *PA14_25620* | hypothetical protein | 240 | 212 | 341 | 220 | 147 | 135 | 1 |
| 2082 | - | 2240959 | 2241141 | + | *rpmF* | *PA14_25630* | 50S ribosomal protein L32 | 197 | 291 | 228 | 162 | 162 | 106 | 1 |
| 2083 | - | 2241175 | 2242155 | + | *plsX* | *PA14_25640* | glycerol-3-phosphate acyltransferase PlsX | 53 | 63 | 64 | 42 | 46 | 35 | 1 |
| 2084 | - | 2242261 | 2243199 | + | *fabD* | *PA14_25650* | malonyl-CoA-ACP transacylase | 89 | 107 | 108 | 87 | 85 | 66 | 1 |
| 2085 | - | 2243222 | 2243965 | + | *fabG* | *PA14_25660* | 3-ketoacyl-ACP reductase | 462 | 612 | 537 | 361 | 404 | 340 | 1 |
| 2086 | - | 2244062 | 2244090 | ? | *-* | predicted RNA | - | 619 | 775 | 547 | 421 | 732 | 501 | 1 |
| 2087 | - | 2244161 | 2244397 | + | *acpP* | *PA14_25670* | acyl carrier protein | 600 | 896 | 540 | 356 | 565 | 398 | 1 |
| 2088 | - | 2244526 | 2245770 | + | *fabF1* | *PA14_25690* | 3-oxoacyl-ACP synthase | 183 | 210 | 168 | 145 | 190 | 142 | 1 |
| 2089 | - | 2245770 | 2246585 | + | *pabC* | *PA14_25710* | 4-amino-4-deoxychorismate lyase | 170 | 164 | 179 | 136 | 160 | 131 | 1 |
| 2090 | - | 2246591 | 2247640 | + | *-* | *PA14_25730* | hypothetical protein | 77 | 57 | 83 | 73 | 63 | 59 | 1 |
| 2091 | - | 2247669 | 2248301 | + | *tmk* | *PA14_25740* | thymidylate kinase | 123 | 116 | 135 | 123 | 128 | 111 | 1 |
| 2092 | - | 2248294 | 2249280 | + | *holB* | *PA14_25760* | DNA polymerase III subunit delta' | 113 | 120 | 125 | 102 | 122 | 106 | 1 |
| 2093 | - | 2249314 | 2249670 | + | *pilZ* | *PA14_25770* | type 4 fimbrial biogenesis protein PilZ | 100 | 87 | 129 | 103 | 95 | 100 | 1 |
| 2094 | - | 2249685 | 2250461 | + | *-* | *PA14_25780* | TatD family deoxyribonuclease | 67 | 66 | 81 | 65 | 53 | 55 | 1 |
| 2095 | - | 2251820 | 2250687 | - | *-* | *PA14_25790* | hypothetical protein | 47 | 44 | 49 | 48 | 36 | 44 | 1 |
| 2096 | - | 2251906 | 2252544 | + | *-* | *PA14_25800* | TetR family transcriptional regulator | 99 | 117 | 118 | 111 | 91 | 91 | 1 |
| 2097 | - | 2252711 | 2253607 | + | *-* | *PA14_25810* | hypothetical protein | 54 | 56 | 61 | 59 | 49 | 43 | 1 |
| 2098 | - | 2253672 | 2254304 | + | *-* | *PA14_25820* | lipoprotein | 40 | 34 | 44 | 45 | 37 | 32 | 1 |
| 2099 | - | 2254297 | 2254866 | + | *-* | *PA14_25830* | hypothetical protein | 27 | 35 | 33 | 34 | 27 | 27 | 1 |
| 2100 | - | 2256570 | 2254915 | - | *-* | *PA14_25840* | electron transfer flavoprotein-ubiquinone oxidoreductase | 128 | 116 | 129 | 88 | 104 | 100 | 1 |

|  | A | B | C | D | E | F | G | H | I | J | K | L | M | N |
| --- | --- | --- | --- | --- | --- | --- | --- | --- | --- | --- | --- | --- | --- | --- |
| 2101 | - | 2256891 | 2257640 | + | *etfB* | *PA14_25860* | electron transfer flavoprotein subunit beta | 382 | 547 | 322 | 251 | 408 | 252 | 1 |
| 2102 | - | 2257640 | 2258569 | + | *etfA* | *PA14_25880* | electron transfer flavoprotein subunit alpha | 775 | 865 | 691 | 586 | 713 | 520 | 1 |
| 2103 | - | 2258754 | 2259950 | + | *-* | *PA14_25900* | trans-2-enoyl-CoA reductase | 77 | 102 | 84 | 76 | 87 | 60 | 1 |
| 2104 | - | 2260970 | 2260023 | - | *-* | *PA14_25910* | lipase | 19 | 21 | 24 | 26 | 19 | 20 | 1 |
| 2105 | - | 2261851 | 2261099 | - | *cobM* | *PA14_25920* | precorrin-3 methylase | 22 | 24 | 24 | 25 | 26 | 24 | 1 |
| 2106 | - | 2262276 | 2261848 | - | *-* | *PA14_25930* | hypothetical protein | 16 | 14 | 15 | 14 | 14 | 13 | 1 |
| 2107 | - | 2263443 | 2262349 | - | *-* | *PA14_25940* | hypothetical protein | 57 | 65 | 58 | 52 | 59 | 61 | 1 |
| 2108 | - | 2263799 | 2264926 | + | *-* | *PA14_25960* | cobalamin biosynthesis protein cobW | 40 | 48 | 35 | 38 | 44 | 36 | 1 |
| 2109 | - | 2265002 | 2268748 | + | *cobN* | *PA14_25970* | cobaltochelatase subunit CobN | 43 | 46 | 37 | 43 | 47 | 43 | 1 |
| 2110 | - | 2269871 | 2268777 | - | *aroF* | *PA14_25980* | phospho-2-dehydro-3-deoxyheptonate aldolase | 21 | 21 | 21 | 18 | 22 | 15 | 1 |
| 2111 | - | 2270442 | 2271455 | + | *-* | *PA14_25990* | magnesium chelatase | 60 | 47 | 53 | 45 | 45 | 43 | 1 |
| 2112 | - | 2271476 | 2272120 | + | *-* | *PA14_26000* | magnesium chelatase | 60 | 46 | 55 | 53 | 49 | 49 | 1 |
| 2113 | - | 2272242 | 2273381 | + | *-* | *PA14_26010* | acyl-CoA thiolase | 32 | 31 | 35 | 40 | 30 | 34 | 1 |
| 2114 | - | 2275078 | 2273468 | - | *-* | *PA14_26020* | aminopeptidase | 97 | 165 | 119 | 197 | 157 | 224 | 1 |
| 2115 | - | 2275579 | 2277036 | + | *-* | *PA14_26050* | transporter | 17 | 21 | 26 | 31 | 19 | 25 | 1 |
| 2116 | - | 2277400 | 2277092 | - | *-* | *PA14_26060* | hypothetical protein | 34 | 53 | 56 | 47 | 34 | 57 | 1 |
| 2117 | - | 2278077 | 2277496 | - | *-* | *PA14_26070* | hypothetical protein | 4 | 4 | 5 | 3 | 3 | 3 | 1 |
| 2118 | - | 2278467 | 2278667 | + | *-* | *PA14_26080* | hypothetical protein | 6 | 5 | 3 | 5 | 3 | 4 | 1 |
| 2119 | - | 2279646 | 2278687 | - | *-* | *PA14_26090* | hydrolase | 5 | 6 | 7 | 5 | 6 | 5 | 1 |
| 2120 | - | 2280846 | 2279668 | - | *-* | *PA14_26110* | MFS transporter | 3 | 3 | 5 | 3 | 3 | 4 | 1 |
| 2121 | - | 2282021 | 2280912 | - | *morB* | *PA14_26130* | morphinone reductase | 10 | 9 | 9 | 9 | 6 | 7 | 1 |
| 2122 | - | 2282147 | 2282737 | + | *-* | *PA14_26140* | transcriptional regulator | 149 | 108 | 139 | 149 | 119 | 136 | 1 |
| 2123 | - | 2283674 | 2282748 | - | *-* | *PA14_26150* | transcriptional regulator | 52 | 44 | 56 | 39 | 42 | 45 | 1 |
| 2124 | - | 2283740 | 2284354 | + | *-* | *PA14_26160* | hypothetical protein | 4 | 3 | 3 | 4 | 4 | 3 | 1 |
| 2125 | - | 2284423 | 2285985 | + | *-* | *PA14_26165* | hypothetical protein | 23 | 25 | 26 | 28 | 23 | 23 | 1 |
| 2126 | - | 2287367 | 2286036 | - | *-* | *PA14_26190* | hypothetical protein | 35 | 30 | 32 | 41 | 33 | 34 | 1 |
| 2127 | - | 2287497 | 2287679 | + | *-* | *PA14_26200* | hypothetical protein | 17 | 20 | 25 | 16 | 14 | 13 | 1 |
| 2128 | - | 2288456 | 2287689 | - | *hisP* | *PA14_26210* | histidine transport | 7 | 8 | 9 | 7 | 8 | 6 | 1 |
| 2129 | - | 2289190 | 2288477 | - | *hisM* | *PA14_26220* | histidine ABC transporter, inner membrane permease | 11 | 11 | 11 | 12 | 12 | 10 | 1 |
| 2130 | - | 2289876 | 2289187 | - | *hisQ* | *PA14_26230* | histidine transport system permease | 6 | 5 | 7 | 5 | 6 | 6 | 1 |
| 2131 | - | 2290717 | 2289932 | - | *hisJ* | *PA14_26240* | periplasmic histidine-binding protein HisJ | 5 | 4 | 3 | 3 | 4 | 2 | 1 |
| 2132 | - | 2291946 | 2290777 | - | *-* | *PA14_26260* | hydrolase | 4 | 3 | 3 | 4 | 4 | 3 | 1 |
| 2133 | - | 2292047 | 2293036 | + | *-* | *PA14_26270* | transcriptional regulator | 10 | 10 | 12 | 12 | 9 | 12 | 1 |
| 2134 | - | 2293121 | 2294758 | + | *-* | *PA14_26280* | chemotaxis transducer | 128 | 130 | 180 | 171 | 162 | 181 | 1 |
| 2135 | - | 2295027 | 2294770 | - | *-* | *PA14_26300* | hypothetical protein | 89 | 110 | 132 | 157 | 139 | 166 | 1 |
| 2136 | - | 2295854 | 2295081 | - | *-* | *PA14_26310* | short chain dehydrogenase | 32 | 38 | 42 | 47 | 45 | 53 | 1 |
| 2137 | - | 2296065 | 2296901 | + | *-* | *PA14_26330* | AraC family transcriptional regulator | 22 | 28 | 34 | 42 | 28 | 28 | 1 |
| 2138 | - | 2296952 | 2297545 | + | *-* | *PA14_26340* | hypothetical protein | 5 | 10 | 10 | 9 | 8 | 5 | 0.272776094 |
| 2139 | - | 2297665 | 2298531 | + | *-* | *PA14_26350* | hypothetical protein | 136 | 128 | 118 | 127 | 119 | 147 | 1 |
| 2140 | - | 2299563 | 2298532 | - | *-* | *PA14_26360* | ABC transporter permease | 56 | 47 | 51 | 62 | 53 | 70 | 1 |
| 2141 | - | 2300531 | 2299560 | - | *-* | *PA14_26390* | hypothetical protein | 15 | 14 | 15 | 18 | 15 | 21 | 1 |
| 2142 | - | 2301301 | 2300528 | - | *-* | *PA14_26400* | ABC transporter ATP-binding protein | 10 | 11 | 12 | 14 | 10 | 14 | 1 |
| 2143 | - | 2303510 | 2301354 | - | *-* | *PA14_26420* | TonB-dependent receptor | 15 | 15 | 19 | 20 | 15 | 15 | 1 |
| 2144 | - | 2304147 | 2304716 | + | *-* | *PA14_26450* | hypothetical protein | 4 | 6 | 6 | 8 | 5 | 5 | 1 |
| 2145 | - | 2305498 | 2304770 | - | *cobK* | *PA14_26460* | cobalt-precorrin-6x reductase | 49 | 39 | 46 | 41 | 45 | 38 | 1 |
| 2146 | - | 2306595 | 2305495 | - | *cbiD* | *PA14_26470* | cobalt-precorrin-6A synthase | 33 | 35 | 33 | 32 | 32 | 29 | 1 |
| 2147 | - | 2307835 | 2306588 | - | *cobL* | *PA14_26480* | precorrin-6y-dependent methyltransferase CobL | 33 | 33 | 31 | 30 | 30 | 26 | 1 |
| 2148 | - | 2308172 | 2309632 | + | *-* | *PA14_26485* | oxidoreductase | 36 | 30 | 34 | 26 | 28 | 27 | 1 |
| 2149 | - | 2309629 | 2310255 | + | *cobH* | *PA14_26500* | precorrin-8X methylmutase | 29 | 35 | 33 | 26 | 32 | 23 | 1 |
| 2150 | - | 2310252 | 2311004 | + | *cobI* | *PA14_26510* | precorrin-2 C(20)-methyltransferase | 39 | 49 | 40 | 34 | 43 | 36 | 1 |
| 2151 | - | 2310997 | 2312676 | + | *cobJ* | *PA14_26530* | precorrin-3 methylase CobJ | 55 | 61 | 56 | 47 | 63 | 48 | 1 |
| 2152 | - | 2312746 | 2313594 | + | *-* | *PA14_26540* | hypothetical protein | 70 | 68 | 76 | 72 | 59 | 59 | 1 |
| 2153 | - | 2313591 | 2313950 | + | *-* | *PA14_26550* | lipoprotein | 143 | 153 | 186 | 166 | 124 | 117 | 1 |
| 2154 | - | 2313950 | 2314759 | + | *-* | *PA14_26560* | outer membrane protein | 150 | 157 | 162 | 145 | 122 | 119 | 1 |
| 2155 | - | 2314953 | 2315591 | + | *-* | *PA14_26570* | transcriptional regulator | 90 | 66 | 86 | 85 | 79 | 88 | 1 |
| 2156 | - | 2315677 | 2316045 | + | *-* | *PA14_26580* | hypothetical protein | 14 | 8 | 10 | 9 | 8 | 9 | 0.283677495 |
| 2157 | - | 2316267 | 2317706 | + | *-* | *PA14_26590* | GntR family transcriptional regulator | 138 | 128 | 138 | 140 | 133 | 151 | 1 |
| 2158 | - | 2318037 | 2318621 | + | *-* | *PA14_26600* | RNA polymerase sigma factor | 88 | 78 | 97 | 75 | 80 | 78 | 1 |
| 2159 | - | 2318660 | 2319382 | + | *-* | *PA14_26610* | hypothetical protein | 126 | 103 | 157 | 115 | 116 | 122 | 1 |
| 2160 | - | 2319728 | 2320150 | + | *-* | *PA14_26620* | hypothetical protein | 72 | 83 | 92 | 96 | 80 | 89 | 1 |
| 2161 | - | 2321990 | 2320164 | - | *-* | *PA14_26640* | long-chain-acyl-CoA synthetase | 30 | 30 | 32 | 35 | 27 | 33 | 1 |
| 2162 | - | 2322977 | 2322153 | - | *-* | *PA14_26650* | short chain dehydrogenase | 13 | 14 | 13 | 14 | 12 | 17 | 1 |
| 2163 | - | 2325014 | 2323029 | - | *-* | *PA14_26670* | biotin carboxylase | 21 | 16 | 15 | 17 | 13 | 21 | 1 |
| 2164 | - | 2325854 | 2325060 | - | *-* | *PA14_26690* | enoyl-CoA hydratase/isomerase | 15 | 12 | 10 | 11 | 10 | 13 | 1 |
| 2165 | - | 2327035 | 2325875 | - | *-* | *PA14_26700* | acyl-CoA dehydrogenase | 22 | 25 | 18 | 24 | 21 | 30 | 1 |
| 2166 | - | 2328768 | 2327152 | - | *-* | *PA14_26720* | biotin-dependent carboxylase | 20 | 16 | 15 | 18 | 13 | 20 | 1 |
| 2167 | - | 2329648 | 2328770 | - | *-* | *PA14_26730* | short-chain dehydrogenase | 19 | 16 | 14 | 18 | 14 | 19 | 1 |
| 2168 | - | 2331523 | 2329721 | - | *-* | *PA14_26750* | hypothetical protein | 11 | 9 | 8 | 11 | 9 | 11 | 1 |
| 2169 | - | 2331804 | 2332400 | + | *-* | *PA14_26760* | transcriptional regulator | 205 | 192 | 195 | 164 | 199 | 180 | 1 |
| 2170 | - | 2333165 | 2332401 | - | *-* | *PA14_26770* | hypothetical protein | 65 | 70 | 90 | 69 | 78 | 75 | 1 |
| 2171 | - | 2333565 | 2333398 | - | *-* | *PA14_26780* | hypothetical protein | 694 | 774 | 947 | 775 | 829 | 767 | 1 |
| 2172 | - | 2333988 | 2335103 | + | *-* | *PA14_26810* | two-component sensor | 3 | 3 | 5 | 3 | 3 | 2 | 1 |
| 2173 | - | 2335105 | 2336016 | + | *-* | *PA14_26830* | two-component response regulator | 4 | 5 | 6 | 4 | 4 | 4 | 1 |
| 2174 | - | 2336540 | 2336025 | - | *-* | *PA14_26850* | hypothetical protein | 3 | 4 | 5 | 4 | 4 | 4 | 1 |
| 2175 | - | 2336587 | 2337477 | + | *-* | *PA14_26860* | LysR family transcriptional regulator | 102 | 102 | 88 | 73 | 103 | 98 | 1 |
| 2176 | - | 2338141 | 2337500 | - | *-* | *PA14_26870* | hypothetical protein | 47 | 41 | 47 | 35 | 49 | 48 | 1 |
| 2177 | - | 2338187 | 2339080 | + | *-* | *PA14_26880* | LysR family transcriptional regulator | 30 | 28 | 30 | 26 | 26 | 29 | 1 |
| 2178 | - | 2339177 | 2339875 | + | *pyrF* | *PA14_26890* | orotidine 5'-phosphate decarboxylase | 36 | 36 | 40 | 35 | 32 | 28 | 1 |
| 2179 | - | 2340124 | 2341041 | + | *-* | *PA14_26910* | hypothetical protein | 42 | 43 | 51 | 50 | 40 | 47 | 1 |
| 2180 | - | 2341041 | 2341997 | + | *-* | *PA14_26920* | hypothetical protein | 44 | 55 | 46 | 51 | 51 | 43 | 1 |
| 2181 | - | 2341994 | 2344000 | + | *-* | *PA14_26930* | transglutaminase | 41 | 47 | 42 | 51 | 47 | 42 | 1 |
| 2182 | - | 2343997 | 2344767 | + | *-* | *PA14_26940* | hypothetical protein | 73 | 82 | 86 | 81 | 71 | 70 | 1 |
| 2183 | - | 2344848 | 2345645 | + | *-* | *PA14_26960* | hypothetical protein | 59 | 58 | 65 | 67 | 63 | 51 | 1 |
| 2184 | - | 2347231 | 2345654 | - | *-* | *PA14_26970* | hypothetical protein | 29 | 28 | 34 | 32 | 27 | 30 | 1 |

|  | A | B | C | D | E | F | G | H | I | J | K | L | M | N |
| --- | --- | --- | --- | --- | --- | --- | --- | --- | --- | --- | --- | --- | --- | --- |
| 2185 | - | 2347729 | 2347241 | - | *-* | *PA14_26980* | hypothetical protein | 16 | 11 | 15 | 11 | 10 | 17 | 1 |
| 2186 | - | 2348175 | 2347816 | - | *-* | *PA14_26990* | hypothetical protein | 55 | 24 | 24 | 21 | 18 | 64 | 0.020457471 |
| 2187 | - | 2349773 | 2348301 | - | *-* | *PA14_27000* | chemotaxis transducer | 223 | 181 | 261 | 182 | 148 | 198 | 1 |
| 2188 | - | 2350715 | 2349912 | - | *mttC* | *PA14_27020* | secretion protein MttC | 27 | 22 | 29 | 25 | 22 | 25 | 1 |
| 2189 | - | 2350962 | 2352392 | + | *-* | *PA14_27050* | Slt family transglycosylase | 37 | 30 | 43 | 36 | 31 | 33 | 1 |
| 2190 | - | 2352491 | 2352925 | + | *-* | *PA14_27070* | hypothetical protein | 80 | 96 | 98 | 84 | 80 | 89 | 1 |
| 2191 | - | 2353959 | 2353093 | - | *lipH* | *PA14_27090* | lipase chaperone | 5 | 8 | 8 | 8 | 8 | 10 | 1 |
| 2192 | - | 2355101 | 2354166 | - | *lipA* | *PA14_27100* | lactonizing lipase | 5 | 9 | 7 | 7 | 7 | 6 | 1 |
| 2193 | - | 2355845 | 2355297 | - | *ligT* | *PA14_27110* | 2'-5' RNA ligase | 7 | 6 | 8 | 5 | 5 | 7 | 1 |
| 2194 | - | 2355899 | 2356339 | + | *-* | *PA14_27120* | hypothetical protein | 66 | 65 | 74 | 79 | 63 | 61 | 1 |
| 2195 | - | 2356858 | 2356352 | - | *greB* | *PA14_27130* | transcription elongation factor GreB | 65 | 65 | 68 | 56 | 58 | 53 | 1 |
| 2196 | - | 2359402 | 2356910 | - | *-* | *PA14_27140* | hypothetical protein | 40 | 37 | 37 | 38 | 36 | 32 | 1 |
| 2197 | - | 2360094 | 2359411 | - | *-* | *PA14_27150* | ABC transporter ATP-binding protein | 41 | 28 | 36 | 27 | 25 | 26 | 1 |
| 2198 | - | 2360105 | 2360710 | + | *tesA* | *PA14_27160* | acyl-CoA thioesterase | 71 | 80 | 87 | 88 | 80 | 68 | 1 |
| 2199 | - | 2360766 | 2361044 | + | *-* | *PA14_27170* | hypothetical protein | 25 | 29 | 26 | 26 | 29 | 23 | 1 |
| 2200 | - | 2361122 | 2362093 | + | *-* | *PA14_27180* | hypothetical protein | 189 | 142 | 191 | 135 | 139 | 164 | 1 |
| 2201 | - | 2362974 | 2362888 | - | *-* | *PA14_27190* | Ser tRNA | 110 | 70 | 128 | 96 | 39 | 48 | 0.601884591 |
| 2202 | - | 2363130 | 2364260 | + | *-* | *PA14_27200* | hypothetical protein | 42 | 30 | 39 | 34 | 30 | 29 | 1 |
| 2203 | - | 2364303 | 2364869 | + | *efp* | *PA14_27210* | elongation factor P | 137 | 192 | 135 | 85 | 109 | 81 | 1 |
| 2204 | - | 2365385 | 2364957 | - | *ohr* | *PA14_27220* | organic hydroperoxide resistance protein | 19 | 28 | 17 | 15 | 18 | 12 | 1 |
| 2205 | - | 2365985 | 2365530 | - | *-* | *PA14_27230* | MarR family transcriptional regulator | 22 | 26 | 20 | 20 | 15 | 16 | 1 |
| 2206 | - | 2367090 | 2366071 | - | *-* | *PA14_27250* | LysR family transcriptional regulator | 12 | 9 | 10 | 9 | 11 | 10 | 1 |
| 2207 | - | 2367128 | 2367877 | + | *-* | *PA14_27270* | hypothetical protein | 36 | 24 | 30 | 30 | 31 | 29 | 1 |
| 2208 | - | 2368733 | 2367879 | - | *-* | *PA14_27280* | LysR family transcriptional regulator | 30 | 29 | 26 | 25 | 28 | 28 | 1 |
| 2209 | - | 2368830 | 2369063 | + | *-* | *PA14_27290* | hypothetical protein | 5 | 7 | 8 | 5 | 7 | 7 | 1 |
| 2210 | - | 2369146 | 2370354 | + | *-* | *PA14_27310* | hypothetical protein | 29 | 29 | 34 | 34 | 27 | 30 | 1 |
| 2211 | - | 2371741 | 2370395 | - | *-* | *PA14_27330* | phospho-2-dehydro-3-deoxyheptonate aldolase | 67 | 38 | 47 | 47 | 43 | 55 | 1 |
| 2212 | - | 2371917 | 2372666 | + | *-* | *PA14_27350* | hypothetical protein | 40 | 45 | 47 | 49 | 43 | 36 | 1 |
| 2213 | - | 2373484 | 2372693 | - | *-* | *PA14_27360* | enoyl-CoA hydratase | 35 | 38 | 43 | 40 | 35 | 32 | 1 |
| 2214 | - | 2373795 | 2375498 | + | *-* | *PA14_27370* | ATP-dependent RNA helicase | 22 | 15 | 40 | 16 | 16 | 13 | 1 |
| 2215 | - | 2376635 | 2375865 | - | *-* | *PA14_27390* | hypothetical protein | 11 | 12 | 12 | 7 | 10 | 10 | 1 |
| 2216 | - | 2377697 | 2376726 | - | *-* | *PA14_27400* | LysR family transcriptional regulator | 11 | 12 | 10 | 11 | 7 | 10 | 1 |
| 2217 | - | 2379173 | 2377734 | - | *-* | *PA14_27410* | outer membrane protein | 4 | 3 | 5 | 5 | 3 | 3 | 1 |
| 2218 | - | 2380234 | 2379170 | - | *-* | *PA14_27420* | secretion protein | 5 | 5 | 7 | 8 | 4 | 6 | 1 |
| 2219 | - | 2381838 | 2380246 | - | *-* | *PA14_27430* | multidrug efflux MFS transporter | 3 | 3 | 3 | 5 | 2 | 4 | 1 |
| 2220 | - | 2382941 | 2381976 | - | *-* | *PA14_27440* | transcriptional regulator | 11 | 9 | 11 | 9 | 7 | 11 | 1 |
| 2221 | - | 2383065 | 2383511 | + | *-* | *PA14_27450* | hypothetical protein | 31 | 32 | 31 | 30 | 27 | 24 | 1 |
| 2222 | - | 2383567 | 2384223 | + | *tpm* | *PA14_27460* | thiopurine S-methyltransferase | 148 | 135 | 122 | 152 | 123 | 110 | 1 |
| 2223 | - | 2385382 | 2384255 | - | *-* | *PA14_27470* | zinc carboxypeptidase | 46 | 49 | 45 | 43 | 45 | 37 | 1 |
| 2224 | - | 2386397 | 2385522 | - | *htpX* | *PA14_27480* | heat shock protein HtpX | 196 | 183 | 230 | 125 | 174 | 145 | 1 |
| 2225 | - | 2386983 | 2386531 | - | *-* | *PA14_27490* | hypothetical protein | 15 | 16 | 22 | 16 | 12 | 16 | 1 |
| 2226 | - | 2388245 | 2387034 | - | *-* | *PA14_27500* | aminotransferase | 28 | 27 | 29 | 27 | 23 | 22 | 1 |
| 2227 | - | 2388430 | 2388828 | + | *-* | *PA14_27510* | methionine sulfoxide reductase B | 256 | 254 | 269 | 245 | 204 | 264 | 1 |
| 2228 | - | 2388940 | 2389425 | + | *-* | *PA14_27520* | glutathione peroxidase | 58 | 79 | 60 | 55 | 64 | 60 | 1 |
| 2229 | - | 2389422 | 2389913 | + | *-* | *PA14_27530* | MarR family transcriptional regulator | 85 | 94 | 79 | 73 | 85 | 89 | 1 |
| 2230 | - | 2392292 | 2389932 | - | *-* | *PA14_27550* | sensor/response regulator hybrid | 61 | 55 | 68 | 70 | 61 | 68 | 1 |
| 2231 | - | 2392375 | 2393265 | + | *-* | *PA14_27560* | hypothetical protein | 48 | 51 | 48 | 47 | 40 | 39 | 1 |
| 2232 | - | 2393262 | 2393744 | + | *-* | *PA14_27570* | GAF domain-containing protein | 108 | 120 | 103 | 111 | 93 | 92 | 1 |
| 2233 | - | 2394416 | 2393754 | - | *-* | *PA14_27580* | glutathione S-transferase | 66 | 78 | 67 | 70 | 65 | 66 | 1 |
| 2234 | - | 2395251 | 2394478 | - | *-* | *PA14_27590* | hypothetical protein | 77 | 64 | 96 | 53 | 62 | 72 | 1 |
| 2235 | - | 2395469 | 2395541 | + | *-* | *PA14_27600* | Glu tRNA | 173 | 26 | 57 | 28 | 22 | 21 | 4.60686E-27 |
| 2236 | - | 2395547 | 2395619 | + | *-* | *PA14_27610* | Gly tRNA | 245 | 101 | 202 | 98 | 49 | 70 | 0.00634821 |
| 2237 | - | 2395707 | 2395779 | + | *-* | *PA14_27620* | Gly tRNA | 112 | 46 | 95 | 48 | 21 | 33 | 0.003088724 |
| 2238 | - | 2396312 | 2395992 | - | *-* | *PA14_27630* | protein associated with synthesis and assembly of refractile inclusion bodies | 5 | 7 | 4 | 5 | 4 | 6 | 1 |
| 2239 | - | 2396695 | 2396375 | - | *-* | *PA14_27640* | protein associated with synthesis and assembly of refractile inclusion bodies | 18 | 25 | 23 | 23 | 17 | 21 | 1 |
| 2240 | - | 2397391 | 2396846 | - | *-* | *PA14_27650* | hypothetical protein | 18 | 17 | 21 | 22 | 15 | 19 | 1 |
| 2241 | - | 2397684 | 2397445 | - | *-* | *PA14_27660* | hypothetical protein | 3 | 5 | 4 | 3 | 3 | 2 | 0.655993826 |
| 2242 | - | 2400054 | 2397913 | - | *-* | *PA14_27675* | hypothetical protein | 9 | 9 | 9 | 10 | 8 | 9 | 1 |
| 2243 | - | 2400737 | 2400105 | - | *-* | *PA14_27680* | hypothetical protein | 4 | 3 | 4 | 5 | 3 | 3 | 1 |
| 2244 | - | 2401672 | 2401079 | - | *-* | *PA14_27690* | RNA polymerase sigma factor | 30 | 28 | 32 | 38 | 32 | 32 | 1 |
| 2245 | - | 2402463 | 2401738 | - | *-* | *PA14_27700* | transcriptional regulator | 67 | 68 | 87 | 90 | 65 | 67 | 1 |
| 2246 | - | 2403137 | 2402727 | - | *-* | *PA14_27710* | hypothetical protein | 43 | 45 | 55 | 47 | 42 | 39 | 1 |
| 2247 | - | 2403576 | 2403196 | - | *-* | *PA14_27720* | hypothetical protein | 24 | 24 | 28 | 22 | 20 | 21 | 1 |
| 2248 | - | 2406152 | 2403705 | - | *fadE* | *PA14_27730* | acyl-CoA dehydrogenase | 151 | 159 | 164 | 178 | 155 | 170 | 1 |
| 2249 | - | 2406305 | 2406976 | + | *-* | *PA14_27740* | hypothetical protein | 38 | 38 | 47 | 29 | 42 | 43 | 1 |
| 2250 | - | 2407091 | 2407711 | + | *-* | *PA14_27755* | glutathione S-transferase | 200 | 191 | 232 | 244 | 191 | 201 | 1 |
| 2251 | - | 2407787 | 2408719 | + | *-* | *PA14_27770* | ABC transporter ATP-binding protein | 43 | 48 | 47 | 43 | 44 | 43 | 1 |
| 2252 | - | 2408716 | 2409495 | + | *-* | *PA14_27780* | ABC transporter permease | 54 | 65 | 55 | 55 | 70 | 50 | 1 |
| 2253 | - | 2410876 | 2409545 | - | *-* | *PA14_27800* | two-component sensor | 43 | 38 | 47 | 48 | 36 | 35 | 1 |
| 2254 | - | 2411553 | 2410873 | - | *-* | *PA14_27810* | two-component response regulator | 28 | 26 | 24 | 27 | 22 | 19 | 1 |
| 2255 | - | 2411679 | 2411870 | + | *-* | *PA14_27830* | hypothetical protein | 8 | 11 | 10 | 14 | 8 | 8 | 1 |
| 2256 | - | 2412042 | 2412659 | + | *-* | *PA14_27840* | hypothetical protein | 6 | 6 | 7 | 7 | 5 | 4 | 1 |
| 2257 | - | 2412777 | 2413607 | + | *queF* | *PA14_27850* | 7-cyano-7-deazaguanine reductase | 21 | 19 | 22 | 19 | 17 | 19 | 1 |
| 2258 | - | 2413937 | 2413674 | - | *-* | *PA14_27870* | hypothetical protein | 110 | 166 | 140 | 86 | 128 | 110 | 1 |
| 2259 | - | 2414592 | 2414011 | - | *-* | *PA14_27880* | hypothetical protein | 16 | 12 | 16 | 18 | 15 | 17 | 1 |
| 2260 | - | 2415332 | 2414589 | - | *-* | *PA14_27890* | hypothetical protein | 12 | 12 | 16 | 14 | 9 | 11 | 1 |
| 2261 | - | 2415466 | 2416185 | + | *-* | *PA14_27900* | transcriptional regulator | 43 | 42 | 48 | 47 | 37 | 36 | 1 |
| 2262 | - | 2416591 | 2416187 | - | *-* | *PA14_27910* | hypothetical protein | 33 | 34 | 29 | 34 | 28 | 30 | 1 |
| 2263 | - | 2416694 | 2417398 | + | *-* | *PA14_27920* | hypothetical protein | 114 | 107 | 123 | 110 | 103 | 125 | 1 |
| 2264 | - | 2417755 | 2417456 | - | *-* | *PA14_27930* | hypothetical protein | 132 | 142 | 159 | 133 | 151 | 162 | 1 |
| 2265 | - | 2418001 | 2419185 | + | *-* | *PA14_27940* | two-component response regulator | 200 | 170 | 205 | 199 | 175 | 184 | 1 |
| 2266 | - | 2419182 | 2419664 | + | *-* | *PA14_27950* | hypothetical protein | 265 | 271 | 320 | 317 | 308 | 296 | 1 |
| 2267 | - | 2420673 | 2419750 | - | *tal* | *PA14_27960* | transaldolase B | 105 | 117 | 84 | 85 | 110 | 100 | 1 |
| 2268 | - | 2421751 | 2420753 | - | *-* | *PA14_27980* | tRNA-dihydrouridine synthase A | 21 | 17 | 21 | 18 | 18 | 19 | 1 |

|  | A | B | C | D | E | F | G | H | I | J | K | L | M | N |
| --- | --- | --- | --- | --- | --- | --- | --- | --- | --- | --- | --- | --- | --- | --- |
| 2269 | - | 2422054 | 2423370 | + | *-* | *PA14_27990* | sialidase | 52 | 51 | 49 | 70 | 54 | 62 | 1 |
| 2270 | - | 2423812 | 2424852 | + | *-* | *PA14_28000* | hypothetical protein | 16 | 15 | 19 | 15 | 14 | 13 | 1 |
| 2271 | - | 2424932 | 2425528 | + | *-* | *PA14_28010* | hypothetical protein | 10 | 8 | 9 | 7 | 11 | 6 | 1 |
| 2272 | - | 2425622 | 2425912 | + | *-* | *PA14_28020* | hypothetical protein | 15 | 10 | 16 | 11 | 12 | 14 | 1 |
| 2273 | - | 2426498 | 2426013 | - | *-* | *PA14_28030* | hypothetical protein | 133 | 105 | 133 | 103 | 115 | 120 | 1 |
| 2274 | - | 2428012 | 2426933 | - | *-* | *PA14_28040* | hypothetical protein | 6 | 7 | 7 | 5 | 4 | 4 | 1 |
| 2275 | - | 2428256 | 2429851 | + | *-* | *PA14_28050* | chemotaxis transducer | 327 | 239 | 283 | 339 | 409 | 428 | 1 |
| 2276 | - | 2431111 | 2429873 | - | *cpg2* | *PA14_28060* | glutamate carboxypeptidase | 109 | 78 | 90 | 132 | 113 | 137 | 1 |
| 2277 | - | 2431645 | 2431178 | - | *-* | *PA14_28070* | hypothetical protein | 7 | 7 | 7 | 11 | 8 | 10 | 1 |
| 2278 | - | 2431978 | 2431760 | - | *-* | *PA14_28080* | transcriptional regulator | 7 | 4 | 4 | 6 | 4 | 3 | 1 |
| 2279 | - | 2432544 | 2431987 | - | *-* | *PA14_28090* | hypothetical protein | 6 | 7 | 7 | 9 | 6 | 9 | 1 |
| 2280 | - | 2434523 | 2432715 | - | *-* | *PA14_28100* | hypothetical protein | 10 | 8 | 11 | 10 | 9 | 9 | 1 |
| 2281 | - | 2435260 | 2434601 | - | *-* | *PA14_28110* | hypothetical protein | 9 | 7 | 11 | 10 | 9 | 10 | 1 |
| 2282 | - | 2436079 | 2435738 | - | *-* | *PA14_28120* | hypothetical protein | 23 | 26 | 26 | 31 | 20 | 23 | 1 |
| 2283 | - | 2436420 | 2436076 | - | *-* | *PA14_28130* | hypothetical protein | 15 | 12 | 11 | 15 | 11 | 12 | 1 |
| 2284 | - | 2436828 | 2437226 | + | *-* | *PA14_28140* | hypothetical protein | 109 | 115 | 130 | 116 | 137 | 145 | 1 |
| 2285 | - | 2437310 | 2438188 | + | *-* | *PA14_28150* | hypothetical protein | 55 | 49 | 72 | 56 | 56 | 65 | 1 |
| 2286 | - | 2438270 | 2439226 | + | *-* | *PA14_28170* | formate/nitrate transporter | 72 | 84 | 97 | 96 | 83 | 79 | 1 |
| 2287 | - | 2440560 | 2439277 | - | *-* | *PA14_28180* | hypothetical protein | 51 | 65 | 58 | 53 | 53 | 55 | 1 |
| 2288 | - | 2440744 | 2440817 | + | *-* | *PA14_28190* | Val tRNA | 2 | 0 | 2 | 0 | 0 | 0 | 0.007235124 |
| 2289 | - | 2441012 | 2441449 | + | *-* | *PA14_28200* | hypothetical protein | 27 | 18 | 31 | 30 | 17 | 16 | 0.87182855 |
| 2290 | - | 2441454 | 2442041 | + | *-* | *PA14_28210* | hypothetical protein | 43 | 35 | 41 | 30 | 38 | 29 | 1 |
| 2291 | - | 2442240 | 2442761 | + | *-* | *PA14_28220* | hypothetical protein | 25 | 25 | 23 | 27 | 21 | 18 | 1 |
| 2292 | - | 2442951 | 2442781 | - | *-* | *PA14_28230* | hypothetical protein | 27 | 38 | 28 | 32 | 24 | 21 | 1 |
| 2293 | - | 2443112 | 2443336 | + | *-* | *PA14_28240* | hypothetical protein | 71 | 78 | 89 | 103 | 70 | 66 | 1 |
| 2294 | 2444398 | 2444509 | 2443370 | - | *-* | *PA14_28250* | secreted acid phosphatase | 62 | 67 | 78 | 77 | 62 | 55 | 1 |
| 2295 | 2444398 | 2444516 | 2444812 | + | *-* | *PA14_28260* | hypothetical protein | 113 | 91 | 98 | 144 | 107 | 164 | 1 |
| 2296 | - | 2444914 | 2445693 | + | *-* | *PA14_28280* | PhzF family phenazine biosynthesis protein | 59 | 59 | 53 | 53 | 61 | 52 | 1 |
| 2297 | - | 2445715 | 2446125 | + | *-* | *PA14_28290* | hypothetical protein | 43 | 58 | 60 | 57 | 54 | 52 | 1 |
| 2298 | - | 2446747 | 2446265 | - | *-* | *PA14_28300* | hypothetical protein | 8 | 9 | 6 | 8 | 7 | 7 | 1 |
| 2299 | - | 2447721 | 2446753 | - | *-* | *PA14_28310* | enoyl-CoA hydratase | 10 | 8 | 12 | 9 | 7 | 8 | 1 |
| 2300 | - | 2447809 | 2448390 | + | *-* | *PA14_28320* | TetR family transcriptional regulator | 14 | 21 | 17 | 19 | 21 | 21 | 1 |
| 2301 | - | 2448449 | 2448544 | + | *-* | *PA14_28330* | hypothetical protein | 13 | 14 | 13 | 16 | 15 | 15 | 1 |
| 2302 | - | 2448732 | 2449631 | + | *-* | *PA14_28340* | hypothetical protein | 51 | 68 | 46 | 46 | 58 | 38 | 1 |
| 2303 | - | 2450374 | 2449679 | - | *-* | *PA14_28350* | hypothetical protein | 24 | 33 | 28 | 33 | 27 | 27 | 1 |
| 2304 | - | 2450705 | 2450914 | + | *-* | *PA14_28360* | hypothetical protein | 77 | 13 | 34 | 46 | 36 | 92 | 1.69482E-25 |
| 2305 | - | 2451388 | 2451597 | + | *-* | *PA14_28370* | hypothetical protein | 12 | 9 | 12 | 12 | 10 | 12 | 1 |
| 2306 | - | 2451709 | 2452053 | + | *-* | *PA14_28380* | hypothetical protein | 20 | 18 | 19 | 21 | 23 | 21 | 1 |
| 2307 | - | 2452512 | 2452096 | - | *-* | *PA14_28390* | hypothetical protein | 10 | 10 | 12 | 13 | 14 | 10 | 1 |
| 2308 | - | 2453901 | 2452624 | - | *-* | *PA14_28400* | outer membrane OprD family porin | 179 | 252 | 185 | 134 | 160 | 137 | 1 |
| 2309 | - | 2454266 | 2454580 | + | *-* | *PA14_28410* | hypothetical protein | 103 | 101 | 150 | 67 | 27 | 38 | 1 |
| 2310 | - | 2455678 | 2454791 | - | *-* | *PA14_28420* | LysR family transcriptional regulator | 12 | 17 | 15 | 13 | 12 | 14 | 1 |
| 2311 | - | 2455781 | 2456224 | + | *-* | *PA14_28430* | hypothetical protein | 3 | 7 | 5 | 3 | 3 | 3 | 0.515742977 |
| 2312 | - | 2456363 | 2456797 | + | *-* | *PA14_28440* | hypothetical protein | 577 | 429 | 578 | 679 | 559 | 537 | 1 |
| 2313 | - | 2457319 | 2456849 | - | *eco* | *PA14_28450* | ecotin | 58 | 101 | 57 | 47 | 59 | 33 | 0.680948229 |
| 2314 | - | 2457898 | 2457668 | - | *-* | *PA14_28460* | hypothetical protein | 94 | 116 | 111 | 115 | 92 | 98 | 1 |
| 2315 | - | 2458077 | 2458274 | + | *-* | *PA14_28470* | hypothetical protein | 58 | 79 | 76 | 48 | 64 | 61 | 1 |
| 2316 | - | 2458669 | 2458340 | - | *-* | *PA14_28490* | hypothetical protein | 240 | 280 | 285 | 154 | 225 | 236 | 1 |
| 2317 | - | 2459154 | 2458780 | - | *-* | *PA14_28500* | hypothetical protein | 702 | 779 | 802 | 358 | 414 | 679 | 1 |
| 2318 | - | 2459289 | 2459732 | + | *-* | *PA14_28510* | hypothetical protein | 15 | 14 | 16 | 15 | 10 | 11 | 1 |
| 2319 | - | 2459780 | 2460205 | + | *-* | *PA14_28520* | hypothetical protein | 51 | 42 | 53 | 57 | 48 | 50 | 1 |
| 2320 | - | 2461094 | 2460195 | - | *-* | *PA14_28530* | hypothetical protein | 33 | 25 | 35 | 35 | 31 | 30 | 1 |
| 2321 | - | 2461317 | 2461550 | + | *-* | *PA14_28540* | hypothetical protein | 16 | 18 | 16 | 15 | 11 | 14 | 1 |
| 2322 | - | 2462339 | 2461821 | - | *-* | *PA14_28560* | hypothetical protein | 65 | 52 | 71 | 70 | 53 | 57 | 1 |
| 2323 | - | 2463071 | 2462358 | - | *endA* | *PA14_28570* | DNA-specific endonuclease I | 43 | 35 | 40 | 50 | 39 | 45 | 1 |
| 2324 | - | 2463228 | 2463449 | + | *-* | *PA14_28580* | hypothetical protein | 25 | 18 | 25 | 26 | 23 | 23 | 1 |
| 2325 | - | 2463446 | 2464228 | + | *-* | *PA14_28590* | methionine aminopeptidase | 36 | 35 | 46 | 43 | 31 | 34 | 1 |
| 2326 | - | 2464714 | 2465001 | + | *-* | *PA14_28600* | hypothetical protein | 373 | 286 | 336 | 288 | 210 | 280 | 1 |
| 2327 | - | 2465503 | 2465327 | - | *-* | *PA14_28610* | hypothetical protein | 243 | 267 | 310 | 255 | 264 | 272 | 1 |
| 2328 | - | 2465914 | 2465588 | - | *-* | *PA14_28620* | hypothetical protein | 65 | 79 | 108 | 77 | 76 | 63 | 1 |
| 2329 | - | 2466106 | 2466969 | + | *-* | *PA14_28630* | hydrolase | 29 | 19 | 29 | 24 | 18 | 19 | 1 |
| 2330 | - | 2467219 | 2469141 | + | *thrS* | *PA14_28650* | threonyl-tRNA synthetase | 148 | 176 | 148 | 133 | 145 | 124 | 1 |
| 2331 | - | 2469159 | 2469692 | + | *infC* | *PA14_28660* | translation initiation factor IF-3 | 620 | 696 | 604 | 543 | 531 | 479 | 1 |
| 2332 | - | 2469754 | 2469948 | + | *rpmI* | *PA14_28670* | 50S ribosomal protein L35 | 1416 | 1689 | 1057 | 1084 | 1267 | 900 | 1 |
| 2333 | - | 2469972 | 2470328 | + | *rplT* | *PA14_28680* | 50S ribosomal protein L20 | 1346 | 1424 | 1150 | 910 | 1089 | 731 | 1 |
| 2334 | - | 2470426 | 2471442 | + | *pheS* | *PA14_28690* | phenylalanyl-tRNA synthetase subunit alpha | 53 | 77 | 55 | 47 | 58 | 38 | 1 |
| 2335 | - | 2471477 | 2473855 | + | *pheT* | *PA14_28710* | phenylalanyl-tRNA synthetase subunit beta | 113 | 129 | 100 | 93 | 102 | 85 | 1 |
| 2336 | - | 2473859 | 2474161 | + | *ihfA* | *PA14_28720* | integration host factor subunit alpha | 3260 | 2838 | 3279 | 2459 | 2122 | 2316 | 1 |
| 2337 | - | 2474142 | 2474498 | + | *-* | *PA14_28730* | transcriptional regulator | 725 | 819 | 646 | 600 | 558 | 598 | 1 |
| 2338 | - | 2474588 | 2474661 | + | *-* | *PA14_28740* | Pro tRNA | 50 | 57 | 33 | 43 | 35 | 32 | 1 |
| 2339 | - | 2475199 | 2474825 | - | *-* | *PA14_28750* | hypothetical protein | 27 | 25 | 26 | 39 | 24 | 25 | 1 |
| 2340 | - | 2476088 | 2475474 | - | *-* | *PA14_28760* | hypothetical protein | 26 | 25 | 23 | 28 | 21 | 18 | 1 |
| 2341 | - | 2476671 | 2478314 | + | *-* | *PA14_28770* | hypothetical protein | 25 | 23 | 20 | 26 | 21 | 26 | 1 |
| 2342 | - | 2478590 | 2478874 | + | *-* | *PA14_28780* | hypothetical protein | 8 | 7 | 8 | 9 | 7 | 8 | 1 |
| 2343 | - | 2478975 | 2479136 | + | *-* | *PA14_28790* | hypothetical protein | 16 | 13 | 14 | 13 | 8 | 12 | 1 |
| 2344 | - | 2479279 | 2480274 | + | *-* | *PA14_28800* | hypothetical protein | 69 | 49 | 49 | 55 | 51 | 52 | 1 |
| 2345 | - | 2480409 | 2485532 | + | *-* | *PA14_28810* | DNA helicase | 203 | 169 | 164 | 195 | 182 | 192 | 1 |
| 2346 | - | 2485638 | 2488136 | + | *-* | *PA14_28820* | hypothetical protein | 228 | 148 | 168 | 214 | 197 | 214 | 1 |
| 2347 | - | 2488133 | 2489428 | + | *-* | *PA14_28830* | hypothetical protein | 415 | 311 | 301 | 377 | 342 | 383 | 1 |
| 2348 | - | 2489403 | 2491613 | + | *-* | *PA14_28840* | helicase | 258 | 217 | 206 | 248 | 254 | 262 | 1 |
| 2349 | - | 2491674 | 2491988 | + | *-* | *PA14_28850* | hypothetical protein | 1139 | 1147 | 928 | 1309 | 1112 | 1302 | 1 |
| 2350 | - | 2492021 | 2492317 | + | *-* | *PA14_28870* | hypothetical protein | 461 | 400 | 471 | 525 | 446 | 553 | 1 |
| 2351 | - | 2493942 | 2492593 | - | *-* | *PA14_28880* | hypothetical protein | 40 | 30 | 28 | 23 | 33 | 30 | 1 |
| 2352 | - | 2500235 | 2493939 | - | *-* | *PA14_28895* | hypothetical protein | 41 | 32 | 29 | 27 | 38 | 31 | 1 |

|  | A | B | C | D | E | F | G | H | I | J | K | L | M | N |
| --- | --- | --- | --- | --- | --- | --- | --- | --- | --- | --- | --- | --- | --- | --- |
| 2353 | - | 2500888 | 2500250 | - | *-* | *PA14_28910* | radical activating enzyme | 53 | 45 | 55 | 51 | 45 | 46 | 1 |
| 2354 | - | 2502773 | 2500890 | - | *-* | *PA14_28920* | chaperone | 43 | 39 | 36 | 33 | 41 | 33 | 1 |
| 2355 | - | 2503392 | 2502850 | - | *-* | *PA14_28930* | hypothetical protein | 10 | 6 | 7 | 7 | 9 | 8 | 0.895687725 |
| 2356 | - | 2503745 | 2503467 | - | *-* | *PA14_28940* | hypothetical protein | 63 | 54 | 61 | 36 | 54 | 67 | 1 |
| 2357 | - | 2504167 | 2503775 | - | *-* | *PA14_28950* | hypothetical protein | 19 | 15 | 20 | 25 | 18 | 26 | 1 |
| 2358 | - | 2504678 | 2504199 | - | *-* | *PA14_28960* | hypothetical protein | 28 | 26 | 37 | 37 | 25 | 33 | 1 |
| 2359 | - | 2504847 | 2505386 | + | *-* | *PA14_28970* | hypothetical protein | 18 | 32 | 13 | 55 | 21 | 12 | 0.828318293 |
| 2360 | - | 2505374 | 2506324 | + | *-* | *PA14_28980* | Fe2+-dicitrate sensor | 2 | 9 | 4 | 25 | 4 | 3 | 5.77642E-08 |
| 2361 | - | 2506753 | 2507391 | + | *-* | *PA14_28990* | hypothetical protein | 14 | 13 | 19 | 15 | 14 | 14 | 1 |
| 2362 | - | 2508237 | 2507551 | - | *-* | *PA14_29000* | hypothetical protein | 20 | 19 | 18 | 23 | 16 | 26 | 1 |
| 2363 | - | 2508825 | 2508337 | - | *-* | *PA14_29010* | MerR family transcriptional regulator | 47 | 38 | 50 | 46 | 45 | 45 | 1 |
| 2364 | - | 2509058 | 2509888 | + | *cpo* | *PA14_29020* | chloroperoxidase | 25 | 27 | 29 | 29 | 27 | 35 | 1 |
| 2365 | - | 2510015 | 2511250 | + | *-* | *PA14_29030* | FMN oxidoreductase | 20 | 17 | 19 | 18 | 17 | 19 | 1 |
| 2366 | - | 2511603 | 2511265 | - | *-* | *PA14_29040* | ferredoxin | 14 | 18 | 12 | 17 | 19 | 18 | 1 |
| 2367 | - | 2513929 | 2511620 | - | *-* | *PA14_29050* | molybdopterin oxidoreductase | 14 | 12 | 14 | 13 | 11 | 11 | 1 |
| 2368 | - | 2514283 | 2514762 | + | *-* | *PA14_29060* | transcriptional regulator | 71 | 59 | 62 | 67 | 62 | 58 | 1 |
| 2369 | - | 2515673 | 2514810 | - | *-* | *PA14_29070* | hypothetical protein | 31 | 27 | 33 | 31 | 27 | 29 | 1 |
| 2370 | - | 2517184 | 2516093 | - | *-* | *PA14_29090* | periplasmic spermidine/putrescine-binding protein | 11 | 9 | 10 | 9 | 10 | 11 | 1 |
| 2371 | - | 2517997 | 2517383 | - | *-* | *PA14_29100* | hypothetical protein | 37 | 45 | 36 | 41 | 42 | 35 | 1 |
| 2372 | - | 2519030 | 2518056 | - | *cysK* | *PA14_29110* | cysteine synthase A | 115 | 137 | 106 | 85 | 110 | 87 | 1 |
| 2373 | - | 2519151 | 2520236 | + | *-* | *PA14_29120* | hypothetical protein | 38 | 41 | 47 | 44 | 47 | 49 | 1 |
| 2374 | - | 2520399 | 2521244 | + | *-* | *PA14_29130* | ATPase | 107 | 163 | 100 | 107 | 154 | 142 | 1 |
| 2375 | - | 2521309 | 2521704 | + | *-* | *PA14_29150* | hypothetical protein | 93 | 124 | 81 | 91 | 103 | 116 | 1 |
| 2376 | - | 2521753 | 2522934 | + | *-* | *PA14_29160* | hypothetical protein | 118 | 152 | 100 | 137 | 132 | 155 | 1 |
| 2377 | - | 2523048 | 2524067 | + | *-* | *PA14_29180* | AraC family transcriptional regulator | 18 | 19 | 18 | 19 | 15 | 19 | 1 |
| 2378 | - | 2524582 | 2524349 | - | *-* | *PA14_29190* | hypothetical protein | 8 | 11 | 9 | 9 | 11 | 7 | 1 |
| 2379 | - | 2525068 | 2524592 | - | *-* | *PA14_29200* | hypothetical protein | 29 | 33 | 34 | 29 | 33 | 29 | 1 |
| 2380 | - | 2526804 | 2525215 | - | *-* | *PA14_29210* | MFS transporter | 8 | 8 | 11 | 15 | 6 | 9 | 1 |
| 2381 | - | 2528114 | 2526807 | - | *-* | *PA14_29220* | porin | 6 | 5 | 6 | 7 | 4 | 6 | 1 |
| 2382 | - | 2530001 | 2528163 | - | *-* | *PA14_29230* | hypothetical protein | 16 | 19 | 17 | 19 | 19 | 23 | 1 |
| 2383 | - | 2530609 | 2531289 | + | *-* | *PA14_29240* | hydrolase | 15 | 19 | 12 | 14 | 17 | 14 | 1 |
| 2384 | - | 2531346 | 2531627 | + | *-* | *PA14_29250* | hypothetical protein | 10 | 10 | 9 | 10 | 9 | 10 | 1 |
| 2385 | - | 2531797 | 2532690 | + | *-* | *PA14_29260* | transcriptional regulator | 16 | 16 | 16 | 21 | 14 | 18 | 1 |
| 2386 | - | 2532804 | 2533907 | + | *-* | *PA14_29270* | outer membrane lipoprotein | 23 | 22 | 22 | 25 | 19 | 19 | 1 |
| 2387 | - | 2533988 | 2534314 | + | *-* | *PA14_29280* | thioredoxin | 47 | 46 | 57 | 46 | 39 | 43 | 1 |
| 2388 | - | 2534842 | 2534342 | - | *-* | *PA14_29290* | hypothetical protein | 13 | 10 | 13 | 13 | 14 | 15 | 1 |
| 2389 | - | 2535536 | 2535012 | - | *-* | *PA14_29300* | transcriptional regulator | 23 | 21 | 25 | 26 | 23 | 21 | 1 |
| 2390 | - | 2535805 | 2537010 | + | *-* | *PA14_29320* | NADH dehydrogenase, FAD-containing subunit | 38 | 63 | 48 | 67 | 46 | 39 | 0.831807123 |
| 2391 | - | 2538037 | 2537045 | - | *-* | *PA14_29330* | hypothetical protein | 115 | 110 | 143 | 143 | 117 | 148 | 1 |
| 2392 | - | 2539410 | 2538496 | - | *-* | *PA14_29340* | hypothetical protein | 3 | 3 | 3 | 3 | 2 | 3 | 1 |
| 2393 | - | 2541670 | 2539430 | - | *pfeA* | *PA14_29350* | outer membrane receptor FepA | 14 | 12 | 15 | 15 | 11 | 12 | 1 |
| 2394 | - | 2543109 | 2541769 | - | *pfeS* | *PA14_29360* | two-component sensor PfeS | 16 | 33 | 19 | 73 | 49 | 18 | 0.292421048 |
| 2395 | - | 2544083 | 2546152 | + | *-* | *PA14_29390* | hypothetical protein | 124 | 95 | 106 | 107 | 117 | 89 | 1 |
| 2396 | - | 2546202 | 2550155 | + | *-* | *PA14_29400* | hypothetical protein | 96 | 75 | 69 | 59 | 88 | 66 | 1 |
| 2397 | - | 2550506 | 2551468 | + | *-* | *PA14_29410* | serine/threonine dehydratase | 55 | 48 | 44 | 33 | 47 | 41 | 1 |
| 2398 | - | 2552717 | 2551476 | - | *-* | *PA14_29420* | hypothetical protein | 24 | 21 | 23 | 23 | 23 | 21 | 1 |
| 2399 | - | 2553829 | 2552900 | - | *-* | *PA14_29440* | LysR family transcriptional regulator | 14 | 15 | 13 | 14 | 16 | 13 | 1 |
| 2400 | - | 2554826 | 2553852 | - | *-* | *PA14_29460* | quinone oxidoreductase | 62 | 46 | 75 | 77 | 48 | 57 | 1 |
| 2401 | - | 2555052 | 2555804 | + | *-* | *PA14_29470* | hypothetical protein | 217 | 109 | 153 | 113 | 92 | 97 | 1 |
| 2402 | - | 2556397 | 2557200 | + | *-* | *PA14_29480* | ABC-2 transporter permease | 8 | 7 | 7 | 9 | 7 | 8 | 1 |
| 2403 | - | 2557190 | 2558917 | + | *-* | *PA14_29490* | type II secretion protein | 9 | 11 | 10 | 13 | 10 | 10 | 1 |
| 2404 | - | 2558922 | 2560109 | + | *-* | *PA14_29500* | type II secretion system protein | 5 | 5 | 4 | 6 | 4 | 4 | 1 |
| 2405 | - | 2560163 | 2560597 | + | *-* | *PA14_29510* | type II secretion system protein | 9 | 7 | 6 | 7 | 8 | 6 | 1 |
| 2406 | - | 2560566 | 2560976 | + | *-* | *PA14_29520* | type II secretion system protein | 9 | 8 | 7 | 9 | 8 | 7 | 1 |
| 2407 | - | 2560976 | 2561401 | + | *-* | *PA14_29530* | type II secretion system protein | 12 | 9 | 10 | 12 | 9 | 9 | 1 |
| 2408 | - | 2561398 | 2561988 | + | *-* | *PA14_29540* | type II secretion system protein | 4 | 5 | 3 | 3 | 5 | 3 | 1 |
| 2409 | - | 2561978 | 2563057 | + | *-* | *PA14_29550* | hypothetical protein | 10 | 9 | 9 | 11 | 9 | 10 | 1 |
| 2410 | - | 2563101 | 2564087 | + | *-* | *PA14_29560* | hypothetical protein | 15 | 15 | 16 | 20 | 14 | 16 | 1 |
| 2411 | - | 2564087 | 2564671 | + | *-* | *PA14_29570* | hypothetical protein | 11 | 10 | 12 | 10 | 10 | 9 | 1 |
| 2412 | - | 2564914 | 2565231 | + | *-* | *PA14_29575* | hypothetical protein | 24 | 29 | 24 | 19 | 25 | 22 | 1 |
| 2413 | - | 2565660 | 2565307 | - | *-* | *PA14_29590* | transcriptional regulator | 107 | 184 | 85 | 82 | 126 | 89 | 0.793216635 |
| 2414 | - | 2566324 | 2565968 | - | *-* | *PA14_29600* | 6-pyruvoyl-tetrahydropterin synthase | 31 | 31 | 39 | 33 | 29 | 26 | 1 |
| 2415 | 2568048 | 2567978 | 2566425 | - | *-* | *PA14_29620* | anaerobic nitric oxide reductase transcriptional regulator | 45 | 62 | 60 | 52 | 44 | 37 | 1 |
| 2416 | 2568048 | 2568133 | 2569314 | + | *fhp* | *PA14_29640* | nitric oxide dioxygenase | 10 | 404 | 9 | 336 | 550 | 40 | 0 |
| 2417 | 2568048 | 2569368 | 2569625 | + | *-* | *PA14_29650* | hypothetical protein | 28 | 491 | 29 | 172 | 291 | 63 | 1.4453E-232 |
| 2418 | 2568048 | 2569612 | 2570805 | + | *-* | *PA14_29660* | hypothetical protein | 50 | 565 | 52 | 293 | 491 | 142 | 7.35411E-59 |
| 2419 | - | 2571949 | 2571056 | - | *-* | *PA14_29680* | hypothetical protein | 21 | 118 | 24 | 58 | 106 | 45 | 1.38852E-14 |
| 2420 | - | 2573058 | 2571976 | - | *-* | *PA14_29690* | hypothetical protein | 15 | 28 | 20 | 19 | 27 | 20 | 0.415011252 |
| 2421 | - | 2573273 | 2573581 | + | *-* | *PA14_29710* | hypothetical protein | 131 | 147 | 136 | 120 | 122 | 124 | 1 |
| 2422 | - | 2573581 | 2573895 | + | *-* | *PA14_29720* | hypothetical protein | 53 | 69 | 66 | 56 | 55 | 53 | 1 |
| 2423 | - | 2573895 | 2574566 | + | *-* | *PA14_29730* | two-component response regulator | 79 | 77 | 86 | 76 | 69 | 70 | 1 |
| 2424 | - | 2574563 | 2575900 | + | *-* | *PA14_29740* | two-component sensor | 65 | 63 | 80 | 72 | 63 | 63 | 1 |
| 2425 | - | 2575991 | 2576299 | + | *-* | *PA14_29750* | hypothetical protein | 45 | 61 | 50 | 56 | 54 | 51 | 1 |
| 2426 | - | 2578448 | 2576304 | - | *-* | *PA14_29760* | chemotaxis transducer | 70 | 89 | 84 | 82 | 89 | 77 | 1 |
| 2427 | - | 2578707 | 2580032 | + | *-* | *PA14_29770* | transporter | 20 | 19 | 23 | 20 | 16 | 20 | 1 |
| 2428 | - | 2580146 | 2581831 | + | *-* | *PA14_29800* | chemotaxis transducer | 69 | 88 | 80 | 73 | 83 | 72 | 1 |
| 2429 | - | 2581991 | 2583049 | + | *-* | *PA14_29820* | hypothetical protein | 66 | 51 | 67 | 50 | 54 | 47 | 1 |
| 2430 | - | 2584065 | 2583256 | - | *-* | *PA14_29830* | methyltransferase | 21 | 19 | 24 | 23 | 20 | 21 | 1 |
| 2431 | - | 2585640 | 2584180 | - | *nuoN* | *PA14_29850* | NADH dehydrogenase subunit N | 160 | 173 | 148 | 143 | 153 | 149 | 1 |
| 2432 | - | 2587177 | 2585648 | - | *nuoM* | *PA14_29860* | NADH dehydrogenase subunit M | 191 | 211 | 189 | 177 | 183 | 180 | 1 |
| 2433 | - | 2589052 | 2587205 | - | *nuoL* | *PA14_29880* | NADH dehydrogenase subunit L | 163 | 186 | 167 | 153 | 169 | 162 | 1 |
| 2434 | - | 2589357 | 2589049 | - | *nuoK* | *PA14_29890* | NADH dehydrogenase subunit K | 164 | 167 | 158 | 132 | 159 | 162 | 1 |
| 2435 | - | 2589903 | 2589403 | - | *nuoJ* | *PA14_29900* | NADH dehydrogenase subunit J | 195 | 219 | 202 | 177 | 179 | 160 | 1 |
| 2436 | - | 2590463 | 2589915 | - | *nuoI* | *PA14_29920* | NADH dehydrogenase subunit I | 226 | 265 | 205 | 220 | 227 | 233 | 1 |

|  | A | B | C | D | E | F | G | H | I | J | K | L | M | N |
| --- | --- | --- | --- | --- | --- | --- | --- | --- | --- | --- | --- | --- | --- | --- |
| 2437 | - | 2591470 | 2590475 | - | *nuoH* | *PA14_29930* | NADH dehydrogenase subunit H | 223 | 239 | 215 | 192 | 219 | 210 | 1 |
| 2438 | - | 2594184 | 2591467 | - | *nuoG* | *PA14_29940* | NADH dehydrogenase subunit G | 270 | 311 | 271 | 269 | 253 | 257 | 1 |
| 2439 | - | 2595665 | 2594316 | - | *nuoF* | *PA14_29970* | NADH dehydrogenase I subunit F | 324 | 332 | 307 | 295 | 257 | 277 | 1 |
| 2440 | - | 2596162 | 2595662 | - | *nuoE* | *PA14_29980* | NADH dehydrogenase subunit E | 305 | 344 | 291 | 315 | 300 | 289 | 1 |
| 2441 | - | 2597945 | 2596164 | - | *nuoD* | *PA14_29990* | bifunctional NADH:ubiquinone oxidoreductase subunit C/D | 204 | 247 | 199 | 214 | 214 | 200 | 1 |
| 2442 | - | 2598703 | 2598026 | - | *nuoB* | *PA14_30010* | NADH dehydrogenase subunit B | 370 | 406 | 397 | 300 | 277 | 308 | 1 |
| 2443 | - | 2599127 | 2598714 | - | *nuoA* | *PA14_30020* | NADH dehydrogenase subunit A | 174 | 124 | 200 | 109 | 116 | 128 | 1 |
| 2444 | - | 2600281 | 2599727 | - | *-* | *PA14_30030* | hypothetical protein | 7 | 9 | 9 | 9 | 10 | 8 | 1 |
| 2445 | - | 2602378 | 2600360 | - | *-* | *PA14_30040* | hypothetical protein | 20 | 18 | 22 | 24 | 16 | 18 | 1 |
| 2446 | - | 2604153 | 2602558 | - | *-* | *PA14_30050* | isocitrate lyase | 145 | 163 | 105 | 60 | 101 | 74 | 1 |
| 2447 | - | 2605569 | 2604748 | - | *-* | *PA14_30070* | hypothetical protein | 91 | 99 | 114 | 106 | 109 | 123 | 1 |
| 2448 | - | 2606186 | 2605566 | - | *-* | *PA14_30080* | hypothetical protein | 62 | 64 | 78 | 62 | 64 | 68 | 1 |
| 2449 | - | 2606618 | 2606193 | - | *-* | *PA14_30090* | acetyl transferase | 285 | 275 | 325 | 245 | 235 | 280 | 1 |
| 2450 | - | 2607780 | 2606611 | - | *-* | *PA14_30100* | hypothetical protein | 113 | 99 | 149 | 64 | 58 | 95 | 1 |
| 2451 | - | 2609210 | 2607840 | - | *purB* | *PA14_30110* | adenylosuccinate lyase | 66 | 62 | 81 | 75 | 54 | 62 | 1 |
| 2452 | - | 2610176 | 2609286 | - | *-* | *PA14_30130* | hypothetical protein | 30 | 33 | 38 | 43 | 31 | 32 | 1 |
| 2453 | - | 2610799 | 2610179 | - | *-* | *PA14_30140* | hypothetical protein | 79 | 76 | 90 | 82 | 79 | 65 | 1 |
| 2454 | - | 2611923 | 2610796 | - | *mnmA* | *PA14_30150* | tRNA-specific 2-thiouridylase MnmA | 103 | 83 | 122 | 92 | 80 | 84 | 1 |
| 2455 | - | 2612437 | 2611967 | - | *-* | *PA14_30160* | hypothetical protein | 23 | 26 | 24 | 25 | 23 | 23 | 1 |
| 2456 | - | 2614749 | 2612524 | - | *idh* | *PA14_30180* | monomeric isocitrate dehydrogenase | 138 | 126 | 108 | 71 | 101 | 73 | 1 |
| 2457 | - | 2615108 | 2616364 | + | *icd* | *PA14_30190* | isocitrate dehydrogenase | 1036 | 1457 | 940 | 673 | 987 | 701 | 1 |
| 2458 | - | 2616710 | 2616438 | - | *cspD* | *PA14_30200* | cold-shock protein CspD | 339 | 395 | 381 | 415 | 329 | 330 | 1 |
| 2459 | - | 2616936 | 2617304 | + | *clpS* | *PA14_30210* | ATP-dependent Clp protease adaptor protein ClpS | 389 | 385 | 534 | 551 | 511 | 614 | 1 |
| 2460 | - | 2617305 | 2617319 | ? | *-* | predicted RNA | - | 671 | 611 | 817 | 1073 | 963 | 1145 | 1 |
| 2461 | - | 2617332 | 2619608 | + | *clpA* | *PA14_30230* | ATP-dependent Clp protease, ATP-binding subunit ClpA | 787 | 766 | 827 | 901 | 862 | 980 | 1 |
| 2462 | - | 2619908 | 2619690 | - | *infA* | *PA14_30240* | translation initiation factor IF-1 | 30 | 45 | 44 | 36 | 41 | 38 | 1 |
| 2463 | - | 2620720 | 2620013 | - | *-* | *PA14_30260* | arginyl-tRNA-protein transferase | 80 | 98 | 105 | 73 | 71 | 96 | 1 |
| 2464 | - | 2621455 | 2620775 | - | *aat* | *PA14_30270* | leucyl/phenylalanyl-tRNA--protein transferase | 26 | 28 | 27 | 23 | 21 | 23 | 1 |
| 2465 | - | 2622443 | 2621493 | - | *trxB1* | *PA14_30280* | thioredoxin reductase 1 | 99 | 127 | 93 | 85 | 89 | 78 | 1 |
| 2466 | - | 2622671 | 2625106 | + | *ftsK* | *PA14_30290* | cell division protein FtsK | 193 | 179 | 195 | 214 | 189 | 200 | 1 |
| 2467 | - | 2625132 | 2625758 | + | *lolA* | *PA14_30310* | outer-membrane lipoprotein carrier protein | 134 | 146 | 142 | 126 | 128 | 140 | 1 |
| 2468 | - | 2625768 | 2627093 | + | *-* | *PA14_30320* | recombination factor protein RarA | 202 | 176 | 198 | 178 | 189 | 194 | 1 |
| 2469 | - | 2627215 | 2628495 | + | *serS* | *PA14_30330* | seryl-tRNA synthetase | 148 | 153 | 138 | 119 | 128 | 135 | 1 |
| 2470 | - | 2628497 | 2629894 | + | *cysG* | *PA14_30340* | siroheme synthase | 247 | 224 | 230 | 214 | 226 | 226 | 1 |
| 2471 | - | 2630873 | 2629899 | - | *-* | *PA14_30350* | hypothetical protein | 40 | 43 | 41 | 46 | 38 | 41 | 1 |
| 2472 | - | 2631944 | 2630961 | - | *-* | *PA14_30360* | hypothetical protein | 76 | 77 | 76 | 76 | 64 | 75 | 1 |
| 2473 | - | 2632276 | 2631941 | - | *-* | *PA14_30370* | hypothetical protein | 71 | 71 | 95 | 61 | 63 | 68 | 1 |
| 2474 | - | 2632578 | 2632273 | - | *-* | *PA14_30380* | hypothetical protein | 62 | 53 | 72 | 44 | 53 | 52 | 1 |
| 2475 | - | 2632937 | 2632578 | - | *-* | *PA14_30390* | sulfur relay protein TusC | 123 | 110 | 134 | 102 | 106 | 100 | 1 |
| 2476 | - | 2633329 | 2632934 | - | *-* | *PA14_30400* | sulfur transfer complex subunit TusD | 111 | 99 | 123 | 86 | 104 | 100 | 1 |
| 2477 | - | 2634108 | 2633440 | - | *-* | *PA14_30410* | hypothetical protein | 259 | 289 | 267 | 149 | 194 | 191 | 1 |
| 2478 | - | 2634238 | 2634324 | + | *-* | *PA14_30420* | Ser tRNA | 37 | 26 | 31 | 25 | 15 | 9 | 1 |
| 2479 | - | 2636045 | 2634462 | - | *-* | *PA14_30430* | thiosulfate sulfurtransferase | 29 | 33 | 29 | 30 | 30 | 32 | 1 |
| 2480 | - | 2636647 | 2636042 | - | *-* | *PA14_30440* | hypothetical protein | 9 | 13 | 14 | 10 | 13 | 13 | 1 |
| 2481 | - | 2636760 | 2637656 | + | *-* | *PA14_30450* | LysR family transcriptional regulator | 31 | 31 | 30 | 30 | 33 | 34 | 1 |
| 2482 | - | 2637996 | 2639063 | + | *-* | *PA14_30460* | flavin-dependent oxidoreductase | 10 | 10 | 9 | 9 | 11 | 10 | 1 |
| 2483 | - | 2639073 | 2640017 | + | *-* | *PA14_30470* | periplasmic aliphatic sulfonate-binding protein | 10 | 12 | 11 | 11 | 10 | 10 | 1 |
| 2484 | - | 2640027 | 2641109 | + | *-* | *PA14_30490* | lavin-dependent oxidoreductase | 9 | 9 | 8 | 7 | 7 | 7 | 1 |
| 2485 | - | 2641114 | 2642265 | + | *-* | *PA14_30500* | acyl-CoA dehydrogenase | 18 | 17 | 17 | 20 | 16 | 16 | 1 |
| 2486 | - | 2642373 | 2643359 | + | *-* | *PA14_30520* | periplasmic aliphatic sulfonate-binding protein | 7 | 6 | 7 | 6 | 5 | 5 | 1 |
| 2487 | - | 2643371 | 2644327 | + | *-* | *PA14_30540* | periplasmic aliphatic sulfonate-binding protein | 13 | 8 | 8 | 10 | 9 | 8 | 0.678622091 |
| 2488 | - | 2644483 | 2645442 | + | *-* | *PA14_30550* | periplasmic aliphatic sulfonate-binding protein | 28 | 23 | 23 | 27 | 25 | 23 | 1 |
| 2489 | - | 2646081 | 2645509 | - | *-* | *PA14_30560* | hypothetical protein | 31 | 21 | 23 | 28 | 23 | 26 | 1 |
| 2490 | 2647472 | 2647394 | 2646291 | - | *-* | *PA14_30570* | periplasmic spermidine/putrescine-binding protein | 143 | 81 | 100 | 139 | 110 | 127 | 1 |
| 2491 | 2647472 | 2647547 | 2648353 | + | *-* | *PA14_30580* | LuxR family transcriptional regulator | 219 | 279 | 277 | 363 | 278 | 235 | 1 |
| 2492 | - | 2648473 | 2651127 | + | *-* | *PA14_30590* | outer membrane receptor protein | 11 | 10 | 14 | 12 | 9 | 9 | 1 |
| 2493 | - | 2651138 | 2652352 | + | *-* | *PA14_30600* | permease | 20 | 13 | 13 | 22 | 15 | 28 | 1 |
| 2494 | - | 2653396 | 2652368 | - | *-* | *PA14_30620* | AraC family transcriptional regulator | 165 | 88 | 100 | 147 | 108 | 217 | 1 |
| 2495 | - | 2654014 | 2655162 | + | *pqsH* | *PA14_30630* | FAD-dependent monooxygenase | 392 | 406 | 451 | 504 | 418 | 395 | 1 |
| 2496 | - | 2655504 | 2656148 | + | *gacA* | *PA14_30650* | response regulator GacA | 430 | 332 | 533 | 487 | 392 | 487 | 1 |
| 2497 | - | 2656149 | 2657975 | + | *uvrC* | *PA14_30660* | excinuclease ABC subunit C | 85 | 72 | 90 | 82 | 74 | 84 | 1 |
| 2498 | - | 2658009 | 2658569 | + | *pgsA* | *PA14_30670* | CDP-diacylglycerol--glycerol-3-phosphate 3-phosphatidyltransferase | 33 | 34 | 36 | 30 | 27 | 25 | 1 |
| 2499 | - | 2658638 | 2658710 | + | *-* | *PA14_30680* | Gly tRNA | 87 | 39 | 68 | 32 | 16 | 24 | 0.032235375 |
| 2500 | - | 2660507 | 2659452 | - | *-* | *PA14_30690* | hypothetical protein | 30 | 25 | 24 | 35 | 31 | 27 | 1 |
| 2501 | - | 2661144 | 2664122 | + | *-* | *PA14_30700* | sensor/response regulator hybrid | 74 | 69 | 77 | 74 | 78 | 73 | 1 |
| 2502 | - | 2664213 | 2664746 | + | *-* | *PA14_30710* | osmoprotectant transporter activator protein | 240 | 236 | 232 | 231 | 201 | 233 | 1 |
| 2503 | - | 2665032 | 2665102 | + | *-* | *PA14_30720* | Cys tRNA | 58 | 31 | 55 | 31 | 13 | 14 | 0.503413992 |
| 2504 | - | 2666538 | 2665498 | - | *-* | *PA14_30730* | hypothetical protein | 53 | 44 | 46 | 51 | 55 | 41 | 1 |
| 2505 | - | 2666962 | 2667552 | + | *-* | *PA14_30740* | NADPH specific quinone oxidoreductase | 6 | 4 | 6 | 5 | 5 | 4 | 0.940967938 |
| 2506 | - | 2667877 | 2668743 | + | *-* | *PA14_30750* | tryptophan oxygenase | 37 | 32 | 39 | 41 | 34 | 37 | 1 |
| 2507 | - | 2669335 | 2668775 | - | *-* | *PA14_30760* | acetyltransferase | 24 | 29 | 24 | 20 | 25 | 21 | 1 |
| 2508 | - | 2669787 | 2669350 | - | *-* | *PA14_30770* | AsnC family transcriptional regulator | 51 | 49 | 42 | 34 | 42 | 40 | 1 |
| 2509 | - | 2669920 | 2670819 | + | *-* | *PA14_30790* | hypothetical protein | 39 | 29 | 38 | 31 | 28 | 32 | 1 |
| 2510 | - | 2671487 | 2670885 | - | *-* | *PA14_30800* | hypothetical protein | 79 | 88 | 51 | 38 | 46 | 46 | 1 |
| 2511 | - | 2671834 | 2672982 | + | *alkB1* | *PA14_30810* | alkane-1 monooxygenase | 23 | 36 | 29 | 32 | 22 | 27 | 1 |
| 2512 | - | 2673094 | 2674701 | + | *-* | *PA14_30820* | methyl-accepting chemotaxis transducer | 173 | 164 | 215 | 223 | 161 | 227 | 1 |
| 2513 | - | 2676055 | 2674712 | - | *-* | *PA14_30830* | two-component response regulator | 123 | 139 | 162 | 150 | 141 | 174 | 1 |
| 2514 | 2677542 | 2676132 | 2677544 | + | *-* | *PA14_30840* | signal transduction histidine kinase | 159 | 127 | 180 | 195 | 134 | 210 | 1 |
| 2515 | - | 2677870 | 2678133 | ? | *-* | predicted RNA | - | 1087 | 686 | 773 | 842 | 882 | 817 | 1 |
| 2516 | - | 2679706 | 2678435 | - | *-* | *PA14_30850* | TrbI-like protein | 7 | 5 | 9 | 7 | 6 | 10 | 1 |
| 2517 | - | 2680698 | 2679709 | - | *-* | *PA14_30860* | TrbG-like protein | 4 | 4 | 4 | 4 | 4 | 3 | 1 |
| 2518 | - | 2681399 | 2680695 | - | *-* | *PA14_30870* | conjugal transfer protein TrbF | 11 | 17 | 12 | 18 | 13 | 16 | 1 |
| 2519 | - | 2682782 | 2681412 | - | *-* | *PA14_30880* | conjugal transfer protein TrbL | 4 | 4 | 4 | 5 | 3 | 4 | 1 |
| 2520 | - | 2682980 | 2683123 | + | *-* | *PA14_30890* | hypothetical protein | 11 | 9 | 7 | 8 | 8 | 11 | 1 |

|  | A | B | C | D | E | F | G | H | I | J | K | L | M | N |
| --- | --- | --- | --- | --- | --- | --- | --- | --- | --- | --- | --- | --- | --- | --- |
| 2521 | - | 2683836 | 2683111 | - | *-* | *PA14_30900* | conjugal transfer protein TrbJ | 5 | 4 | 4 | 4 | 4 | 4 | 1 |
| 2522 | - | 2686286 | 2683833 | - | *-* | *PA14_30910* | conjugal transfer ATPase TrbE | 4 | 5 | 3 | 4 | 3 | 4 | 1 |
| 2523 | - | 2686960 | 2686568 | - | *-* | *PA14_30930* | TrbC-like protein | 1 | 4 | 1 | 2 | 3 | 2 | 0.055881303 |
| 2524 | - | 2688027 | 2686957 | - | *-* | *PA14_30940* | conjugal transfer protein | 6 | 6 | 5 | 7 | 7 | 6 | 1 |
| 2525 | - | 2688488 | 2688024 | - | *-* | *PA14_30950* | hypothetical protein | 17 | 16 | 17 | 14 | 14 | 12 | 1 |
| 2526 | - | 2690485 | 2688485 | - | *-* | *PA14_30960* | conjugal transfer coupling protein TraG | 12 | 13 | 14 | 17 | 10 | 13 | 1 |
| 2527 | - | 2692000 | 2691005 | - | *-* | *PA14_30970* | transcriptional regulator | 42 | 44 | 46 | 36 | 41 | 41 | 1 |
| 2528 | - | 2692432 | 2692127 | - | *-* | *PA14_30980* | hypothetical protein | 165 | 181 | 162 | 169 | 138 | 134 | 1 |
| 2529 | - | 2693151 | 2692483 | - | *-* | *PA14_30990* | hypothetical protein | 149 | 139 | 132 | 141 | 131 | 121 | 1 |
| 2530 | - | 2693907 | 2693164 | - | *-* | *PA14_31000* | hypothetical protein | 67 | 65 | 63 | 60 | 72 | 68 | 1 |
| 2531 | - | 2697041 | 2693904 | - | *-* | *PA14_31010* | cation efflux system protein | 30 | 32 | 28 | 29 | 30 | 31 | 1 |
| 2532 | - | 2698281 | 2697052 | - | *-* | *PA14_31030* | cation efflux system protein | 34 | 37 | 32 | 32 | 30 | 32 | 1 |
| 2533 | - | 2699525 | 2698278 | - | *-* | *PA14_31040* | cation efflux system protein | 24 | 26 | 25 | 24 | 23 | 24 | 1 |
| 2534 | - | 2700664 | 2699666 | - | *-* | *PA14_31050* | hypothetical protein | 176 | 141 | 199 | 177 | 184 | 191 | 1 |
| 2535 | - | 2701278 | 2700661 | - | *-* | *PA14_31060* | hypothetical protein | 111 | 101 | 148 | 97 | 119 | 121 | 1 |
| 2536 | - | 2703530 | 2701170 | - | *-* | *PA14_31070* | hypothetical protein | 3 | 3 | 4 | 4 | 3 | 3 | 1 |
| 2537 | - | 2704179 | 2703604 | - | *-* | *PA14_31080* | conjugal transfer protein | 8 | 9 | 10 | 9 | 7 | 9 | 1 |
| 2538 | - | 2705093 | 2704176 | - | *-* | *PA14_31090* | hypothetical protein | 5 | 6 | 6 | 5 | 5 | 6 | 1 |
| 2539 | - | 2705649 | 2705011 | - | *-* | *PA14_31100* | plasmid partitioning protein | 5 | 4 | 5 | 4 | 2 | 4 | 1 |
| 2540 | - | 2707231 | 2705903 | - | *-* | *PA14_31110* | replication initiator and transcriptional repressor protein | 11 | 8 | 11 | 9 | 8 | 10 | 1 |
| 2541 | - | 2707950 | 2707180 | - | *-* | *PA14_31130* | hypothetical protein | 6 | 5 | 6 | 5 | 5 | 6 | 1 |
| 2542 | - | 2708695 | 2708294 | - | *-* | *PA14_31150* | hypothetical protein | 204 | 154 | 169 | 155 | 151 | 266 | 1 |
| 2543 | - | 2708860 | 2709183 | + | *-* | *PA14_31160* | hypothetical protein | 74 | 116 | 116 | 95 | 109 | 16 | 1 |
| 2544 | - | 2709230 | 2709337 | + | *-* | *PA14_31170* | hypothetical protein | 129 | 161 | 155 | 140 | 149 | 28 | 1 |
| 2545 | - | 2710638 | 2709577 | - | *-* | *PA14_31180* | hypothetical protein | 28 | 27 | 39 | 26 | 23 | 13 | 1 |
| 2546 | - | 2712999 | 2710945 | - | *-* | *PA14_31190* | hypothetical protein | 3 | 2 | 3 | 3 | 3 | 2 | 1 |
| 2547 | - | 2713905 | 2713081 | - | *-* | *PA14_31200* | hypothetical protein | 2 | 3 | 2 | 4 | 3 | 3 | 1 |
| 2548 | - | 2714903 | 2714625 | - | *-* | *PA14_31220* | hypothetical protein | 5 | 7 | 8 | 11 | 7 | 8 | 1 |
| 2549 | - | 2715496 | 2714969 | - | *-* | *PA14_31230* | hypothetical protein | 5 | 2 | 6 | 4 | 3 | 3 | 0.405144617 |
| 2550 | - | 2716943 | 2715633 | - | *-* | *PA14_31240* | nrbE-like protein | 89 | 73 | 85 | 63 | 82 | 84 | 1 |
| 2551 | - | 2717738 | 2717346 | - | *-* | *PA14_31250* | hypothetical protein | 8 | 4 | 5 | 4 | 3 | 5 | 0.434308967 |
| 2552 | - | 2718521 | 2718015 | - | *-* | *PA14_31260* | RadC-like protein | 1 | 1 | 1 | 1 | 1 | 1 | 1 |
| 2553 | - | 2720011 | 2718848 | - | *-* | *PA14_31270* | hypothetical protein | 15 | 14 | 16 | 16 | 11 | 14 | 1 |
| 2554 | - | 2721213 | 2720008 | - | *-* | *PA14_31280* | integrase | 20 | 21 | 19 | 19 | 18 | 19 | 1 |
| 2555 | 2721761 | 2721861 | 2722229 | + | *pa1L* | *PA14_31290* | PA-I galactophilic lectin | 71 | 12 | 11 | 42 | 27 | 161 | 1.60308E-29 |
| 2556 | - | 2722503 | 2722844 | + | *-* | *PA14_31300* | hypothetical protein | 56 | 48 | 50 | 58 | 47 | 63 | 1 |
| 2557 | - | 2722963 | 2723370 | + | *-* | *PA14_31310* | hypothetical protein | 172 | 149 | 169 | 244 | 174 | 186 | 1 |
| 2558 | - | 2724928 | 2723429 | - | *-* | *PA14_31330* | EAL domain-containing protein | 22 | 19 | 24 | 13 | 17 | 27 | 1 |
| 2559 | - | 2725301 | 2725657 | + | *-* | *PA14_31340* | hypothetical protein | 4 | 2 | 3 | 4 | 4 | 4 | 1 |
| 2560 | - | 2726001 | 2727038 | + | *-* | *PA14_31350* | hypothetical protein | 240 | 71 | 167 | 327 | 118 | 340 | 0.062668448 |
| 2561 | - | 2727061 | 2727486 | + | *-* | *PA14_31360* | hypothetical protein | 102 | 23 | 39 | 98 | 45 | 133 | 3.49395E-10 |
| 2562 | - | 2727476 | 2728303 | + | *-* | *PA14_31370* | hypothetical protein | 194 | 44 | 73 | 192 | 96 | 289 | 1.5759E-05 |
| 2563 | - | 2729800 | 2728313 | - | *-* | *PA14_31380* | sulfate transporter | 36 | 12 | 18 | 35 | 22 | 61 | 0.005762476 |
| 2564 | - | 2730150 | 2730662 | + | *-* | *PA14_31390* | hypothetical protein | 43 | 48 | 47 | 59 | 40 | 56 | 1 |
| 2565 | - | 2732504 | 2730777 | - | *-* | *PA14_31400* | chemotaxis transducer | 20 | 22 | 26 | 28 | 19 | 19 | 1 |
| 2566 | - | 2732652 | 2732939 | + | *-* | *PA14_31420* | hypothetical protein | 52 | 37 | 48 | 29 | 29 | 42 | 1 |
| 2567 | - | 2733244 | 2733014 | - | *-* | *PA14_31430* | hypothetical protein | 55 | 56 | 78 | 57 | 65 | 51 | 1 |
| 2568 | - | 2733563 | 2734105 | + | *-* | *PA14_31440* | hypothetical protein | 295 | 267 | 320 | 339 | 266 | 304 | 1 |
| 2569 | - | 2734291 | 2734145 | - | *-* | *PA14_31450* | hypothetical protein | 91 | 90 | 109 | 115 | 87 | 107 | 1 |
| 2570 | - | 2735120 | 2734428 | - | *-* | *PA14_31460* | transporter | 26 | 22 | 30 | 27 | 22 | 24 | 1 |
| 2571 | - | 2735341 | 2737035 | + | *-* | *PA14_31470* | AMP-binding protein | 94 | 145 | 106 | 141 | 111 | 124 | 1 |
| 2572 | - | 2737104 | 2738189 | + | *-* | *PA14_31480* | AraC family transcriptional regulator | 72 | 80 | 74 | 96 | 76 | 86 | 1 |
| 2573 | - | 2738366 | 2740033 | + | *-* | *PA14_31500* | AMP-binding protein | 291 | 345 | 282 | 418 | 384 | 449 | 1 |
| 2574 | - | 2740053 | 2740820 | + | *-* | *PA14_31510* | short-chain dehydrogenase | 441 | 476 | 373 | 586 | 516 | 589 | 1 |
| 2575 | - | 2740838 | 2742028 | + | *-* | *PA14_31530* | acyl-CoA thiolase | 397 | 550 | 377 | 524 | 541 | 553 | 1 |
| 2576 | - | 2742057 | 2743184 | + | *-* | *PA14_31540* | acyl-CoA dehydrogenase | 285 | 431 | 272 | 367 | 373 | 378 | 1 |
| 2577 | - | 2744233 | 2743301 | - | *-* | *PA14_31560* | LysR family transcriptional regulator | 83 | 95 | 90 | 98 | 101 | 99 | 1 |
| 2578 | - | 2744407 | 2745636 | + | *-* | *PA14_31580* | acyl-CoA dehydrogenase | 40 | 44 | 43 | 45 | 54 | 57 | 1 |
| 2579 | - | 2746870 | 2745827 | - | *-* | *PA14_31610* | TerC family protein | 26 | 20 | 31 | 20 | 21 | 17 | 1 |
| 2580 | - | 2748414 | 2747020 | - | *-* | *PA14_31620* | hypothetical protein | 7 | 6 | 8 | 8 | 7 | 5 | 1 |
| 2581 | - | 2749379 | 2748474 | - | *-* | *PA14_31630* | LysR family transcriptional regulator | 11 | 9 | 14 | 14 | 9 | 9 | 1 |
| 2582 | - | 2749483 | 2749914 | + | *-* | *PA14_31640* | ring-cleaving dioxygenase | 13 | 15 | 12 | 12 | 10 | 11 | 1 |
| 2583 | - | 2750833 | 2750021 | - | *xthA* | *PA14_31650* | exonuclease III | 32 | 36 | 34 | 33 | 35 | 29 | 1 |
| 2584 | - | 2750978 | 2751691 | + | *-* | *PA14_31660* | hypothetical protein | 96 | 89 | 103 | 141 | 98 | 108 | 1 |
| 2585 | - | 2751825 | 2753564 | + | *-* | *PA14_31680* | hypothetical protein | 46 | 39 | 45 | 46 | 40 | 40 | 1 |
| 2586 | - | 2753561 | 2757226 | + | *-* | *PA14_31690* | hypothetical protein | 45 | 34 | 37 | 39 | 38 | 39 | 1 |
| 2587 | - | 2757361 | 2757996 | + | *-* | *PA14_31700* | CDP-alcohol phosphatidyltransferase | 82 | 51 | 74 | 67 | 61 | 60 | 1 |
| 2588 | - | 2758086 | 2759846 | + | *-* | *PA14_31720* | hypothetical protein | 27 | 22 | 20 | 17 | 17 | 18 | 1 |
| 2589 | - | 2759882 | 2761195 | + | *-* | *PA14_31730* | hypothetical protein | 31 | 20 | 22 | 20 | 24 | 23 | 1 |
| 2590 | - | 2761188 | 2761640 | + | *-* | *PA14_31740* | hypothetical protein | 36 | 20 | 28 | 17 | 25 | 25 | 0.546512063 |
| 2591 | - | 2761652 | 2762281 | + | *-* | *PA14_31750* | acyltransferase | 20 | 14 | 14 | 10 | 16 | 12 | 1 |
| 2592 | - | 2762283 | 2763218 | + | *-* | *PA14_31760* | phosphatidate cytidylyltransferase | 26 | 21 | 20 | 17 | 24 | 24 | 1 |
| 2593 | - | 2764402 | 2763407 | - | *-* | *PA14_31770* | oxidoreductase | 11 | 11 | 11 | 9 | 9 | 9 | 1 |
| 2594 | - | 2764521 | 2765432 | + | *-* | *PA14_31780* | LysR family transcriptional regulator | 34 | 26 | 42 | 29 | 22 | 29 | 1 |
| 2595 | - | 2765588 | 2766937 | + | *-* | *PA14_31800* | sodium:alanine symporter | 46 | 59 | 47 | 47 | 48 | 38 | 1 |
| 2596 | - | 2767072 | 2767569 | + | *tpx* | *PA14_31810* | thiol peroxidase | 157 | 168 | 81 | 127 | 171 | 180 | 1 |
| 2597 | - | 2768843 | 2767719 | - | *-* | *PA14_31820* | aminotransferase | 6 | 6 | 5 | 4 | 5 | 4 | 1 |
| 2598 | - | 2770365 | 2769040 | - | *-* | *PA14_31840* | hypothetical protein | 47 | 45 | 36 | 44 | 40 | 43 | 1 |
| 2599 | - | 2771798 | 2770365 | - | *-* | *PA14_31850* | hypothetical protein | 45 | 41 | 46 | 51 | 40 | 42 | 1 |
| 2600 | - | 2772055 | 2773335 | + | *-* | *PA14_31870* | RND efflux membrane fusion protein | 35 | 33 | 36 | 37 | 25 | 25 | 1 |
| 2601 | - | 2773332 | 2776463 | + | *-* | *PA14_31890* | RND efflux transporter | 32 | 34 | 33 | 37 | 28 | 28 | 1 |
| 2602 | - | 2776460 | 2779570 | + | *-* | *PA14_31900* | efflux transporter | 41 | 37 | 40 | 50 | 38 | 41 | 1 |
| 2603 | - | 2779567 | 2781063 | + | *-* | *PA14_31920* | outer membrane protein | 46 | 58 | 45 | 60 | 50 | 47 | 1 |
| 2604 | - | 2781107 | 2781430 | + | *-* | *PA14_31930* | hypothetical protein | 23 | 21 | 16 | 20 | 19 | 15 | 1 |

|  | A | B | C | D | E | F | G | H | I | J | K | L | M | N |
| --- | --- | --- | --- | --- | --- | --- | --- | --- | --- | --- | --- | --- | --- | --- |
| 2605 | - | 2782857 | 2781439 | - | *-* | *PA14_31950* | two-component sensor | 19 | 19 | 19 | 19 | 17 | 16 | 1 |
| 2606 | - | 2783528 | 2782854 | - | *-* | *PA14_31960* | two-component response regulator | 12 | 15 | 11 | 11 | 11 | 10 | 1 |
| 2607 | - | 2784041 | 2785327 | + | *czcC* | *PA14_31970* | CzcC family cobalt/zinc/cadmium efflux transporter outer membrane protein | 1 | 1 | 1 | 1 | 0 | 1 | 1 |
| 2608 | - | 2785380 | 2786834 | + | *czcB* | *PA14_31990* | cobalt/zinc/cadmium efflux RND transporter, membrane fusion protein, CzcB famil | 5 | 3 | 3 | 3 | 2 | 2 | 1 |
| 2609 | - | 2790219 | 2791175 | + | *xylS* | *PA14_32060* | transcriptional regulator XylS | 18 | 15 | 16 | 16 | 13 | 16 | 1 |
| 2610 | - | 2791292 | 2792659 | + | *xylX* | *PA14_32080* | toluate 1,2-dioxygenase subunit alpha | 8 | 9 | 9 | 9 | 7 | 8 | 1 |
| 2611 | - | 2792656 | 2793144 | + | *xylY* | *PA14_32100* | toluate 1,2-dioxygenase subunit beta | 2 | 0 | 1 | 2 | 1 | 1 | 9.5179E-11 |
| 2612 | - | 2793178 | 2794191 | + | *xylZ* | *PA14_32110* | toluate 1,2-dioxygenase electron transfer component | 6 | 4 | 7 | 5 | 5 | 5 | 0.381163237 |
| 2613 | - | 2794217 | 2794978 | + | *benD* | *PA14_32130* | 1,6-dihydroxycyclohexa-2,4-diene-1-carboxylate dehydrogenase | 3 | 4 | 3 | 3 | 3 | 3 | 1 |
| 2614 | - | 2796006 | 2794984 | - | *antC* | *PA14_32140* | anthranilate dioxygenase reductase | 2 | 2 | 2 | 2 | 0 | 2 | 1 |
| 2615 | - | 2796516 | 2796025 | - | *antB* | *PA14_32150* | anthranilate dioxygenase small subunit | 9 | 7 | 8 | 9 | 6 | 6 | 1 |
| 2616 | - | 2797907 | 2796513 | - | *antA* | *PA14_32160* | anthranilate dioxygenase large subunit | 6 | 5 | 4 | 5 | 4 | 3 | 1 |
| 2617 | - | 2798224 | 2799225 | + | *-* | *PA14_32190* | transcriptional regulator | 24 | 18 | 19 | 18 | 16 | 23 | 1 |
| 2618 | - | 2800106 | 2799234 | - | *catR* | *PA14_32200* | transcriptional regulator CatR | 10 | 9 | 13 | 11 | 9 | 11 | 1 |
| 2619 | - | 2800268 | 2801389 | + | *catB* | *PA14_32220* | muconate cycloisomerase I | 4 | 3 | 3 | 3 | 3 | 2 | 0.831807123 |
| 2620 | - | 2801421 | 2801711 | + | *catC* | *PA14_32230* | muconolactone delta-isomerase | 4 | 5 | 5 | 5 | 4 | 3 | 1 |
| 2621 | - | 2801756 | 2802688 | + | *catA* | *PA14_32240* | catechol 1,2-dioxygenase | 3 | 5 | 4 | 4 | 6 | 4 | 1 |
| 2622 | - | 2803064 | 2802846 | - | *-* | *PA14_32250* | hypothetical protein | 4 | 3 | 5 | 4 | 5 | 5 | 1 |
| 2623 | - | 2803427 | 2804773 | + | *-* | *PA14_32270* | porin | 7 | 11 | 9 | 9 | 8 | 9 | 1 |
| 2624 | - | 2805025 | 2805642 | + | *-* | *PA14_32280* | hypothetical protein | 172 | 194 | 210 | 288 | 175 | 244 | 1 |
| 2625 | - | 2806858 | 2808357 | + | *-* | *PA14_32300* | kinase | 9 | 7 | 10 | 8 | 5 | 6 | 1 |
| 2626 | - | 2806899 | 2805646 | - | *-* | *PA14_32290* | hypothetical protein | 71 | 71 | 85 | 100 | 66 | 87 | 1 |
| 2627 | - | 2808461 | 2808628 | + | *-* | *PA14_32310* | hypothetical protein | 1720 | 2056 | 2466 | 1541 | 1824 | 2505 | 1 |
| 2628 | - | 2809898 | 2808672 | - | *-* | *PA14_32330* | cyanate permease | 173 | 182 | 235 | 120 | 145 | 195 | 1 |
| 2629 | - | 2810350 | 2809895 | - | *-* | *PA14_32340* | deaminase | 11 | 9 | 10 | 10 | 10 | 11 | 1 |
| 2630 | - | 2811000 | 2810368 | - | *-* | *PA14_32350* | hypothetical protein | 14 | 11 | 16 | 14 | 10 | 13 | 1 |
| 2631 | - | 2811128 | 2812006 | + | *-* | *PA14_32360* | lysR family transcriptional regulator | 23 | 24 | 25 | 21 | 24 | 23 | 1 |
| 2632 | - | 2812065 | 2812580 | + | *-* | *PA14_32370* | DNA damage-inducible gene | 9 | 14 | 11 | 12 | 10 | 10 | 1 |
| 2633 | - | 2814157 | 2812739 | - | *oprN* | *PA14_32380* | multidrug efflux outer membrane protein OprN precursor | 6 | 7 | 7 | 6 | 7 | 6 | 1 |
| 2634 | - | 2817342 | 2814154 | - | *mexF* | *PA14_32390* | RND multidrug efflux transporter MexF | 9 | 8 | 8 | 8 | 7 | 9 | 1 |
| 2635 | - | 2818608 | 2817364 | - | *mexE* | *PA14_32400* | RND multidrug efflux membrane fusion protein MexE | 4 | 4 | 5 | 4 | 5 | 4 | 1 |
| 2636 | - | 2819753 | 2818839 | - | *mexT* | *PA14_32410* | transcriptional regulator MexT | 48 | 35 | 41 | 42 | 40 | 41 | 1 |
| 2637 | - | 2819974 | 2820993 | + | *-* | *PA14_32420* | oxidoreductase | 149 | 117 | 140 | 123 | 104 | 120 | 1 |
| 2638 | - | 2821426 | 2821052 | - | *-* | *PA14_32440* | hypothetical protein | 42 | 38 | 44 | 34 | 37 | 32 | 1 |
| 2639 | - | 2822322 | 2821507 | - | *-* | *PA14_32450* | AraC family transcriptional regulator | 21 | 21 | 27 | 20 | 21 | 18 | 1 |
| 2640 | - | 2823116 | 2822349 | - | *-* | *PA14_32460* | transcriptional regulator | 23 | 19 | 25 | 22 | 24 | 21 | 1 |
| 2641 | - | 2823214 | 2823486 | + | *-* | *PA14_32470* | hypothetical protein | 11 | 15 | 14 | 14 | 14 | 13 | 1 |
| 2642 | - | 2823706 | 2823512 | - | *-* | *PA14_32480* | hypothetical protein | 59 | 75 | 64 | 61 | 77 | 60 | 1 |
| 2643 | - | 2824001 | 2823723 | - | *-* | *PA14_32490* | hypothetical protein | 55 | 42 | 65 | 52 | 70 | 55 | 1 |
| 2644 | - | 2824030 | 2824644 | + | *-* | *PA14_32500* | TetR family transcriptional regulator | 94 | 67 | 91 | 67 | 63 | 66 | 1 |
| 2645 | - | 2824795 | 2825796 | + | *-* | *PA14_32520* | hypothetical protein | 32 | 31 | 29 | 34 | 33 | 35 | 1 |
| 2646 | - | 2825911 | 2826564 | + | *-* | *PA14_32530* | cytochrome c | 103 | 92 | 99 | 61 | 77 | 95 | 1 |
| 2647 | - | 2826561 | 2827442 | + | *-* | *PA14_32540* | hypothetical protein | 118 | 109 | 114 | 80 | 101 | 119 | 1 |
| 2648 | - | 2828954 | 2827632 | - | *-* | *PA14_32570* | two-component sensor | 59 | 47 | 58 | 45 | 47 | 50 | 1 |
| 2649 | - | 2829631 | 2828951 | - | *-* | *PA14_32580* | two-component response regulator | 23 | 19 | 23 | 18 | 15 | 21 | 1 |
| 2650 | - | 2829794 | 2831563 | + | *-* | *PA14_32590* | thiol:disulfide interchange protein | 21 | 23 | 26 | 29 | 19 | 22 | 1 |
| 2651 | - | 2831563 | 2832399 | + | *-* | *PA14_32600* | thiol:disulfide interchange protein | 28 | 27 | 30 | 39 | 32 | 30 | 1 |
| 2652 | - | 2832396 | 2833166 | + | *dsbG* | *PA14_32610* | disulfide isomerase/thiol-disulfide oxidase | 50 | 64 | 58 | 70 | 64 | 60 | 1 |
| 2653 | - | 2833239 | 2834573 | + | *-* | *PA14_32630* | cytochrome P450 | 49 | 36 | 47 | 53 | 39 | 52 | 1 |
| 2654 | - | 2835466 | 2834555 | - | *-* | *PA14_32640* | hypothetical protein | 11 | 11 | 14 | 15 | 11 | 14 | 1 |
| 2655 | - | 2836152 | 2835508 | - | *-* | *PA14_32650* | glutathione S-transferase | 7 | 5 | 9 | 9 | 5 | 8 | 1 |
| 2656 | - | 2837511 | 2836165 | - | *-* | *PA14_32660* | MFS family transporter | 5 | 5 | 6 | 6 | 5 | 4 | 1 |
| 2657 | - | 2838287 | 2837589 | - | *-* | *PA14_32670* | hypothetical protein | 4 | 5 | 7 | 5 | 5 | 5 | 1 |
| 2658 | - | 2839361 | 2838300 | - | *gtdA* | *PA14_32690* | gentisate 1,2-dioxygenase | 6 | 5 | 4 | 4 | 5 | 3 | 1 |
| 2659 | - | 2839486 | 2840400 | + | *-* | *PA14_32700* | transcriptional regulator | 10 | 10 | 10 | 9 | 10 | 8 | 1 |
| 2660 | - | 2840489 | 2841007 | + | *-* | *PA14_32710* | ECF subfamily RNA polymerase sigma-70 factor | 17 | 80 | 26 | 139 | 91 | 29 | 1.78038E-09 |
| 2661 | - | 2841016 | 2842002 | + | *-* | *PA14_32720* | transmembrane sensor | 13 | 26 | 19 | 52 | 32 | 18 | 0.41668339 |
| 2662 | - | 2842146 | 2844608 | + | *-* | *PA14_32740* | TonB-dependent receptor | 8 | 9 | 8 | 12 | 10 | 7 | 1 |
| 2663 | - | 2844726 | 2845874 | + | *-* | *PA14_32750* | hypothetical protein | 3 | 2 | 3 | 3 | 2 | 3 | 1 |
| 2664 | - | 2845921 | 2846445 | + | *-* | *PA14_32770* | hypothetical protein | 34 | 26 | 33 | 29 | 24 | 26 | 1 |
| 2665 | - | 2846758 | 2848455 | + | *-* | *PA14_32780* | hypothetical protein | 34 | 30 | 39 | 35 | 31 | 28 | 1 |
| 2666 | - | 2848568 | 2864206 | + | *-* | *PA14_32790* | hypothetical protein | 46 | 39 | 43 | 33 | 43 | 31 | 1 |
| 2667 | - | 2865179 | 2865490 | + | *-* | *PA14_32810* | hypothetical protein | 10 | 11 | 9 | 10 | 11 | 9 | 1 |
| 2668 | - | 2868300 | 2868887 | + | *-* | *PA14_32820* | hypothetical protein | 16 | 10 | 11 | 10 | 10 | 8 | 0.569926259 |
| 2669 | - | 2873250 | 2870581 | - | *-* | *PA14_32830* | hypothetical protein | 37 | 41 | 34 | 47 | 43 | 37 | 1 |
| 2670 | - | 2874218 | 2873265 | - | *-* | *PA14_32840* | hypothetical protein | 39 | 38 | 32 | 38 | 42 | 41 | 1 |
| 2671 | - | 2876003 | 2874987 | - | *-* | *PA14_32850* | hypothetical protein | 40 | 38 | 43 | 57 | 45 | 41 | 1 |
| 2672 | - | 2877069 | 2876533 | - | *-* | *PA14_32860* | hypothetical protein | 27 | 29 | 31 | 30 | 31 | 28 | 1 |
| 2673 | - | 2877917 | 2877066 | - | *-* | *PA14_32880* | hypothetical protein | 17 | 15 | 16 | 14 | 15 | 13 | 1 |
| 2674 | - | 2878192 | 2877971 | - | *-* | *PA14_32890* | hypothetical protein | 37 | 66 | 54 | 43 | 40 | 25 | 0.831807123 |
| 2675 | - | 2878349 | 2879929 | + | *-* | *PA14_32905* | hypothetical protein | 9 | 8 | 10 | 11 | 8 | 10 | 1 |
| 2676 | - | 2880132 | 2881049 | + | *-* | *PA14_32930* | hypothetical protein | 15 | 13 | 13 | 13 | 10 | 13 | 1 |
| 2677 | - | 2881131 | 2882666 | + | *-* | *PA14_32940* | transcriptional regulator | 42 | 29 | 33 | 41 | 31 | 47 | 1 |
| 2678 | - | 2884416 | 2882674 | - | *-* | *PA14_32950* | hypothetical protein | 50 | 19 | 25 | 36 | 27 | 61 | 0.137571381 |
| 2679 | - | 2884539 | 2885462 | + | *-* | *PA14_32970* | transcriptional regulator | 7 | 8 | 7 | 10 | 7 | 6 | 1 |
| 2680 | - | 2885721 | 2886104 | + | *gcvH2* | *PA14_32985* | glycine cleavage system protein H | 106 | 173 | 154 | 182 | 169 | 150 | 0.885197681 |
| 2681 | - | 2886115 | 2888994 | + | *gcvP2* | *PA14_33000* | glycine dehydrogenase | 119 | 263 | 147 | 258 | 230 | 245 | 1 |
| 2682 | - | 2889137 | 2890393 | + | *glyA2* | *PA14_33010* | serine hydroxymethyltransferase | 93 | 192 | 132 | 243 | 193 | 223 | 1 |
| 2683 | - | 2890441 | 2891817 | + | *sdaA* | *PA14_33030* | L-serine dehydratase | 124 | 200 | 183 | 244 | 220 | 244 | 1 |
| 2684 | - | 2891911 | 2893032 | + | *gcvT2* | *PA14_33040* | glycine cleavage system protein T2 | 181 | 308 | 256 | 398 | 364 | 382 | 1 |
| 2685 | - | 2893954 | 2893079 | - | *-* | *PA14_33050* | hypothetical protein | 79 | 133 | 97 | 172 | 160 | 151 | 1 |
| 2686 | - | 2894922 | 2893951 | - | *-* | *PA14_33060* | hypothetical protein | 16 | 15 | 15 | 16 | 17 | 18 | 1 |
| 2687 | - | 2895611 | 2897611 | + | *-* | *PA14_33070* | hypothetical protein | 19 | 14 | 22 | 20 | 17 | 17 | 1 |
| 2688 | - | 2897623 | 2898636 | + | *-* | *PA14_33080* | hypothetical protein | 22 | 19 | 21 | 23 | 21 | 21 | 1 |

|  | A | B | C | D | E | F | G | H | I | J | K | L | M | N |
| --- | --- | --- | --- | --- | --- | --- | --- | --- | --- | --- | --- | --- | --- | --- |
| 2689 | - | 2898633 | 2899673 | + | *-* | *PA14_33110* | hypothetical protein | 19 | 15 | 17 | 19 | 19 | 19 | 1 |
| 2690 | - | 2899827 | 2900261 | + | *-* | *PA14_33120* | hypothetical protein | 100 | 105 | 113 | 111 | 105 | 91 | 1 |
| 2691 | - | 2900290 | 2902275 | + | *-* | *PA14_33130* | cation-transporting P-type ATPase | 72 | 71 | 95 | 89 | 75 | 87 | 1 |
| 2692 | - | 2902272 | 2902817 | + | *-* | *PA14_33150* | hypothetical protein | 85 | 70 | 86 | 90 | 78 | 83 | 1 |
| 2693 | - | 2903221 | 2902952 | - | *-* | *PA14_33160* | hypothetical protein | 67 | 70 | 67 | 81 | 75 | 84 | 1 |
| 2694 | - | 2904250 | 2903336 | - | *-* | *PA14_33170* | transcriptional regulator | 11 | 13 | 8 | 12 | 12 | 11 | 1 |
| 2695 | - | 2904651 | 2906825 | + | *-* | *PA14_33190* | hypothetical protein | 13 | 12 | 17 | 19 | 15 | 16 | 1 |
| 2696 | - | 2906815 | 2907024 | + | *-* | *PA14_33200* | hypothetical protein | 6 | 10 | 10 | 12 | 10 | 12 | 1 |
| 2697 | - | 2907021 | 2908025 | + | *-* | *PA14_33220* | hypothetical protein | 15 | 21 | 19 | 26 | 19 | 21 | 1 |
| 2698 | - | 2908307 | 2908062 | - | *-* | *PA14_33230* | hypothetical protein | 12 | 18 | 13 | 16 | 14 | 22 | 1 |
| 2699 | - | 2908569 | 2909483 | + | *-* | *PA14_33240* | hypothetical protein | 4 | 4 | 4 | 4 | 4 | 3 | 1 |
| 2700 | - | 2909570 | 2910037 | + | *-* | *PA14_33250* | hypothetical protein | 2 | 3 | 2 | 14 | 3 | 3 | 1 |
| 2701 | - | 2910617 | 2910054 | - | *pvdS* | *PA14_33260* | extracytoplasmic-function sigma-70 factor | 3 | 10 | 4 | 32 | 9 | 5 | 0.006435902 |
| 2702 | - | 2911261 | 2912025 | + | *pvdG* | *PA14_33270* | protein PvdG | 1 | 0 | 1 | 2 | 0 | 0 | 1 |
| 2703 | - | 2912099 | 2925127 | + | *pvdL* | *PA14_33280* | peptide synthase | 4 | 4 | 5 | 5 | 4 | 4 | 1 |
| 2704 | - | 2926368 | 2925613 | - | *-* | *PA14_33290* | hypothetical protein | 59 | 62 | 71 | 83 | 59 | 74 | 1 |
| 2705 | - | 2928020 | 2927517 | - | *-* | *PA14_33300* | hypothetical protein | 184 | 240 | 157 | 129 | 183 | 146 | 1 |
| 2706 | - | 2929112 | 2928084 | - | *-* | *PA14_33310* | hypothetical protein | 209 | 271 | 180 | 138 | 194 | 166 | 1 |
| 2707 | - | 2930106 | 2929123 | - | *-* | *PA14_33320* | hypothetical protein | 189 | 221 | 183 | 164 | 185 | 176 | 1 |
| 2708 | - | 2931397 | 2930093 | - | *-* | *PA14_33330* | hypothetical protein | 148 | 171 | 146 | 115 | 134 | 141 | 1 |
| 2709 | - | 2934685 | 2931455 | - | *-* | *PA14_33340* | helicase | 74 | 68 | 65 | 75 | 65 | 66 | 1 |
| 2710 | - | 2935656 | 2934682 | - | *-* | *PA14_33350* | hypothetical protein | 95 | 60 | 77 | 82 | 79 | 62 | 1 |
| 2711 | - | 2935897 | 2937291 | + | *-* | *PA14_33360* | hypothetical protein | 30 | 17 | 27 | 36 | 17 | 19 | 1 |
| 2712 | - | 2937790 | 2937476 | - | *-* | *PA14_33370* | hypothetical protein | 7 | 8 | 5 | 7 | 7 | 10 | 1 |
| 2713 | - | 2939415 | 2938495 | - | *-* | *PA14_33380* | hypothetical protein | 3 | 3 | 4 | 4 | 2 | 3 | 1 |
| 2714 | - | 2940867 | 2939449 | - | *-* | *PA14_33410* | porin | 4 | 5 | 6 | 5 | 3 | 4 | 1 |
| 2715 | - | 2941629 | 2940949 | - | *-* | *PA14_33420* | hydrolase | 10 | 14 | 10 | 11 | 10 | 10 | 1 |
| 2716 | - | 2942587 | 2941727 | - | *-* | *PA14_33430* | pirin-related protein | 2 | 3 | 3 | 3 | 2 | 2 | 1 |
| 2717 | - | 2942688 | 2943626 | + | *-* | *PA14_33440* | LysR family transcriptional regulator | 46 | 45 | 52 | 48 | 46 | 51 | 1 |
| 2718 | - | 2945267 | 2943630 | - | *treA* | *PA14_33450* | trehalase | 18 | 22 | 21 | 32 | 22 | 28 | 1 |
| 2719 | - | 2945626 | 2946051 | + | *-* | *PA14_33460* | hypothetical protein | 23 | 25 | 27 | 39 | 27 | 32 | 1 |
| 2720 | - | 2946044 | 2947363 | + | *sndH* | *PA14_33480* | L-sorbosone dehydrogenase | 48 | 61 | 72 | 111 | 63 | 87 | 1 |
| 2721 | - | 2947542 | 2948951 | + | *pvdH* | *PA14_33500* | diaminobutyrate--2-oxoglutarate aminotransferase | 8 | 12 | 12 | 22 | 13 | 16 | 1 |
| 2722 | - | 2949029 | 2949247 | + | *-* | *PA14_33510* | hypothetical protein | 3 | 6 | 5 | 12 | 4 | 6 | 0.711763148 |
| 2723 | - | 2949248 | 2950012 | + | *-* | *PA14_33520* | thioesterase | 10 | 9 | 12 | 22 | 11 | 13 | 1 |
| 2724 | - | 2951029 | 2950112 | - | *-* | *PA14_33530* | hypothetical protein | 6 | 9 | 6 | 7 | 7 | 6 | 1 |
| 2725 | - | 2951931 | 2951026 | - | *-* | *PA14_33540* | ABC transporter permease | 3 | 4 | 3 | 6 | 6 | 3 | 1 |
| 2726 | - | 2952683 | 2951928 | - | *-* | *PA14_33550* | ABC transporter ATP-binding protein | 7 | 9 | 8 | 9 | 8 | 5 | 1 |
| 2727 | - | 2953633 | 2952680 | - | *-* | *PA14_33560* | adhesion protein | 6 | 7 | 6 | 7 | 5 | 4 | 1 |
| 2728 | - | 2954226 | 2953666 | - | *-* | *PA14_33570* | hypothetical protein | 7 | 8 | 10 | 13 | 10 | 9 | 1 |
| 2729 | - | 2954552 | 2954223 | - | *-* | *PA14_33580* | hypothetical protein | 13 | 15 | 14 | 22 | 18 | 20 | 1 |
| 2730 | - | 2955088 | 2954549 | - | *-* | *PA14_33590* | hypothetical protein | 8 | 13 | 9 | 19 | 13 | 11 | 1 |
| 2731 | - | 2956296 | 2955085 | - | *-* | *PA14_33600* | hypothetical protein | 13 | 13 | 15 | 15 | 14 | 13 | 1 |
| 2732 | - | 2956615 | 2972064 | + | *-* | *PA14_33610* | peptide synthase | 13 | 11 | 14 | 15 | 11 | 13 | 1 |
| 2733 | - | 2972068 | 2978637 | + | *pvdJ* | *PA14_33630* | protein PvdJ | 24 | 20 | 25 | 24 | 20 | 23 | 1 |
| 2734 | - | 2978649 | 2985995 | + | *pvdD* | *PA14_33650* | pyoverdine synthetase D | 27 | 25 | 30 | 32 | 24 | 28 | 1 |
| 2735 | - | 2988606 | 2986159 | - | *fpvA* | *PA14_33680* | ferripyoverdine receptor | 19 | 25 | 20 | 39 | 30 | 22 | 1 |
| 2736 | - | 2990358 | 2988709 | - | *pvdE* | *PA14_33690* | pyoverdine biosynthesis protein PvdE | 34 | 37 | 39 | 44 | 33 | 41 | 1 |
| 2737 | - | 2990736 | 2991563 | + | *pvdF* | *PA14_33700* | pyoverdine synthetase F | 18 | 18 | 17 | 20 | 14 | 16 | 1 |
| 2738 | - | 2992486 | 2991632 | - | *pvdO* | *PA14_33710* | protein PvdO | 4 | 5 | 3 | 4 | 4 | 3 | 1 |
| 2739 | - | 2993798 | 2992515 | - | *pvdN* | *PA14_33720* | protein PvdN | 3 | 4 | 4 | 5 | 3 | 4 | 1 |
| 2740 | - | 2995167 | 2993821 | - | *-* | *PA14_33730* | dipeptidase | 3 | 5 | 5 | 5 | 3 | 4 | 1 |
| 2741 | - | 2995383 | 2997017 | + | *pvdP* | *PA14_33740* | protein PvdP | 4 | 5 | 5 | 4 | 4 | 4 | 1 |
| 2742 | - | 2998490 | 2997066 | - | *-* | *PA14_33750* | outer membrane protein | 9 | 7 | 9 | 7 | 7 | 7 | 1 |
| 2743 | - | 3000487 | 2998496 | - | *-* | *PA14_33760* | ABC transporter ATP-binding protein/permease | 11 | 11 | 12 | 15 | 14 | 11 | 1 |
| 2744 | - | 3001662 | 3000487 | - | *-* | *PA14_33770* | hypothetical protein | 8 | 6 | 9 | 10 | 10 | 9 | 1 |
| 2745 | - | 3002758 | 3001763 | - | *-* | *PA14_33780* | transmembrane sensor | 77 | 108 | 82 | 153 | 154 | 116 | 1 |
| 2746 | - | 3002922 | 3003401 | + | *-* | *PA14_33800* | RNA polymerase sigma factor | 30 | 38 | 43 | 58 | 64 | 44 | 1 |
| 2747 | - | 3003534 | 3004865 | + | *pvdA* | *PA14_33810* | L-ornithine N5-oxygenase | 7 | 8 | 9 | 16 | 8 | 9 | 1 |
| 2748 | - | 3004988 | 3007276 | + | *pvdQ* | *PA14_33820* | penicillin acylase-related protein | 4 | 4 | 4 | 6 | 4 | 4 | 1 |
| 2749 | - | 3007457 | 3007780 | + | *-* | *PA14_33830* | hypothetical protein | 1 | 6 | 4 | 11 | 5 | 4 | 7.29817E-07 |
| 2750 | - | 3007822 | 3008742 | + | *-* | *PA14_33840* | transcriptional regulator | 16 | 16 | 21 | 18 | 16 | 15 | 1 |
| 2751 | - | 3008839 | 3009990 | + | *lldA* | *PA14_33860* | L-lactate dehydrogenase | 31 | 32 | 29 | 36 | 28 | 27 | 1 |
| 2752 | - | 3010299 | 3010057 | - | *-* | *PA14_33870* | hypothetical protein | 260 | 352 | 190 | 312 | 269 | 330 | 1 |
| 2753 | - | 3010594 | 3010830 | + | *-* | *PA14_33880* | hypothetical protein | 28 | 34 | 34 | 41 | 29 | 33 | 1 |
| 2754 | - | 3011096 | 3011566 | + | *-* | *PA14_33890* | oxidoreductase | 45 | 46 | 46 | 51 | 40 | 42 | 1 |
| 2755 | - | 3011563 | 3013878 | + | *-* | *PA14_33900* | aldehyde dehydrogenase | 71 | 77 | 72 | 85 | 72 | 81 | 1 |
| 2756 | - | 3015569 | 3014295 | - | *-* | *PA14_33910* | hypothetical protein | 8 | 10 | 9 | 9 | 9 | 9 | 1 |
| 2757 | - | 3016342 | 3015701 | - | *-* | *PA14_33920* | transcriptional regulator | 5 | 4 | 5 | 5 | 5 | 5 | 1 |
| 2758 | - | 3016619 | 3017014 | + | *-* | *PA14_33930* | hypothetical protein | 46 | 51 | 66 | 74 | 52 | 58 | 1 |
| 2759 | - | 3017573 | 3017037 | - | *-* | *PA14_33940* | hypothetical protein | 33 | 34 | 38 | 40 | 32 | 43 | 1 |
| 2760 | - | 3019590 | 3017584 | - | *-* | *PA14_33960* | hypothetical protein | 16 | 15 | 20 | 25 | 17 | 25 | 1 |
| 2761 | - | 3020694 | 3019627 | - | *-* | *PA14_33970* | hypothetical protein | 39 | 35 | 49 | 46 | 32 | 52 | 1 |
| 2762 | - | 3021266 | 3020733 | - | *-* | *PA14_33980* | hypothetical protein | 60 | 48 | 69 | 71 | 51 | 87 | 1 |
| 2763 | - | 3023889 | 3021340 | - | *-* | *PA14_33990* | ClpA/B-type protease | 38 | 27 | 47 | 51 | 31 | 52 | 1 |
| 2764 | - | 3024907 | 3023891 | - | *-* | *PA14_34000* | hypothetical protein | 25 | 19 | 30 | 32 | 20 | 32 | 1 |
| 2765 | - | 3026664 | 3024871 | - | *-* | *PA14_34010* | hypothetical protein | 22 | 16 | 24 | 29 | 17 | 30 | 1 |
| 2766 | - | 3027073 | 3026648 | - | *-* | *PA14_34020* | hypothetical protein | 30 | 24 | 31 | 36 | 23 | 39 | 1 |
| 2767 | - | 3027583 | 3027086 | - | *-* | *PA14_34030* | hypothetical protein | 53 | 40 | 45 | 57 | 39 | 55 | 1 |
| 2768 | - | 3029141 | 3027657 | - | *-* | *PA14_34050* | hypothetical protein | 42 | 35 | 42 | 47 | 28 | 41 | 1 |
| 2769 | - | 3029709 | 3029164 | - | *-* | *PA14_34070* | hypothetical protein | 25 | 26 | 38 | 32 | 18 | 23 | 1 |
| 2770 | - | 3029917 | 3030393 | + | *-* | *PA14_34080* | hypothetical protein | 106 | 122 | 174 | 145 | 104 | 113 | 1 |
| 2771 | - | 3030453 | 3031784 | + | *-* | *PA14_34100* | hypothetical protein | 142 | 130 | 181 | 199 | 129 | 164 | 1 |
| 2772 | - | 3031802 | 3032560 | + | *-* | *PA14_34110* | hypothetical protein | 53 | 39 | 52 | 49 | 40 | 50 | 1 |

|  | A | B | C | D | E | F | G | H | I | J | K | L | M | N |
| --- | --- | --- | --- | --- | --- | --- | --- | --- | --- | --- | --- | --- | --- | --- |
| 2773 | - | 3032557 | 3036372 | + | *-* | *PA14_34130* | hypothetical protein | 44 | 34 | 54 | 56 | 41 | 55 | 1 |
| 2774 | - | 3036369 | 3037469 | + | *-* | *PA14_34140* | hypothetical protein | 55 | 54 | 61 | 68 | 54 | 79 | 1 |
| 2775 | - | 3037579 | 3038664 | + | *-* | *PA14_34150* | transcriptional regulator | 12 | 23 | 12 | 11 | 14 | 11 | 0.415011252 |
| 2776 | - | 3038777 | 3039166 | + | *-* | *PA14_34170* | hypothetical protein | 3 | 4 | 4 | 4 | 4 | 3 | 1 |
| 2777 | - | 3039326 | 3039886 | + | *msuE* | *PA14_34180* | NADH-dependent FMN reductase MsuE | 1 | 1 | 1 | 1 | 1 | 1 | 1 |
| 2778 | - | 3039896 | 3041041 | + | *msuD* | *PA14_34190* | methanesulfonate sulfonatase MsuD | 2 | 2 | 2 | 3 | 2 | 2 | 1 |
| 2779 | - | 3041071 | 3042255 | + | *-* | *PA14_34200* | FMNH2-dependent monooxygenase | 5 | 5 | 8 | 7 | 5 | 6 | 1 |
| 2780 | - | 3042252 | 3043382 | + | *-* | *PA14_34210* | transcriptional regulator | 3 | 3 | 3 | 3 | 2 | 3 | 1 |
| 2781 | - | 3043554 | 3044708 | + | *-* | *PA14_34230* | hypothetical protein | 16 | 18 | 16 | 17 | 15 | 14 | 1 |
| 2782 | - | 3045901 | 3044774 | - | *-* | *PA14_34250* | glycerophosphoryl diester phosphodiesterase | 7 | 9 | 7 | 8 | 8 | 6 | 1 |
| 2783 | - | 3046732 | 3046079 | - | *-* | *PA14_34260* | ABC transporter permease | 6 | 5 | 7 | 6 | 5 | 6 | 1 |
| 2784 | - | 3047825 | 3046716 | - | *-* | *PA14_34270* | ABC transporter ATP-binding protein | 3 | 3 | 3 | 4 | 3 | 3 | 1 |
| 2785 | - | 3048616 | 3047822 | - | *-* | *PA14_34280* | hypothetical protein | 3 | 3 | 5 | 4 | 3 | 3 | 1 |
| 2786 | - | 3050036 | 3048648 | - | *-* | *PA14_34290* | DszA family monooxygenase | 3 | 2 | 3 | 2 | 2 | 3 | 1 |
| 2787 | - | 3051271 | 3050054 | - | *-* | *PA14_34300* | DszC family monooxygenase | 7 | 7 | 9 | 9 | 9 | 7 | 1 |
| 2788 | - | 3052517 | 3051282 | - | *-* | *PA14_34320* | DszC family monooxygenase | 6 | 6 | 7 | 6 | 4 | 5 | 1 |
| 2789 | - | 3052936 | 3054171 | + | *-* | *PA14_34330* | hypothetical protein | 78 | 97 | 96 | 117 | 95 | 105 | 1 |
| 2790 | - | 3055134 | 3054202 | - | *mtlZ* | *PA14_34340* | fructokinase | 48 | 50 | 61 | 72 | 64 | 65 | 1 |
| 2791 | - | 3056684 | 3055176 | - | *mtlY* | *PA14_34350* | xylulose kinase | 4 | 4 | 5 | 5 | 3 | 4 | 1 |
| 2792 | - | 3058156 | 3056681 | - | *mtlD* | *PA14_34360* | mannitol dehydrogenase | 5 | 5 | 5 | 5 | 4 | 5 | 1 |
| 2793 | - | 3059291 | 3058179 | - | *-* | *PA14_34370* | ABC maltose/mannitol transporter ATP-binding protein | 11 | 8 | 12 | 11 | 7 | 9 | 1 |
| 2794 | - | 3060164 | 3059331 | - | *-* | *PA14_34390* | binding-protein-dependent maltose/mannitol transport protein | 6 | 6 | 7 | 6 | 6 | 6 | 1 |
| 2795 | - | 3061107 | 3060175 | - | *-* | *PA14_34410* | binding-protein-dependent maltose/mannitol transport protein | 11 | 12 | 13 | 11 | 9 | 12 | 1 |
| 2796 | - | 3062493 | 3061183 | - | *-* | *PA14_34420* | maltose/mannitol ABC transporter substrate-binding protein | 8 | 8 | 9 | 11 | 8 | 6 | 1 |
| 2797 | - | 3063556 | 3062651 | - | *mtlR* | *PA14_34440* | transcriptional regulator MtlR | 23 | 20 | 29 | 28 | 21 | 23 | 1 |
| 2798 | - | 3064684 | 3063779 | - | *-* | *PA14_34450* | AraC family transcriptional regulator | 24 | 27 | 21 | 21 | 25 | 18 | 1 |
| 2799 | - | 3064789 | 3065349 | + | *-* | *PA14_34460* | hypothetical protein | 99 | 37 | 41 | 71 | 56 | 67 | 0.027358573 |
| 2800 | - | 3065399 | 3066466 | + | *-* | *PA14_34490* | hypothetical protein | 186 | 51 | 86 | 117 | 116 | 118 | 0.012682198 |
| 2801 | - | 3066463 | 3067299 | + | *-* | *PA14_34500* | ABC transporter ATP-binding protein | 101 | 28 | 48 | 69 | 66 | 68 | 0.000612412 |
| 2802 | - | 3067265 | 3068464 | + | *-* | *PA14_34510* | hypothetical protein | 61 | 19 | 31 | 47 | 39 | 45 | 0.006979836 |
| 2803 | - | 3068461 | 3069225 | + | *-* | *PA14_34520* | ABC transporter permease | 57 | 27 | 39 | 60 | 52 | 51 | 0.317658547 |
| 2804 | - | 3069451 | 3070860 | + | *-* | *PA14_34540* | xenobiotic compound DszA family monooxygenase | 22 | 8 | 13 | 18 | 17 | 20 | 0.015343712 |
| 2805 | - | 3072155 | 3070920 | - | *-* | *PA14_34550* | hypothetical protein | 14 | 8 | 13 | 13 | 12 | 11 | 0.679564673 |
| 2806 | - | 3073445 | 3072186 | - | *-* | *PA14_34580* | hypothetical protein | 3 | 4 | 5 | 5 | 4 | 4 | 1 |
| 2807 | - | 3075288 | 3073663 | - | *-* | *PA14_34600* | glyceraldehyde-3-phosphate dehydrogenase | 13 | 77 | 78 | 36 | 93 | 57 | 4.36376E-14 |
| 2808 | - | 3075596 | 3075453 | - | *-* | *PA14_34610* | hypothetical protein | 3 | 10 | 6 | 7 | 8 | 6 | 0.000532638 |
| 2809 | - | 3076974 | 3075622 | - | *gnuT* | *PA14_34630* | gluconate permease | 16 | 19 | 20 | 21 | 16 | 18 | 1 |
| 2810 | - | 3077592 | 3077071 | - | *-* | *PA14_34640* | gluconokinase | 12 | 19 | 16 | 17 | 19 | 13 | 1 |
| 2811 | - | 3077787 | 3078818 | + | *gntR* | *PA14_34660* | transcriptional regulator GntR | 47 | 47 | 49 | 47 | 43 | 42 | 1 |
| 2812 | - | 3079537 | 3079172 | - | *-* | *PA14_34670* | cupin superfamily protein | 52 | 57 | 53 | 67 | 52 | 51 | 1 |
| 2813 | - | 3080868 | 3079570 | - | *-* | *PA14_34680* | oxidoreductase | 22 | 26 | 27 | 31 | 29 | 23 | 1 |
| 2814 | - | 3081865 | 3080972 | - | *-* | *PA14_34690* | LysR family transcriptional regulator | 19 | 11 | 14 | 11 | 11 | 9 | 0.8413024 |
| 2815 | - | 3081983 | 3083158 | + | *-* | *PA14_34700* | beta lactamase | 2 | 2 | 2 | 2 | 2 | 2 | 1 |
| 2816 | - | 3083158 | 3084411 | + | *-* | *PA14_34710* | major facilitator transporter | 8 | 7 | 9 | 8 | 7 | 6 | 1 |
| 2817 | - | 3085272 | 3084472 | - | *-* | *PA14_34720* | hypothetical protein | 28 | 28 | 37 | 31 | 22 | 22 | 1 |
| 2818 | - | 3085870 | 3085289 | - | *-* | *PA14_34730* | XRE family transcriptional regulator | 8 | 20 | 9 | 7 | 10 | 8 | 0.015737353 |
| 2819 | - | 3086083 | 3085925 | - | *-* | *PA14_34740* | hypothetical protein | 10 | 28 | 8 | 11 | 16 | 9 | 0.013429913 |
| 2820 | - | 3086373 | 3087260 | + | *-* | *PA14_34750* | taurine catabolism dioxygenase | 6 | 5 | 5 | 6 | 3 | 5 | 1 |
| 2821 | - | 3087301 | 3088323 | + | *-* | *PA14_34770* | ABC transporter substrate-binding protein | 6 | 7 | 5 | 6 | 6 | 5 | 1 |
| 2822 | - | 3088331 | 3089179 | + | *-* | *PA14_34780* | ABC transporter ATP-binding protein | 2 | 4 | 4 | 4 | 2 | 1 | 0.723542393 |
| 2823 | - | 3089196 | 3090062 | + | *-* | *PA14_34790* | ABC transporter permease | 7 | 8 | 8 | 10 | 6 | 9 | 1 |
| 2824 | - | 3090172 | 3090789 | + | *-* | *PA14_34800* | amino acid transporter LysE | 57 | 43 | 58 | 42 | 44 | 47 | 1 |
| 2825 | - | 3090885 | 3094634 | + | *-* | *PA14_34810* | non-ribosomal peptide synthetase | 214 | 176 | 192 | 213 | 165 | 188 | 1 |
| 2826 | - | 3094663 | 3095751 | + | *-* | *PA14_34820* | regulatory protein | 167 | 145 | 131 | 143 | 125 | 168 | 1 |
| 2827 | - | 3095748 | 3096767 | + | *-* | *PA14_34830* | regulatory protein | 178 | 136 | 144 | 151 | 145 | 201 | 1 |
| 2828 | - | 3096787 | 3103164 | + | *-* | *PA14_34840* | non-ribosomal peptide synthetase | 335 | 230 | 300 | 278 | 272 | 347 | 1 |
| 2829 | - | 3103232 | 3103774 | + | *-* | *PA14_34850* | tRNA synthase | 126 | 99 | 125 | 113 | 127 | 140 | 1 |
| 2830 | - | 3103993 | 3105444 | + | *chiC* | *PA14_34870* | chitinase | 320 | 16 | 26 | 83 | 62 | 326 | 7.9991E-209 |
| 2831 | - | 3105621 | 3106370 | + | *-* | *PA14_34880* | GntR family transcriptional regulator | 36 | 14 | 17 | 19 | 19 | 31 | 0.01418108 |
| 2832 | - | 3106367 | 3108091 | + | *-* | *PA14_34900* | oxidoreductase | 31 | 17 | 15 | 19 | 21 | 35 | 0.950259107 |
| 2833 | - | 3108177 | 3108422 | + | *-* | *PA14_34920* | ferredoxin | 18 | 7 | 6 | 15 | 10 | 20 | 0.033575691 |
| 2834 | - | 3108760 | 3109725 | + | *-* | *PA14_34930* | phycobiliprotein | 5 | 2 | 3 | 2 | 4 | 5 | 0.065349589 |
| 2835 | - | 3109722 | 3110003 | + | *-* | *PA14_34940* | hypothetical protein | 6 | 5 | 6 | 6 | 6 | 6 | 1 |
| 2836 | - | 3110344 | 3111702 | + | *-* | *PA14_34960* | glucose-sensitive porin | 70 | 544 | 315 | 440 | 452 | 407 | 7.79018E-18 |
| 2837 | - | 3111856 | 3114267 | + | *gcd* | *PA14_34970* | glucose dehydrogenase | 62 | 78 | 63 | 80 | 83 | 103 | 1 |
| 2838 | - | 3114399 | 3116519 | + | *-* | *PA14_34990* | TonB-dependent receptor | 27 | 35 | 36 | 47 | 43 | 56 | 1 |
| 2839 | - | 3116640 | 3116927 | + | *-* | *PA14_35000* | hypothetical protein | 67 | 77 | 65 | 92 | 80 | 101 | 1 |
| 2840 | - | 3117575 | 3116940 | - | *-* | *PA14_35010* | hypothetical protein | 31 | 34 | 32 | 37 | 40 | 39 | 1 |
| 2841 | - | 3119106 | 3117562 | - | *-* | *PA14_35020* | hypothetical protein | 9 | 9 | 10 | 11 | 10 | 10 | 1 |
| 2842 | - | 3119769 | 3119107 | - | *-* | *PA14_35030* | hypothetical protein | 5 | 6 | 6 | 7 | 3 | 7 | 1 |
| 2843 | - | 3120437 | 3119766 | - | *-* | *PA14_35040* | hypothetical protein | 16 | 11 | 15 | 15 | 10 | 13 | 0.934705131 |
| 2844 | - | 3121891 | 3120434 | - | *-* | *PA14_35050* | hypothetical protein | 2 | 2 | 2 | 3 | 2 | 2 | 1 |
| 2845 | - | 3122326 | 3121898 | - | *-* | *PA14_35060* | hypothetical protein | 18 | 19 | 20 | 16 | 14 | 16 | 1 |
| 2846 | - | 3122614 | 3123468 | + | *-* | *PA14_35070* | AraC family transcriptional regulator | 26 | 27 | 32 | 28 | 25 | 24 | 1 |
| 2847 | - | 3124173 | 3123481 | - | *-* | *PA14_35080* | arsenical resistance protein | 16 | 19 | 17 | 15 | 17 | 15 | 1 |
| 2848 | - | 3124655 | 3124185 | - | *arsC* | *PA14_35100* | arsenate reductase | 7 | 13 | 8 | 9 | 9 | 10 | 0.679564673 |
| 2849 | - | 3125970 | 3124687 | - | *arsB* | *PA14_35110* | arsenite-antimonite efflux pump ArsB | 8 | 8 | 9 | 9 | 8 | 7 | 1 |
| 2850 | - | 3126334 | 3125984 | - | *arsR* | *PA14_35130* | arsenic resistance transcriptional regulator | 8 | 7 | 8 | 8 | 9 | 9 | 1 |
| 2851 | - | 3127303 | 3126413 | - | *-* | *PA14_35140* | AraC family transcriptional regulator | 8 | 7 | 9 | 9 | 6 | 7 | 1 |
| 2852 | - | 3127512 | 3128573 | + | *-* | *PA14_35150* | alcohol dehydrogenase | 35 | 11 | 12 | 13 | 10 | 47 | 0.000776048 |
| 2853 | - | 3128975 | 3128598 | - | *-* | *PA14_35160* | hypothetical protein | 114 | 10 | 7 | 8 | 5 | 116 | 1.2809E-136 |
| 2854 | - | 3129053 | 3129523 | + | *-* | *PA14_35170* | redox-sensing activator of soxS | 90 | 70 | 61 | 74 | 62 | 135 | 1 |
| 2855 | - | 3131228 | 3129531 | - | *pbpC* | *PA14_35190* | penicillin-binding protein 3A | 50 | 45 | 43 | 55 | 39 | 64 | 1 |
| 2856 | - | 3131865 | 3131350 | - | *-* | *PA14_35200* | acetyltransferase | 30 | 41 | 33 | 32 | 29 | 31 | 1 |

|  | A | B | C | D | E | F | G | H | I | J | K | L | M | N |
| --- | --- | --- | --- | --- | --- | --- | --- | --- | --- | --- | --- | --- | --- | --- |
| 2857 | - | 3132463 | 3131873 | - | *-* | *PA14_35210* | TetR family transcriptional regulator | 24 | 25 | 23 | 21 | 19 | 20 | 1 |
| 2858 | - | 3132610 | 3133815 | + | *-* | *PA14_35230* | hypothetical protein | 17 | 18 | 19 | 18 | 15 | 14 | 1 |
| 2859 | - | 3134849 | 3133779 | - | *-* | *PA14_35240* | hypothetical protein | 15 | 11 | 17 | 13 | 12 | 15 | 1 |
| 2860 | - | 3134936 | 3135832 | + | *-* | *PA14_35250* | LysR family transcriptional regulator | 38 | 45 | 40 | 44 | 37 | 41 | 1 |
| 2861 | - | 3137251 | 3135932 | - | *-* | *PA14_35270* | cytochrome c precursor | 48 | 62 | 57 | 63 | 64 | 73 | 1 |
| 2862 | - | 3139038 | 3137263 | - | *gnd* | *PA14_35290* | gluconate dehydrogenase | 30 | 49 | 44 | 45 | 48 | 50 | 0.940967938 |
| 2863 | - | 3139757 | 3139041 | - | *-* | *PA14_35300* | hypothetical protein | 33 | 47 | 48 | 42 | 51 | 51 | 1 |
| 2864 | - | 3140911 | 3139925 | - | *-* | *PA14_35320* | 2-hydroxyacid dehydrogenase | 4 | 8 | 5 | 7 | 8 | 7 | 0.381163237 |
| 2865 | - | 3142237 | 3140930 | - | *-* | *PA14_35330* | 2-ketogluconate transporter | 4 | 6 | 7 | 7 | 7 | 5 | 1 |
| 2866 | - | 3143250 | 3142300 | - | *-* | *PA14_35340* | 2-ketogluconate kinase | 16 | 23 | 31 | 31 | 25 | 21 | 1 |
| 2867 | - | 3144025 | 3143243 | - | *-* | *PA14_35360* | hypothetical protein | 8 | 20 | 17 | 17 | 19 | 10 | 0.049721315 |
| 2868 | - | 3145119 | 3144097 | - | *ptxS* | *PA14_35370* | transcriptional regulator PtxS | 14 | 31 | 25 | 27 | 29 | 19 | 0.173484736 |
| 2869 | - | 3145681 | 3146619 | + | *ptxR* | *PA14_35380* | transcriptional regulator PtxR | 10 | 16 | 15 | 10 | 9 | 10 | 1 |
| 2870 | - | 3147369 | 3146722 | - | *pvcD* | *PA14_35390* | pyoverdine biosynthesis protein PvcD | 5 | 6 | 7 | 6 | 4 | 4 | 1 |
| 2871 | - | 3148864 | 3147362 | - | *pvcC* | *PA14_35400* | pyoverdine biosynthesis protein PvcC | 4 | 4 | 5 | 4 | 4 | 3 | 1 |
| 2872 | - | 3149791 | 3148916 | - | *pvcB* | *PA14_35420* | pyoverdine biosynthesis protein PvcB | 6 | 5 | 4 | 6 | 6 | 5 | 1 |
| 2873 | - | 3150795 | 3149809 | - | *pvcA* | *PA14_35430* | pyoverdine biosynthesis protein PvcA | 3 | 3 | 3 | 3 | 3 | 4 | 1 |
| 2874 | - | 3151997 | 3151011 | - | *ansA* | *PA14_35440* | L-asparaginase I | 17 | 14 | 13 | 12 | 8 | 10 | 1 |
| 2875 | - | 3153542 | 3152097 | - | *-* | *PA14_35460* | AGCS sodium/alanine/glycine symporter | 6 | 5 | 6 | 6 | 5 | 6 | 1 |
| 2876 | - | 3153711 | 3154529 | + | *-* | *PA14_35470* | hypothetical protein | 19 | 23 | 19 | 19 | 19 | 18 | 1 |
| 2877 | - | 3156056 | 3154662 | - | *lpdV* | *PA14_35490* | dihydrolipoamide dehydrogenase | 645 | 966 | 801 | 979 | 869 | 1119 | 1 |
| 2878 | - | 3157346 | 3156060 | - | *bkdB* | *PA14_35500* | branched-chain alpha-keto acid dehydrogenase subunit E2 | 499 | 778 | 581 | 671 | 597 | 761 | 1 |
| 2879 | - | 3158399 | 3157347 | - | *bkdA2* | *PA14_35520* | 2-oxoisovalerate dehydrogenase subunit beta | 451 | 693 | 474 | 542 | 513 | 620 | 1 |
| 2880 | - | 3159628 | 3158396 | - | *bkdA1* | *PA14_35530* | 2-oxoisovalerate dehydrogenase subunit alpha | 323 | 534 | 313 | 320 | 363 | 394 | 1 |
| 2881 | - | 3159938 | 3160399 | + | *bkdR* | *PA14_35540* | transcriptional regulator BkdR | 71 | 90 | 100 | 96 | 83 | 92 | 1 |
| 2882 | - | 3160737 | 3160432 | - | *pslO* | *PA14_35550* | hypothetical protein | 45 | 56 | 45 | 48 | 45 | 44 | 1 |
| 2883 | - | 3161766 | 3160765 | - | *pslN* | *PA14_35570* | hypothetical protein | 46 | 44 | 54 | 50 | 50 | 49 | 1 |
| 2884 | - | 3163555 | 3161822 | - | *pslM* | *PA14_35590* | FAD-binding dehydrogenase | 4 | 4 | 4 | 5 | 4 | 4 | 1 |
| 2885 | - | 3164810 | 3163743 | - | *pslL* | *PA14_35600* | hypothetical protein | 9 | 9 | 10 | 9 | 10 | 12 | 1 |
| 2886 | - | 3166302 | 3164893 | - | *pslK* | *PA14_35620* | hypothetical protein | 6 | 5 | 6 | 8 | 6 | 6 | 1 |
| 2887 | - | 3167740 | 3166304 | - | *pslJ* | *PA14_35630* | hypothetical protein | 6 | 6 | 5 | 8 | 6 | 8 | 1 |
| 2888 | - | 3168846 | 3167743 | - | *pslI* | *PA14_35640* | transferase | 8 | 8 | 9 | 11 | 9 | 14 | 1 |
| 2889 | - | 3170045 | 3168837 | - | *pslH* | *PA14_35650* | hypothetical protein | 10 | 9 | 10 | 14 | 11 | 15 | 1 |
| 2890 | - | 3171382 | 3170054 | - | *pslG* | *PA14_35670* | glycosyl hydrolase | 7 | 6 | 7 | 8 | 9 | 11 | 1 |
| 2891 | - | 3172559 | 3171372 | - | *pslF* | *PA14_35680* | hypothetical protein | 5 | 4 | 3 | 5 | 4 | 4 | 1 |
| 2892 | - | 3174547 | 3172559 | - | *pslE* | *PA14_35690* | hypothetical protein | 8 | 6 | 7 | 10 | 6 | 7 | 1 |
| 2893 | - | 3174603 | 3175307 | + | *-* | *PA14_35700* | hypothetical protein | 32 | 28 | 26 | 26 | 29 | 30 | 1 |
| 2894 | - | 3176147 | 3175803 | - | *-* | *PA14_35710* | hypothetical protein | 50 | 39 | 47 | 66 | 62 | 58 | 1 |
| 2895 | - | 3176615 | 3176214 | - | *-* | *PA14_35720* | hypothetical protein | 31 | 31 | 27 | 24 | 23 | 26 | 1 |
| 2896 | - | 3177358 | 3176984 | - | *-* | *PA14_35730* | hypothetical protein | 8 | 5 | 9 | 7 | 6 | 6 | 1 |
| 2897 | - | 3177507 | 3180521 | + | *-* | *PA14_35740* | transposase | 33 | 33 | 29 | 50 | 29 | 35 | 1 |
| 2898 | - | 3180518 | 3180880 | + | *-* | *PA14_35750* | tpnA repressor protein | 133 | 111 | 107 | 145 | 103 | 123 | 1 |
| 2899 | - | 3181524 | 3181060 | - | *-* | *PA14_35760* | hypothetical protein | 298 | 202 | 205 | 236 | 245 | 256 | 1 |
| 2900 | - | 3182437 | 3181532 | - | *-* | *PA14_35770* | hypothetical protein | 72 | 36 | 47 | 49 | 58 | 55 | 0.670565447 |
| 2901 | - | 3183162 | 3182434 | - | *-* | *PA14_35780* | hypothetical protein | 64 | 51 | 40 | 30 | 41 | 34 | 1 |
| 2902 | - | 3184587 | 3183178 | - | *-* | *PA14_35790* | homospermidine synthase | 108 | 64 | 66 | 60 | 82 | 74 | 1 |
| 2903 | - | 3186315 | 3184999 | - | *-* | *PA14_35800* | hypothetical protein | 113 | 106 | 98 | 93 | 96 | 103 | 1 |
| 2904 | - | 3187047 | 3186337 | - | *-* | *PA14_35810* | hypothetical protein | 333 | 357 | 315 | 337 | 306 | 322 | 1 |
| 2905 | 3188223 | 3188081 | 3187110 | - | *tnpS* | *PA14_35820* | cointegrate resolution protein S | 244 | 195 | 256 | 246 | 238 | 245 | 1 |
| 2906 | 3188223 | 3188265 | 3189263 | + | *tnpT* | *PA14_35830* | cointegrate resolution protein T | 108 | 107 | 89 | 91 | 101 | 96 | 1 |
| 2907 | 3188223 | 3189302 | 3189946 | + | *-* | *PA14_35840* | hypothetical protein | 257 | 221 | 211 | 248 | 262 | 280 | 1 |
| 2908 | 3188223 | 3189927 | 3190406 | + | *-* | *PA14_35850* | hypothetical protein | 359 | 351 | 312 | 401 | 380 | 406 | 1 |
| 2909 | - | 3190988 | 3192292 | + | *-* | *PA14_35860* | amino acid permease | 11 | 11 | 12 | 11 | 9 | 11 | 1 |
| 2910 | - | 3192371 | 3193795 | + | *-* | *PA14_35880* | gamma-aminobutyraldehyde dehydrogenase | 20 | 14 | 18 | 21 | 16 | 22 | 1 |
| 2911 | - | 3193841 | 3195094 | + | *-* | *PA14_35890* | diaminobutyrate--2-oxoglutarate aminotransferase | 12 | 10 | 11 | 10 | 11 | 14 | 1 |
| 2912 | - | 3195183 | 3196103 | + | *-* | *PA14_35900* | dehydrogenase | 12 | 12 | 13 | 16 | 12 | 15 | 1 |
| 2913 | - | 3198054 | 3196396 | - | *-* | *PA14_35920* | acetate permease | 13 | 12 | 15 | 17 | 10 | 10 | 1 |
| 2914 | - | 3198149 | 3198051 | - | *-* | *PA14_35930* | hypothetical protein | 7 | 11 | 12 | 12 | 7 | 9 | 1 |
| 2915 | - | 3200078 | 3198432 | - | *-* | *PA14_35940* | acyl-CoA synthetase | 9 | 9 | 10 | 11 | 7 | 9 | 1 |
| 2916 | - | 3200932 | 3200165 | - | *-* | *PA14_35950* | dehydrogenase | 4 | 6 | 6 | 6 | 7 | 5 | 1 |
| 2917 | - | 3202104 | 3200932 | - | *-* | *PA14_35970* | acyl-CoA dehydrogenase | 3 | 4 | 4 | 4 | 3 | 3 | 1 |
| 2918 | - | 3202511 | 3202155 | - | *-* | *PA14_35980* | acyl-CoA dehydrogenase | 2 | 4 | 8 | 7 | 5 | 6 | 0.793216635 |
| 2919 | - | 3203704 | 3202508 | - | *-* | *PA14_35990* | FadE36, aminoglycoside phosphotransferase | 3 | 3 | 4 | 3 | 2 | 4 | 1 |
| 2920 | - | 3204291 | 3205712 | + | *prpR* | *PA14_36000* | propionate catabolism operon regulator | 43 | 39 | 36 | 36 | 38 | 38 | 1 |
| 2921 | - | 3206281 | 3205778 | - | *-* | *PA14_36010* | hypothetical protein | 29 | 27 | 25 | 25 | 27 | 28 | 1 |
| 2922 | - | 3208643 | 3206340 | - | *-* | *PA14_36020* | paraquat-inducible protein B | 38 | 35 | 31 | 39 | 32 | 33 | 1 |
| 2923 | - | 3209256 | 3208636 | - | *-* | *PA14_36030* | paraquat-inducible protein A | 19 | 18 | 19 | 20 | 16 | 20 | 1 |
| 2924 | - | 3211497 | 3209914 | - | *-* | *PA14_36050* | aldehyde dehydrogenase | 3 | 4 | 3 | 3 | 3 | 3 | 1 |
| 2925 | - | 3212621 | 3211626 | - | *-* | *PA14_36060* | hypothetical protein | 6 | 6 | 6 | 6 | 6 | 6 | 1 |
| 2926 | - | 3213824 | 3212649 | - | *-* | *PA14_36070* | hypothetical protein | 5 | 5 | 4 | 5 | 4 | 4 | 1 |
| 2927 | - | 3215177 | 3213855 | - | *-* | *PA14_36080* | MFS transporter | 1 | 1 | 2 | 1 | 1 | 1 | 1 |
| 2928 | - | 3216613 | 3215363 | - | *-* | *PA14_36090* | porin | 2 | 2 | 2 | 2 | 1 | 2 | 1 |
| 2929 | - | 3217799 | 3216786 | - | *pdxA* | *PA14_36100* | 4-hydroxythreonine-4-phosphate dehydrogenase | 10 | 10 | 10 | 10 | 8 | 8 | 1 |
| 2930 | - | 3218755 | 3217796 | - | *-* | *PA14_36110* | hypothetical protein | 6 | 8 | 6 | 6 | 6 | 6 | 1 |
| 2931 | - | 3220073 | 3218748 | - | *-* | *PA14_36120* | MFS transporter | 3 | 3 | 4 | 3 | 3 | 4 | 1 |
| 2932 | - | 3220256 | 3221353 | + | *-* | *PA14_36130* | hypothetical protein | 2 | 3 | 5 | 5 | 2 | 3 | 1 |
| 2933 | - | 3221381 | 3221911 | + | *-* | *PA14_36150* | hypothetical protein | 1 | 1 | 2 | 2 | 1 | 1 | 1 |
| 2934 | - | 3221908 | 3223428 | + | *-* | *PA14_36170* | hypothetical protein | 2 | 2 | 2 | 3 | 2 | 2 | 1 |
| 2935 | - | 3223496 | 3224443 | + | *-* | *PA14_36180* | LysR family transcriptional regulator | 30 | 29 | 35 | 32 | 30 | 31 | 1 |
| 2936 | - | 3224525 | 3225001 | + | *-* | *PA14_36190* | hypothetical protein | 19 | 18 | 19 | 17 | 17 | 17 | 1 |
| 2937 | - | 3225300 | 3226106 | + | *-* | *PA14_36200* | ABC transporter substrate-binding protein | 20 | 42 | 10 | 12 | 21 | 10 | 0.225704119 |
| 2938 | - | 3226187 | 3226903 | + | *-* | *PA14_36220* | amino acid permease | 3 | 4 | 3 | 2 | 3 | 2 | 1 |
| 2939 | - | 3226905 | 3227582 | + | *-* | *PA14_36230* | amino acid ABC transporter permease | 13 | 16 | 13 | 12 | 15 | 11 | 1 |
| 2940 | - | 3228482 | 3227598 | - | *-* | *PA14_36250* | hypothetical protein | 19 | 25 | 22 | 20 | 18 | 14 | 1 |

|  | A | B | C | D | E | F | G | H | I | J | K | L | M | N |
| --- | --- | --- | --- | --- | --- | --- | --- | --- | --- | --- | --- | --- | --- | --- |
| 2941 | - | 3230203 | 3228608 | - | *-* | *PA14_36260* | signal transduction protein | 14 | 15 | 15 | 18 | 14 | 12 | 1 |
| 2942 | - | 3231167 | 3230292 | - | *-* | *PA14_36270* | dehydrogenase | 52 | 61 | 64 | 47 | 37 | 37 | 1 |
| 2943 | - | 3231517 | 3231173 | - | *-* | *PA14_36280* | antibiotic biosynthesis monooxygenase | 13 | 26 | 28 | 14 | 16 | 10 | 0.339012339 |
| 2944 | - | 3232551 | 3231514 | - | *-* | *PA14_36290* | hypothetical protein | 22 | 38 | 32 | 26 | 24 | 17 | 0.723542393 |
| 2945 | - | 3233266 | 3232682 | - | *-* | *PA14_36300* | TetR family transcriptional regulator | 104 | 30 | 68 | 46 | 40 | 85 | 0.00024672 |
| 2946 | 3236436 | 3234672 | 3233419 | - | *hcnC* | *PA14_36310* | hydrogen cyanide synthase HcnC | 498 | 60 | 187 | 132 | 172 | 391 | 7.58162E-17 |
| 2947 | 3236436 | 3236069 | 3234675 | - | *hcnB* | *PA14_36320* | hydrogen cyanide synthase HcnB | 226 | 28 | 87 | 69 | 121 | 167 | 1.89869E-23 |
| 2948 | 3236436 | 3236380 | 3236066 | - | *hcnA* | *PA14_36330* | hydrogen cyanide synthase HcnA | 214 | 20 | 87 | 58 | 70 | 141 | 6.8676E-100 |
| 2949 | 3236436 | 3236744 | 3237988 | + | *exoY* | *PA14_36345* | adenylate cyclase | 64 | 45 | 74 | 85 | 50 | 71 | 1 |
| 2950 | - | 3238539 | 3237985 | - | *-* | *PA14_36350* | hypothetical protein | 69 | 57 | 80 | 100 | 66 | 86 | 1 |
| 2951 | - | 3239118 | 3238696 | - | *-* | *PA14_36360* | hypothetical protein | 52 | 46 | 64 | 78 | 53 | 61 | 1 |
| 2952 | - | 3240360 | 3239146 | - | *-* | *PA14_36370* | carboxylate-amine ligase | 25 | 28 | 39 | 40 | 34 | 33 | 1 |
| 2953 | - | 3240498 | 3241922 | + | *-* | *PA14_36375* | hypothetical protein | 7 | 10 | 10 | 11 | 9 | 10 | 1 |
| 2954 | - | 3241906 | 3242856 | + | *-* | *PA14_36390* | hypothetical protein | 12 | 15 | 17 | 17 | 14 | 14 | 1 |
| 2955 | - | 3243160 | 3242840 | - | *-* | *PA14_36400* | hypothetical protein | 7 | 10 | 12 | 14 | 13 | 11 | 1 |
| 2956 | - | 3243223 | 3243831 | + | *-* | *PA14_36410* | hypothetical protein | 18 | 27 | 25 | 33 | 22 | 33 | 1 |
| 2957 | - | 3245934 | 3243835 | - | *-* | *PA14_36420* | sensor/response regulator hybrid | 30 | 33 | 34 | 44 | 35 | 43 | 1 |
| 2958 | - | 3246017 | 3246607 | + | *-* | *PA14_36450* | hypothetical protein | 86 | 89 | 103 | 68 | 87 | 75 | 1 |
| 2959 | - | 3246620 | 3246967 | + | *-* | *PA14_36460* | hypothetical protein | 53 | 60 | 70 | 59 | 77 | 70 | 1 |
| 2960 | - | 3247252 | 3247560 | + | *-* | *PA14_36470* | hypothetical protein | 56 | 81 | 106 | 67 | 73 | 89 | 1 |
| 2961 | - | 3247812 | 3247591 | - | *-* | *PA14_36480* | hypothetical protein | 10 | 19 | 16 | 22 | 15 | 21 | 0.860626969 |
| 2962 | - | 3248218 | 3247868 | - | *-* | *PA14_36490* | hypothetical protein | 27 | 42 | 38 | 56 | 36 | 40 | 1 |
| 2963 | - | 3249321 | 3248245 | - | *-* | *PA14_36500* | hypothetical protein | 40 | 64 | 68 | 79 | 57 | 56 | 0.934705131 |
| 2964 | - | 3249795 | 3249325 | - | *-* | *PA14_36520* | hypothetical protein | 46 | 73 | 72 | 91 | 75 | 71 | 0.970587038 |
| 2965 | - | 3250449 | 3249997 | - | *-* | *PA14_36530* | hypothetical protein | 26 | 53 | 48 | 57 | 36 | 40 | 0.317658547 |
| 2966 | - | 3251351 | 3250575 | - | *-* | *PA14_36540* | hypothetical protein | 16 | 14 | 15 | 24 | 16 | 18 | 1 |
| 2967 | - | 3252445 | 3251348 | - | *-* | *PA14_36550* | hypothetical protein | 29 | 26 | 34 | 40 | 29 | 32 | 1 |
| 2968 | - | 3253139 | 3252774 | - | *-* | *PA14_36560* | hypothetical protein | 96 | 87 | 65 | 93 | 63 | 71 | 1 |
| 2969 | - | 3253507 | 3255048 | + | *glgA* | *PA14_36570* | glycogen synthase | 38 | 53 | 57 | 64 | 45 | 50 | 1 |
| 2970 | - | 3255048 | 3256799 | + | *-* | *PA14_36580* | glycosyl hydrolase | 27 | 43 | 48 | 64 | 49 | 47 | 0.955702824 |
| 2971 | - | 3256792 | 3258846 | + | *-* | *PA14_36590* | 4-alpha-glucanotransferase | 21 | 36 | 36 | 50 | 43 | 39 | 0.831807123 |
| 2972 | - | 3258839 | 3261619 | + | *-* | *PA14_36605* | maltooligosyl trehalose synthase | 23 | 49 | 39 | 64 | 51 | 52 | 0.436346238 |
| 2973 | - | 3261616 | 3261921 | + | *-* | *PA14_36620* | hypothetical protein | 9 | 29 | 23 | 36 | 31 | 29 | 0.000287258 |
| 2974 | - | 3261934 | 3264084 | + | *-* | *PA14_36630* | glycosyl hydrolase | 21 | 50 | 34 | 60 | 48 | 48 | 0.139802753 |
| 2975 | - | 3264184 | 3264600 | + | *-* | *PA14_36650* | hypothetical protein | 40 | 106 | 73 | 122 | 98 | 99 | 0.022142733 |
| 2976 | - | 3264667 | 3265914 | + | *-* | *PA14_36660* | alcohol dehydrogenase | 17 | 34 | 30 | 44 | 32 | 34 | 0.418246621 |
| 2977 | - | 3265918 | 3266856 | + | *-* | *PA14_36670* | hypothetical protein | 17 | 27 | 32 | 41 | 31 | 32 | 0.931437202 |
| 2978 | - | 3266853 | 3267590 | + | *-* | *PA14_36680* | hypothetical protein | 13 | 24 | 22 | 31 | 21 | 19 | 0.580848337 |
| 2979 | - | 3267587 | 3268792 | + | *-* | *PA14_36690* | cardiolipin synthase 2 | 20 | 29 | 29 | 47 | 34 | 37 | 1 |
| 2980 | - | 3268789 | 3269784 | + | *-* | *PA14_36700* | hypothetical protein | 11 | 27 | 23 | 35 | 30 | 29 | 0.0839756 |
| 2981 | - | 3271984 | 3269786 | - | *glgB* | *PA14_36710* | glycogen branching protein | 23 | 47 | 42 | 61 | 49 | 50 | 0.415011252 |
| 2982 | - | 3275283 | 3271981 | - | *-* | *PA14_36730* | trehalose synthase | 28 | 46 | 41 | 58 | 50 | 51 | 1 |
| 2983 | - | 3277288 | 3275294 | - | *-* | *PA14_36740* | hypothetical protein | 26 | 33 | 34 | 41 | 36 | 37 | 1 |
| 2984 | - | 3278313 | 3277432 | - | *-* | *PA14_36760* | KU domain-containing protein | 10 | 16 | 13 | 17 | 15 | 17 | 1 |
| 2985 | - | 3278578 | 3278336 | - | *-* | *PA14_36770* | hypothetical protein | 5 | 10 | 12 | 13 | 9 | 10 | 0.828709947 |
| 2986 | - | 3279083 | 3278592 | - | *-* | *PA14_36780* | hypothetical protein | 33 | 48 | 63 | 67 | 60 | 54 | 1 |
| 2987 | - | 3279214 | 3279080 | - | *-* | *PA14_36790* | hypothetical protein | 41 | 71 | 86 | 89 | 74 | 67 | 0.849496297 |
| 2988 | - | 3281440 | 3279311 | - | *katE* | *PA14_36810* | hydroperoxidase II | 11 | 18 | 12 | 19 | 17 | 13 | 0.967915794 |
| 2989 | - | 3281688 | 3281521 | - | *-* | *PA14_36820* | hypothetical protein | 16 | 66 | 48 | 157 | 47 | 90 | 1.60548E-08 |
| 2990 | - | 3282187 | 3282585 | + | *-* | *PA14_36830* | hypothetical protein | 16 | 27 | 30 | 42 | 32 | 31 | 0.895687725 |
| 2991 | - | 3285030 | 3282592 | - | *glgP* | *PA14_36840* | glycogen phosphorylase | 34 | 49 | 51 | 62 | 53 | 54 | 1 |
| 2992 | - | 3285370 | 3285083 | - | *-* | *PA14_36850* | hypothetical protein | 205 | 298 | 530 | 528 | 380 | 316 | 1 |
| 2993 | - | 3285614 | 3285802 | + | *-* | *PA14_36860* | hypothetical protein | 40 | 61 | 65 | 64 | 63 | 81 | 1 |
| 2994 | - | 3286682 | 3285822 | - | *-* | *PA14_36870* | short-chain dehydrogenase | 26 | 40 | 41 | 46 | 38 | 48 | 1 |
| 2995 | - | 3287226 | 3286708 | - | *-* | *PA14_36880* | ompetence-damaged protein | 4 | 9 | 12 | 11 | 9 | 10 | 0.181497819 |
| 2996 | - | 3287476 | 3287237 | - | *-* | *PA14_36890* | metallothionein | 9 | 15 | 17 | 14 | 15 | 15 | 1 |
| 2997 | - | 3287711 | 3287496 | - | *-* | *PA14_36900* | hypothetical protein | 8 | 14 | 13 | 15 | 11 | 10 | 0.907738182 |
| 2998 | - | 3290309 | 3287787 | - | *ligD* | *PA14_36910* | ATP-dependent DNA ligase | 13 | 9 | 12 | 12 | 12 | 13 | 1 |
| 2999 | - | 3290863 | 3290327 | - | *-* | *PA14_36920* | hypothetical protein | 14 | 6 | 10 | 7 | 5 | 8 | 0.023098865 |
| 3000 | - | 3290965 | 3291489 | + | *-* | *PA14_36930* | hypothetical protein | 32 | 30 | 40 | 18 | 18 | 25 | 1 |
| 3001 | - | 3291515 | 3291964 | + | *-* | *PA14_36940* | hypothetical protein | 4 | 8 | 10 | 9 | 7 | 8 | 0.45673241 |
| 3002 | - | 3293306 | 3291942 | - | *-* | *PA14_36960* | transporter | 7 | 11 | 13 | 14 | 12 | 13 | 1 |
| 3003 | - | 3293907 | 3293335 | - | *-* | *PA14_36980* | hypothetical protein | 23 | 38 | 46 | 42 | 35 | 34 | 1 |
| 3004 | - | 3294857 | 3294000 | - | *-* | *PA14_36990* | EAL domain-containing protein | 4 | 3 | 4 | 4 | 3 | 2 | 1 |
| 3005 | - | 3295567 | 3294854 | - | *cupA5* | *PA14_37000* | chaperone CupA5 | 2 | 2 | 2 | 3 | 1 | 1 | 1 |
| 3006 | - | 3296918 | 3295557 | - | *cupA4* | *PA14_37010* | fimbrial subunit CupA4 | 2 | 2 | 3 | 3 | 2 | 2 | 1 |
| 3007 | - | 3299533 | 3296915 | - | *cupA3* | *PA14_37030* | usher | 7 | 6 | 7 | 8 | 5 | 4 | 1 |
| 3008 | - | 3300263 | 3299517 | - | *cupA2* | *PA14_37040* | chaperone CupA2 | 4 | 3 | 4 | 3 | 2 | 2 | 1 |
| 3009 | - | 3300902 | 3300351 | - | *cupA1* | *PA14_37060* | fimbrial subunit CupA1 | 16 | 16 | 11 | 11 | 12 | 6 | 1 |
| 3010 | - | 3301756 | 3302982 | + | *-* | *PA14_37070* | hypothetical protein | 146 | 131 | 184 | 87 | 76 | 125 | 1 |
| 3011 | - | 3303409 | 3304044 | + | *-* | *PA14_37080* | hypothetical protein | 81 | 97 | 104 | 50 | 56 | 69 | 1 |
| 3012 | - | 3305528 | 3304080 | - | *-* | *PA14_37090* | aldehyde dehydrogenase | 15 | 16 | 18 | 13 | 12 | 14 | 1 |
| 3013 | - | 3307187 | 3305550 | - | *-* | *PA14_37100* | dehydrogenase | 6 | 5 | 7 | 5 | 5 | 5 | 1 |
| 3014 | - | 3308224 | 3307292 | - | *-* | *PA14_37120* | LysR family transcriptional regulator | 18 | 17 | 17 | 19 | 14 | 20 | 1 |
| 3015 | - | 3309397 | 3308261 | - | *-* | *PA14_37130* | hypothetical protein | 11 | 12 | 11 | 12 | 12 | 14 | 1 |
| 3016 | - | 3309523 | 3310428 | + | *-* | *PA14_37140* | LysR family transcriptional regulator | 10 | 12 | 11 | 10 | 11 | 10 | 1 |
| 3017 | - | 3310576 | 3311004 | + | *-* | *PA14_37150* | hypothetical protein | 6 | 11 | 8 | 8 | 10 | 9 | 1 |
| 3018 | - | 3311021 | 3312133 | + | *-* | *PA14_37170* | hypothetical protein | 27 | 25 | 28 | 32 | 28 | 25 | 1 |
| 3019 | - | 3313744 | 3312668 | - | *ada* | *PA14_37190* | O6-methylguanine-DNA methyltransferase | 20 | 28 | 24 | 24 | 19 | 16 | 1 |
| 3020 | - | 3313936 | 3314919 | + | *-* | *PA14_37200* | hypothetical protein | 17 | 76 | 37 | 76 | 57 | 37 | 1.49732E-07 |
| 3021 | - | 3315701 | 3314904 | - | *-* | *PA14_37210* | hypothetical protein | 17 | 52 | 30 | 46 | 34 | 30 | 0.003189572 |
| 3022 | - | 3316761 | 3315808 | - | *-* | *PA14_37220* | LysR family transcriptional regulator | 3 | 4 | 4 | 3 | 4 | 2 | 1 |
| 3023 | - | 3317016 | 3318287 | + | *-* | *PA14_37250* | major facilitator transporter | 4 | 26 | 11 | 17 | 16 | 10 | 3.27339E-21 |
| 3024 | - | 3318312 | 3319541 | + | *-* | *PA14_37260* | porin | 9 | 18 | 12 | 14 | 12 | 13 | 0.445579457 |

|  | A | B | C | D | E | F | G | H | I | J | K | L | M | N |
| --- | --- | --- | --- | --- | --- | --- | --- | --- | --- | --- | --- | --- | --- | --- |
| 3025 | - | 3319574 | 3320317 | + | *-* | *PA14_37270* | LamB/YcsF family protein | 6 | 40 | 16 | 25 | 19 | 14 | 2.33936E-22 |
| 3026 | - | 3320314 | 3321027 | + | *-* | *PA14_37290* | hypothetical protein | 3 | 25 | 11 | 16 | 15 | 9 | 1.01442E-37 |
| 3027 | - | 3321024 | 3321965 | + | *-* | *PA14_37310* | hypothetical protein | 6 | 29 | 14 | 25 | 19 | 13 | 4.51048E-12 |
| 3028 | - | 3322033 | 3322509 | + | *-* | *PA14_37320* | hypothetical protein | 10 | 19 | 18 | 20 | 17 | 13 | 0.530445973 |
| 3029 | - | 3324300 | 3322528 | - | *-* | *PA14_37340* | thiamine pyrophosphate protein | 9 | 13 | 13 | 16 | 14 | 13 | 1 |
| 3030 | - | 3324471 | 3324869 | + | *-* | *PA14_37350* | hypothetical protein | 6 | 9 | 11 | 11 | 8 | 9 | 1 |
| 3031 | - | 3326026 | 3325268 | - | *-* | *PA14_37360* | short chain dehydrogenase | 8 | 11 | 9 | 11 | 8 | 10 | 1 |
| 3032 | - | 3326940 | 3326011 | - | *-* | *PA14_37370* | esterase | 4 | 5 | 3 | 3 | 3 | 4 | 1 |
| 3033 | - | 3328426 | 3326951 | - | *-* | *PA14_37380* | flavin-binding monooxygenase | 3 | 4 | 4 | 4 | 3 | 4 | 1 |
| 3034 | - | 3328573 | 3329607 | + | *-* | *PA14_37400* | AraC family transcriptional regulator | 16 | 14 | 17 | 15 | 12 | 14 | 1 |
| 3035 | - | 3329756 | 3330601 | + | *-* | *PA14_37410* | hypothetical protein | 9 | 10 | 9 | 11 | 8 | 11 | 1 |
| 3036 | - | 3331559 | 3330603 | - | *-* | *PA14_37420* | transmembrane sensor protein | 10 | 8 | 8 | 7 | 9 | 5 | 1 |
| 3037 | - | 3332065 | 3331556 | - | *-* | *PA14_37430* | RNA polymerase sigma factor | 8 | 8 | 7 | 5 | 7 | 6 | 1 |
| 3038 | - | 3333364 | 3332168 | - | *-* | *PA14_37440* | MFS transporter | 19 | 13 | 20 | 18 | 18 | 18 | 1 |
| 3039 | - | 3334634 | 3333351 | - | *-* | *PA14_37460* | permease | 7 | 6 | 5 | 5 | 8 | 6 | 1 |
| 3040 | - | 3335710 | 3334631 | - | *-* | *PA14_37470* | flavin-dependent oxidoreductase | 8 | 6 | 5 | 4 | 5 | 5 | 1 |
| 3041 | - | 3338374 | 3335723 | - | *-* | *PA14_37490* | TonB-dependent receptor | 23 | 21 | 22 | 21 | 18 | 20 | 1 |
| 3042 | - | 3339314 | 3338466 | - | *-* | *PA14_37510* | hypothetical protein | 12 | 10 | 10 | 9 | 10 | 10 | 1 |
| 3043 | - | 3339954 | 3339298 | - | *-* | *PA14_37520* | hypothetical protein | 10 | 7 | 5 | 6 | 7 | 8 | 1 |
| 3044 | - | 3340853 | 3339951 | - | *-* | *PA14_37530* | hydrolase | 8 | 8 | 6 | 7 | 6 | 6 | 1 |
| 3045 | - | 3341373 | 3340864 | - | *-* | *PA14_37550* | ring-hydroxylating dioxygenase small subunit | 11 | 9 | 8 | 7 | 10 | 7 | 1 |
| 3046 | - | 3343233 | 3341401 | - | *-* | *PA14_37560* | asparagine synthetase, glutamine-hydrolysing | 6 | 7 | 4 | 3 | 6 | 4 | 1 |
| 3047 | - | 3344595 | 3343321 | - | *-* | *PA14_37570* | ring-hydroxylating dioxygenase, large terminal subunit | 11 | 13 | 12 | 12 | 16 | 13 | 1 |
| 3048 | - | 3345273 | 3344797 | - | *-* | *PA14_37580* | leucine-responsive regulatory protein | 41 | 39 | 42 | 37 | 45 | 51 | 1 |
| 3049 | - | 3345406 | 3346047 | + | *kynB* | *PA14_37590* | kynurenine formamidase, KynB | 28 | 43 | 35 | 32 | 36 | 29 | 1 |
| 3050 | - | 3346051 | 3347301 | + | *-* | *PA14_37610* | kynureninase | 38 | 58 | 40 | 41 | 45 | 37 | 1 |
| 3051 | - | 3347457 | 3348863 | + | *-* | *PA14_37630* | amino acid permease | 14 | 17 | 13 | 14 | 16 | 14 | 1 |
| 3052 | - | 3349149 | 3351023 | + | *-* | *PA14_37640* | hypothetical protein | 8 | 8 | 9 | 10 | 9 | 9 | 1 |
| 3053 | - | 3351063 | 3352967 | + | *-* | *PA14_37650* | hypothetical protein | 8 | 8 | 9 | 8 | 8 | 7 | 1 |
| 3054 | - | 3353906 | 3353004 | - | *-* | *PA14_37660* | transcriptional regulator | 14 | 19 | 20 | 19 | 18 | 15 | 1 |
| 3055 | - | 3354363 | 3354629 | + | *-* | *PA14_37670* | hypothetical protein | 41 | 40 | 65 | 39 | 35 | 43 | 1 |
| 3056 | - | 3354806 | 3356473 | + | *-* | *PA14_37680* | hypothetical protein | 17 | 16 | 22 | 16 | 18 | 21 | 1 |
| 3057 | - | 3359460 | 3356866 | - | *-* | *PA14_37690* | sensory box protein | 57 | 48 | 65 | 93 | 58 | 91 | 1 |
| 3058 | - | 3361740 | 3359632 | - | *fusA2* | *PA14_37710* | elongation factor G | 116 | 81 | 106 | 153 | 98 | 153 | 1 |
| 3059 | - | 3362016 | 3364658 | + | *-* | *PA14_37730* | TonB dependent receptor | 27 | 15 | 21 | 31 | 22 | 32 | 1 |
| 3060 | 3364761 | 3364838 | 3366562 | + | *-* | *PA14_37745* | carbamoyl transferase | 204 | 5 | 13 | 135 | 82 | 232 | 0 |
| 3061 | 3364761 | 3366606 | 3367769 | + | *-* | *PA14_37760* | MFS transporter | 115 | 7 | 12 | 71 | 57 | 127 | 3.8621E-279 |
| 3062 | 3364761 | 3367771 | 3368439 | + | *-* | *PA14_37770* | hydrolase | 195 | 18 | 27 | 128 | 88 | 205 | 7.70864E-77 |
| 3063 | 3364761 | 3368436 | 3369074 | + | *-* | *PA14_37780* | hypothetical protein | 110 | 16 | 20 | 76 | 56 | 122 | 2.0367E-25 |
| 3064 | - | 3369223 | 3371043 | + | *pcoA* | *PA14_37790* | copper resistance protein A | 9 | 7 | 8 | 10 | 7 | 9 | 1 |
| 3065 | - | 3371040 | 3372095 | + | *pcoB* | *PA14_37810* | copper resistance protein B | 8 | 4 | 4 | 5 | 6 | 6 | 0.037558237 |
| 3066 | - | 3373338 | 3372115 | - | *-* | *PA14_37820* | hypothetical protein | 9 | 9 | 10 | 7 | 10 | 7 | 1 |
| 3067 | - | 3374722 | 3373541 | - | *-* | *PA14_37830* | pyridoxal-phosphate dependent protein | 10 | 15 | 10 | 12 | 12 | 8 | 1 |
| 3068 | - | 3376478 | 3374868 | - | *-* | *PA14_37840* | ABC transporter ATP-binding protein | 9 | 8 | 10 | 11 | 7 | 8 | 1 |
| 3069 | - | 3377496 | 3376480 | - | *-* | *PA14_37850* | ABC transporter permease | 1 | 1 | 1 | 1 | 1 | 1 | 1 |
| 3070 | - | 3378571 | 3377498 | - | *-* | *PA14_37870* | peptide ABC transporter permease | 3 | 2 | 3 | 3 | 2 | 2 | 1 |
| 3071 | - | 3380381 | 3378573 | - | *-* | *PA14_37880* | ABC transporter substrate-binding protein | 3 | 3 | 2 | 3 | 3 | 3 | 1 |
| 3072 | - | 3382925 | 3380385 | - | *-* | *PA14_37900* | TonB-dependent receptor | 4 | 5 | 6 | 7 | 4 | 6 | 1 |
| 3073 | - | 3384506 | 3383604 | - | *-* | *PA14_37910* | LysR family transcriptional regulator | 5 | 8 | 5 | 6 | 8 | 4 | 1 |
| 3074 | - | 3384617 | 3386032 | + | *-* | *PA14_37915* | major facilitator transporter | 9 | 8 | 8 | 5 | 6 | 6 | 1 |
| 3075 | - | 3386926 | 3386039 | - | *cynR* | *PA14_37940* | DNA-binding transcriptional regulator CynR | 25 | 27 | 27 | 18 | 20 | 20 | 1 |
| 3076 | - | 3387041 | 3387703 | + | *cynT* | *PA14_37950* | carbonate dehydratase | 10 | 10 | 10 | 11 | 9 | 9 | 1 |
| 3077 | - | 3387743 | 3388213 | + | *cynS* | *PA14_37965* | cyanate hydratase | 12 | 19 | 11 | 14 | 15 | 15 | 1 |
| 3078 | - | 3389204 | 3388251 | - | *-* | *PA14_37980* | Fe2+-dicitrate sensor, membrane protein | 3 | 4 | 2 | 2 | 2 | 2 | 1 |
| 3079 | - | 3389707 | 3389201 | - | *-* | *PA14_37990* | RNA polymerase sigma factor | 1 | 2 | 2 | 2 | 2 | 3 | 1 |
| 3080 | - | 3390035 | 3390499 | + | *-* | *PA14_38000* | hypothetical protein | 21 | 13 | 24 | 13 | 11 | 18 | 0.434308967 |
| 3081 | - | 3390598 | 3392031 | + | *-* | *PA14_38010* | hypothetical protein | 42 | 38 | 39 | 41 | 38 | 37 | 1 |
| 3082 | - | 3392496 | 3392089 | - | *-* | *PA14_38020* | ntibiotic biosynthesis monooxygenase | 24 | 24 | 28 | 24 | 16 | 19 | 1 |
| 3083 | - | 3392910 | 3393899 | + | *-* | *PA14_38040* | AraC family transcriptional regulator | 28 | 23 | 22 | 24 | 20 | 23 | 1 |
| 3084 | - | 3394935 | 3395342 | + | *-* | *PA14_38050* | hypothetical protein | 20 | 14 | 24 | 20 | 27 | 20 | 0.958024133 |
| 3085 | - | 3395401 | 3395661 | + | *-* | *PA14_38060* | hypothetical protein | 21 | 18 | 20 | 18 | 18 | 17 | 1 |
| 3086 | - | 3395836 | 3397710 | + | *-* | *PA14_38080* | hypothetical protein | 34 | 40 | 35 | 38 | 35 | 33 | 1 |
| 3087 | - | 3398770 | 3397868 | - | *-* | *PA14_38090* | pseudouridylate synthase | 36 | 40 | 41 | 34 | 37 | 27 | 1 |
| 3088 | - | 3400120 | 3398891 | - | *-* | *PA14_38110* | serine/threonine transporter SstT | 32 | 36 | 34 | 37 | 38 | 23 | 1 |
| 3089 | - | 3401978 | 3400608 | - | *-* | *PA14_38130* | amino acid permease | 14 | 19 | 15 | 18 | 14 | 13 | 1 |
| 3090 | - | 3403511 | 3402135 | - | *-* | *PA14_38140* | glutamine synthetase | 50 | 49 | 41 | 51 | 39 | 48 | 1 |
| 3091 | - | 3404017 | 3404775 | + | *-* | *PA14_38160* | hypothetical protein | 17 | 13 | 17 | 15 | 13 | 11 | 1 |
| 3092 | - | 3404760 | 3405074 | + | *-* | *PA14_38170* | hypothetical protein | 19 | 25 | 26 | 25 | 25 | 23 | 1 |
| 3093 | - | 3406659 | 3405199 | - | *-* | *PA14_38180* | hypothetical protein | 9 | 11 | 9 | 11 | 13 | 10 | 1 |
| 3094 | - | 3407320 | 3406799 | - | *-* | *PA14_38190* | hypothetical protein | 2 | 1 | 1 | 2 | 1 | 0 | 0.853730745 |
| 3095 | - | 3408055 | 3409713 | + | *-* | *PA14_38200* | thiamine pyrophosphate protein | 34 | 33 | 34 | 40 | 33 | 31 | 1 |
| 3096 | - | 3410395 | 3409721 | - | *-* | *PA14_38210* | hypothetical protein | 27 | 37 | 26 | 83 | 31 | 22 | 1 |
| 3097 | - | 3411303 | 3410395 | - | *-* | *PA14_38220* | hypothetical protein | 24 | 40 | 25 | 110 | 38 | 26 | 0.831724413 |
| 3098 | - | 3412862 | 3411438 | - | *-* | *PA14_38250* | transcriptional regulator | 36 | 29 | 31 | 38 | 27 | 31 | 1 |
| 3099 | - | 3413042 | 3413296 | + | *-* | *PA14_38260* | hypothetical protein | 85 | 25 | 37 | 57 | 39 | 96 | 1.16747E-06 |
| 3100 | - | 3413293 | 3413550 | + | *-* | *PA14_38270* | hypothetical protein | 179 | 74 | 99 | 161 | 110 | 234 | 0.083747076 |
| 3101 | - | 3413924 | 3413625 | - | *-* | *PA14_38290* | hypothetical protein | 18 | 21 | 20 | 23 | 18 | 20 | 1 |
| 3102 | - | 3414421 | 3413948 | - | *-* | *PA14_38300* | transcriptional regulator | 11 | 8 | 10 | 7 | 7 | 10 | 1 |
| 3103 | - | 3414554 | 3414946 | + | *-* | *PA14_38310* | hypothetical protein | 4 | 3 | 6 | 5 | 4 | 7 | 1 |
| 3104 | - | 3415099 | 3416100 | + | *-* | *PA14_38320* | bile acid/Na+ symporter family transporter | 68 | 47 | 70 | 38 | 52 | 67 | 1 |
| 3105 | - | 3417510 | 3416155 | - | *gor* | *PA14_38330* | glutathione reductase | 259 | 236 | 250 | 222 | 246 | 263 | 1 |
| 3106 | - | 3417655 | 3418077 | + | *-* | *PA14_38340* | ring-cleaving dioxygenase | 130 | 176 | 185 | 191 | 235 | 224 | 1 |
| 3107 | - | 3419098 | 3418259 | - | *galU* | *PA14_38350* | UTP-glucose-1-phosphate uridylyltransferase | 191 | 144 | 199 | 138 | 151 | 144 | 1 |
| 3108 | - | 3420507 | 3419146 | - | *-* | *PA14_38360* | nucleotide sugar dehydrogenase | 12 | 12 | 19 | 17 | 15 | 14 | 1 |

|  | A | B | C | D | E | F | G | H | I | J | K | L | M | N |
| --- | --- | --- | --- | --- | --- | --- | --- | --- | --- | --- | --- | --- | --- | --- |
| 3109 | - | 3420659 | 3420883 | + | *-* | *PA14_38370* | hypothetical protein | 24 | 28 | 31 | 33 | 33 | 34 | 1 |
| 3110 | - | 3421521 | 3420889 | - | *-* | *PA14_38380* | transcriptional regulator | 70 | 62 | 71 | 71 | 66 | 61 | 1 |
| 3111 | - | 3421686 | 3422876 | + | *-* | *PA14_38395* | periplasmic multidrug efflux lipoprotein | 7 | 7 | 7 | 5 | 6 | 7 | 1 |
| 3112 | - | 3422892 | 3426029 | + | *amrB* | *PA14_38410* | multidrug efflux protein | 9 | 10 | 12 | 12 | 10 | 11 | 1 |
| 3113 | - | 3427200 | 3426271 | - | *-* | *PA14_38420* | hypothetical protein | 46 | 43 | 47 | 51 | 51 | 54 | 1 |
| 3114 | - | 3427393 | 3427797 | + | *gnyR* | *PA14_38430* | regulatory gene of gnyRDBHAL cluster, GnyR | 232 | 336 | 280 | 229 | 428 | 366 | 1 |
| 3115 | - | 3427846 | 3429009 | + | *gnyD* | *PA14_38440* | citronelloyl-CoA dehydrogenase, GnyD | 432 | 689 | 446 | 537 | 637 | 617 | 1 |
| 3116 | - | 3429132 | 3430739 | + | *gnyB* | *PA14_38460* | acyl-CoA carboxyltransferase subunit beta | 218 | 302 | 213 | 250 | 271 | 269 | 1 |
| 3117 | - | 3430753 | 3431550 | + | *gnyH* | *PA14_38470* | gamma-carboxygeranoyl-CoA hydratase | 156 | 218 | 140 | 153 | 187 | 169 | 1 |
| 3118 | - | 3431547 | 3433514 | + | *gnyA* | *PA14_38480* | alpha subunit of geranoyl-CoA carboxylase, GnyA | 267 | 365 | 247 | 310 | 326 | 320 | 1 |
| 3119 | - | 3433535 | 3434437 | + | *gnyL* | *PA14_38490* | hydroxymethylglutaryl-CoA lyase | 456 | 505 | 419 | 470 | 506 | 497 | 1 |
| 3120 | - | 3435308 | 3434505 | - | *-* | *PA14_38500* | IclR family transcriptional regulator | 87 | 103 | 126 | 90 | 125 | 109 | 1 |
| 3121 | - | 3435469 | 3436767 | + | *hmgA* | *PA14_38510* | homogentisate 1,2-dioxygenase | 299 | 308 | 289 | 239 | 313 | 340 | 1 |
| 3122 | - | 3436772 | 3438070 | + | *fahA* | *PA14_38530* | fumarylacetoacetase | 245 | 251 | 168 | 232 | 268 | 304 | 1 |
| 3123 | - | 3438067 | 3438705 | + | *maiA* | *PA14_38550* | maleylacetoacetate isomerase | 283 | 273 | 201 | 264 | 273 | 320 | 1 |
| 3124 | - | 3438790 | 3440142 | + | *-* | *PA14_38560* | MFS transporter | 16 | 10 | 11 | 12 | 12 | 16 | 1 |
| 3125 | - | 3440271 | 3441641 | + | *-* | *PA14_38570* | transcriptional regulator | 18 | 12 | 13 | 12 | 13 | 16 | 1 |
| 3126 | - | 3441918 | 3443309 | + | *-* | *PA14_38580* | hypothetical protein | 12 | 49 | 35 | 95 | 38 | 53 | 4.78316E-06 |
| 3127 | - | 3443343 | 3444113 | + | *bdhA* | *PA14_38590* | 3-hydroxybutyrate dehydrogenase | 27 | 141 | 90 | 194 | 106 | 117 | 3.91329E-11 |
| 3128 | - | 3445728 | 3444304 | - | *-* | *PA14_38610* | hypothetical protein | 23 | 40 | 31 | 42 | 36 | 42 | 0.767674398 |
| 3129 | - | 3447122 | 3445941 | - | *atoB* | *PA14_38630* | acetyl-CoA acetyltransferase | 250 | 343 | 233 | 320 | 259 | 396 | 1 |
| 3130 | - | 3447928 | 3447272 | - | *-* | *PA14_38640* | CoA transferase subunit B | 184 | 242 | 134 | 168 | 191 | 267 | 1 |
| 3131 | - | 3448661 | 3447963 | - | *-* | *PA14_38660* | CoA transferase, subunit A | 382 | 401 | 273 | 309 | 323 | 436 | 1 |
| 3132 | - | 3448793 | 3449713 | + | *-* | *PA14_38680* | LysR family transcriptional regulator | 15 | 16 | 16 | 18 | 18 | 19 | 1 |
| 3133 | - | 3449781 | 3451736 | + | *-* | *PA14_38690* | acetoacetyl-CoA synthetase | 56 | 49 | 52 | 56 | 44 | 66 | 1 |
| 3134 | - | 3451794 | 3452072 | + | *ppiC1* | *PA14_38700* | peptidyl-prolyl cis-trans isomerase C1 | 59 | 57 | 63 | 73 | 48 | 55 | 1 |
| 3135 | - | 3452091 | 3452447 | + | *-* | *PA14_38710* | hypothetical protein | 103 | 107 | 129 | 131 | 112 | 120 | 1 |
| 3136 | - | 3452444 | 3453007 | + | *-* | *PA14_38720* | hypothetical protein | 35 | 33 | 37 | 36 | 28 | 36 | 1 |
| 3137 | - | 3453132 | 3454340 | + | *-* | *PA14_38730* | major facilitator superfamily transporter | 13 | 10 | 14 | 11 | 8 | 14 | 1 |
| 3138 | - | 3456069 | 3454285 | - | *-* | *PA14_38740* | two-component sensor | 33 | 39 | 27 | 34 | 31 | 35 | 1 |
| 3139 | - | 3457216 | 3456053 | - | *-* | *PA14_38750* | iron-containing alcohol dehydrogenase | 26 | 31 | 24 | 27 | 27 | 27 | 1 |
| 3140 | - | 3459128 | 3457302 | - | *-* | *PA14_38770* | peptidase | 67 | 38 | 55 | 53 | 44 | 53 | 1 |
| 3141 | - | 3460278 | 3459133 | - | *pqqE* | *PA14_38780* | pyrroloquinoline quinone biosynthesis protein PqqE | 68 | 52 | 54 | 68 | 48 | 60 | 1 |
| 3142 | - | 3460528 | 3460250 | - | *pqqD* | *PA14_38790* | pyrroloquinoline quinone biosynthesis protein PqqD | 43 | 33 | 35 | 40 | 36 | 37 | 1 |
| 3143 | - | 3461277 | 3460525 | - | *pqqC* | *PA14_38800* | pyrroloquinoline quinone biosynthesis protein PqqC | 61 | 43 | 48 | 50 | 45 | 52 | 1 |
| 3144 | - | 3462201 | 3461287 | - | *pqqB* | *PA14_38820* | pyrroloquinoline quinone biosynthesis protein PqqB | 37 | 26 | 28 | 29 | 25 | 25 | 1 |
| 3145 | - | 3462325 | 3462254 | - | *pqqA* | *PA14_38825* | coenzyme PQQ synthesis protein PqqA | 56 | 52 | 53 | 54 | 38 | 58 | 1 |
| 3146 | - | 3464203 | 3462683 | - | *-* | *PA14_38840* | NAD+ dependent acetaldehyde dehydrogenase | 80 | 150 | 108 | 94 | 94 | 88 | 1 |
| 3147 | - | 3464727 | 3464290 | - | *exaB* | *PA14_38850* | cytochrome c550 | 3 | 3 | 4 | 4 | 3 | 5 | 1 |
| 3148 | - | 3465040 | 3466911 | + | *exaA* | *PA14_38860* | quinoprotein alcohol dehydrogenase | 5 | 5 | 8 | 7 | 4 | 6 | 1 |
| 3149 | - | 3466965 | 3467612 | + | *-* | *PA14_38880* | hypothetical protein | 4 | 2 | 4 | 3 | 4 | 3 | 1 |
| 3150 | - | 3468316 | 3467639 | - | *-* | *PA14_38900* | two-component response regulator | 7 | 5 | 8 | 5 | 7 | 7 | 1 |
| 3151 | - | 3468979 | 3468329 | - | *-* | *PA14_38910* | sensor kinase | 10 | 7 | 9 | 6 | 6 | 7 | 0.831724413 |
| 3152 | - | 3469164 | 3468988 | - | *-* | *PA14_38920* | hypothetical protein | 10 | 8 | 11 | 9 | 7 | 10 | 1 |
| 3153 | - | 3469644 | 3470309 | + | *-* | *PA14_38930* | transcriptional regulator | 17 | 13 | 19 | 15 | 12 | 12 | 1 |
| 3154 | - | 3470319 | 3471182 | + | *-* | *PA14_38950* | hypothetical protein | 11 | 7 | 13 | 10 | 9 | 8 | 0.555553781 |
| 3155 | - | 3473829 | 3471184 | - | *-* | *PA14_38970* | two-component sensor | 9 | 5 | 6 | 6 | 4 | 5 | 0.770507726 |
| 3156 | - | 3474922 | 3473759 | - | *-* | *PA14_38990* | hypothetical protein | 10 | 7 | 8 | 8 | 4 | 5 | 0.956402005 |
| 3157 | - | 3476224 | 3475025 | - | *-* | *PA14_39000* | hypothetical protein | 2 | 2 | 3 | 3 | 2 | 2 | 1 |
| 3158 | - | 3478818 | 3476491 | - | *pqqF* | *PA14_39010* | pyrroloquinoline quinone biosynthesis protein F | 40 | 26 | 27 | 25 | 24 | 22 | 1 |
| 3159 | - | 3480598 | 3478895 | - | *-* | *PA14_39020* | hypothetical protein | 5 | 5 | 5 | 5 | 3 | 5 | 1 |
| 3160 | - | 3481126 | 3482439 | + | *braZ* | *PA14_39050* | branched-chain amino acid transport carrier | 9 | 8 | 10 | 8 | 7 | 7 | 1 |
| 3161 | - | 3482675 | 3482914 | + | *-* | *PA14_39060* | lipoprotein | 31 | 29 | 30 | 22 | 30 | 23 | 1 |
| 3162 | - | 3483373 | 3482981 | - | *-* | *PA14_39070* | hypothetical protein | 76 | 121 | 63 | 49 | 80 | 45 | 0.950259107 |
| 3163 | - | 3483653 | 3483420 | - | *-* | *PA14_39080* | hypothetical protein | 26 | 24 | 25 | 17 | 20 | 14 | 1 |
| 3164 | - | 3483838 | 3484338 | + | *-* | *PA14_39090* | hypothetical protein | 88 | 121 | 110 | 108 | 115 | 103 | 1 |
| 3165 | - | 3484730 | 3484359 | - | *-* | *PA14_39100* | hypothetical protein | 93 | 116 | 127 | 111 | 120 | 115 | 1 |
| 3166 | - | 3485360 | 3485025 | - | *-* | *PA14_39110* | hypothetical protein | 41 | 36 | 51 | 46 | 31 | 37 | 1 |
| 3167 | - | 3485688 | 3487253 | + | *-* | *PA14_39130* | ABC transporter ATP-binding protein | 71 | 49 | 81 | 50 | 54 | 60 | 1 |
| 3168 | - | 3487606 | 3487340 | - | *-* | *PA14_39140* | hypothetical protein | 163 | 142 | 148 | 163 | 153 | 188 | 1 |
| 3169 | - | 3488321 | 3487713 | - | *-* | *PA14_39150* | azoreductase | 8 | 5 | 9 | 5 | 6 | 6 | 0.956402005 |
| 3170 | - | 3488469 | 3489404 | + | *-* | *PA14_39160* | LysR family transcriptional regulator | 20 | 18 | 26 | 18 | 18 | 20 | 1 |
| 3171 | - | 3489849 | 3489415 | - | *-* | *PA14_39180* | hypothetical protein | 20 | 22 | 22 | 21 | 21 | 18 | 1 |
| 3172 | - | 3491037 | 3490204 | - | *bacA* | *PA14_39190* | UDP pyrophosphate phosphatase | 13 | 9 | 14 | 9 | 9 | 8 | 1 |
| 3173 | - | 3491319 | 3491894 | + | *-* | *PA14_39200* | transporter | 38 | 35 | 48 | 47 | 32 | 28 | 1 |
| 3174 | - | 3491891 | 3492418 | + | *-* | *PA14_39210* | hypothetical protein | 18 | 19 | 18 | 17 | 18 | 18 | 1 |
| 3175 | - | 3492721 | 3493212 | + | *-* | *PA14_39220* | hypothetical protein | 2 | 1 | 3 | 3 | 2 | 3 | 1 |
| 3176 | - | 3493209 | 3493778 | + | *-* | *PA14_39230* | hypothetical protein | 4 | 5 | 5 | 3 | 3 | 5 | 1 |
| 3177 | - | 3493835 | 3494857 | + | *-* | *PA14_39240* | hypothetical protein | 5 | 5 | 6 | 6 | 5 | 6 | 1 |
| 3178 | - | 3494919 | 3495599 | + | *-* | *PA14_39250* | double-glycine peptidase | 8 | 9 | 7 | 10 | 6 | 8 | 1 |
| 3179 | - | 3495613 | 3496365 | + | *-* | *PA14_39260* | hypothetical protein | 4 | 4 | 5 | 4 | 3 | 3 | 1 |
| 3180 | - | 3496426 | 3497691 | + | *-* | *PA14_39270* | hypothetical protein | 34 | 53 | 46 | 65 | 46 | 51 | 0.940967938 |
| 3181 | - | 3498751 | 3497825 | - | *rbsK* | *PA14_39280* | ribokinase | 167 | 416 | 300 | 468 | 391 | 313 | 0.723542393 |
| 3182 | - | 3499818 | 3498805 | - | *rbsR* | *PA14_39300* | ribose operon repressor RbsR | 155 | 374 | 287 | 387 | 308 | 278 | 0.80035376 |
| 3183 | - | 3500820 | 3499822 | - | *rbsC* | *PA14_39320* | membrane protein component of ABC ribose transporter | 40 | 147 | 92 | 136 | 107 | 91 | 0.000387326 |
| 3184 | - | 3502376 | 3500844 | - | *rbsA* | *PA14_39330* | ribose transporter | 71 | 263 | 189 | 264 | 219 | 179 | 0.016403065 |
| 3185 | - | 3503357 | 3502398 | - | *rbsB* | *PA14_39350* | ribose ABC transporter substrate-binding protein | 104 | 512 | 343 | 533 | 479 | 349 | 1.45127E-05 |
| 3186 | - | 3504904 | 3503576 | - | *-* | *PA14_39360* | sigma-54 dependent transcriptional regulator | 32 | 32 | 32 | 41 | 33 | 38 | 1 |
| 3187 | - | 3506527 | 3505040 | - | *-* | *PA14_39390* | 30S ribosomal protein S6 modification protein | 42 | 40 | 41 | 54 | 42 | 51 | 1 |
| 3188 | - | 3506718 | 3507815 | + | *-* | *PA14_39410* | hypothetical protein | 35 | 34 | 34 | 32 | 29 | 34 | 1 |
| 3189 | - | 3507877 | 3508107 | + | *-* | *PA14_39420* | hypothetical protein | 16 | 8 | 8 | 10 | 6 | 16 | 0.401171332 |
| 3190 | - | 3508418 | 3510313 | + | *-* | *PA14_39440* | hypothetical protein | 29 | 31 | 25 | 31 | 29 | 29 | 1 |
| 3191 | - | 3510492 | 3511631 | + | *-* | *PA14_39460* | hypothetical protein | 47 | 41 | 44 | 53 | 45 | 48 | 1 |
| 3192 | - | 3512076 | 3512852 | + | *-* | *PA14_39470* | hypothetical protein | 76 | 52 | 42 | 37 | 46 | 57 | 1 |

|  | A | B | C | D | E | F | G | H | I | J | K | L | M | N |
| --- | --- | --- | --- | --- | --- | --- | --- | --- | --- | --- | --- | --- | --- | --- |
| 3193 | - | 3513093 | 3515246 | + | *-* | *PA14_39480* | hypothetical protein | 115 | 65 | 54 | 76 | 88 | 114 | 1 |
| 3194 | - | 3516510 | 3516130 | - | *-* | *PA14_39500* | hypothetical protein | 21 | 23 | 21 | 20 | 20 | 20 | 1 |
| 3195 | - | 3519209 | 3517005 | - | *-* | *PA14_39520* | hydroxylase large subunit | 24 | 34 | 33 | 43 | 40 | 41 | 1 |
| 3196 | - | 3520195 | 3519206 | - | *-* | *PA14_39530* | hydroxylase molybdopterin-containing subunit | 27 | 28 | 28 | 32 | 26 | 23 | 1 |
| 3197 | - | 3520704 | 3520192 | - | *-* | *PA14_39540* | ferredoxin | 43 | 47 | 52 | 51 | 44 | 44 | 1 |
| 3198 | - | 3522169 | 3520874 | - | *-* | *PA14_39560* | chemotaxis transducer | 65 | 77 | 88 | 94 | 70 | 104 | 1 |
| 3199 | - | 3522478 | 3522774 | + | *-* | *PA14_39570* | hypothetical protein | 13 | 12 | 13 | 10 | 11 | 10 | 1 |
| 3200 | - | 3522821 | 3523390 | + | *rimJ* | *PA14_39580* | ribosomal protein alanine acetyltransferase | 12 | 14 | 11 | 10 | 12 | 14 | 1 |
| 3201 | - | 3525752 | 3523452 | - | *metE* | *PA14_39590* | 5-methyltetrahydropteroyltriglutamate/homocysteine S-methyltransferase | 22 | 8 | 10 | 11 | 8 | 31 | 0.049546026 |
| 3202 | - | 3527735 | 3525885 | - | *-* | *PA14_39610* | hypothetical protein | 34 | 38 | 47 | 36 | 34 | 29 | 1 |
| 3203 | - | 3528562 | 3528236 | - | *-* | *PA14_39620* | hypothetical protein | 3 | 4 | 3 | 3 | 4 | 3 | 1 |
| 3204 | - | 3529038 | 3528562 | - | *-* | *PA14_39630* | hypothetical protein | 1 | 1 | 1 | 1 | 1 | 1 | 1 |
| 3205 | - | 3532880 | 3529035 | - | *cobN* | *PA14_39640* | cobaltochelatase subunit CobN | 3 | 3 | 4 | 4 | 3 | 4 | 1 |
| 3206 | - | 3534841 | 3532880 | - | *-* | *PA14_39650* | TonB-dependent receptor | 2 | 2 | 2 | 3 | 3 | 2 | 1 |
| 3207 | - | 3535009 | 3535818 | + | *-* | *PA14_39660* | hypothetical protein | 7 | 7 | 5 | 5 | 6 | 5 | 1 |
| 3208 | - | 3535948 | 3535826 | - | *-* | *PA14_39670* | hypothetical protein | 2 | 1 | 2 | 1 | 3 | 3 | 0.239905719 |
| 3209 | - | 3535976 | 3538003 | + | *-* | *PA14_39690* | anaerobic ribonucleoside triphosphate reductase | 16 | 14 | 15 | 14 | 15 | 18 | 1 |
| 3210 | - | 3538047 | 3538193 | + | *-* | *PA14_39700* | hypothetical protein | 23 | 32 | 29 | 24 | 21 | 22 | 1 |
| 3211 | - | 3538190 | 3538888 | + | *-* | *PA14_39710* | radical SAM protein | 80 | 74 | 75 | 90 | 72 | 77 | 1 |
| 3212 | - | 3539082 | 3540482 | + | *-* | *PA14_39720* | amino acid oxidase | 5 | 5 | 5 | 6 | 5 | 6 | 1 |
| 3213 | - | 3540495 | 3540842 | + | *-* | *PA14_39730* | hypothetical protein | 4 | 3 | 4 | 4 | 5 | 5 | 1 |
| 3214 | - | 3540887 | 3542119 | + | *-* | *PA14_39750* | amino acid permease | 7 | 8 | 11 | 9 | 8 | 9 | 1 |
| 3215 | - | 3542183 | 3543730 | + | *-* | *PA14_39770* | regulatory protein | 50 | 48 | 55 | 59 | 50 | 54 | 1 |
| 3216 | - | 3544298 | 3545524 | + | *-* | *PA14_39780* | hypothetical protein | 30 | 10 | 12 | 17 | 19 | 86 | 0.003750459 |
| 3217 | - | 3545631 | 3546311 | + | *-* | *PA14_39790* | hypothetical protein | 46 | 37 | 48 | 44 | 41 | 33 | 1 |
| 3218 | - | 3546403 | 3546909 | + | *-* | *PA14_39800* | ECF subfamily RNA polymerase sigma-70 factor | 6 | 42 | 9 | 96 | 39 | 7 | 2.77209E-25 |
| 3219 | - | 3546906 | 3547856 | + | *-* | *PA14_39810* | transmembrane sensor | 8 | 32 | 12 | 90 | 32 | 9 | 9.20065E-07 |
| 3220 | - | 3548064 | 3550478 | + | *-* | *PA14_39820* | tonB-dependent receptor protein | 6 | 6 | 7 | 11 | 7 | 6 | 1 |
| 3221 | - | 3550519 | 3551670 | + | *-* | *PA14_39830* | hypothetical protein | 4 | 3 | 3 | 5 | 3 | 3 | 1 |
| 3222 | - | 3551857 | 3553068 | + | *-* | *PA14_39850* | MFS transporter | 9 | 2 | 4 | 4 | 3 | 6 | 1.56217E-07 |
| 3223 | - | 3553065 | 3554705 | + | *-* | *PA14_39860* | hypothetical protein | 89 | 5 | 6 | 14 | 10 | 66 | 0 |
| 3224 | - | 3554748 | 3555284 | + | *-* | *PA14_39870* | hydrolase | 226 | 10 | 15 | 31 | 28 | 167 | 0 |
| 3225 | 3561969 | 3556106 | 3555459 | - | *phzG2* | *PA14_39880* | pyridoxamine 5'-phosphate oxidase | 897 | 11 | 30 | 70 | 78 | 704 | 0 |
| 3226 | 3561969 | 3556965 | 3556129 | - | *phzF2* | *PA14_39890* | phenazine biosynthesis protein | 899 | 10 | 27 | 57 | 72 | 708 | 0 |
| 3227 | 3561969 | 3558862 | 3556979 | - | *phzE2* | *PA14_39910* | phenazine biosynthesis protein PhzE | 1059 | 14 | 37 | 79 | 84 | 814 | 0 |
| 3228 | 3561969 | 3559482 | 3558859 | - | *phzD2* | *PA14_39925* | phenazine biosynthesis protein PhzD | 888 | 6 | 19 | 67 | 76 | 691 | 0 |
| 3229 | 3561157 | 3560696 | 3559479 | - | *phzC2* | *PA14_39945* | phenazine biosynthesis protein PhzC | 1323 | 9 | 38 | 88 | 81 | 870 | 0 |
| 3230 | 3561157 | 3561208 | 3560720 | - | *phzB2* | *PA14_39960* | phenazine biosynthesis protein | 1000 | 9 | 24 | 119 | 78 | 797 | 0 |
| 3231 | 3561969 | 3561732 | 3561244 | - | *phzA2* | *PA14_39970* | phenazine biosynthesis protein | 176 | 1 | 5 | 20 | 9 | 134 | 0 |
[truncated: 344,427 more chars]
