# Supplementary material for: Promoter selectivity of the RhlR quorum-sensing transcription factor receptor in Pseudomonas aeruginosa is coordinated by distinct and overlapping dependencies on C4-homoserine lactone and PqsE
Source: PLoS Genet. 2023 Dec 8;19(12):e1010900. doi: 10.1371/journal.pgen.1010900 (PMC10732425; doi:10.1371/journal.pgen.1010900)
Supplement: S3 Table — (DOCX) [file pgen.1010900.s006.docx]

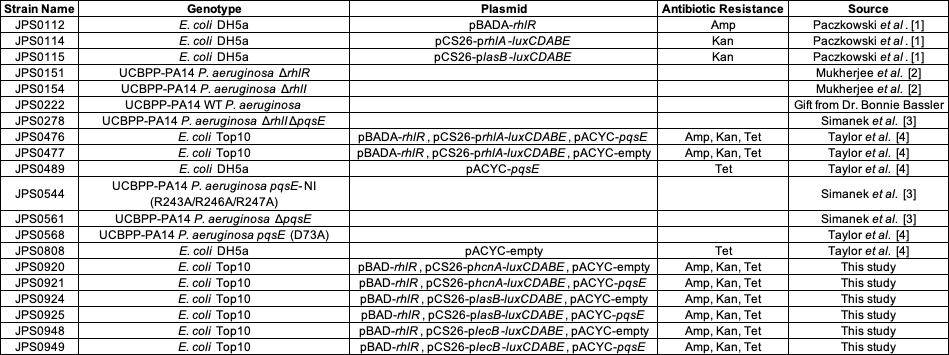


[1] Paczkowski JE, Mukherjee S, McCready AR, Cong J-P, Aquino CJ, Kim H, et al. Flavonoids suppress *Pseudomonas aeruginosa* virulence through allosteric inhibition of quorum-sensing Receptors. J Biol Chem. 2017;292. doi:10.1074/jbc.M116.770552

[2] Mukherjee S, Moustafa D, Smith CD, Goldberg JB, Bassler BL. The RhlR quorum-sensing receptor controls *Pseudomonas aeruginosa* pathogenesis and biofilm development independently of its canonical homoserine lactone autoinducer. PLoS Pathog. 2017/07/18. 2017;13: e1006504. doi:10.1371/journal.ppat.1006504

[3] Simanek KA, Taylor IR, Richael EK, Lasek-Nesselquist E, Bassler BL, Paczkowski JE. The PqsE-RhlR Interaction Regulates RhlR DNA Binding to Control Virulence Factor Production in *Pseudomonas aeruginosa*. Microbiol Spectr. 2022;10. doi:10.1128/spectrum.02108-21

[4] Taylor IR, Paczkowski JE, Jeffrey PD, Henke BR, Smith CD, Bassler BL. Inhibitor Mimetic Mutations in the *Pseudomonas aeruginosa* PqsE Enzyme Reveal a Protein–Protein Interaction with the Quorum-Sensing Receptor RhlR That Is Vital for Virulence Factor Production. ACS Chem Biol. 2021;16: 740–752. doi:10.1021/acschembio.1c00049
