# Supplementary material for: Promoter selectivity of the RhlR quorum-sensing transcription factor receptor in Pseudomonas aeruginosa is coordinated by distinct and overlapping dependencies on C4-homoserine lactone and PqsE
Source: PLoS Genet. 2023 Dec 8;19(12):e1010900. doi: 10.1371/journal.pgen.1010900 (PMC10732425; doi:10.1371/journal.pgen.1010900)
Supplement: S4 Table — (PDF) [file pgen.1010900.s007.pdf]

	A	B	C
1	**Primer Name**	**Primer Sequence**	**Description**
2	oJP967	CAACCAGAAGATCGGCAAGTA	*lasB* RT-PCR primer
3	oJP968	GTTCATGTCGACGGTGATGA	lasB RT-PCR primer
4	oJP1326	CAACACGATATCCAGCCCCT	*hcnA* RT-PCR primer
5	oJP1327	CATTGAGCACGTTGAGCACG	*hcnA* RT-PCR primer
6	oJP1328	CCTGGCCGAACATTTCAACG	*rhlA* RT-PCR primer
7	oJP1329	TTTCCACCTCGTCGTCCTTG	*rhlA* RT-PCR primer
8	oJP1330	GAGGAACTGGAAGCGGTCAA	*gyrA* RT-PCR primer
9	oJP1331	CTTCCTCGGTGATCAGGTCG	*gyrA* RT-PCR primer
10	oJP1358	ATAAGCCTCACCCCCTGTCA	*sigX* RT-PCR primer
11	oJP1359	AACGCCGCATCAATTCTTCA	*sigX* RT-PCR primer
12	oJP1436	AATGATACGGCGACCACCGAGATCTACAC	ChIP primer
13	oJP1437	CAAGCAGAAGACGGCATACGAGAT	ChIP primer
14	oJP1474	CAGGTCAACAACGAAACCGTC	*lecB* RT-PCR primer
15	oJP1475	GGCGAAGTTCAGCTCATTGG	*lecB* RT-PCR primer
16	oJP1506	ATTACAGACAGCGTAACAATCG	*lecB* promoter 220 bp fragment
17	oJP1507	TATGGCGTGTCGGGAGAGAT	*lecB* promoter 220 bp fragment
18	oJP1514	CTGGGGTAGGCGCGGGTTCGA	*hcnA* promoter 220 bp fragment
19	oJP1515	TGGATGGTCATGTCTGCCCGC	*hcnA* promoter 220 bp fragment
20	oJP1522	TTTTCATGCCTTTTCCGCCAACC	*rhlA* promoter 220 bp fragment
21	oJP1523	GGGGCTTGTGTGGGTCTTGC	*rhlA* promoter 220 bp fragment
22	oJP1592	GAGAACCACATGGGGCCTCC	*hcnA* promoter fused to luxCDABE (5'-P)
23	oJP1593	tatta ggatcc ATCCGTGAGAGAGAGTCGGG	*hcnA* promoter fused to luxCDABE (BamHI)
24	oJP1594	AGTATATTTCGAGACAATCGCCCC	*lecB* promoter fused to luxCDABE (5'-P)
25	oJP1595	tatta ggatcc GAATACCTGGCCTGCCTTTTC	*lecB* promoter fused to luxCDABE (BamHI)
