# Supplementary material for: Promoter selectivity of the RhlR quorum-sensing transcription factor receptor in Pseudomonas aeruginosa is coordinated by distinct and overlapping dependencies on C4-homoserine lactone and PqsE
Source: PLoS Genet. 2023 Dec 8;19(12):e1010900. doi: 10.1371/journal.pgen.1010900 (PMC10732425; doi:10.1371/journal.pgen.1010900)
Supplement: Attachment — Submitted filename: Review of Keegan et al..docx [file pgen.1010900.s009.docx]

Review of Keegan et al.

This manuscript is much improved. There are a few points still needing addressed to improve clarity.

1.The term “direct RhlR regulon” and “direct RhlR target genes” is still confusing in some places, for example in the abstract and in lines 206-208. It might be clearer to say “the set of genes identified by ChiP-seq in this study (Table xx)” or “the set of RhlR-occupied gene promoters” or something along those lines.

2.Line 207. Based on the response to reviews, it seems the recent RNA-seq study mentioned on this line was published, but there is no citation here. A citation is needed or a description of where the RNA-seq study came from if not published is needed for clarity.

3.Line 186 (2x), 194, 196, 210 and elsewhere. and it is important for clarity that the authors state that they compared expression of rhlR genes +/- rhlI instead of +/- C4 to reflect the experiment that was actually done. (this point was brought up in first review – reviewer #1 point 4, but only corrected in the one instance that was noted by the reviewer, and needs to be corrected throughout this section).

4.Line 288 (now line 308-309). A heterologous E. coli system is not “equivalent” or even “somewhat equivalent” to an in vitro system. A more precise way to say this could be that the heterologous system enables studies of regulatory effects in the absence of other regulators, and therefore provides insight into whether there are direct interactions with the gene promoter.

5.Consider changing “promoter selectivity” to “promoter occupancy” in the title to more precisely reflect how the study was carried out.
