# Supplementary material for: Promoter selectivity of the RhlR quorum-sensing transcription factor receptor in Pseudomonas aeruginosa is coordinated by distinct and overlapping dependencies on C4-homoserine lactone and PqsE
Source: PLoS Genet. 2023 Dec 8;19(12):e1010900. doi: 10.1371/journal.pgen.1010900 (PMC10732425; doi:10.1371/journal.pgen.1010900)
Supplement: Attachment — Submitted filename: Keegan et al Response to Reviewers.docx [file pgen.1010900.s010.docx]

Reviewer #1: This manuscript presents a valuable dataset to the field of bacterial cell-cell signaling and the revisions have significantly improved the clarity of the authors’ analysis. The authors should consider the following minor revisions to further improve clarity in their discussion of the data.

1) Line 191 – there appears to be a misplaced reference in the middle of the sentence ([59]).

We thank the reviewer for catching this oversight. We have removed the misplaced reference.

2) Lines 297-298: The authors state for hcnA and lecB qPCR experiments, “maximal expression was dependent on the presence of PqsE as the ∆rhlI strain had higher expression levels for both genes than the ∆rhlI∆pqsE strain”. At 25 uM C4-HSL, the strains appear to have similar transcript levels, especially given the error bars. What is the difference in expression level and is it consequential?

We have addressed the issue by qualifying our above statement with “although the differences are not statistically significant in every instance (p > 0.05), especially at the highest C_4_HSL concentration” on lines 313-314. We also include t-tests to compare the effects of +/- *pqsE* at the different concentrations of C_4_HSL.

3) Could the authors provide more guidance on interpretation of Table 1? Specifically what does “RhlR expression change, PqsE expression change, etc.” refer to? It would seem it is the RNASeq transcript level in WT compared to the deletion strain, but it is unclear how the numbers in the table translate to analysis in the text.

a. For example, on line 263, the authors state “the effect of deleting pqsE on the expression of hcnA was similar to the effect of deleting rhlR”. But in Table 1, the expression change for hcnA is 10.2 and 3.6 for RhlR and PqsE, respectively. As such, it appears the effect of deleting rhlR is much greater than the effect of deleting pqsE. This is consistent with the qPCR data in 5A where ∆pqsE has a much higher transcript level than ∆rhlR.

b. Similarly, on line 270, the authors state “the effect of deleting pqsE on expression of PA14_01490 was only 30% the effect of deleting rhlR”. The values for RhlR expression change and PqsE expression change are 18.4 and 1.4, respectively. How does that translate to the effect of pqsE being 30% that of rhlR?

Values in Table 1 for RNA-seq data represent fold-change versus wild-type. Values plotted in Figures 3C and 4C show the effect of deleting *rhlI*/*pqsE* on RNA levels relative to that of deleting *rhlR*. If we consider *PA14_01490*, normalized RNA levels in wild-type, Δ*rhlR*, and Δ*pqsE* strains are 2956, 160, and 2108, respectively. Hence, the ratios of RNA levels for wild-type to Δ*rhlR* and wild-type to Δ*pqsE* strains are 18.4 and 1.4, respectively. However, if we consider the absolute difference in RNA level from wild-type to Δ*pqsE* strains, the number is 848, as compared to 2796 for wild-type to Δ*rhlR*, i.e., 30%. Similarly, the absolute difference in RNA levels for *hcnA* between Δ*pqsE* and wild-type cells is 80% of the difference between Δ*rhlR* and wild-type cells, despite the fold-change numbers being rather different. This information is now included in the Methods section.

Reviewer #2:

Review of Keegan et al.

This manuscript is much improved. There are a few points still needing addressed to improve clarity.

1.The term “direct RhlR regulon” and “direct RhlR target genes” is still confusing in some places, for example in the abstract and in lines 206-208. It might be clearer to say “the set of genes identified by ChiP-seq in this study (Table xx)” or “the set of RhlR-occupied gene promoters” or something along those lines.

We use this terminology to highlight the genes that have an upstream RhlR binding site and are differentially expressed in a Δ*rhlR* strain relative to wild-type cells. To help clarify this point, we have made the following changes:

In the abstract, we changed “to map the direct RhlR regulon” to “to map the binding of RhlR across the *P. aeruginosa* genome”.

On line 123, we changed “comprehensively map the direct RhlR regulon” to “comprehensively map genes that are directly regulated by RhlR”.

On line 213, we changed “expression of direct RhlR target genes” to “expression of directly RhlR-regulated genes”.

2.Line 207. Based on the response to reviews, it seems the recent RNA-seq study mentioned on this line was published, but there is no citation here. A citation is needed or a description of where the RNA-seq study came from if not published is needed for clarity.

We added a reference to our previously published work at this location.

3.Line 186 (2x), 194, 196, 210 and elsewhere. and it is important for clarity that the authors state that they compared expression of rhlR genes +/- rhlI instead of +/- C4 to reflect the experiment that was actually done. (this point was brought up in first review – reviewer #1 point 4, but only corrected in the one instance that was noted by the reviewer, and needs to be corrected throughout this section).

We changed all instances of “+/- C_4_HSL” to “+/- *rhlI*” to more accurately reflect the experiment being conducted.

4.Line 288 (now line 308-309). A heterologous E. coli system is not “equivalent” or even “somewhat equivalent” to an in vitro system. A more precise way to say this could be that the heterologous system enables studies of regulatory effects in the absence of other regulators, and therefore provides insight into whether there are direct interactions with the gene promoter.

We appreciate the reviewer’s view on the matter and their helpful suggestion to provide clarity. We have modified the text to state, “we view the *E. coli* heterologous reporter system as a way to study the regulatory effects at different promoters in the absence of other regulators, permitting us to draw inferences about the direct effects of the factors being tested” on lines 325-7.

5.Consider changing “promoter selectivity” to “promoter occupancy” in the title to more precisely reflect how the study was carried out.

Our data report on selectivity more than they do on occupancy. This is in part because of the challenges in using ChIP-seq data to infer absolute occupancy (see lines 184-8 and 225-8), and also because we include RNA-seq data in our analyses. We will defer to the opinion of the editorial staff.

Reviewer #3:

The revised manuscript titled "Promoter Selectivity of the RhlR Quorum-Sensing Transcription Factor Receptor in Pseudomonas aeruginosa Is Coordinated by Distinct and Overlapping Dependencies on C4-Homoserine Lactone and PqsE," authored by Keegan and colleagues, represents a significant improvement over the original version. The authors have addressed the specific comments raised by the reviewers, including my own, resulting in a notably clearer manuscript that enhances the reader's understanding of the results. However, the authors have chosen to retain the original structure of the manuscript. Consequently, despite the improvements, the revised version still exhibits some structural imbalances, as previously noted. I still have some minor comments.

They should address the title to incorporate the term "promoter occupancy," which the authors have adopted. As the core of the manuscript relies on ChIP-Seq analysis, it is also advisable for the authors to address the limitations they themselves have acknowledged regarding the constraints of the technique.

Our data report on selectivity more than they do on occupancy. This is in part because of the challenges in using ChIP-seq data to infer absolute occupancy (see lines 184-8 and 225-8), and because we include RNA-seq data in our analyses. We will defer to the opinion of the editorial staff.

In the revised version, C4-HSL concentration for figure 6 was changed from 1 µM to 4 (it was also changed in the method section). The authors justified to reviewer 1 that they based their use of 1 µM to what they found in the literature. There is no explanation as why the concentration was changed, and the figures are the same. Which concentration is the right one?

There was an oversight when calculating the final concentrations used during our dilution series from which we used a single data point for ease of viewing the data. 4 µM is the correct concentration. We apologize for not noting this during the resubmission process.

Figure 5 (A and C) should still show signs of statistical analyses as was requested by reviewers.

For Figure 5A, we now include a one-tailed t test p-values for claims on lines 282 and 283 and a two-tailed t-test p-value for claim on line 284, as these are the relevant comparisons we make in the manuscript. We now change “substantially” to “significantly” when the p-value is less than 0.05.

For Figure 5C, we now depict our data into three separate graphs as subpanels to compare *rhlA, hcnA*, and *lecB* expression between Δ*rhlI* and Δ*rhlIpqsE* mutants. We do this for the ease of making and displaying comparisons. We performed multiple paired t tests for all three data sets and show the comparisons between Δ*rhlI* and Δ*rhlIpqsE* at the different concentrations of C_4_HSL.

This is only minor but:

Table S3: even if the WT strain is listed as being PA14 in the table, it should be mentioned for each mutant that PA14 is the background. Also the numbers from the first column don’t line up start from 2 ?

We apologize for this oversight. There was an issue arising from our conversion to a PDF. We now indicate that every *P. aeruginosa* strain is from the PA14 background. We’ve re-built Table S3 as Word document and include that with our final submission.

Strain 21 does not have a strain number ?

We apologize for this oversight. There was an issue arising from our conversion to a PDF. We’ve re-built Table S3 as Word document and include that with our final submission.
