# Supplementary material for: Promoter selectivity of the RhlR quorum-sensing transcription factor receptor in Pseudomonas aeruginosa is coordinated by distinct and overlapping dependencies on C4-homoserine lactone and PqsE
Source: PLoS Genet. 2023 Dec 8;19(12):e1010900. doi: 10.1371/journal.pgen.1010900 (PMC10732425; doi:10.1371/journal.pgen.1010900)
Supplement: Attachment — Submitted filename: Reviewer Comments Keegan et al. PLOS Genetics.pdf [file pgen.1010900.s008.pdf]

Dear editors,

Thank you again for handling our manuscript. We are grateful to you and to the three referees for their careful reading, their insight, and their efforts to help us improve the work. We have addressed all of their concerns in the revised manuscript. In brief, we edited the abstract to better align our conclusions in the abstract with those of the rest of the manuscript; we edited the conclusions to provide more precision and clarity to our interpretation; and we performed additional statistical tests to address concerns about significance (for example, Figure 6A). Below, we provide our point-by-point responses (in red text) to all referee remarks. Referee text is in black. We hope you now find the manuscript acceptable for publication.

Comments to the Authors:

Reviewer #1: The pathogen Pseudomonas aeruginsoa regulates hundreds of genes through its interconnected quorum sensing (QS) systems. Of particular importance is the LuxR-type quorum sensing receptor RhlR, which is involved in the regulation of several key virulence factors. To-date, a direct RhlR regulon has been elusive, with evidence for RhlR gene regulation limited to RNA-Seq studies and therefore to genes that are both directly and indirectly regulated by RhlR. Here, the authors use ChIP-Seq to determine regions of DNA directly bound by RhlR and to illuminate three distinct categories of RhlR-bound promoters: 1) those that require the cognate signal, C4-HSL, 2) those that require the protein PqsE, and 3) those that require both C4-HSL and PqsE for binding. The authors go on to correlate RhlR binding at these sites with gene expression in various genetic mutants. The authors should be commended on a well-written and rigorous study of the RhlR regulon and the influence of PqsE on RhlR activity. Notably, the authors perform their experiments in P. aeruginosa PA14 that is WT or has specific genes deleted. Given the known impact of receptor overexpression on both signal sensitivity and signal-independent gene activation, it is significant that the authors conducted their experiments in strains that preserve native regulation of RhlR expression. I have only minor comments on what is sure to be a broadly interesting and important contribution to the fields of cell-cell signaling and gene regulation.

1) In the abstract (lines 34-35) the authors state RhlR binds PqsE-dependent sites and “regulates transcription from [them] independent of the presence of autoinducer”. This is somewhat misrepresentative of the data presented. Even for genes where C4-HSL does not affect binding, the authors “still observed a substantial requirement for C4-HSL for regulation by RhlR (line 204) and “C4-HSL is required for RhlR to maximally activate transcription” from these genes (line 206). It would be accurate to state RhlR can activate transcription from the genes without C4-HSL, but C4-HSL still plays a role in their regulation.

We have extensively edited the abstract to correct this and other inconsistencies.

2) In the abstract (line 36) the authors state the effect of C4-HSL on binding does not correlate with its role in regulating RhlR-dependent gene expression, but on line 200, the authors state C4-HSL-dependent changes in gene expression correlate positively with C4-HSL dependent changes in RhlR binding. These appear to be opposite conclusions. In figure 3C, the relationship between C4-dependent binding and gene expression seems flat or only weakly positive. The authors should provide a quantitative metric for the correlation (or lack thereof) along with its statistical significance.

See response to previous comment.

3) Line 137 – It would be of interest for the authors to briefly explain how the RhlR binding motif compares to lux-box sequences for other receptors.

We have added text to address this issue. On lines 156-158, we now state: “This sequence motif matches the known RhlR binding consensus, which was previously determined using promoter sequences from the core regulon; this motif is similar to those of other LuxR-type family proteins” and provide a citation.

4) Line 197 – It would be clearer for the authors to state they compared expression of direct RhlR target genes +/- rhlI to identify C4-dependent changes.

We replaced “+/- C_4HSL” with “+/- *rhlI*” in the text on line 227 to improve clarity.

5) Figure 4C – It would be useful to provide a quantitative metric of correlation in PqsE dependence that could be compared to C4-dependencies shown in Fig 3C.

For Figures 3C and 4C, we performed a simple linear regression model to determine a best-fit line. We now include the R^2 and *p*-values on the graph, which indicate statistical significance.

6) Line 271 – The authors state the ChIP-Seq analysis of RhlR in a ΔrhlIΔpqsE strain results in nearly same decrease in binding occupancy as ΔrhlR and reference Fig 5B, which shows a comparison of ΔrhlIΔpqsE and WT enrichment. It would be helpful to include a comparison of ΔrhlIΔpqsE and ΔrhlR to justify the claim that they have “nearly the same decrease in binding occupancy”. This could be a supplemental figure.

We have included a new supplemental figure (Figure S3) to depict the combined contributions of *rhlI* and *pqsE* to RhlR binding across all sites.

7) It would be helpful for the authors to include statistical analyses of key comparisons, particularly in Fig 5C where some differences in expression level seem minor or have large errors.

The important conclusion from this figure is the relative difference between the effects of the loss *pqsE* on different genes.

8) In figure 6, the authors use 1 uM C4-HSL to stimulate the activity of RhlR heterologously expressed in E. coli. Is this a sufficient concentration to stimulate maximal activity for each of the reporters? It seems unlikely as published EC50 values for RhlR expressed in E. coli range from 9 - 120 uM (PMID 30114353, 26460240, 30837333). The authors should justify their choice of 1 uM C4-HSL in this experiment and consider repeating the experiment with a higher concentration of C4-HSL.

We have addressed this concern in the manuscript on lines 343-345 and included a citation of previous work done at this concentration to examine the effects of PqsE on RhlR activity.

Relatedly, is it possible “C4-independent” genes simply require a higher concentration of C4-HSL than provided, or produced in these experiments? This may be true in both the E. coli

studies and in P. aeruginosa where C4 production in planktonic culture could be less than that in biofilms or in other environments. Perhaps PqsE stabilizes RhlR at low concentrations of C4-HSL but at higher concentrations, the impact of PqsE is lower or even negligible?

Our data and data from earlier studies support this model. Over the course of growth, we expect that cells will be exposed to different concentrations of C_4HSL. We consider the implications of this in the discussion.

9) Line 518 – What was the OD of the cultures when collected for qPCR? The authors state "until the optimal cell density was achieved" without giving a specific number.

We apologize for this oversight. We have included this information in the material and methods.

Reviewer #2: This study describes a comprehensive Chip-Seq analysis of the regulon of P. aeruginosa quorum sensing regulator RhlR. The study also looks at how the RhlR-binding protein PqsE influence RhlR promoter occupancy from in vivo CHIP-seq studies. The results map the set of gene promoters that RhlR occupies in the presence and absence of its ligand C4-HSL and a regulator protein PqsE.

Global studies of RhlR binding have not been previously carried out. Thus, the studies provide important new insight into RhlR and P. aeruginosa quorum sensing biology. The finding that RhlR promoter occupancy is altered by interacting with PqsE also represents a newly described mechanism of transcription regulation and is very interesting.

The paper is very clearly written and has a very appealing systems-level approach. However, there are some issues with clarity of writing and presentation that need to be addressed before the manuscript is ready for publication.

Major criticisms

1. The Chip-seq results using natively expressed RhlR show that PqsE alters the promoter occupancy of RhlR but a limitation of this approach is that there are a variety of mechanisms that could alter RhlR promoter occupancy. Care should be taken in drawing conclusions from these results, for example by replacing terms such as "PqsE alters RhlR promoter selection" with "PqsE alters promoter occupancy."

We have decided to use the terminology "selective RhlR promoter occupancy" because it addresses the reviewer's comment, and highlights that the effect of PqsE is different at different promoters.

2. There are some problems with the figures that need addressed. The figure headings are not informative and in some cases do not reflect what is seen as the main point of the figure. In some cases the figures are also difficult to interpret or lack critical information. See below for specific examples.

3. The discussion, particularly the first paragraph, needs revision. In particular, many of the statements are not clear and there are some issues with the conclusions being drawn – see below for specific examples.

We address the specific criticisms below.

Specific criticisms

1. Fig. 2 needs clarification. First, the heading is not accurately describing the data. The data are more related to which genes from the Chip-seq study correlate with the RhlR-regulated genes from RNAseq. Also, it seems you used some sort of cutoff (log2 of 2?) to decide which genes were transcriptionally induced by RhlR and which weren't, where this cutoff is should be indicated on the graph or at least described in the legend.

We have changed the title of Figure 2. We have included the cut off (*q*-value) for defining RhlR-dependent regulation in the figure legend. We also now indicate using different colors which of the genes are direct RhlR targets.

2. Fig. 3C – this figure is intuitively difficult to understand. Also, the text on lines 200-202 states that there is a positive correlation between C4-dependent changes in gene expression and RhlR binding, but this is not clear from the data, and there is no positive correlation line or statistics to support this statement. Also, based on the rest of description in the results, and looking at the data, it seems that the point is actually that RhlR does not necessarily need C4-HSL for binding but it does need it to activate. That's not really reflective of a positive correlation.

For Figures 3C and 4C, we performed a simple linear regression model to determine a best-fit line and included the R^2 and *p*-values on the graph, which indicate statistical significance.

3. Fig. 4 – no correlation line or statistics to support the conclusion that there is a correlation. In addition, the discussion of this data in lines 246-255 seems to be highlighting the variability (although the example on lines 245-247 was difficult to follow), and not the correlation.

We agree with the reviewer that the text was confusing around the interpretation of these data. We have clarified our assertions in the text that while the data are largely positively correlated, some genes deviate from this trend stating on line 292 "Despite the positive correlation between the dependence on PqsE for RhlR binding and activation, the expression of some genes was largely independent of PqsE notwithstanding a strong dependence of RhlR binding on PqsE."

4. Fig. 6A – it would be helpful to see these data in their raw form (+ and – C4-HSL, so that an evaluation of the relative levels of induction can be taken into consideration. In addition, statistical comparisons are needed to support the conclusions.

We agree that these data are best visualized by displaying the data in their raw form. We have modified Figure 6A to accommodate these changes.

5. Fig. 6B – because EMSA experiments cannot be conducted in +/- C4-HSL conditions due to technical limitations, it is unclear whether PqsE is altering the ability of RhlR to respond to C4 or simply increasing its promoter affinity. The data in Fig. 6A do seem to support the idea that it is the former and not the latter (although visualizing the data in its raw form would help with that interpretation). Also, in absence of promoter binding affinity calculations, it is difficult to determine if the promoter affinity is altered in the same way for each of the genes (in which case PqsE would be altering the promoter selectivity and not just affinity). Without the EMSA +/- C4 data and a

rigorous study of promoter binding affinities with several different promoters, care should be taken in describing the interpretation of the Chip-Seq data (see major point #1 above and point #6 below).

RhlR is purified in the presence of the agonist and PqsE is added to RhlR in the EMSA reaction. Thus, PqsE cannot alter the ability of RhlR to respond to C_4HSL. We agree with the reviewer that the affinity for different sites is likely to be different, and we note this in the manuscript on line 359.

6. Lines 154-155. The wording here suggests the RNAseq study used for this analysis was published previously, but upon closer examination of the methods it appears that those experiments were done for this study. This section needs to be rewritten to clarify that where the RNAseq results came from. Also, it appears that at least one other RNAseq study focused on PqsE is in the published literature (ref. 42). A comparison of the approach and differences in results of each study should be discussed either here or in the discussion.

We have clarified in the methods section that the RNA-seq data are from our earlier paper, but we note that our analysis of those data in our current study is new. We believe that lines 376-377 address this sufficiently.

7. Line 288 – the heterologous E. coli reporter system is not equivalent to an in vitro system because it is not in vitro.

We agree that it is not an "*in vitro*" system, but since *E. coli* does not produce RhlR, PqsE, or C_4HSL, it can be considered as a proxy for *in vitro* assays. There might be other RhlR co-factors present in *E. coli* that we are unaware of, so we have qualified the statement with "somewhat equivalent."

8. How were the DNA probes in the EMSA experiments detected? It appears they were detected by fluorescence. This needs to be clarified in the methods.

We modified the methods to include the wavelength for the detection of SYBR Gold.

Relatively minor:
Line 191: it doesn't seem like there should be a dash between "C4-HSL" and "dependent"

We are not sure how to address this comment because it depends on the editorial style of the journal and believe it is a question best left to the editorial staff.

The paragraph starting at line 222 is not clear. I think what it is trying to say is that several new genes have been identified that have robust effects of PqsE, but that is not clear.

We have edited the second sentence of this paragraph on line 244 to make the text clearer.

Lines 258-260: please remind the reader where the data are to support this statement – ("ChIP-seq and RNA-seq data showed that RhlR binding and RhlR-dependent transcription activation of rhlA and lasB are more dependent on C4HSL than PqsE, whereas the opposite is true for hcnA and lecB.")

We now refer to Table 1 to remind the reader of the location of the data.

Specific examples of problems in the discussion that needs addressed (just from the first paragraph) are below -
- Line 306: “the direct RhlR regulon” is an unclear term

Addressed (see response to comment below about lines 311-312).
- Line 307: “RhlR-dependent changes in RNA levels” is also unclear - do you mean RhlR-dependent changes in gene transcription?

Addressed.
- Line 308: This sentence could be softened – perhaps “provides insight into direct vs. indirect regulation”

Addressed.
- Line 310: This section is unclear. Having RhlR binding sites without evidence of regulation does not necessarily support condition-specific regulation. Perhaps what was meant was that some genes are RhlR regulated only under some conditions?

We do not understand this comment, as we think the reviewer’s suggestion is already described in the discussion.

- Line 311-312: what is “direct RhlR target genes” – is this meant to be “genes directly regulated by RhlR”?

We clarify this statement in the discussion and on lines 374-377, we state: “This is the first comprehensive assessment of the direct RhlR regulon (i.e., genes where regulation by RhlR is due to RhlR binding upstream).”

- Line 314 – it is not clear how the RNAseq study in ref. 42 is different or how the results compared with those in this study. Also, why do the prior RNAseq studies suggest that most of the regulon is indirectly controlled by RhlR? Don’t your results also suggest this?

We agree, and now make a general statement about direct vs indirect regulation by RhlR, rather than referring specifically to reference 42.

Reviewer #3: The manuscript entitled “Promoter selectivity of the RhlR quorum-sensing transcription factor receptor in Pseudomonas aeruginosa is coordinated by distinct and overlapping dependencies on C4-homoserine lactone and PqsE” by Keegan and collaborators aims to enlighten the current comprehension of the quorum sensing (QS) network in the bacterium P. aeruginosa. The authors performed ChIP-Seq analyses of the transcriptional regulator RhlR to decipher the role of the cognate autoinducer C4-HSL and PqsE in DNA binding. The significance of this work lies in its ability to unravel the direct contributions of RhlR to the QS network, thereby advancing our understanding of this crucial communication system. Despite the intriguing nature of the provided data, the manuscript suffers from a lack of overall cohesion in its structural organization, hampering the potential of the findings.

Overall remarks:
The manuscript contains crucial data for individuals interested in studying gene regulation and quorum sensing of the opportunistic pathogen P. aeruginosa. However, to enhance its impact and clarity, some structural revisions are essential. It is imperative for the authors to establish a primary focus for the manuscript and then reorganize its content accordingly. The current arrangement lacks coherence, leading to confusion even among experts in the field.

Certain sections have been disproportionately emphasized, such as Figures 5 and 6, while novel findings (ChIP-Seq results in the double rhlI and pqsE mutant), have not received adequate attention. These imbalances should be rectified in the revised version of the manuscript.

Major comments:
The Abstract contains a couple of statements that are not in line with the data. By reading it, (1) it is suggested that the binding of PqsE with RhlR changes the affinity of the latter for its binding sites – implying a distinct consensus region for the different forms of RhlR. (2) The transcription of these regions is not induced by the presence of C4-HSL. (3) C4-HSL is generally required for the RhlR DNA binding. None of those are supported by the data presented in the manuscript.

See response to Reviewer 1.

The manuscript is based on multiple analyses of ChIP-Seq. Those were designed to have the parallel analyses in the rhlR mutant as control. In accordance with the experimental design, the authors attribute 40 regions to be true RhlR-bound regions. As also mentioned by the authors, multiple genes coding known transcriptional regulators have associated RhlR binding sites (ln 166-167). Taking the experimental design into consideration, and considering the lack of specificity of the polyclonal antibody anti-RhlR, the authors should address the limitations.

We address this concern in the manuscript by stating on lines 606-607: “We expect that these only include sites that are associated with RhlR binding, but it is formally possible that the antibody cross-reacts with proteins whose abundance is dependent upon RhlR.”

Overall, there is a lot of confusion in how the manuscript is written and it should be reworked for clarity.

Ln 211-212: It is stated: “We speculated that PqsE can assist RhlR binding to some sites in the absence of C4HSL, which would explain the variable dependence of different RhlR binding sites for C4HSL.”
However, this paragraph does not adresse this as it focuses on data in the pqsE mutant in presence of C4HSL. They did perform analyses in the double pqsErhlI mutant but these data are not discussed here.

We have modified the text on line 244 here to address the reviewers’ comment. We have also included a new supplemental figure (Figure S3) to depict the combined contributions of *rhlI* and *pqsE* to RhlR binding across all sites.

Ln 237: “Consistent with our prior study, binding of RhlR in cells expressing pqsE D73A was very similar to that in WT cells (Figure S2A), and binding of RhlR in cells expressing pqsE-NI was very similar to that of ΔpqsE cells (Figure S2B), consistent with the overlapping expression profiles of both pqsE D73A with WT (Figure S2C) and pqsE-NI with ΔpqsE (Figure S2D) [29,47].”

Very similar in what way? Avoid vague qualification of data. Is the difference significant or not ?

We now include R^2 values for the relevant comparisons.

Ln 281: "To directly assess the role of each of the regulators in gene expression, we compared the effect of C4HSL and PqsE on RhlR-dependent transcription activation of rhlA, lasB, hcnA, and lecB using luciferase reporter fusions in Escherichia coli expressing either rhlR or rhlR and pqsE, +/- C4HSL (Figure 6A) [29,47,59]. "

The use of each of the regulators here is confusing…the only regulator listed here is RhlR.

We have clarified this statement on lines 340.

Related to Figure 5: Based on the data presented, the authors seem to suggest a complementary role of C4-HSL and PqsE to RhlR functioning. The latter seems to be required for the formation of a DNA binding complex (as PqsE does not greatly alter the transcriptional profile but is required for RhlR binding) whilst the former induces its transcriptional activity (C4-HSL induces the transcription of genes that do not require this AI for binding). Can that assumption be generalized for most of the RhlR binding sites examined in this manuscript? And if so, can the authors propose a model in which RhlR binds to and activates its target genes? In line with that, have the authors observed the binding of RhlR to its regulated sites in the absence of both C4-HSL and PqsE? Knowing whether the inactivated RhlR can bind to DNA in the absence of PqsE would help us further understand the steps required for RhlR activation.

We believe this is addressed with the new supplementary figure (Figure S3).

Also, the authors infer three distinct modes of activation by RhlR in the Abstract. One of them states that RhlR can be active when bound to PqsE only. However, although this complex can bind to DNA, the transcription of most targets seems to be C4-HSL-dependent. Considering this, is the complex RhlR:PqsE really active?

Figure 3C shows that there is some activity of the RhlR:PqsE complex at certain sites in the absence of C_4-HSL.

Ln 325-330. The logic used by the authors in this section confuses me. It is stated that C4-HSL is required for efficient transcription activation (thus, probably inducing a conformational change that stabilizes RhlR). However, the conclusion is that RhlR can be stable and functional without the presence of the ligand.

We have modified the text in the discussion to clarify this point by stating on lines 404-406: "These findings indicate that RhlR can be stable without a small molecule ligand in *P. aeruginosa*, which is not the case in *E. coli* overexpression systems."

Minor comments:

Ln 85: Pseudomonas should be in italic
Ln 108: "For example, the rhamnolipid synthase, rhlA," should be the gene coding for the rhamnolipid synthase or RhlA.
Ln 110: "Conversely, the hydrogen cyanide synthase, hcnA, Same comment: the synthase refers to the protein and should be written as HcnA.
Ln 161. Which RhlR-regulated transcript is negatively regulated by RhlR?

Ln 226. Typo in gene name; replace by PA14_30840.
Table S3: complete reference for strains needed and P. aeruginosa strain background should be mentioned.

We thank the reviewer for catching these oversights and we have revised the text at those locations. We have also modified Table S3 to include complete references and the PA14 background strain.

Please note that we have included one additional change to the manuscript. In the course of our revision, we realized that our description of the ChIP-seq data analysis was not sufficiently precise. We now note that comparing RhlR ChIP-seq data across strains reports on the relative occupancy of RhlR at its different DNA sites. We believe the ChIP-seq data also provide an accurate comparison of the absolute occupancy of RhlR at different sites between strains, but in the absence of a spike-in control we cannot be certain of this. This is a nuanced point that may be of interest to ChIP-seq afficionados and does not impact the conclusions of the paper. Rather than including a lengthy discussion of this complex topic, we cite a reference that describes ChIP-seq data analysis and the application of spike-in controls in much greater detail.
